# GLP-1-mediated delivery of tesaglitazar improves obesity and glucose metabolism in male mice

**DOI:** 10.1038/s42255-022-00617-6

**Published:** 2022-08-22

**Authors:** Carmelo Quarta, Kerstin Stemmer, Aaron Novikoff, Bin Yang, Felix Klingelhuber, Alex Harger, Mostafa Bakhti, Aimee Bastidas-Ponce, Eric Baugé, Jonathan E. Campbell, Megan Capozzi, Christoffer Clemmensen, Gustav Collden, Perla Cota, Jon Douros, Daniel J. Drucker, Barent DuBois, Annette Feuchtinger, Cristina Garcia-Caceres, Gerald Grandl, Nathalie Hennuyer, Stephan Herzig, Susanna M. Hofmann, Patrick J. Knerr, Konxhe Kulaj, Fanny Lalloyer, Heiko Lickert, Arek Liskiewicz, Daniela Liskiewicz, Gandhari Maity, Diego Perez-Tilve, Sneha Prakash, Miguel A. Sanchez-Garrido, Qian Zhang, Bart Staels, Natalie Krahmer, Richard D. DiMarchi, Matthias H. Tschöp, Brian Finan, Timo D. Müller

**Affiliations:** 1 Institute for Diabetes and Obesity, Helmholtz Zentrum München, Neuherberg, Germany; 2 German Center for Diabetes Research (DZD), Neuherberg, Germany; 3 University of Bordeaux, INSERM, Neurocentre Magendie, Bordeaux, France; 4 Molecular Cell Biology, Institute for Theoretical Medicine, University of Augsburg, Augsburg, Germany; 5 Division of Metabolic Diseases, Department of Medicine, Technical University of München, Munich, Germany; 6 Novo Nordisk Research Center Indianapolis, Indianapolis, IN USA; 7 Institute of Diabetes and Regeneration Research, Helmholtz Zentrum München, Neuherberg, Germany; 8 Inserm, CHU Lille, Institute of Pasteur de Lille, European Genomic Institute for Genomics, University of Lille, Lille, France; 9 Department of Medicine, Division of Endocrinology, Duke University, Durham, NC USA; 10 Novo Nordisk Foundation Center for Basic Metabolic Research, Faculty of Health and Medical Sciences, University of Copenhagen, Copenhagen, Denmark; 11 Lunenfeld-Tanenbaum Research Institute, Mount Sinai Hospital, University of Toronto, Toronto, ON Canada; 12 Research Unit Analytical Pathology, Helmholtz Zentrum München-German Research Center for Environmental Health, Neuherberg, Germany; 13 Institute for Diabetes and Cancer, Helmholtz Diabetes Center, Helmholtz Center Munich, Neuherberg, Germany; 14 Medical Clinic and Polyclinic IV, Ludwig-Maximilians University of München, Munich, Germany; 15 Department of Pharmacology and Systems Physiology, University of Cincinnati College of Medicine, Cincinnati, OH USA; 16 Department of Cell Biology, Physiology and Immunology, Faculty of Medicine, University of Córdoba, Córdoba, Spain; 17 Department of Chemistry, Indiana University, Bloomington, IN USA; 18 Helmholtz Zentrum München, Neuherberg, Germany

**Keywords:** Type 2 diabetes, Pharmacology, Obesity

## Abstract

Dual agonists activating the peroxisome proliferator-activated receptors alpha and gamma (PPARɑ/ɣ) have beneficial effects on glucose and lipid metabolism in patients with type 2 diabetes, but their development was discontinued due to potential adverse effects. Here we report the design and preclinical evaluation of a molecule that covalently links the PPARɑ/ɣ dual-agonist tesaglitazar to a GLP-1 receptor agonist (GLP-1RA) to allow for GLP-1R-dependent cellular delivery of tesaglitazar. GLP-1RA/tesaglitazar does not differ from the pharmacokinetically matched GLP-1RA in GLP-1R signalling, but shows GLP-1R-dependent PPARɣ-retinoic acid receptor heterodimerization and enhanced improvements of body weight, food intake and glucose metabolism relative to the GLP-1RA or tesaglitazar alone in obese male mice. The conjugate fails to affect body weight and glucose metabolism in GLP-1R knockout mice and shows preserved effects in obese mice at subthreshold doses for the GLP-1RA and tesaglitazar. Liquid chromatography–mass spectrometry-based proteomics identified PPAR regulated proteins in the hypothalamus that are acutely upregulated by GLP-1RA/tesaglitazar. Our data show that GLP-1RA/tesaglitazar improves glucose control with superior efficacy to the GLP-1RA or tesaglitazar alone and suggest that this conjugate might hold therapeutic value to acutely treat hyperglycaemia and insulin resistance.

## Abstract (web summary)

A conjugate drug consisting of GLP-1 receptor agonist and the PPARɑ/ɣ dual-agonist tesaglitazar is shown to have superior anti-diabetic effects than monotherapy.

## Main

The peroxisome proliferator-activated receptors alpha and gamma (PPARɑ/ɣ) play important roles in the regulation of energy, lipid and glucose metabolism^1^. PPARs are nuclear transcription factors that on ligand activation heterodimerize with the 9-*cis* retinoic acid receptor (RXR) to promote target gene expression via binding to DNA response elements^2^. While selective activation of PPARɑ by fibric acid derivates (fibrates) primarily improves hepatic lipid and cholesterol metabolism^3^, the selective activation of PPARɣ by thiazolidinediones (TZDs) enhances insulin sensitivity in peripheral tissues such as the adipose tissue, liver and skeletal muscle^4^. Although predominantly expressed in the adipose tissue^4^, PPARɣ messenger RNA and immunoreactivity has also been detected in hypothalamic nuclei governing energy balance^5^, and third ventricle adenoviral-mediated overexpression of PPARɣ decreases food intake in lean and diet-induced obese (DIO) mice^6^. Inhibition of food intake by hypothalamic PPARɣ activation is, however, not undisputed since hypothalamic viral-mediated overexpression of PPARɣ, or third ventricle administration of the PPARɣ agonist Rosiglitazone, has also been shown to increase food intake and body weight in rats^7^. Moreover, systemic activation of PPARɣ increases food intake and body weight in rodents^8^ and neuron-specific deletion of PPARɣ decreases body weight and food intake in mice fed a high-fat diet^9^. Fibrates and TZDs have both shown beneficial cardiovascular effects in clinical trials^3,10,11^, which together with their complementary action on glucose and lipid metabolism spurred the development of PPARɑ/ɣ dual agonists (Glitazars) for the treatment of type 2 diabetes (T2D) and dyslipidaemia^1^. These agents effectively improved glucose and lipid metabolism in clinical trials^12,13^. However, although TZDs can have cardiac benefits in selected patient cohorts^14^, the development of many Glitazars was terminated due to potential unfavourable effects on the cardiovascular and/or renal system^15^. Adverse effects of Glitazars may include myopathy and muscle catabolism, fluid retention, renal damage, weight gain, peripheral oedema and early indications of an increased cardiovascular risk^16^.

Tesaglitazar is a PPARɑ/ɣ dual-agonist, which in phase II and III clinical trials improved glucose and lipid metabolism with greater efficacy relative to selective PPARɣ agonism^13,17–21^. Unfavourable effects of tesaglitazar are dose dependent and may include undesired weight gain, increased serum levels of creatinine and decreased glomerular filtration^13,17,19,20^. In 2006, the development of tesaglitazar was terminated on the basis of an estimated benefit/risk profile that was not expected to be superior to existing therapies.

Here we report the preclinical pharmacological evaluation of a single molecule conjugate of tesaglitazar covalently attached to a biochemically optimized non-acylated glucagon-like peptide-1 analogue (GLP-1 Aib2 Glu16 CEX Lys40). We proposed that the insulinotropic effect of this GLP-1R agonist (GLP-1RA) would synergize with the insulin-sensitizing effect of tesaglitazar to optimize glucose handling, while the body weight lowering effect of the GLP-1RA would buffer against any potential obesogenic activity of PPARɣ agonism. We further proposed that GLP-1R-mediated delivery of tesaglitazar would provide beneficial glucometabolic effects at doses subthreshold for each monotherapy, thereby improving systemic metabolism at more tolerable doses. In human embryonic kidney 293 (HEK293) and pancreatic β cells derived from an insulinoma of a transgenic mouse line (MIN6 cells)^22^, GLP-1RA/tesaglitazar showed comparable ligand-induced GLP-1R signalling and receptor internalization relative to the pharmacokinetically matched GLP-1RA. The conjugate showed GLP-1R-dependent PPARɣ-RXR heterodimerization and enhanced in vivo efficacy to reduce body weight and food intake, and to improve glucose metabolism relative to the GLP-1RA, tesaglitazar and to the fixed dose combination of the GLP-1RA and tesaglitazar in DIO mice. The ability of the conjugate to decrease body weight and to improve glucose control is absent in GLP-1R knockout mice and is preserved in DIO mice even at doses subthreshold for the GLP-1RA and tesaglitazar alone to improve glucose control. In line with reports indicating that PPARɣ may act on hypothalamic neurocircuitries^5,6^, we identified a series of new PPAR protein targets in the hypothalamus that are acutely upregulated by tesaglitazar and by GLP-1RA/tesaglitazar, but not by treatment with the GLP-1RA. Collectively, our data identify a series of tesaglitazar targets in the hypothalamus and show that the GLP-1RA/tesaglitazar conjugate improves body weight, food intake and glucose metabolism with superior efficacy relative to treatment with the GLP-1RA, tesaglitazar or the fixed dose combination of GLP-1RA and tesaglitazar. Our data indicate that GLP-1RA/tesaglitazar might hold therapeutic value for conditions characterized by hyperglycaemia and insulin resistance.

## Results

### GLP-1RA/tesaglitazar shows GLP-1R-dependent effects in HEK293 and Min6 cells

**Fig. 1:**
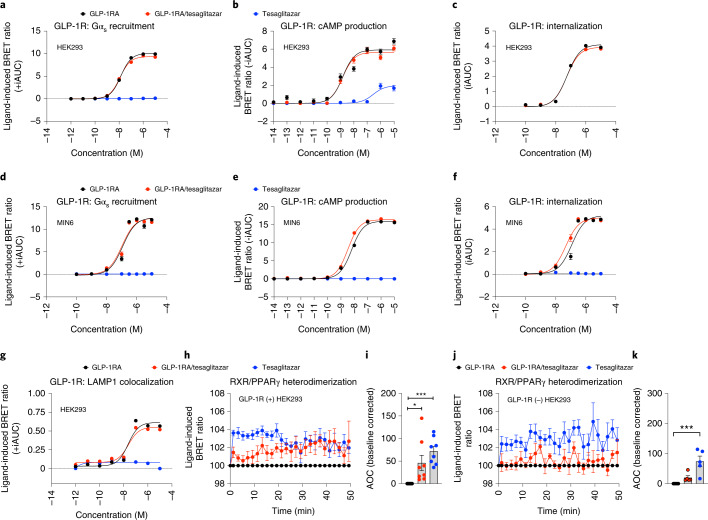
In vitro effects of GLP-1RA/tesaglitazar on GLP-1R signalling. **a**,**b**, Dose responses for ligand-induced recruitment of Nluc-tagged Mini-Gα_s_ to hGLP-1R-GFP (**a**) and cAMP production in hGLP-1R^+^ HEK293T cells (**b**). **c**, Dose responses for ligand-induced hGLP-1R-Rluc8 internalization as measured by loss of BRET with plasma membrane marker Venus-KRAS in HEK293T cells. **d**–**f**, Dose responses for ligand-induced hGLP-1R-GFP Mini-Gα_s_ recruitment (**d**), cAMP production (**e**) and hGLP-1R-Rluc8 internalization (**f**) in the mouse β-cell MIN6 cells. **g**, Dose responses for ligand-induced hGLP-1R-Rluc8 co-localization with the terminal lysosome marker Lamp1-mNeonGreen in HEK293T cells. **h**–**k**, Temporal resolution (**h**,**j**) and AOC (**i**,**k**) of ligand-induced (1 μM) RXR-Rluc8 and PPARγ2-YFP heterodimerization in hGLP-1R(+) HEK293T cells (**h**,**i**) and hGLP-1R(−) HEK293T cells (**j**,**k**). Data in **a**,**c**,**d**,**g** represent ± iAUC of *n* = 3 independent biological samples, each measured in an independent study with *n* = 4 technical replicates. Data in **b**,**e**,**f** represent ± iAUC of *n* = 4 independent biological samples, each measured in an independent study with *n* = 4 technical replicates. Data in **h**,**i**,**j**,**k** represent baseline-corrected temporal dynamics (**h**,**j**) and AOC (**i**,**k**) of *n* = 8 (**h**,**i**) or *n* = 5 (**j**,**k**) independent biological samples, each measured in an independent study with *n* = 4 technical replicates. Data were analysed using one-way ANOVA using the Bonferroni’s multiple comparison test. Date represent mean ± s.e.m.; asterisks indicate *P < 0.05; **P < 0.01 and ***P < 0.001. Exact *P* values for treatment effects are **i**, *P* = 0.0208 and *P* = 0.0005, **k**, *P* = 0.0008. Source data

**Extended Data Fig. 1:**
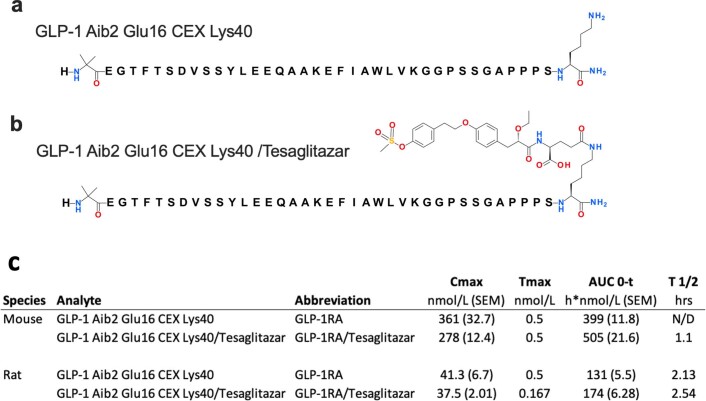
Structure and pharmacokinetics of the GLP-1RA and the GLP-1RA/Tesaglitazar conjugate. Chemical structures of the GLP-1RA (a) and the GLP-1RA/Tesaglitazar conjugate (b). Pharmacokinetic analysis of GLP-1RA and GLP-1RA/Tesaglitazar in 25–30-week-old male C57BL6/J mice (n = 4/4 mice) and 8–10-week-old male Sprague Dawley rats (n = 4/4 rats) (c). Abbreviations indicate half-life (T_1/2_), maximum plasma concentration (C_max_), time for maximal plasma concentration (T_max_) and area under the curve from zero to last valid measurable concentration-timepoint (AUC_0-t_). N/D not determinable. Data represent means ± SEM. Source data

The GLP-1 Aib2 Glu16 CEX Lys40/tesaglitazar conjugate (henceforth just named GLP-1RA/tesaglitazar) was assembled on a previously validated GLP-1 receptor agonist (GLP-1 Aib2 Glu16 CEX Lys40, henceforth named GLP-1RA)^23,24^. This GLP-1RA features the C-terminal extension of exendin-4, which was sequentially added to the human GLP-1 sequence that includes two substitutions (aminoisobutyric acid (Aib) at position 2 and glutamic acid (Glu) at position 16) (Extended Data Fig. 1a). Tesaglitazar was covalently attached to the side chain amine of the C-terminal lysine 40 residue via a gamma glutamic acid spacer (Extended Data Fig. 1b). In rats and mice, the GLP-1RA/tesaglitazar conjugate showed comparable pharmacokinetics relative to the GLP-1RA, including half-life (*T*_1/2_), maximum plasma concentration (*C*_max_), time for maximal plasma concentration (*T*_max_) and area under the curve (AUC) from zero to last valid measurable concentration-time point (AUC_0-t_) (Extended Data Fig. 1c). In HEK293 cells transfected with human GLP-1R, GLP-1RA/tesaglitazar showed comparable efficacy relative to the pharmacokinetically matched GLP-1RA to induce GLP-1R-mediated Gα_s_ recruitment, cAMP production and GLP-1R internalization (Fig. 1a–c), and this was confirmed also in mouse MIN6 β-cells (Fig. 1d–f). No difference was observed in lysosomal GLP-1R appearance, as indicated by comparable GLP-1R-Lamp1 colocalization following treatment with GLP-1RA or GLP-1RA/tesaglitazar in HEK293 cells (Fig. 1g). Tesaglitazar alone had no effect on GLP-1R Gαs recruitment, cAMP production, GLP-1R internalization or GLP-1R-Lamp1 colocalization (Fig. 1a–g). Consistent with the kinetic of ligand-induced GLP-1R internalization (Fig. 1c,f), GLP-1RA/tesaglitazar showed a delayed onset of PPARγ/RXR heterodimerization with comparable potency relative to tesaglitazar after 30 min when tested in GLP-1R expressing HEK293 cells (Fig. 1h,i). No meaningful PPARγ/RXR heterodimerization was observed by GLP-1RA/tesaglitazar treatment in HEK293 cells that lack GLP-1R (Fig. 1j,k). Thus, GLP-1RA/tesaglitazar induces PPARγ/RXR heterodimerization in a GLP-1R-dependent manner and shows equal, yet not superior, efficacy as the pharmacokinetically matched GLP-1RA control in activation, internalization and degradation of GLP-1R.

### GLP-1RA/tesaglitazar improves energy and glucose metabolism in DIO mice

**Fig. 2:**
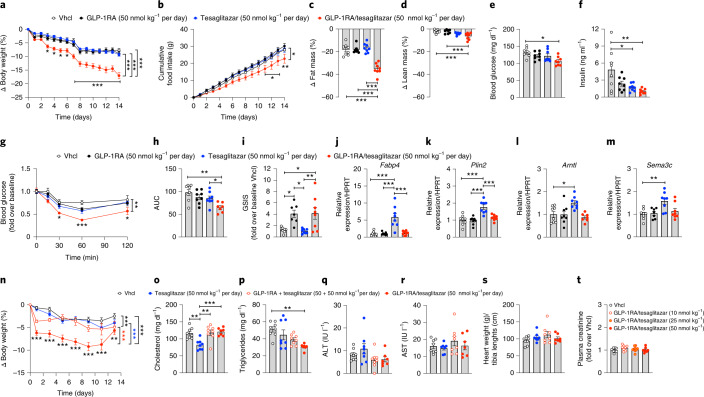
Chronic high-dose effects of GLP-1RA/tesaglitazar in obese and lean mice. **a**–**d**, Effects on body weight (**a**), cumulative food intake (**b**) and change of fat (**c**) and lean tissue mass (**d**) in 34-week-old male C57BL/6J DIO mice treated for 14 days with 50 nmol kg^−1^ per day. **e**,**f**, Fasting levels of blood glucose (**e**) and insulin (**f**) at study day 7. **g**,**h**, i.p. insulin tolerance (ipITT) assessed at day 14: blood glucose (**g**) and AUC (**h**). **i**, In vivo glucose-stimulated insulin secretion (GSIS) in 47-week-old male DIO mice treated i.p. with 1.5 g glucose per kg body weight (blood was collected at time points 0 and 15 min after glucose injection) (**i**). **j**–**m**, Expression of *Fabp4* (**j**) and *Plin2* (**k**) in liver and of *Arntl* (**l**) and *Sema3c* (**m**) in skeletal muscle of 30-week-old male DIO mice treated for 3 days with 50 nmol kg^−1^ per day. **n**–**s**, Changes in body weight (**n**), plasma levels of cholesterol (**o**), triglycerides (**p**), aspartate-aminotransferase (**q**), alanine-transferase (**r**) as well as heart weight (**s**) in 16-week-old male lean C57BL/6J mice after 14 days treatment with 50 nmol kg^−1^ per day. **t**, Plasma levels of creatinine in 44-week-old male C57BL/6J DIO mice treated for 14 days with vehicle (Vhcl) or GLP-1RA/tesaglitazar at a dose of 10, 25 or 50 nmol kg^−1^ per day (*n* = 8 mice each group). Sample sizes for treatment with Vhcl, GLP-1RA, tesaglitazar or GLP-1RA/tesaglitazar (**a**–**m**) are *n* = 8/8/8/8 mice (**a**,**d**,**f**,**j**,**k**), *n* = 8/7/8/8 mice (**c**,**i**,**m**), *n* = 7/8/8/8 mice (**e**) and *n* = 8/8/8/7 mice (**g**,**h**,**l**). Cumulative food intake (**b**) was assessed per cage in *n* = 4/4/4/4 cages containing *n* = 8/8/8/8 mice. Sample sizes for treatment with Vhcl, tesaglitazar, GLP-1RA + tesaglitazar or GLP-1RA/tesaglitazar (**n**–**s**) are *n* = 8/7/8/7 mice (**n**), *n* = 8/8/8/7 mice (**o**–**q**,**s**) and *n* = 8/8/7/7 mice (**r**). Data represent means ± s.e.m. Data in **a**, **b**, **g** and **n** have been analysed by two-way ANOVA with Bonferroni post hoc comparison for individual time points. Data in **c**–**f**, **h**–**m** and **o**–**t** have been analysed by one-way ANOVA using the Bonferroni’s multiple comparison test. Asterisks indicate *P < 0.05, **P < 0.01, ***P < 0.001. Exact *P* values for treatment effects are **a** all *P* ≤ 0.0001; **b** *P* = 0.043; **c** all *P* < 0.0001, **d** *P* = 0.0007 (versus Vhcl) and *P* = 0.0010 (versus GLP-1RA); **e** *P* = 0.0106; **f** *P* = 0.0216 and *P* = 0.0035; **g** *P* = 0.0064; **h** *P* = 0.0011 and *P* = 0.0359; **i** *P* = 0.024 (Vhcl versus GLP-1RA), *P* = 0.0143 (Vhcl versus GLP-1RA/tesaglitazar), *P* = 0.0125 (GLP-1RA versus GLP-1RA/tesaglitazar), *P* = 0.0072 (tesaglitazar versus GLP-1RA/tesaglitazar; **j** all *P* < 0.0001; **k** all *P* < 0.0001; **l** *P* = 0.0127; **m** *P* = 0.0032; **n** *P* = 0.0002 (GLP-1RA + tesaglitazar versus Vhcl), all other *P* < 0.0001; **o** *P* = 0.0012 (GLP-1RA + tesaglitazar versus Vhcl), *P* = 0.0008 (GLP-1RA/tesaglitazar versus tesaglitazar), *P* = 0.0020 (Vhcl versus tesaglitazar); **p** *P* = 0.0031. For exact *P* values at individual time points (**a**,**b**,**g**,**n**), see the data source file. Source data

**Extended Data Fig. 2:**
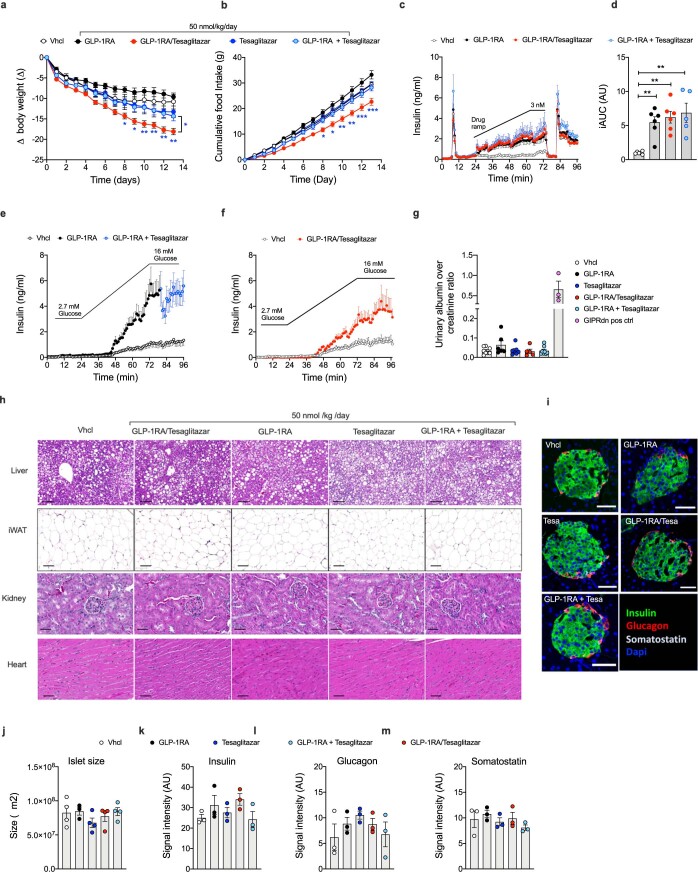
Effects of GLP-1RA/Tesaglitazar relative to GLP-1RA + Tesaglitazar co-therapy. Change in body weight **(a)** and food intake **(b)** of 47-wk old male DIO C57BL6/J mice treated daily s.c. with Vhcl (n = 8 mice) or 50 nmol/kg of either GLP-1RA (n = 7 mice), Tesaglitazar (n = 8 mice), GLP-1RA/Tesaglitazar (n = 8 mice) or GLP-1RA + Tesaglitazar (n = 8 mice). Cumulative food intake was assessed per cage in n = 4/4/4/4 cages containing n = 8/7/8/8 mice **(b)**. Glucose-stimulated insulin secretion in islets isolated from n = 10 male 18-wk old C57BL6/J mice (Run 1) and n = 10 female 12-wk old C57BL/6 J mice (Run 2), and which were treated with either control, GLP-1RA, GLP-1/Tesaglitazar or GLP-1RA + Tesaglitazar (n = 6/6/6/5 biologically independent replicates) under dynamic drug concentrations up to 3 nM under glucose concentrations of 10 mM **(c,d)**. Data in **(c,d)** represent combined results from the two independent runs. Islets were pooled and separated randomly between the treatment groups. After 4 min of low glucose (2.7 mM), levels of glucose were increased to 10 mM at t = 5 min. The peak at t = 10 min represents 1^st^ phase insulin secretion, the peak at t = 80 min represents treatment with 2.7 mM glucose + 30 mM KCL **(c,d)**. Glucose-stimulated insulin secretion in pooled islets isolated from 10–15-wk old male (n = 9) and female (n = 6) C57BL6/J mice treated with either control, GLP-1RA or GLP-1RA + Tesaglitazar (n = 9/6/6 biologically independent biological replicates) **(e)** or with control or GLP-1RA/Tesaglitazar (n = 9/6 independent biological replicates) **(f)** under dynamic glucose concentrations ranging from 2.7–16 mM at drug concentrations of 3 nM. Urinary albumin over creatinine ratio in 49-wk old male DIO C57BL6/J mice after 14 days of daily s.c. treatment with Vhcl or 50 nmol/kg of either GLP-1RA/Tesaglitazar, GLP-1RA, Tesaglitazar, GLP-1RA + Tesaglitazar (n = 8/5/6/7/8 mice) **(g)**. Urinary samples of a mouse model for diabetic nephropathy (GIPRdn)^32^ (n = 3 mice) serves as positive control **(g)**. Representative images of histopathological analysis of liver, iWAT, kidney and heart in 49-wk old male DIO C57BL6/J mice after 14 days of daily s.c. treatment with 50 nmol/kg (scale bar liver/iWAT: 100 µm, kidney/heart: 50 µm) **(h)**. Representative confocal images of pancreatic sections obtained in 49-wk old male DIO C57BL6/J mice after 14 days of daily s.c. treatment with 50 nmol/kg and stained for different pancreatic hormones (scale bar 50 μm) **(i)**. Pancreatic islet size **(j)** and hormone content between different groups **(k-m)** of 49-wk old male DIO C57BL6/J mice after 14 days of daily s.c. treatment with Vhcl or 50 nmol/kg of either GLP-1RA, Tesaglitazar, GLP-1RA + Tesglitazar or GLP-1RA/Tesaglitazar. Sample sizes are n = 4/3/4/4/4 mice **(j)** and n = 3/3/3/3 mice **(k-m)**. Data in panel **a** and **b** have been analyzed by 2-way ANOVA using the Bonferroni’s multiple comparison test for individual time points. Data in panel **d, g, and j-m** have been analyzed by 1-way ANOVA with Bonferroni multiple comparison test. Data represent means ± SEM; asterisks indicate * p < 0.05, ** p < 0.01, *** p < 0.001. Blue asterisks in panel **a** and **b** indicate significance of GLP-1RA/Tesaglitazar over the fixed dose combination of the GLP-1RA + Tesaglitazar. Exact p-values for treatment effects are (a) p = 0.0105, (d) p = 0.0089 (Cntrl. vs. GLP-1RA), p = 0.0021 (Cntrl. vs. GLP-1RA/Tesaglitazar), p = 0.0011 (Cntrl. vs. GLP-1RA + Tesaglitazar). For exact p-values at individual time points **(a,b)** see data source file. Source data

**Extended Data Fig. 3:**
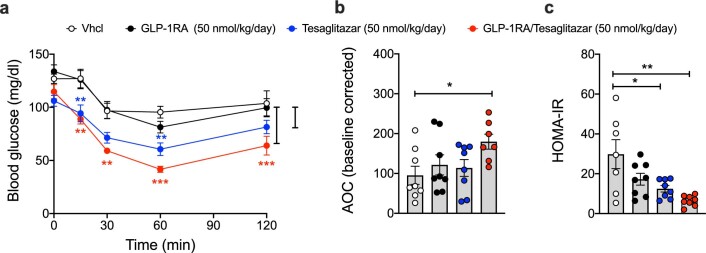
Insulin sensitivity in DIO mice after treatment with GLP-1/Tesaglitazar. Intraperitoneal insulin tolerance (ITT) **(a)**, baseline-corrected area of curve (AOC) **(b)** and HOMA-IR **(c)** of male 34-wk old DIO mice treated for 14-days with Vhcl or 50 nmol/kg of either GLP-1RA, Tesaglitazar or GLP-1RA/Tesaglitazar. Sample sizes are n = 8/8/8/7 mice **(a,b)** and n = 7/8/8/8 mice **(c)**. Data in **(a)** were analyzed by 2-way ANOVA with Bonferroni post-hoc comparison for individual time points. Data in **(b,c)** were analyzed by 1-way ANOVA with Bonferroni multiple comparison test. Data represent means ± SEM; asterisks indicate * p < 0.05, ** p < 0.01, *** p < 0.001. Red asterisks indicate significance of GLP-1RA/Tesaglitazar over Vhcl, blue asterisks indicate significance of Tesaglitazar over Vhcl. Exact p-values for treatment effects are (a) p = 0.0016 and p < 0.0001; (b) p = 0.0424; (c) p = 0.0193 and p = 0.0010. Source data

In DIO mice, 14-day treatment with GLP-1RA/tesaglitazar (50 nmol kg^−1^ per day) decreased body weight and food intake with superior potency relative to treatment with equimolar doses of the GLP-1RA or tesaglitazar (Fig. 2a,b). GLP-1RA/tesaglitazar (50 nmol kg^−1^ per day) also led to a greater decrease in body weight and food intake relative to DIO mice treated with a fixed dose combination of the GLP-1RA and tesaglitazar (Extended Data Fig. 2a,b). The decreased body weight in GLP-1RA/tesaglitazar-treated mice was associated with a decrease in body fat mass, with only a slight but significant decrease in lean tissue mass (Fig. 2c,d). GLP-1RA/tesaglitazar, but not the GLP-1RA or tesaglitazar alone, decreased levels of fasted blood glucose (Fig. 2e). This was paralleled by a more significant decrease in levels of fasted insulin (Fig. 2f), and improved insulin sensitivity relative to vehicle-treated controls, as indicated by direct assessment of insulin tolerance (Fig. 2g,h and Extended Data Fig. 3a,b) and HOMA-IR (Extended Data Fig. 3c). Consistent with this, 15 min after intraperitoneal (i.p.) administration of glucose, DIO mice treated with GLP-1RA/tesaglitazar showed equal insulin secretion relative to mice treated with the pharmacokinetically matched GLP-1RA control (Fig. 2i). Both drugs induced insulin secretion to similar levels in isolated islets under high glucose conditions with dynamic drug concentrations up to 3 nM (Extended Data Fig. 2c,d), and under dynamic glucose concentrations ranging from 2.7 to 16 mM glucose (Extended Data Fig. 2e,f). In line with the observation that GLP-1RA/tesaglitazar is not superior to the GLP-1RA in GLP-1R signalling, internalization or degradation (Fig. 1a–f), this suggests that factors other than enhanced GLP-1R action in the β cells contribute to the superior glucometabolic effect of the GLP-1RA/tesaglitazar conjugate. Consistent with the direct assessment of insulin sensitivity (Fig. 2g,h and Extended Data Fig. 3a,b), these data further indicate that the tesaglitazar moiety of the conjugate contributes to the glycaemic benefits of this molecule by improving insulin sensitivity.

In line with our data showing that GLP-1RA/tesaglitazar only induces PPAR/RXR heterodimerization in the presence of GLP-1R (Fig. 1h–k), tesaglitazar, but not GLP-1RA/tesaglitazar, induced expression of known PPAR target genes in tissues that lack GLP-1R expression, notably the liver (*Fabp4* and *Plin2*) and skeletal muscle (*Arntl* and *Sema3c*) (Fig. 2j–m). GLP-1RA/tesaglitazar also decreased body weight in lean mice with superior efficacy relative to equimolar treatment with either tesaglitazar or to a combination of GLP-1RA and tesaglitazar (Fig. 2n). Consistent with the observation that tesaglitazar, but not GLP-1RA/tesaglitazar, increased expression of PPAR target genes in the livers of mice that lack GLP-1R expression (Fig. 2j–m), only treatment with tesaglitazar, but not (equimolar doses of) GLP-1RA or GLP-1RA/tesaglitazar, reduced plasma cholesterol levels (Fig. 2o). These data hence indicate that conjugation of tesaglitazar to the GLP-1RA prevents hepatic PPAR activation by tesaglitazar. In line with the greater decrease in body weight and body fat mass in mice treated with GLP-1RA/tesaglitazar (Fig. 2a,c), plasma levels of triglycerides were decreased by treatment with GLP-1RA/tesaglitazar but not with GLP-1RA or tesaglitazar alone (Fig. 2p). No differences were observed in any of the treatment groups in markers indicative of liver damage (AST and ALT) (Fig. 2q,r) or heart weight (Fig. 2s). Daily treatment of DIO mice with GLP-1RA/tesaglitazar at doses of 10, 25 or 50 nmol kg^−1^ per day showed no difference in plasma creatinine levels, relative to vehicle controls after 14 days of treatment, suggesting normal kidney function (Fig. 2t). Urinary albumin:creatinine ratio was likewise not changed after 14-day treatment of DIO mice with 50 nmol kg^−1^ per day of GLP-1RA/tesaglitazar, tesaglitazar, the GLP-1RA or the combination of GLP-1RA + tesaglitazar (Extended Data Fig. 2g). Emphasizing the value of this finding, urinary albumin:creatinine was highly increased in positive control GIPRdn mice (Extended Data Fig. 2g), a mouse model exhibiting diabetic nephrophathy^25^. Histopathological analysis of liver, iWAT, kidney and heart revealed no abnormalities in any of the treatment groups (Extended Data Fig. 2h). No differences were observed in islet histology, islet size or fluorescent intensity of insulin, glucagon or somatostatin (Extended Data Fig. 2i–m).

### GLP-1RA/tesaglitazar acutely improves glucose metabolism in DIO mice

**Fig. 3:**
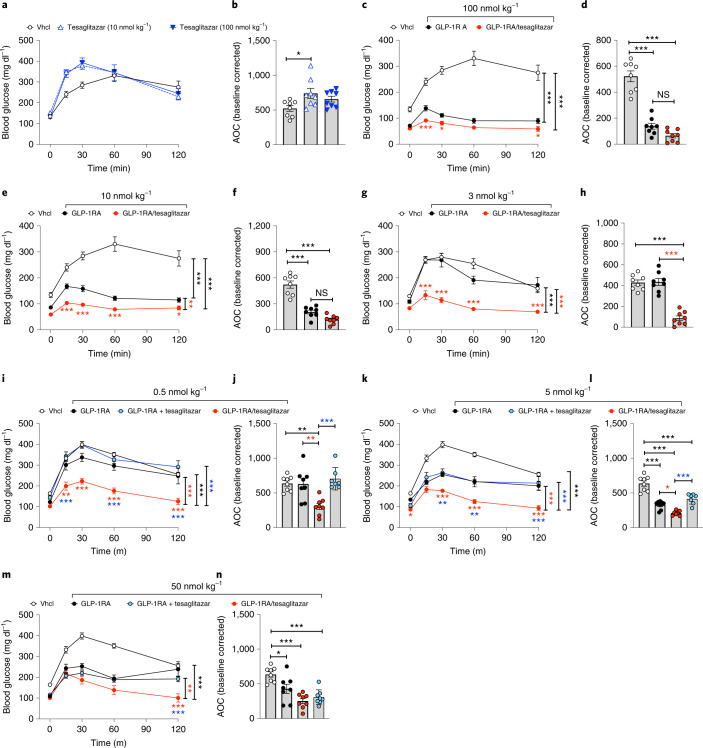
Acute glycaemic effects of GLP-1RA/tesaglitazar in DIO mice. **a**–**h**, i.p. GTT in 34-week-old male naïve DIO mice 6 h after treatment with a single s.c. dose of tesaglitazar (10 (**a**) or 100 nmol kg^−1^ (**b**)), or with the GLP-1RA or GLP-1RA/tesaglitazar at a dose of 100 nmol kg^−1^ (**c**,**d**), 10 nmol kg^−1^ (**e**,**f**) or 3 nmol kg^−1^ (**g**,**h**). **i**–**n**, i.p. GTT in 27–29-week-old male DIO mice 6 h after single s.c. treatment at doses of 0.5 nmol kg^−1^ (**i**,**j**), 5 nmol kg^−1^ (**k**,**l**) or 50 nmol kg^−1^ (**m**,**n**). In **a**–**h**, sample sizes are *n* = 8 mice each treatment group. In **i**–**n**, sample sizes for treatment with Vhcl, GLP-1RA, GLP-1RA + tesaglitazar and GLP-1RA/tesaglitazar are *n* = 8/8/8/7 mice (**i**,**j**,**m**,**n**), *n* = 8/8/8/8 mice (**k**) and *n* = 8/8/7/7 mice (**l**). Data represent means ± s.e.m. Data in **a**, **c**, **e**, **g**, **I**, **k** and **m** have been analysed by two-way ANOVA with Bonferroni post hoc comparison for individual time points. Data in **b**, **d**, **f**, **h**, **j**, **l** and **n** have been analysed by one-way ANOVA using Bonferroni’s multiple comparison test. Asterisks indicate *P < 0.05, **P < 0.01, ***P < 0.001. Black asterisks indicate comparison to Vhcl, red asterisks indicate comparison to GLP-1RA, blue asterisks indicate comparison to the GLP-1RA + tesaglitazar cotherapy. Exact *P* values for treatment effects are **b** *P* = 0.0199; **c** both *P* < 0.0001; **d** both *P* < 0.0001; **e** *P* = 0.0056, *P* < 0.0001 and *P* < 0.0001; **f** both *P* < 0.0001; **g** both *P* < 0.0001; **h** both *P* < 0.0001; **i** all *P* < 0.0001; **j** *P* = 0.0021 (black asterisks), *P* = 0.0027 (red asterisks), *P* = 0.0003 (blue asterisks); **k** *P* = 0.0011 (red asterisks), *P* < 0.0001 (blue asterisks) and *P* < 0.0001 (black asterisks); **l** *P* = 0.0388 (red asterisks), *P* = 0.0002 (blue asterisks), *P* < 0.0001 (Vhcl versus all other groups); **m** *P* = 0.0036 (red asterisks), *P* < 0.0001 (black asterisks); **n** *P* = 0.0229, *P* = 0.0002 (Vhcl versus GLP-1RA + tesaglitazar), *P* < 0.0001 (Vhcl versus GLP-1RA/tesaglitazar). For exact *P* values at individual time points (**b**,**e**,**g**,**i**,**k**,**m**), see the data source file. Source data

On the basis of the insulin-sensitizing effect of GLP-1RA/tesaglitazar in DIO mice (Fig. 2g,h and Extended Data Fig. 3a–c), we next assessed in naïve and weight-matched DIO mice whether the acute glycaemic benefits prevail even at single subcutaneous (s.c.) doses subthreshold for the GLP-1RA to improve glucose metabolism. Single bolus peripheral treatment of weight-matched DIO mice with tesaglitazar at doses of 10 and 100 nmol kg^−1^ acutely worsened i.p. glucose tolerance (Fig. 3a,b). When given at a dose of 100 nmol kg^−1^, the GLP-1RA and GLP-1RA/tesaglitazar both improved glucose tolerance with only slightly enhanced efficacy of the conjugate relative to the GLP-1RA (Fig. 3c,d), which is not surprising as these doses far exceed the maximally efficacious doses of this GLP-1RA. At a dose of 10 nmol kg^−1^ GLP-1RA/tesaglitazar showed slightly superior efficacy at improving glucose control (Fig. 3e) but with no statistical superiority over the GLP-1RA in the area of the curve (AOC) (Fig. 3f). At a dose of 3 nmol kg^−1^, the GLP-1RA failed to improve glucose tolerance, while the beneficial glycaemic effect of GLP-1RA/tesaglitazar fully prevailed (Fig. 3g,h). These data indicate that GLP-1RA/tesaglitazar possesses, particularly at low concentrations, a substantially enhanced potency to acutely improve glucose tolerance relative to the GLP-1RA monotherapy. Emphasizing the value of this finding, glucose tolerance was even substantially improved by GLP-1RA/tesaglitazar, but not by any other treatment group, at a dose of 0.5 nmol kg^−1^ (Fig. 3i,j). When compared at doses of 0.5, 5 and 50 nmol kg^−1^ in the same study, GLP-1RA/tesaglitazar also improved glucose tolerance with superior efficacy relative to the fixed dose combination of the GLP-1RA + tesaglitazar, but with decreasing superiority with increased concentrations (Fig. 3i–n). At none of the tested doses was glucose tolerance different between mice treated with the fixed dose combination of the GLP-1RA + tesaglitazar and the GLP-1RA monotherapy (Fig. 3i–n).

### Low-dose GLP-1/tesaglitazar improves glucose handling in DIO mice

**Fig. 4:**
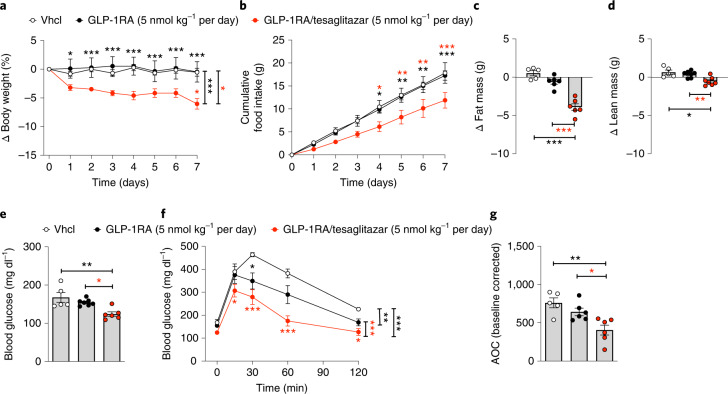
Chronic low-dose effects of GLP-1RA/tesaglitazar in DIO mice. **a**–**d**, Body weight (**a**), cumulative food intake (**b**) and change in fat (**c**) and lean tissue mass (**d**) of 36-week-old male C57BL/6J DIO mice treated for 7 days with either vehicle or 5 nmol kg^−1^ per day of either GLP-1 or GLP-1/tesaglitazar. **e**–**g**, Fasting levels of blood glucose (**e**) and i.p. GTT (**f**,**g**). Sample sizes for treatment with Vhcl, GLP-1RA or GLP-1RA/tesaglitazar are *n* = 5/7/6 (**a**–**g**). Cumulative food intake was assessed per cage in *n* = 3/4/3 cages containing *n* = 5/7/6 mice (**d**). Data in **a**,**b** and **f** have been analysed by two-way ANOVA with Bonferroni post hoc comparison for individual time points. Data in **c**, **d**, **e** and **g** have been analysed by one-way ANOVA using the Bonferroni’s multiple comparison test. Data represent means ± s.e.m.; asterisks indicate *P < 0.05, **P < 0.01, ***P < 0.001. Red asterisks indicate comparison to the GLP-1RA, black asterisks indicate comparison to Vhcl. Exact *P* values for treatment effects are: **a** *P* = 0.044 and *P* = 0.0007, **c** all *P* < 0.0001; **d** *P* = 0.0099 and *P* = 0.0284; **e** *P* = 0.0035 and *P* = 0.0242; **f** *P* = 0.0030, *P* = 0.0004 (red asterisks) and *P* < 00001 (black asterisks), **g** *P* = 0.0027 and *P* = 0.030. For exact *P* values at individual time points (**a**,**b**,**f**), see the data source file. Source data

We next assessed whether daily chronic treatment with GLP-1RA/tesaglitazar also at low doses of 5 nmol kg^−1^ affects energy and glucose tolerance in DIO mice. When given at a daily dose of 5 nmol kg^−1^, 7 days of treatment with GLP-1RA/tesaglitazar decreased body weight and food intake in DIO mice, while mice treated with the GLP-1RA showed no difference in either body weight or food intake relative to vehicle controls (Fig. 4a,b). Consistent with the lower body weight, GLP-1RA/tesaglitazar decreased body fat mass relative to the GLP-1RA and to vehicle-treated mice (Fig. 4c) with only a slight but significant decrease in lean tissue mass (Fig. 4d). GLP-1RA/tesaglitazar but not the GLP-1RA decreased levels of fasted blood glucose (Fig. 4e), and GLP-1RA/tesaglitazar further improved glucose tolerance relative to mice treated with vehicle or GLP-1RA (Fig. 4f,g).

### GLP-1RA/tesaglitazar depends on functional GLP-1R and shows preserved effects in *db/db* mice

**Fig. 5:**
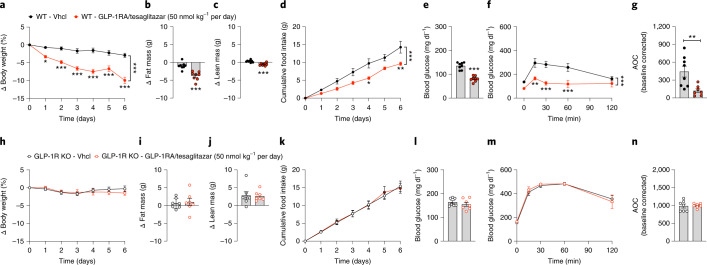
GLP-1RA/tesaglitazar in obese GLP-1R knockout mice. **a**–**n**, Body weight (**a**,**h**), change in fat and lean tissue mass (**b**,**c**,**i**,**j**), cumulative food intake (**d**,**k**), blood glucose (**e**,**l**) and i.p. GTT (**f**,**m**) in 36-week-old male DIO wildtype (WT) (**a**–**g**) or GLP-1R knockout (KO) mice (**h**–**n**) treated for 6 days with either vehicle or 50 nmol kg^−1^ per day of GLP-1RA/tesaglitazar. Sample sizes for treatment with Vhcl or GLP-1RA/tesaglitazar are *n* = 8/8 (**a**–**c**,**e**,**f**), *n* = 8/7 (**g**) and *n* = 7/7 (**h**–**j**,**l**–**n**). Cumulative food intake was assessed per cage in *n* = 4/3 cages containing *n* = 8/8 mice (**d**) and *n* = 4/5 cages containing *n* = 7/7 mice (**k**). Data in **a**, **d**, **f**, **h**, **k** and **m** have been analysed by two-way ANOVA with Bonferroni post hoc comparison for individual time points. Data in **b**, **c**, **e**, **g**, **i**, **j**, **l** and **n** have been analysed by one-way ANOVA using Bonferroni’s multiple comparison test. Data represent means ± s.e.m.; asterisks indicate *P < 0.05, **P < 0.01, ***P < 0.001. Exact *P* values for treatment effects are: **a**,**b**,**d**–**f**, *P* < 0.0001; **c**, *P* = 0.0003; **g**, *P* = 0.0078. For exact *P* values at individual time points (**a**,**d**,**f**,**h**,**k**,**m**), see the data source file. Source data

**Fig. 6:**
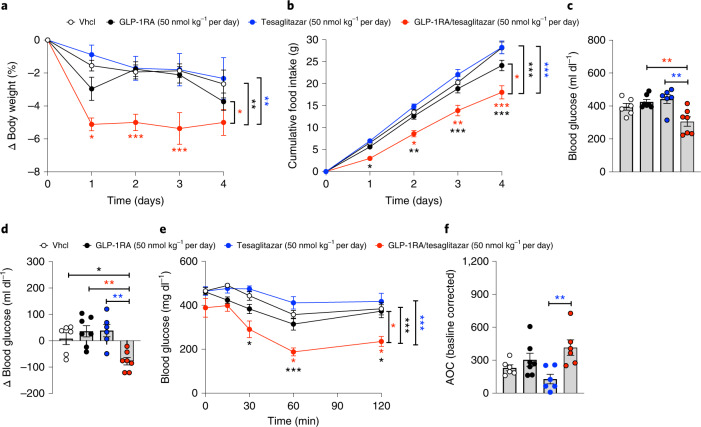
GLP-1RA/tesaglitazar effects in obese *db/db* mice. **a**–**f**, Body weight (**a**), cumulative food intake (**b**), plasma glucose (**c**), change in blood glucose (**d**) and glucose tolerance (**e**,**f**) in 6-week-old obese *db/db* mice treated for 4 days at a dose of 50 nmol kg^−1^ per day. Sample sizes for treatment with Vhcl, GLP-1RA, tesaglitazar or GLP-1RA/tesaglitazar are *n* = 6/7/6/7 (**a**,**c**,**d**) and *n* = 6/7/6/6 (**e**,**f**). Cumulative food intake was assessed per cage in *n* = 4/4/4/4 cages containing *n* = 6/7/6/7 mice (**b**). Data in **a**, **b** and **e** have been analysed by two-way ANOVA with Bonferroni post hoc comparison for individual time points. Data in **c**, **d** and **f** have been analysed by one-way ANOVA using the Bonferroni’s multiple comparison test. Data represent means ± s.e.m.; asterisks indicate *P < 0.05, **P < 0.01, ***P < 0.001. Black asterisks indicate comparison to Vhcl, red asterisks indicate comparison to GLP-1RA, blue asterisks indicate comparison to tesaglitazar. Exact *P* values for treatment effects are: **a**, *P* = 0.0353 (red asterisk), *P* = 0.0078 (black asterisks) and *P* = 0.0031 (blue asterisks); **b**, *P* = 0.0112 (red asterisk), *P* = 0.0007 (black asterisks) and *P* = 0.0002 (blue asterisks); **c**, *P* = 0.0062 (red asterisks) and *P* = 0.0025 (blue asterisks); **d**, *P* = 0.0409 (black asterisk), *P* = 0.0027 (red asterisks) and *P* = 0.0032 (blue asterisks); **e**, *P* = 0.0165 (red asterisk), *P* = 0.0009 (black asterisks) and *P* = 0.0002 (blue asterisks); **f**, *P* = 0.0075. For exact *P* values at individual time points (**a**,**b**,**e**), see the data source file. Source data

When given at a daily dose of 50 nmol kg^−1^, 6 days of treatment with GLP-1RA/tesaglitazar robustly improved body weight, fat mass, food intake, blood glucose and glucose tolerance in DIO wildtype mice (Fig. 5a–g), but not in DIO mice that lack the GLP-1 receptor (Fig. 5h–n). Consistent with the in vitro demonstration for a requirement of the GLP-1R to detect PPARɣ activity (Fig. 1h–k), these data indicate that the metabolic effects of GLP-1RA/tesaglitazar in vivo also require functional GLP-1R signalling. We next assessed whether the metabolic and glycaemic benefits of GLP-1RA/tesaglitazar translate from DIO mice to genetically obese and diabetic *db/db* mice. Consistent with the superior ability of GLP-1RA/tesaglitazar to decrease body weight and to improve glucose metabolism in DIO mice (Fig. 2a,e–h), GLP-1/tesaglitazar (50 nmol kg^−1^ per day), but not equimolar treatment with the GLP-1RA or tesaglitazar alone, decreased body weight in obese and diabetic *db/db* mice (Fig. 6a). Consistent with the greater decrease in body weight, GLP-1RA/tesaglitazar significantly reduced food intake relative to treatment with the GLP-1RA or tesaglitazar alone (Fig. 6b) and this was paralleled by a greater decrease in fasted blood glucose (Fig. 6c,d), and improved insulin sensitivity relative to mice treated with tesaglitazar (Fig. 6e,f). Collectively, these data show that GLP-1RA/tesaglitazar outperforms the GLP-1RA and tesaglitazar to decrease body weight and to improve glucose metabolism in mice with genetically induced obesity and glucose intolerance. The observation that the metabolic effects of GLP-1RA/tesaglitazar depend on functional GLP-1R in vivo align with our in vitro demonstration that GLP-1RA/tesaglitazar fails to induce PPARγ/RXR heterodimerization in the absence of GLP-1R (Fig. 1h–k).

### GLP-1RA/tesaglitazar engages PPAR targets in the hypothalamus

**Fig. 7:**
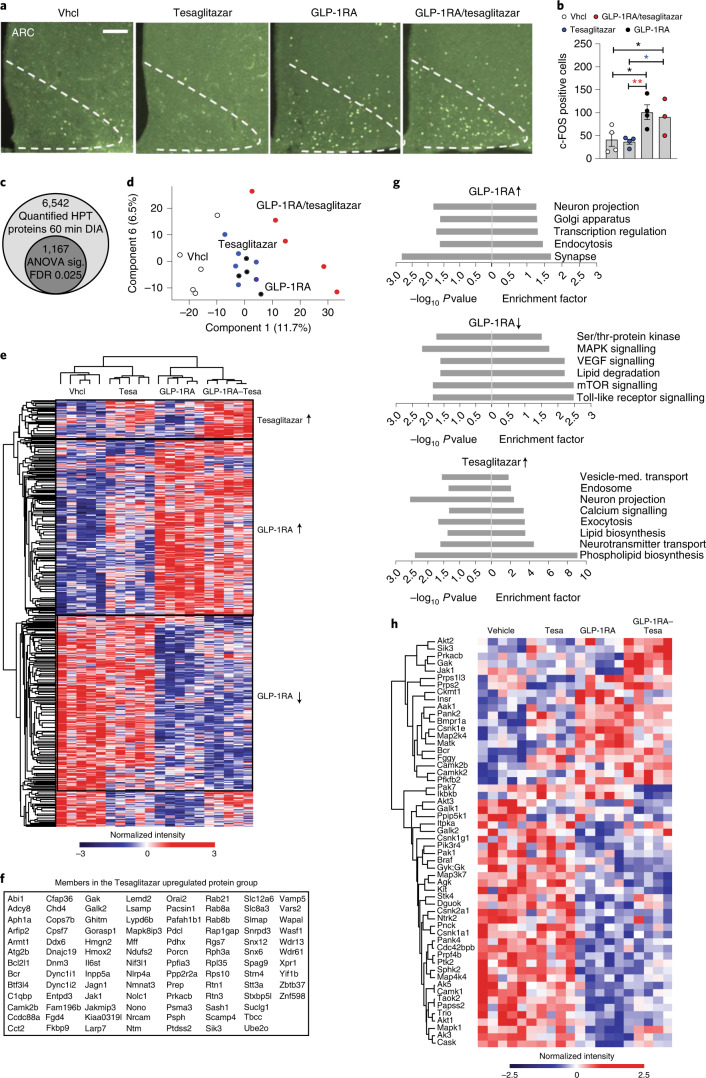
GLP-1RA/tesaglitazar effects on the hypothalamic proteome and cFOS. **a**,**b**, Representative immunofluorescence (**a**) and quantification (**b**) of cFos positive neurons in the ARC of 47-week-old male DIO mice after single s.c. treatment with Vhcl or 150 nmol kg^−1^ of GLP-1RA, tesaglitazar or GLP-1RA/tesaglitazar (*n* = 4/4/4/3 mice; scale bar, 100 µm). **c**–**h**, LC–MS analysis of acute drug effects on the hypothalamic proteome. **c**, Identified total number of quantified protein groups across all samples, as well as significantly changed number of proteins as determined by ANOVA (FDR < 0.025). **d**,**e**, PCA of proteomic samples (**d**) and heat map of *z*-scored protein intensities among all significantly changed proteins (**e**). **f**, List of proteins upregulated by tesaglitazar and by GLP-1/tesaglitazar. **g**,**h**, Selected gene annotations positively enriched in the specific treatment groups (**g**) and heat map of kinases significantly changed (ANOVA, FDR < 0.025) by the respective treatment groups (**h**). Proteins are grouped by hierarchical clustering, colouring represents *z*-scored protein intensities. Data in **a** and **b** were obtained after 90 min of drug exposure in *n* = 4/4/4/3 mice, data in **c**–**h** were obtained after 10 h of drug exposure in 49-week-old C57BL6J DIO mice (*n* = 5 mice each group). Data in **b** were analysed by one-way ANOVA and Fisher’s least-significant difference test. Data represent means ± s.e.m.; asterisks indicate *P < 0.05, **P < 0.01. Exact *P* values for treatment effects are: **b** *P* = 0.0138 (Vhcl versus tesaglitazar), *P* = 0.0466 (Vhcl versus GLP-1RA/tesaglitazar), *P* = 0.0326 (GLP-1RA/tesaglitazar versus tesaglitazar) and *P* = 0.0093 (GLP-1RA versus tesaglitazar). Source data

**Extended Data Fig. 4:**
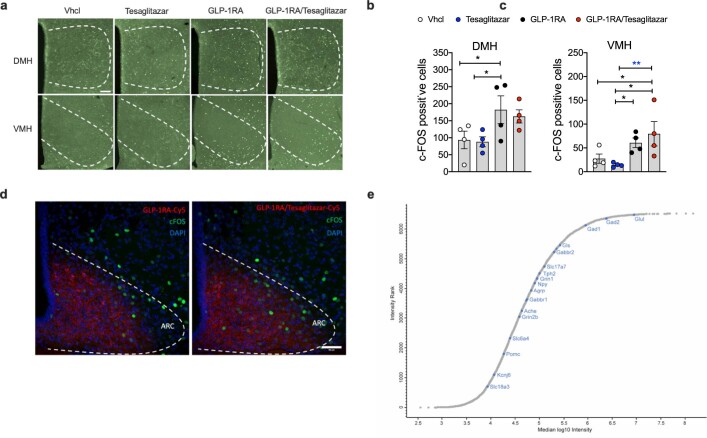
Acute drug effects on hypothalamic cFOS and neuronal marker. Immunofluorescence **(a)** and quantification **(b,c)** of cFos positive neurons in the ventromedial hypothalamus (VMH) and dorsomedial hypothalamus (DMH) (n = 4 male 47-wk old C57BL/6 J mice each group, scale bar: 100 µm). Representative immunofluorescence image of Cyanine 5 (Cy5) labeled appearance of GLP-1RA and GLP-1RA/Tesaglitazar in the ARC of 49-wk old male C57BL/6 J DIO mice after 90 min of treatment with a single s.c. dose of 150 nmol/kg of either GLP-1RA-Cy5 or GLP-1RA/Tesaglitazar-Cy5 (n = 3/4 mice; scale bar: 50 µm) **(d)**. Neuronal markers in the hypothalamic proteome **(e)**. To illustrate the large dynamic range, quantified proteins across all hypothalamic samples were ranked and plotted according to their median Intensity. Marker proteins of different neuronal subtypes are highlighted in blue. Data in **(b,c)** were analyzed by 1-way ANOVA and Fisher’s LSD test. Data represent means ± SEM; asterisks indicate * p < 0.05, ** p < 0.01, *** p < 0.001. Exact p-values for treatment effects are (b) p = 0.0388 (Vhcl vs. GLP-1RA) and p = 0.0299 (Tesaglitazar vs. GLP-1RA); (c) p = 0.0269 (Vhcl vs. GLP-1RA/Tesaglitazar), p = 0.0446 (GLP-1RA vs. Tesaglitazar) and p = 0.0082 (GLP-1RA vs. GLP-1RA/Tesaglitazar). Source data

A single s.c. administration of GLP-1RA/tesaglitazar induced a comparable cFOS neuronal activation pattern relative to the GLP-1RA in the arcuate nucleus (ARC) (Fig. 7a,b) and in the dorsomedial and ventromedial hypothalamic nuclei (Extended Data Fig. 4a–c). Consistent with this, using cyanine 5 (Cy5) peptide labelling, we observed equal efficacy of the GLP-1RA and the GLP-1RA/tesaglitazar conjugate to reach the ARC (Extended Data Fig. 4d). These data are in line with the in vitro demonstration that GLP-1RA/tesaglitazar acts via GLP-1R to promote RXR/PPARɣ heterodimerization (Fig. 1a–k), and the fact that the conjugate fails to affect body weight and glycaemia in the GLP-1R knockout mice (Fig. 5h–n). On the basis of the ability of GLP-1RA/tesaglitazar to induce neuronal activation in the hypothalamus, and recent data indicating that PPARɣ might affect systemic energy metabolism via hypothalamic neurocircuitries^5,6^, we next assessed the response of the hypothalamic proteome of DIO mice 10 h after single peripheral (s.c.) drug treatment using liquid chromatography–mass spectrometry (LC–MS). In single-shot 60-min data-independent acquisition analyses, we quantified more than 6,500 hypothalamic proteins (Fig. 7c). High reproducibility was indicated by a median coefficient of variation <2% for all conditions. The abundance ranked plot of the entire measured proteome spans a dynamic range of six orders of magnitude of estimated absolute abundance covering neuronal marker proteins among various abundance levels (Extended Data Fig. 4e). Principal component analysis (PCA) revealed a clear separation of the treatment groups in the first component. GLP-1RA/tesaglitazar induced a stronger PCA shift than the GLP-1RA or tesaglitazar alone (Fig. 7d). Choosing a stringent cut-off (analyis of variance (ANOVA), false discovery rate (FDR) < 0.025), 1,167 proteins exhibited statistically significant changes in their levels compared to vehicle-treated controls, thereby reflecting a strong proteomic response. Relative to vehicle-treated controls, we observed a solid pattern of the GLP-1RA responsive proteins in the hypothalamus, with 487 proteins being upregulated (Cluster 2) and 576 being downregulated by the GLP-1RA (Cluster 3) (Fig. 7e). Similar GLP-1 responsive protein patterns were observed after treatment with GLP-1RA/tesaglitazar (Fig. 7e,f). Enriched pathways (Fisher exact test, FDR < 0.025) upregulated by the GLP-1RA and GLP-1RA/tesaglitazar included signalling mechanisms implicated in neuronal projection and regulation of transcription and endocytosis, while pathways of TLR, mTOR, VEGF and MAPK signalling were downregulated (Fig. 7g). Notably, we also found a unique and similar pattern of hypothalamic proteins that were acutely upregulated by tesaglitazar and by GLP-1RA/tesaglitazar, but not by treatment with the GLP-1RA (104 proteins were upregulated by treatment with tesaglitazar and by GLP-1RA/tesaglitazar, Cluster 1) (Fig. 7e). Enriched pathways upregulated by tesaglitazar and GLP-1RA/tesaglitazar include mechanisms implicated in vesicle-mediated transport, neuron projection, Ca^2+^ signalling, (phospho)lipid biosynthesis and neurotransmitter transport (Fig. 7g). The GLP-1RA and GLP-1RA/tesaglitazar treatments affected the levels of several key kinases mediating metabolic signalling pathways (for example, insulin signalling) (Fig. 7h). Overall, the GLP-1RA/tesaglitazar proteomic signature reflected a combination of the specific GLP-1RA and tesaglitazar responses, integrating the effects on metabolic pathways and neuronal functions of the individual components. Notably, we observed slightly decreased GLP-1-selective changes in the hypothalamic proteome by treatment with GLP-1RA/tesaglitazar relative to the GLP-1RA (Fig. 7e). However, we observed stronger tesaglitazar-selective changes in the proteome by treatment with GLP-1RA/tesaglitazar relative to tesaglitazar alone (Fig. 7e). These data confirm that GLP-1RA/tesaglitazar engages PPAR targets in the hypothalamus and suggest that the known high expression of GLP-1R in the hypothalamus allows for a favourable delivery of GLP-1/tesaglitazar into this tissue. The enriched amplitude of PPAR-induced signalling observed with the GLP-1RA/tesaglitazar conjugate is potentially due to selective biodistribution of the tesaglitazar to GLP-1R positive tissues, as opposed to systemic biodistribution achieved with unconjugated tesaglitazar treatment that would otherwise dilute the signalling amplitude.

## Discussion

We here report the development and molecular characterization of a GLP-1RA peptide biochemically modified for GLP-1R-dependent delivery of the PPARɑ/ɣ dual-agonist tesaglitazar. We demonstrate that GLP-1RA/tesaglitazar is comparably efficacious as the pharmacokinetically matched GLP-1RA to promote GLP-1R-mediated Gα_s_ recruitment, cAMP production, as well as internalization and degradation of the GLP-1 receptor in vitro, but is superior in decreasing body weight and improving glucose metabolism in vivo. Consistent with the in vitro data showing that GLP-1RA/tesaglitazar is not simply a GLP-1RA analogue of enhanced potency, we see comparable, yet not superior, stimulation of insulin secretion by GLP-1RA/tesaglitazar in vivo and also in isolated murine islets relative to the GLP-1RA. Rather, GLP-1RA/tesaglitazar demonstrates unique pharmacodynamic properties by inducing PPARɣ-RXR heterodimerization only in the presence of GLP-1R. This indicates that GLP-1RA/GLP1R interaction acts as a viable vector for tesaglitazar intracellular delivery. This results in comparable efficacy relative to tesaglitazar, yet with a notable delayed onset. This delayed onset of PPARɣ-RXR heterodimerization by GLP-1RA/tesaglitazar relative to tesaglitazar is consistent with the observed time-dependent kinetics of ligand-induced GLP-1R internalization, and suggests that the rate of GLP-1R-mediated internalization of GLP-1RA/tesaglitazar is critical to allow for an adequate induction of PPARɣ-RXR heterodimerization and hence to promote PPAR target effects. Consistent with this, we show lack of GLP-1RA/tesaglitazar effects in the GLP-1R negative tissues in vivo, notably liver and skeletal muscle, as well as in GLP-1R knockout mice.

Consistent with a differential pharmacodynamic signature, treatment with GLP-1RA/tesaglitazar led to greater reduction in body weight and food intake in DIO and *db/db* mice compared to treatment with the GLP-1RA or tesaglitazar, whereas the glucoregulatory and weight loss actions were eliminated in GLP-1R deficient mice. GLP-1RA/tesaglitazar was also superior to the fixed dose combination of the GLP-1RA and tesaglitazar to improve body weight, food intake and glucose metabolism. GLP-1RA/tesaglitazar was exceptionally potent to acutely improve glucose tolerance after single s.c. treatment in naïve and weight-matched DIO mice, particularly at very low doses subthreshold for either the GLP-1RA or tesaglitazar alone. These data hence indicate that the acute improvement of glucose control by GLP-1RA/tesaglitazar is not related to changes in body weight or food intake. Our data in isolated islets and in DIO mice show that this enhanced effectiveness is not mediated by enhanced insulin secretion but rather improved insulin sensitivity. Although this will require further testing, it is possible that the enhanced glycaemic effects of the conjugate may be mediated by glucoregulatory organs that receive feedback from the central nervous system, and specifically the hypothalamus. The aforementioned possibility would be in accordance with the differential hypothalamic proteomic signatures observed with GLP-1RA/tesaglitazar relative to the GLP-1RA alone. By using cFOS histological analysis and LC–MS proteomics, we identified the hypothalamus as a target of GLP-1RA/tesaglitazar, consistent with the known distribution pattern of GLP-1RA in mice. This is consistent with the observation that the hypothalamus and the hindbrain are primary targets of fluorescently labelled GLP-1R agonists^26,27^ and aligns with the demonstration of high GLP-1R abundance in these brain regions^28,29^. PPARɣ is expressed in various nuclei of the hypothalamus but not in the nucleus tractus solitarius of the brainstem^5^ and targeted overexpression of PPARɣ in the hypothalamus decreases food intake in lean and DIO mice^6^. While the mechanistic underpinnings underlying food intake regulation by PPARɣ remain largely unknown, in a hypothalamic cell line, stimulation of POMC expression by bisphenol A (BPA) is blocked by pretreatment with the PPARɣ antagonist T0070907 (ref. ^30^). In summary, there is accumulating evidence indicating that PPARɣ signalling engages hypothalamic neurocircuitries to control food intake, yet more work is required to delineate precise mechanisms.

The proposed model of GLP-1-mediated delivery of a nuclear acting PPARɑ/ɣ dual-agonist aligns with previous studies that have demonstrated the potential of this concept of peptide-mediated nuclear hormone delivery. In line with these notions, delivery of oestrogen via the GLP-1RA used here improves body weight, glucose metabolism and dyslipidaemia in DIO mice with greater efficacy relative to treatment with the GLP-1RA or oestrogen alone and without detrimental oestrogen effects in GLP-1R negative uterus or breast tissue^23^. The same GLP-1RA–oestrogen conjugate outperformed the GLP-1RA and oestrogen to restore β-cell mass and function in mice made diabetic by treatment with streptozotocin^31^. A molecule delivering dexamethasone via the GLP-1RA studied here improved hypothalamic inflammation and astrogliosis with superior efficacy relative to the GLP-1RA or dexamethasone alone^24^, while preferable delivery of thyroid hormone T3 via glucagon as the peptide carrier improved lipid and cholesterol metabolism with reduced off-target T3 effects in glucagon receptor negative tissues such as the heart, skeletal muscle and the bone^32^. Also, the selective activation of PPARɣ in NPY1R-expressing adipocytes using a molecule that covalently links tesaglitazar to neuropeptide Y was shown to enhance adipocyte differentiation and to prevent diabetes progression in *db/db* mice^33^. These provocative pharmacological findings reported with these peptide-mediated nuclear targeting set a foundation for further chemical refinement of the prototype chemical entities to improve their pharmaceutical properties. Further, this concept has provided a catalysis for receptor-mediated targeting of other effector molecules, including oligonucleotides for extra-hepatic modulation of specific gene targets^34,35^.

## Methods

### Compound synthesis

Peptidyl resin with modified GLP-1 backbone sequence of Boc-His(Trt)-aib-Glu(OtBu)-Gly-Thr(tBu)-Phe-Thr(tBu)-Ser(tBu)-Asp(OtBu)-Val-Ser(tBu)-Ser(tBu)-Tyr(tBu)-Leu-Glu(OtBu)-Glu(OtBu)-Gln(Trt)-Ala-Ala-Lys(Boc)-Glu(OtBu)-Phe-Ile-Ala-Trp-Leu-Val-Lys(Boc)-Gly-Gly-Pro-Ser(tBu)-Ser(tBu)-Gly-Ala-Pro-Pro-Pro-Ser(tBu)-Lys(Mtt)-amide resin was assembled by automated Fmoc/tBu solid-phase chemistry starting with 0.1 mM of H-Rink Amide ChemMatrix (PCAS BioMatrix Inc.) on a Symphony Peptide Synthesizer (Peptide Technology). All Fmoc-amino acids were coupled with 6-Cl-HOBt/DIC activation in dimethylformamide (DMF). The Fmoc were removed by 20% piperidine in DMF. After the Mtt removal by treating the peptidyl resin with 1–2% trifluoroacetic acid (TFA)/5% Tis in dichloromethane, Fmoc-Glu-OtBu was coupled with 6-Cl-HOBt/DIC in DMF. Fmoc was deprotected and tesaglitazar/(*S*)-2-ethoxy-3-(4-(4-((methylsulfonyl)oxy) phenethoxy) phenyl) propanoic acid (Astatech Inc.) was coupled with 6-Cl-HOBt/DIC in DMF with threefold excess. GLP-1 Aib2 Glu16 CEX Lys40–tesaglitazar conjugate peptide was cleaved from the resin with 10 ml TFA cleavage cocktail containing 8.5 ml of TFA, 0.5 ml of water, 0.5 ml Tis (triisopropylsilane), 0.25 g phenol and 0.25 ml 2-mercaptoethanol for 2 h. Peptide was precipitated with cold ether, dissolved in 20% acetonitrile (ACN) containing 2% acetic acid and injected to a Luna 19 × 250 nm/10 μm C8 column (Phenomenex) to purify with 0.1%TFA/ACN eluent solvents in a Waters 2545 preparative high-performance liquid chromatography instrument. Peptide molecular weight characterization was measured by liquid chromatography–mass spectrometry on an Agilent 1260 Infinity/6120 Quadrupole instrument with a Kinetex C8 column with an eluent gradient of 10–80% 0.05% ACN. The purified GLP-1 Aib2 Glu16 CEX Lys40–tesaglitazar conjugate (high-performance liquid chromatography >95%) was characterized with a molecular mass of 1,178.5/[M+4H]4+ and 1,570.8/[M+3H]3+, which is consistent with the theoretically calculated molecular weight of 4,710.1 with formula C_214_H_315_N_49_O_69_S. For assessment of brain penetrance, the GLP-1RA and the GLP-1RA/tesaglitazar conjugate were covalently attached to a cyanine 5 (Cy5) fluorophore (Lumiprobe no. 13380). The Cy5 was attached to the peptide by engineering a single cysteine into the peptide followed by conjugation of the fluorophore using a maleimide-containing building block.

### Animals

C57BL/6J mice and Lepr^db^ (*db/db*) mice were purchased from the Jackson Laboratory (no. 000697). *Glp1r*^−/−^ mice were kindly provided by D. Drucker (University of Toronto, Canada). All wildtype and knockout mice used in our studies were in-house bred on a C57BL/6J background. Mice were double-housed and kept in a constant environment with the ambient temperature set to 22 ± 2 °C with constant humidity (45–65%) and a 12 h/12 h light/dark cycle with lights off from 06:00 until 18:00. For studies in DIO mice, male C57BL/6J mice were fed with a high-fat diet comprising 58% kilocalories from fat (D12331, Research Diets). *db/db* mice were fed with a normal chow diet (T1314, Altromin GmbH) throughout the study. At the beginning of each experiment, mice were equally distributed into experimental groups according to their body weight and body composition. All animal studies were approved by the State of Bavaria, Germany, or the Insitutional Animal Care and Use Committee of the University of Cincinnati, OH, USA and conducted on the basis of the underlying animal welfare law of the respective countries. Compounds were dissolved in PBS and were s.c. administered with a volume of 5 μl g^−1^ body weight in the indicated doses between 15:00 and 16:00.

### Body composition analysis

Fat and lean tissue mass were measured via nuclear magnetic resonance technology (EchoMRI).

### Glucose- and insulin-tolerance tests (GTT/ITT)

Glucose tolerance was assessed in 6 h fasted mice after i.p injection of 1.75 g glucose per kg body weight (DIO mice). Insulin tolerance was assessed in 6 h fasted mice after i.p. injection of either 0.75 U Insulin per gram body weight (DIO mice) or 1 U Insulin per gram body weight (*db/db* mice) (Humalog, Eli Lilly). Tail vein blood glucose was subsequently measured using a handheld glucometer (TheraSense Freestyle) at baseline and after 15, 30, 60 and 120 min. Data were subsequently graphed as baseline-corrected AOC as previously suggested^36^.

### Plasma creatinine

Plasma creatinine was measured by a fluorometric creatinine assay kit (catalogue no. ab65340, Abcam) based on the manufacturer’s instructions.

### Urinary albumin and creatinine measurement

Spot urine samples were collected from male 49-week-old C57BL/6J mice treated for 14 days with either Vhcl (*n* = 8) or 50 nmol kg^−1^ of GLP-1RA/tesaglitazar (*n* = 5), the GLP-1RA (*n* = 6), Tezaglitazar (*n* = 7) or the fixed dose combination of the GLP-1RA and tesaglitazar (*n* = 8). Frozen urine samples from heterozygous GIPR^dn^ mice in a CD1 background (*n* = 3) served as positive controls. Urinary albumin concentrations were measured using a mouse albumin enzyme-linked immunosorbent assay kit (catalogue no. 80630, Crystal Chem) based on the manufacturer’s instructions. Urinary creatinine levels were quantified using a mouse creatinine enzymatic assay kit (catalogue no. 80350, Crystal Chem) based on the manufacturer’s instructions. For each treatment group, uACR was computed as a ratio of urinary albumin (mg ml^−1^) to urinary creatinine (mg ml^−1^).

### Pancreatic section immunostaining and confocal imaging

Adult pancreata were dissected and fixed in 4% PFA in PBS overnight at 4 °C. The samples were cryoprotected by sequential incubation with 7.5, 15 and 30% sucrose-PBS solutions at room temperature (2 h incubation for each solution). Pancreata were then incubated in 30% sucrose and tissue embedded medium (1:1) at 4 °C overnight. Tissues were then embedded in a cryoblock using a tissue-freezing medium, and stored at −80 °C. From each sample, 20-μm-thick sections were cut, mounted on a glass slide and dried for 10 min at room temperature before being used or stored at −20 °C.

Following three washes with 1× PBS, the cryosections were permeabilized with 0.2–0.15% Triton X-100 in H_2_O for 30 min. The sections were then blocked in blocking solution (PBS, 0.1% Tween-20, 1% donkey serum, 5% FCS) for 1 h, followed by incubation with the primary antibodies overnight at 4 °C. The following primary antibodies were used: insulin (Cell Signaling, no. 3014, 1:300), glucagon (TAKARA, no. M182, 1:3,500) and somatostatin (Invitrogen, no. MA5-16987, 1:300). After being rinsed three times and washed three times with 1× PBS, the sections were incubated with secondary antibodies in the blocking buffer. We used the following secondary antibodies: antirabbit-Alexa Flour 488 (Invitrogen, no. A11055, 1:800), antiguinea pig-Cy™3 (Dianova/Jackson, no. 706-165-148, 1:800) and anti-goat-Alexa Flour 633 (Invitrogen, no. A21082, 1:800). After 4–5 h of incubation with the secondary antibodies at room temperature, the pancreatic sections were subsequently stained with DAPI (1:500 in 1× PBS) for 30 min at room temperature, then rinsed and washed three times with 1× PBS and mounted. The images were obtained by using a Leica microscope of the type DMI 6000 and Leica’s LAS AF software. ImageJ and/or LAS AF were used to analyse the images. Mean signal intensity per islet area of confocal images were used to quantify the hormone contents. Nine to 12 islets were quantified for each animal.

### Measurement of islet size area

Dissected pancreata were fixed in Formalin (Formalin 10% neutral buffered, HT501128, Sigma-Aldrich) for 24 h at room temperature and standardly processed for paraffin embedding (Tissue Tec VIP.6, Sakura Europe) Paraffinized pancreata were exhaustively cross-sectioned into four parallel, equidistant slices per case. Maintaining their orientation, the tissue slices were vertically embedded in paraffin. After a costaining for insulin (monoclonal rabbit anti-insulin, no. 3014, Cell Signaling 1:300; AlexaFluor750-conjugated goat antirabbit, A21039, Invitrogen 1:100) and for glucagon (polyclonal guinea pig anti-glucagon, M182, Takara 1:3,500; goat antiguinea pig AlexaFluor555, A21435, Invitrogen 1:200) nuclei were labelled with Hoechst33342 (H1399, Thermo Fischer, 7.5 μg ml^−1^). The stained tissue sections were scanned with an AxioScan.Z1 digital slide scanner (Zeiss, ZEN Blue v.3.5) equipped with a ×20 magnification objective. Insulin expression cells were performed on the entire tissue sections by using image analysis software Definiens Developer XD2 (Definiens AG, v.2.7.0). The insulin expressing cells were classified automatically using the fluorescence intensity. β cell volume (mg) was calculated by multiplying the detected relative insulin-positive cell area by total pancreatic weight. α cell volume (mg) was similarly calculated on the basis of the detected glucagon-positive cell area. The area of the pancreatic islet was calculated on the basis of the insulin and glucagon-positive area.

### Dynamic glucose-stimulated insulin secretion in primary murine islets

After isolation and overnight incubation in RPMI (10% foetal bovine serum (FBS), 1% penicillin/streptomycin), 75 islets were handpicked and placed into chambers containing 2.7 mM glucose Krebs–Ringer phosphate-HEPES (KRPH) buffer (140 mM NaCl, 4.7 mM KCl, 1.5 mM CaCl_2_, 1 mM NaH_2_PO4, 1 mM MgSO_4_, 2 mM NaHCO_3_, 5 mM HEPES and 0.1% FA-free BSA (pH 7.4)) with 100 μl of Bio-Gel P-4 Media (Bio-Rad). Islets were equilibrated for 48 min and then perifused in intervals on the basis of the experimental conditions. All treatments were prepared in KRPH buffer +0.1% BSA. Islet proteins were extracted in acid ethanol to assess total insulin levels. Insulin secretion was assayed with Lumit Insulin Immunoassay Kit (Promega, CS3037A01) and measured using the EnVision plate reader (PerkinElmer).

### Cell culture and transfection

HEK293T cells (ATCC, catalogue no. CRL-3216) or MIN6 cells (AddexBio, catalogue no. C0018008,) were cultured in DMEM (catalogue no. 11995073, Life Technologies) supplemented with 10% (HEK293T) or 15% (MIN6) heat-inactivated FBS (catalogue no. 10500064, Life Technologies), 100 IU ml^−1^ of penicillin and 100 μg ml^−1^ of streptomycin solution (Pen-Strep, catalogue no. P4333, Sigma-Aldrich). HEK293T cells (700,000 per well) were seeded in six-well plates and incubated to 70% confluency in DMEM (10% FBS, 1% Pen/Strep). Twenty-four hours following seeding, transient transfections were performed using Lipofectamine 2000 (catalogue no. 11668019, Invitrogen) according to the manufacturer’s protocol without including additional transformation carrier DNA.

### Ligand-induced bioluminescence resonance energy transfer (BRET) assays

Twenty-four hours following transfection, HEK293T cells or MIN6 cells were washed with PBS and resuspended in FluoroBrite phenol red-free complete media (catalogue no. A1896701, Life Technologies) containing 5% FBS and 2 mM of l-glutamine (catalogue no. 25030081, Life Technologies). 100,000 cells per well were plated into poly-d-lysine-coated (catalogue no. P6403, Sigma-Aldrich) 96-well white polystyrene LumiNunc plates (catalogue no. 10072151, Thermo Fisher Scientific). After 24 h, the media was replaced with PBS (catalogue no. 10010056, Gibco) containing 10 μM of coelenterazine-h (catalogue no. S2011, Promega) or 1:500 dilution of NanoGlo (catalogue no. N1110, Promega). BRET measurements were taken every 30 s–2 min using a PHERAstar FS multi-mode microplate reader. Baseline measurements were taken after an initial 5 min of incubation with coelenterazine-h or NanoGlo-containing PBS after which cells were then treated with either vehicle (PBS) or respective ligands. The resulting ligand-specific ratiometric BRET signals were normalized to vehicle producing the ‘ligand-induced BRET ratio’^37^, followed by an additional normalization step to well-specific baseline readings. Ligand-induced measurements on the temporal scale is represented as the subsequent measurement after time point zero. Positive or negative incremental AUC (+iAUC/−iAUC) were calculated where noted. Each experiment was independently performed at least three times, each with at least three technical replicates for each group.

### Receptor signalling and trafficking BRET assays

Untagged hGLP-1R was purchased from Sino Biological Inc. (catalogue no. HG13944-UT), hGLP-1R-GFP was a kind gift from D. Hodson (University of Birmingham), and hGLP-1R-Rluc8 was a kind gift from P. Sexton (Monash University, Melbourne, Australia). Gα_s_ recruitment to the GLP-1R-GFP was quantified using mini-Gα_s_, a protein probe that translocates to ligand-bound Gα_s_-coupled G-protein coupled receptors^38^. NES-Nluc-MiniGα_s_ was a gift from K. Pfleger (Harry Perkins Institute of Medical Research, Nedlands, Western Australia, Australia). Intracellular cAMP was measured using the YFP-Epac-Rluc CAMYEL sensor^39^. hGLP-1R-RLUC8 internalization was quantified using the intracellular plasma membrane marker Venus-KRAS^40^. Venus-KRAS was a kind gift from K. Pfleger. GLP-1R-Rluc8 lysosomal colocalization was measured using Lamp1-mNeonGreen. Lamp1-mNeonGreen was a gift from D. Gadella (Addgene plasmid no. 98882). Time-dependent RXR/PPARγ heterodimerization was measured using RXR-Rluc8 and PPARγ2-YFP, both of which were kind gifts from V. Ollendorf^41^.

### Gene expression analysis

For assessment of acute drug effects, animals were treated with the respective compounds 4 h before tissue harvesting. Dissected tissues were frozen immediately on dry ice, and RNA was isolated using RNeasy Mini Kits (Qiagen). mRNA levels were determined using TaqMan probes for fatty acid binding protein 4 (*Fabp4*, Mm00445878_m1), perilipin-2 (*Plin2*, Mm00475794_m1), aryl hydrocarbon receptor nuclear translocator-like protein 1 (Arntl, Mm00500223_m), semaphorin 3 C (*Sema3c*, Mm00443121_m1) and hypoxanthine phosphoribosyltransferase 1 (*Hp*rt, Mm03024075_m1) in custom-made low density array cards (Thermo Fisher) or in single assays on a QuantStudio 7 Real-Time PCR system. Target gene expression was normalized to HPRT and fold change was calculated relative to vehicle-treated controls.

### Proteomics sample preparation

Frozen tissue samples were homogenized in 0.1 M Tris-HCl, pH 7.6 and 2% SDC, heated for 5 min at 95 °C and sonicated (Diagenode Bioruptor at high intensity, 15 × 30 s). Then 50 µg of protein was reduced and alkylated with 10 mM TCEP,40 mM CAA for 5 min at 40 °C in the dark. Samples were digested with trypsin and LysC 1:50 (enzyme:protein) overnight at 37 °C. Digested peptides were acidified to a final concentration of 1% TFA and loaded onto activated triple layer styrenedivinylbenzene–reversed phase sulfonated (SDB–RPS, 3M Empore) STAGE tips. STAGE tips were washed with 100 µl ethylacetate 1% TFA, 100 µl of 30% methanol 1% TFA and 150 µl of 0.2% TFA. Peptides were eluted with 60 µl of SDB–RPS elution buffer (80% ACN, 5% NH4OH), concentrated in a SpeedVac for 40 min at 45 °C and dissolved in 10 µl of MS loading buffer (2% ACN, 0.1% TFA).

### LC–MS/MS analysis

Single-shot measurements were performed with 500 ng of purified peptides, determined by absorbance at 280 nm on a NanoDrop 2000. Peptides were loaded onto a 50-cm column, packed in-house with 1.9 µm C18 ReproSil particles (Dr. Maisch GmbH) with an EASY-nLC 1200 system (Thermo Fisher Scientific). Column temperature was kept at 60 °C using a column oven. Peptides were eluted over 60 min using a binary buffer system consisting of buffer A (0.1% formic acid) and buffer B (80% ACN, 0.1% formic acid). In brief, the gradient started with 5% buffer B and increased stepwise to 45% over 45 min, followed by a wash-out at 95% buffer B, all at a flowrate of 300 nl min^−1^. Peptides were then transferred to the gas phase using electrospray ionization, prefiltered by a FAIMS device (coefficient of variation −50 V) before entering the Orbitrap Exploris 480 (Thermo Fisher Scientific) mass spectrometer. A data-independent (DIA) acquisition method was used, in which one full scan (300–1,650 *m/z*, maximum ion fill time of 45 ms, normalized AGC target 300%, *R* = 120.000 at 200 *m/z*) was followed by 66 tMS2 fragment scans of unequally spaced windows with 1 Th overlap, covering the same *m/z* range (fill time 22 ms, normalized AGC target 1,000%, normalized high-collision density energy of 30%, *R* = 15.000).

### Pharmacokinetic analysis

Plasma concentration-time profiles were analysed in C57BL6J mice and Sprague Dawley rats by a non-compartmental method using sparse sampling in Pharsight Phoenix WinNonLin v.6.4 software, and the resulting terminal half-life (*T*_1/2_), maximum plasma concentration (*C*_max_), time for maximum plasma concentration (*T*_max_), and AUC from zero to last valid measurable concentration-time point (AUC_0-t_) were determined. Criteria for estimation of *T*_1/2_ were at least three concentration-time points in the terminal phase not including *C*_max_, with an *R*^2^ ≥ 0.85.

### Immunhistochemical analysis

For cFOS analysis, mice were treated s.c. with a single dose of 150 nmol kg^−1^. Tissues were harvested 90 min following drug exposure. Fixed brains were coronally cryosectioned and 35-μm-thick slices were immunolabelled with the monoclonal rabbit anti-cFOS antibody (1:400, Invitrogen, no. MA5-15055) and the antirabbit Alexa546 secondary antibody (1:4,000, Invitrogen, no. A10040). According to the Allen mouse brain atlas, the ARC and the dorsomedial and ventromedial hypothalamic nuclei were captured at ×20 magnification using the Leica SP8 confocal microscope. In each region, the number of cFOS positive cells was counted in a blinded manner using Fiji/ImageJ software. For assessment of central nervous system drug appearance, mice (*n* = 3–4 each group) were treated with a single s.c. dose of the Cy5-labelled peptides (150 nmol kg^−1^). After 90 min, tissues were harvested and processed similar to the cFOS analysis.

### Bioinformatic workflow and proteomic data analysis

DIA raw files were processed using Spectronaut v.14.5.200813.47784 (ref. ^42^) using the directDIA function with default settings. Raw files from samples of the same tissue were processed together and searched against the mouse UniProt FASTA database (September 2014, 51,210 entries) and the provided MaxQuant contaminants list (245 entries). Spectronaut report files were then loaded into Perseus (v.1.6.2.3). In brief, quantified proteins were filtered for ≥3 valid values in at least one biological condition and annotations mapped from UniProtKB, Gene Ontology and the Kyoto Encyclopedia of Genes and Genomes. Significantly up- or downregulated proteins between the conditions were determined by ANOVA (FDR 0.025).

### Statistics

Statistical analyses were performed using the statistical tools implemented in GraphPad Prism8 (v.8.3.0 or 9.00). All data are shown as mean ± s.e.m. Differences between groups were assessed by one- or two-way ANOVA with time and treatment as covariants followed by Bonferroni’s post hoc multiple comparison testing as indicated in the figure legends. A *P* value <0.05 was considered statistically significant with asterisks indicating *P < 0.05, **P < 0.01 and ***P < 0.001.

### Reporting summary

Further information on research design is available in the Nature Research Reporting Summary linked to this article.

## Supplementary information

Reporting Summary.

Supplementary Data 1 Original as well as cropped pictures of islet histology shown in Extended Data Fig. 2i.

Supplementary Data 2 Original pictures of cFOS and Cy5 drug appearance shown in Extended Data Fig. 4a–c, including replicates.

Supplementary Data 3 Original pictures of cFOS shown in Fig. 7a,b, including replicates.

Supplementary Data 4 Original histology pictures.

## Data Availability

The data used for the statistical analysis are available in the data source file, along with the GraphPad Prizm-derived report on the statistical analysis. The statistical report contains the mean difference between the treatment groups, the 95% confidence intervals, the significance summary, and the exact *P* values (unless *P* < 0.0001). Additional raw data are available from the corresponding author upon reasonable request. Proteomic data shown Fig. 7c–h are available via ProteomeXchange under the identifier PXD033653. Source data are provided with this paper.

## Source data

Source Data Fig. 1 Statistical source data for Fig. 1.
Source Data Fig. 2 Statistical source data for Fig. 2.
Source Data Fig. 3 Statistical source data for Fig. 3.
Source Data Fig. 4 Statistical source data for Fig. 4.
Source Data Fig. 5 Statistical source data for Fig. 5.
Source Data Fig. 6 Statistical source data for Fig. 6.
Source Data Fig. 7 Statistical source data for Fig. 7.
Source Data Extended Data Fig. 1 Statistical source data for Extended Data Fig. 1.
Source Data Extended Data Fig. 2 Statistical source data for Extended Data Fig. 2.
Source Data Extended Data Fig. 3 Statistical source data for Extended Data Fig. 3.
Source Data Extended Data Fig. 4 Statistical source data for Extended Data Fig. 4.

## Extended data

are available for this paper at 10.1038/s42255-022-00617-6.
