# Supplementary material for: GLP-1-mediated delivery of tesaglitazar improves obesity and glucose metabolism in male mice
Source: Nat Metab. 2022 Aug 22;4(8):1071–83. doi: 10.1038/s42255-022-00617-6 (PMC9398908; doi:10.1038/s42255-022-00617-6)
Supplement: Supplementary file 1 — Reporting Summary. [file 42255_2022_617_MOESM1_ESM.pdf]

nature portfolio

Corresponding author(s): Timo D. Müller

Last updated by author(s): Jun 26, 2022

Reporting Summary

Nature Portfolio wishes to improve the reproducibility of the work that we publish. This form provides structure for consistency and transparency in reporting. For further information on Nature Portfolio policies, see our Editorial Policies and the Editorial Policy Checklist.

Statistics

For all statistical analyses, confirm that the following items are present in the figure legend, table legend, main text, or Methods section.

n/a	Confirmed	
☐	☒	The exact sample size (n) for each experimental group/condition, given as a discrete number and unit of measurement
☐	☒	A statement on whether measurements were taken from distinct samples or whether the same sample was measured repeatedly
☐	☒	The statistical test(s) used AND whether they are one- or two-sided<br>*Only common tests should be described solely by name; describe more complex techniques in the Methods section.*
☐	☒	A description of all covariates tested
☐	☒	A description of any assumptions or corrections, such as tests of normality and adjustment for multiple comparisons
☐	☒	A full description of the statistical parameters including central tendency (e.g. means) or other basic estimates (e.g. regression coefficient) AND variation (e.g. standard deviation) or associated estimates of uncertainty (e.g. confidence intervals)
☐	☒	For null hypothesis testing, the test statistic (e.g. F, t, r) with confidence intervals, effect sizes, degrees of freedom and P value noted<br>*Give P values as exact values whenever suitable.*
☒	☐	For Bayesian analysis, information on the choice of priors and Markov chain Monte Carlo settings
☒	☐	For hierarchical and complex designs, identification of the appropriate level for tests and full reporting of outcomes
☒	☐	Estimates of effect sizes (e.g. Cohen's d, Pearson's r), indicating how they were calculated

Our web collection on statistics for biologists contains articles on many of the points above.

Software and code

Policy information about availability of computer code

Data collection	Histological slides in liver, iWAT, kidney and heart were scanned at the Axioscan Z1 using ZEN Blue, Zeiss, Version3.5. Proteomic data were processed with Perseus (v.1.6.2.3).cFOS was imaged using LAS X (version 3.5.7.23225, Leica Microsystems CMS GmbH)
Data analysis	Histological slides in liver, iWAT, kidney and heart were analyzed using NetScope Viewer, Net-Base, Version 1.9 and Definiens Developer XD2 (Definiens AG, Germany, Version 2.7.0). Statistical analysis were made using GraphPad Prism (Version 8.3.0 or 9.00). cFOS data were analyzed using ImageJ (version 1.53c, NIH, USA)

For manuscripts utilizing custom algorithms or software that are central to the research but not yet described in published literature, software must be made available to editors and reviewers. We strongly encourage code deposition in a community repository (e.g. GitHub). See the Nature Portfolio guidelines for submitting code & software for further information.

Data

Policy information about availability of data

All manuscripts must include a data availability statement. This statement should provide the following information, where applicable:

- Accession codes, unique identifiers, or web links for publicly available datasets
- A description of any restrictions on data availability
- For clinical datasets or third party data, please ensure that the statement adheres to our policy

The data used for the statistical analysis are available in the data source file, along with the GraphPad Prizm-derived report on the statistical analysis. The statistical report contains the mean difference between the treatment groups, the 95% confidence intervals, the significance summary, and the exact p-values (unless p<0.0001). Additional raw data are available from the corresponding author upon reasonable request. Proteomic data shown Fig. 7 are available via ProteomeXchange under the identifier PXD033653.

Field-specific reporting

Please select the one below that is the best fit for your research. If you are not sure, read the appropriate sections before making your selection.

- [x] Life sciences
- [ ] Behavioural & social sciences
- [ ] Ecological, evolutionary & environmental sciences

For a reference copy of the document with all sections, see nature.com/documents/nr-reporting-summary-flat.pdf

Life sciences study design

All studies must disclose on these points even when the disclosure is negative.

Sample size	For animal studies, sample sizes were calculated based on a power analysis assuming that a greater or equal (>/=) 5 g difference in body weight between genotypes can be assessed with a power of >/= 75% when using a 2-sided statistical test under the assumption of a standard deviation of 3.5 and an alpha level of 0.05. Sample sizes for In vitro studies were based on preliminary experience and were performed under the assumption that a 1.3-fold difference between treatment groups can be assessed with a power of >/= 80% when using a 2-sided statistical test under the assumption of a standard deviation of 0.13 and an alpha level of 0.05.
Data exclusions	No data were excluded from the analysis unless scientific (sign. outlier based on Grubbs test) or animal welfare reasons (injury due to fighting) demanded exclusion. Outliers are stated in the data source file.
Replication	In vitro data have been replicated independently as indicated in the figure legends. Statements of successful replication of in vitro data are indicated in the figure legends or the methods section. Validity of vivo data on glucose metabolism are verified by several independent experiments using doses between 50 and 0.5 nmol/kg as shown in Fig. 3. Validity of body weight effects in WT mice have been confirmed by two independent studies, performed in two independent laboratories.
Randomization	Animals were either randomly assigned into treatment groups to generate starting groups of equal body weight and body composition, or were grouped based on their genotype (WT or KO). At study start, only age-matched mice were included in the studies. There were no other covariats controlled. For in vitro experiments, cells were equally and randomly distributed across plates.
Blinding	For in vivo and in vitro studies, drugs were aliquoted by a lead scientist in number-coded vials and most, but not all, handling investigators were blinded to the treatment condition. Analyses of glucose and insulin tolerance were performed by experienced research assistants which did not know prior treatment conditions. BRET assays in Fig. 1 were conducted by a single investigator and were hence not blinded.

Reporting for specific materials, systems and methods

We require information from authors about some types of materials, experimental systems and methods used in many studies. Here, indicate whether each material, system or method listed is relevant to your study. If you are not sure if a list item applies to your research, read the appropriate section before selecting a response.

Materials & experimental systems

n/a	Involved in the study	
[ ]	[x]	Antibodies
[ ]	[x]	Eukaryotic cell lines
[x]	[ ]	Palaeontology and archaeology
[ ]	[x]	Animals and other organisms
[x]	[ ]	Human research participants
[x]	[ ]	Clinical data
[x]	[ ]	Dual use research of concern

Methods

n/a	Involved in the study	
[x]	[ ]	ChIP-seq
[x]	[ ]	Flow cytometry
[x]	[ ]	MRI-based neuroimaging

Antibodies

Antibodies used

cFOS (Invitrogen, #MA5-15055; Dilution 1:400)
Anti-rabbit Alexa546 (Invitrogen #A10040; Dilution: 1:4000)
Insulin (Cell Signaling #3014; Dilution: 1:300)
Glucagon (TAKARA #M182; Dilution: 1:3500)
Somatostatin (Invitrogen #MA5-16987; Dilution 1:300)
Anti-rabbit Alexa Flour 488 (Invitrogen #A11055, Dilution: 1:800)
Anti-Guinea pig Cy3 (Dianova/Jackson #706-165-148, Dilution: 1:800)
Anti-goat-Alexa Flour® 633 (Invitrogen, #A21082, Dilution: 1:800)
AlexaFluor750-conjugated goat anti-rabbit (Invitrogen, #A21039, Dilution 1:100)
Goat anti-guinea pig AlexaFluor555 (Invitrogen, #A21435, 1:200)
Anti-rabbit Alexa 546 (Invitrogen, # A10040, 1:4000)

Validation

cFOS (Invitrogen, #MA5-15055): The cFOS monoclonal antibody Invitrogen #MA5-15055 was verified by Relative expression to ensure that the antibody binds to the antigen stated. The antibody shows reactivity in bovine, hamster, human, mouse, pig and rat. The antibody can be used for western blot, immunhistochemistry, immuncytochemistry, flow cytometry and ChIP assays. The antibody does not cross-react with other Fos proteins, including FosB, FRA1 and FRA2. Immunofluorescence analysis of c-Fos was performed using 70% confluent log phase HeLa cells treated with 200 ng/mL EGF for 30 min. The cells were fixed with 4% paraformaldehyde for 10 minutes, permeabilized with 0.1% Triton™ X-100 for 10 minutes, and blocked with 1% BSA for 1 hour at room temperature. The cells were labeled with c-Fos Monoclonal Antibody (T.142.5) (product # MA5-15055) at 1:250 dilution in 0.1% BSA, incubated overnight at 4 degree Celsius and then labeled with Goat anti-Rabbit IgG (H+L) Superclonal™

The anti-rabbit Alexa546 (Invitrogen #A10040): Immunofluorescence analysis of Donkey anti-Rabbit IgG (H+L) Secondary Antibody, Alexa Fluor 546 (Product # A10040) was performed using HepG2 cells stained with alpha-1 antitrypsin Rabbit Polyclonal Primary Antibody (Product # PA5-16661). The cells were fixed with 4% paraformaldehyde for 10 minutes, permeabilized with 0.1% TritonTM X-100 for 10 minutes, blocked with 1% BSA for 1 hour and labeled with 2 µg /mL of rabbit primary antibody for 3 hours at room temperature. Donkey anti-Rabbit IgG (H+L) Secondary Antibody, Alexa Fluor 546 (Product # A10040) was used at a concentration of 4 µg/mL in phosphate buffered saline containing 0.2 % BSA for 45 minutes at room temperature, for detection of alpha-1 antitrypsin in the cytoplasm (Panel a: red). Nuclei (Panel b: blue) were stained with DAPI in SlowFade® Gold Antifade Mountant (Product # S36938). F-actin was stained with Alexa Fluor® 488 Phalloidin (Product # A12379, 1:300) (Panel c: green). Panel d represents the composite image. No nonspecific staining was observed with the secondary antibody alone (panel f), or with an isotype control (panel e). The images were captured at 60X magnification.

Insulin (Cell Signaling #3014) is recommended by the manufacturer for IHC, IF and F. The antibody is specified to react with human, mouse and rat insulin. The antibody was validated using Immunohistochemical analysis of paraffin-embedded mouse pancreas, showing staining of β cells, using Insulin (C27C9) Rabbit mAb.

Glucagon (TAKARA #M182) is a polyclonal antibody validated and recommended by the manufacturer for IHC. The antibody is raised against the peptide [HSQGTFTSDYSKYLDSRRAQDFVQWLMNT] of human Glucagon conjugated with KLH as an immunogen. The antibody is validated for reactivity with human and mouse and can be applied towards the immunohistochemical (IHC) detection of human glucagon in paraffin-embedded tissue and frozen tissue sections. The polyclonal anti-glucagon is raised in guinea pig against a human glucagon immunogen. For validation, the lyophilized antibody was dissolved in 50 µl of specifed water. The antibody dilutions were applied for ELISA assay by colorimetric detection using a microtiter plate immobilized with human Glucagon peptide. The expected antibody titration was obtained.

Somatostatin (Invitrogen #MA5-16987) is a monoclonal antibody validated and recommended by the manufacturer for the specific reaction with SST in guinea pig, human, mouse, pig and rat. The antibody is recommended for use in immunhistochemistry and immunocytochemistry. The antibody has been verified by several publications (e.g. PMID: 32371554, PMID: 30930126)

Anti-rabbit Alexa Flour 488 (Invitrogen #A11055) is recommended by the manufacturer to be used in IHC and FC. The antibody has been used in 2,233 manuscripts. To minimize cross-reactivity, these donkey anti-goat IgG whole antibodies have been cross-adsorbed against rabbit, rat, mouse, and human IgG. Cross-adsorption or pre-adsorption is a purification step to increase specificity of the antibody resulting in higher sensitivity and less background staining. The secondary antibody solution is passed through a column matrix containing immobilized serum proteins from potentially cross-reactive species. Only the nonspecific-binding secondary antibodies are captured in the column, and the highly specific secondaries flow through. The benefits of this extra step are apparent in multiplexing/multicolor-staining experiments (e.g., flow cytometry) where there is potential cross-reactivity with other primary antibodies or in tissue/cell fluorescent staining experiments where there are may be the presence of endogenous immunoglobulins.Alexa Fluor dyes are among the most trusted fluorescent dyes available today. Invitrogen™ Alexa Fluor 488 dye is a bright, green-fluorescent dye with excitation ideally suited to the 488 nm laser line. For stable signal generation in imaging and flow cytometry, Alexa Fluor 488 dye is pH-insensitive over a wide molar range. Probes with high fluorescence quantum yield and high photostability allow detection of low-abundance biological structures with great sensitivity. Alexa Fluor 488 dye molecules can be attached to proteins at high molar ratios without significant self-quenching, enabling brighter conjugates and more sensitive detection. The degree of labeling for each conjugate is typically 2-8 fluorophore molecules per IgG molecule; the exact degree of labeling is indicated on the certificate of analysis for each product lot.Using conjugate solutions: Centrifuge the protein conjugate solution briefly in a microcentrifuge before use; add only the supernatant to the experiment. This step will help eliminate any protein aggregates that may have formed during storage, thereby reducing nonspecific background staining. Because staining protocols vary with application, the appropriate dilution of antibody should be determined empirically. For the fluorophore-labeled antibodies a final concentration of 1-10 µg/mL should be satisfactory for most immunohistochemistry and flow cytometry applications.

Anti-Guinea pig Cy3 (Dianova/Jackson #706-165-148) is a Cy3-AffiniPure Donkey Anti-Guinea Pig IgG (H+L) (minimal cross-reaction to Bovine, Chicken, Goat, Syrian Hamster, Horse, Human, Mouse, Rabbit, Rat, and Sheep Serum Proteins). The antibody was purified from antisera by immunoaffinity chromatography using antigens coupled to agarose beads.Based on immunoelectrophoresis and/or ELISA, the antibody reacts with whole molecule guinea pig IgG. It also reacts with the light chains of other mouse immunoglobulins. No antibody was detected against non immunoglobulin serum proteins. The antibody has been tested by ELISA and/or solid-phase adsorbed to ensure minimal cross-reaction with guinea pig, chicken, goat, Syrian hamster, horse, human, mouse, rabbit, rat, and sheep serum proteins, but it may cross-react with immunoglobulins from other species.

Anti-goat-Alexa Flour® 633 (Invitrogen, #A21082). The Anti-goat-Alexa Flour® 633 antibody is verified for application in IHC, ICC ,and FC. The antibody has been used in 172 manuscripts. Product Specific Information. To minimize cross-reactivity, these donkey anti-goat IgG (H+L) whole secondary antibodies have been affinity purified and cross-adsorbed against rabbit, rat, mouse, and human IgG. Cross-adsorption or pre-adsorption is a purification step to increase specificity of the antibody resulting in higher sensitivity and less background staining. The secondary antibody solution is passed through a column matrix containing immobilized serum proteins from potentially cross-reactive species. Only the nonspecific-binding secondary antibodies are captured in the column, and the highly specific secondaries flow through. The benefits of this extra step are apparent in multiplexing/multicolor-staining experiments (e.g., flow cytometry) where there is potential cross-reactivity with other primary antibodies or in tissue/cell fluorescent staining experiments where there are may be the presence of endogenous immunoglobulins. Alexa Fluor dyes are among the most trusted fluorescent dyes available today. Invitrogen™ Alexa Fluor 633 dye is a bright, far-red-fluorescent dye with excitation ideally suited to

the 633 nm laser line. For stable signal generation in imaging and flow cytometry, Alexa Fluor 633 dye is pH-insensitive over a wide molar range. Probes with high fluorescence quantum yield and high photostability allow detection of low-abundance biological structures with great sensitivity. Alexa Fluor 633 dye molecules can be attached to proteins at high molar ratios without significant self-quenching, enabling brighter conjugates and more sensitive detection. The degree of labeling for each conjugate is typically 2-8 fluorophore molecules per IgG molecule; the exact degree of labeling is indicated on the certificate of analysis for each product lot. Using conjugate solutions: Centrifuge the protein conjugate solution briefly in a microcentrifuge before use; add only the supernatant to the experiment. This step will help eliminate any protein aggregates that may have formed during storage, thereby reducing nonspecific background staining. Because staining protocols vary with application, the appropriate dilution of antibody should be determined empirically. For the fluorophore-labeled antibodies a final concentration of 1-10 µg/mL should be satisfactory for most immunohistochemistry and flow cytometry applications.

The AlexaFluor750-conjugated goat anti-rabbit (Invitrogen, #A21039) is recommended for WB, FC or ICC/IF. Anti-Rabbit secondary antibodies are affinity-purified antibodies with well-characterized specificity for rabbit immunoglobulins and are useful in the detection, sorting or purification of its specified target. Secondary antibodies offer increased versatility enabling users to use many detection systems (e.g. HRP, AP, fluorescence). They can also provide greater sensitivity through signal amplification as multiple secondary antibodies can bind to a single primary antibody. Most commonly, secondary antibodies are generated by immunizing the host animal with a pooled population of immunoglobulins from the target species and can be further purified and modified (i.e. immunoaffinity chromatography, antibody fragmentation, label conjugation, etc.) to generate highly specific reagents. This secondary antibody is designed for fluorescent Western blot detection on various near-infrared fluorescence instruments. This antibody can be used for multi-color and multiplexing detection when using other antibodies conjugated to compatible Alexa Fluor™ dyes and wavelengths. Other applications of this antibody include immunofluorescent and fluorescent imaging applications when using instrumentation with appropriate excitation and detection capabilities. The antibody has been used in 48 manuscripts

Goat anti-guinea pig AlexaFluor555 (Invitrogen, #A21435) is recommended for ICH and IF/ICC. The antibody has been used in 135 manuscripts. Molecular Probes' fluorescent goat anti–guinea pig IgG anti-body #A21435 is prepared from affinity-purified antibodies that react with IgG heavy chains and all classes of immunoglo- bulin light chains from guinea pig. To minimize cross-reactivity, the anti–guinea pig IgG antibodies have been adsorbed against bovine, chicken, goat, hamster, human, mouse, rabbit, rat and sheep sera prior to labeling. The Alexa Fluor® dyes to which these antibodies are conjugated provide for extraordinarily bright antibody conjugates.
Anti-rabbit Alexa546 secondary antibody (Invitrogen, # A10040) has been used in 446 manuscripts. For Antibody verification, Immunofluorescence analysis of Donkey anti-Rabbit IgG (H+L) Secondary Antibody, Alexa Fluor 546 (Product # A10040) was performed using HepG2 cells stained with alpha-1 antitrypsin Rabbit Polyclonal Primary Antibody (Product # PA5-16661). The cells were fixed with 4% paraformaldehyde for 10 minutes, permeabilized with 0.1% Triton™ X-100 for 10 minutes, blocked with 1% BSA for 1 hour and labeled with 2 µg/mL of rabbit primary antibody for 3 hours at room temperature. Donkey anti-Rabbit IgG (H+L) Secondary Antibody, Alexa Fluor 546 (Product # A10040) was used at a concentration of 4 µg/mL in phosphate buffered saline containing 0.2 % BSA for 45 minutes at room temperature, for detection of alpha-1 antitrypsin in the cytoplasm (Panel a: red). Nuclei (Panel b: blue) were stained with DAPI in SlowFade® Gold Antifade Mountant (Product # S36938). F-actin was stained with Alexa Fluor® 488 Phalloidin (Product # A12379, 1:300) (Panel c: green). Panel d represents the composite image. No nonspecific staining was observed with the secondary antibody alone (panel f), or with an isotype control (panel e). The images were captured at 60X magnification.

Eukaryotic cell lines

Policy information about cell lines

Cell line source(s): HEK293T cells (ATCC, USA). MIN6 cells (AddexBio; #C0018008)

Authentication: Authentication according to the manufacturer's website:
The 293T cell line, originally referred as 293tsA1609neo, is a highly transfectable derivative of human embryonic kidney 293 cells, and contains the SV40 T-antigen.This cell line is competent to replicate vectors carrying the SV40 region of replication. It gives high titers when used to produce retroviruses. It has been widely used for retroviral production, gene expression and protein production. Product related references include DuBridge et al., Mol Cell Biol. 1987 Jan;7(1):379-87 and Pear et al., Proc Natl Acad Sci U S A. 1993 Sep 15;90(18):8392-6. https://www.lgcstandards-atcc.org/Products/All/CRL-3216.aspx?geo_country=de#generalinformation
MIN6 cells were generated by SV40 T-antigen induced cell immortalization of a murine Insulinoma. The adherent murine cell line is of pancreatic islet origin and shows beta cell morphology. The cell line is insulin responsive and expresses glucokinase and Glut2. References confirming authentification include PMID: 25658748, PMID: 26162095, PMID: 26679837, PMID: 26997114, PMID: 28666985, PMID: 29211763
and PMID: 28868828

Mycoplasma contamination: cell lines were free of mycoplasm contaminations

Commonly misidentified lines (See ICLAC register): no misidentified cell lines were used in the manuscript

Animals and other organisms

Policy information about studies involving animals; ARRIVE guidelines recommended for reporting animal research

Laboratory animals: Fig. 2a-h: 34-wk old male C57Bl/6J mice
Fig. 2i: 47-wk old male C57Bl/6J mice
Fig. 2j-m: 30-wk old male C57Bl/6J mice
Fig. 2o-s: 16-wk old male C57Bl/6J mice

Fig. 2t: 44-wk old male C57Bl/6J mice
Fig. 3a-h: 34-wk old male C57Bl/6J mice
Fig. 3i-n: 27-29-wk old male C57Bl/6J mice
Fig. 4a-g: 36-wk old male C57Bl/6J mice
Fig. 5a-g: 36-wk old male C57Bl/6J mice
Fig. 5g-n: 36-wk old male GLP-1R ko C57Bl/6J mice
Fig. 6a-f: 6-wk old male db/db mice
Fig. 7a,b: 47-wk old male C57Bl/6J mice
Fig. 7c-h: 49-wk old male C57Bl/6J mice
Extended Data Figure 1b: 25-30-wk old male C57Bl/6J mice and 8-10-wk old male Sprague Dawley rats
Extended Data Figure 2a,b: 47-wk old male C57Bl/6J mice
Extended Data Figure 2a,b, h-m: 49-wk old male C57Bl/6J mice
Extended Data Figure 3a-c: 34-wk old male C57Bl/6J mice
Extended Data Figure 4a-c: 47-wk old male C57Bl/6J mice
Extended Data Figure 4d: 49-wk old male C57Bl/6J mice
Extended Data Figure 4e: 49-wk old male C57Bl/6J mice

Wild animals

no wild animals were used in the study

Field-collected samples

no field collected animals were used in the study

Ethics oversight

Animal experiments were performed in accordance with European or American guidelines under permission of the local animal ethics
committee of the state of Bavaria or the University of Cincinnati, OH, USA.

Note that full information on the approval of the study protocol must also be provided in the manuscript.
