# Supplementary figures and images for: GLP-1-mediated delivery of tesaglitazar improves obesity and glucose metabolism in male mice (part 1 of 2)
Source: Nat Metab. 2022 Aug 22;4(8):1071–83. doi: 10.1038/s42255-022-00617-6 (PMC9398908; doi:10.1038/s42255-022-00617-6)

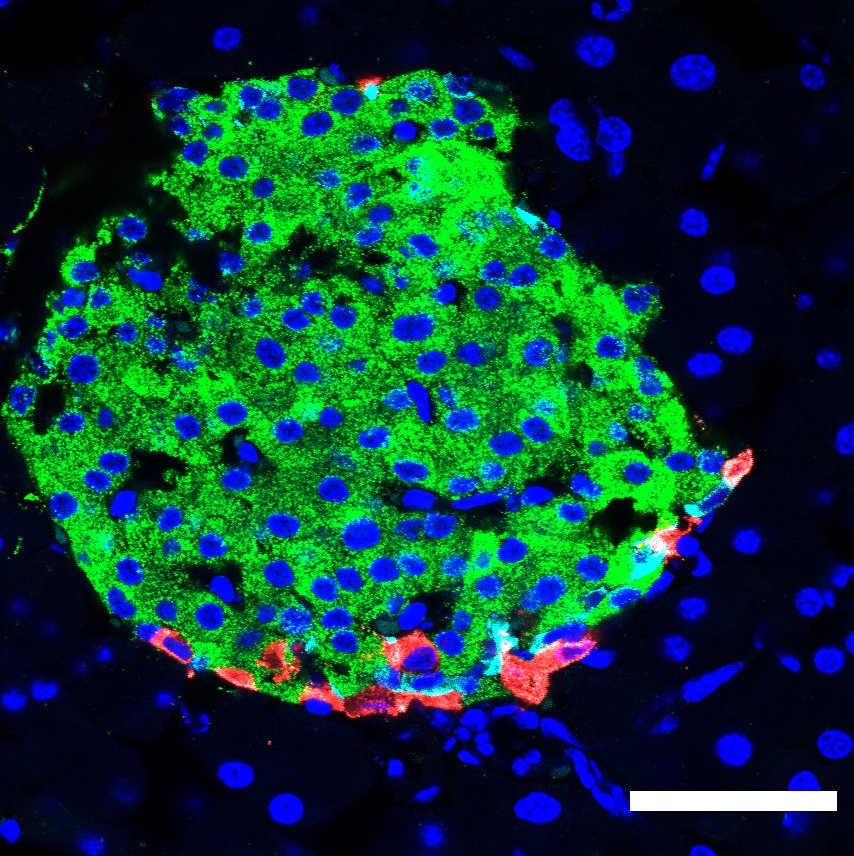

Supplement: Supplementary file 2 — Original as well as cropped pictures of islet histology shown in Extended Data Fig. 2i. [file 42255_2022_617_MOESM2_ESM.zip › Suppl_Fig_2i-islet_histology_pictures/All for Ilustrator/Composite dapi2 Tesaglitazar.jpg]

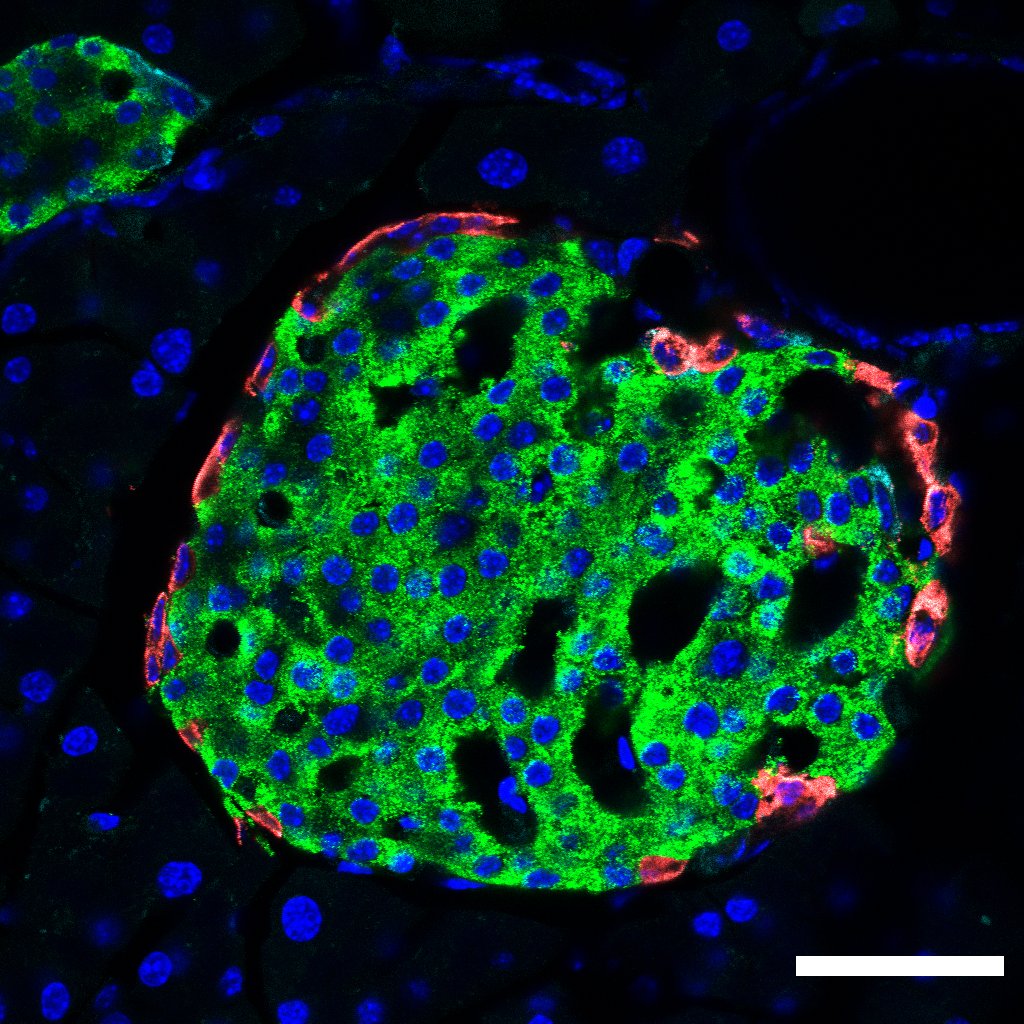

Supplement: Supplementary file 2 — Original as well as cropped pictures of islet histology shown in Extended Data Fig. 2i. [file 42255_2022_617_MOESM2_ESM.zip › Suppl_Fig_2i-islet_histology_pictures/All for Ilustrator/Composite dapi2 GLP-1RA+Tesa.jpg]

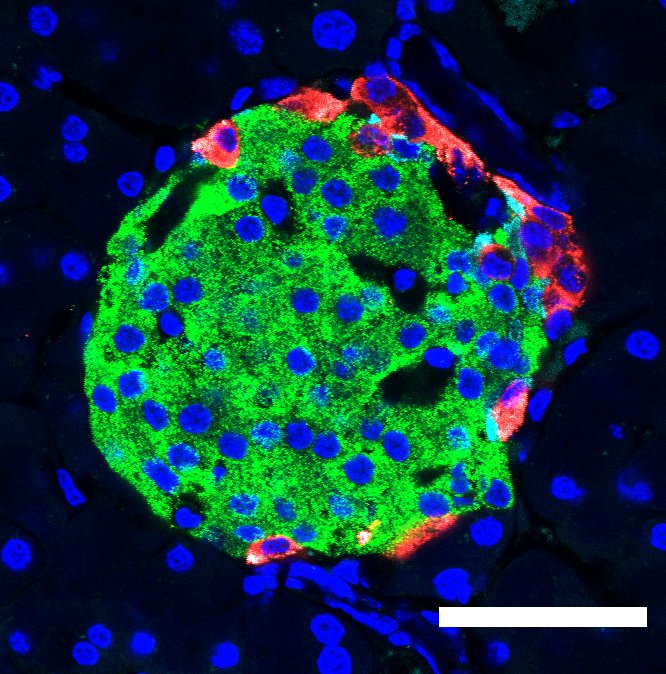

Supplement: Supplementary file 2 — Original as well as cropped pictures of islet histology shown in Extended Data Fig. 2i. [file 42255_2022_617_MOESM2_ESM.zip › Suppl_Fig_2i-islet_histology_pictures/All for Ilustrator/Composite dapi2 GLP-1RA:Tesa.jpg]

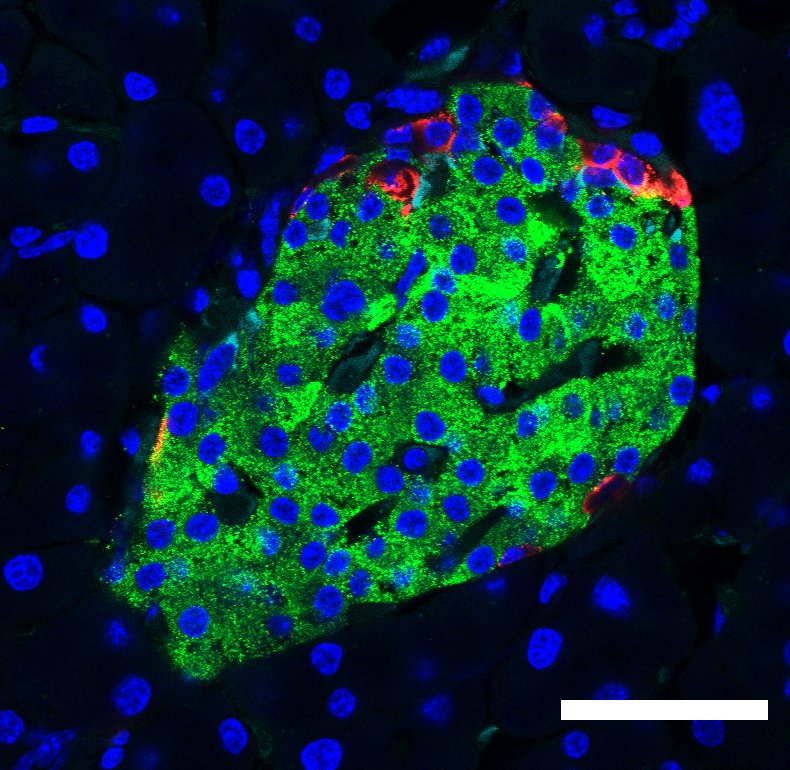

Supplement: Supplementary file 2 — Original as well as cropped pictures of islet histology shown in Extended Data Fig. 2i. [file 42255_2022_617_MOESM2_ESM.zip › Suppl_Fig_2i-islet_histology_pictures/All for Ilustrator/Composite dapi2 GLP-1RA.jpg]

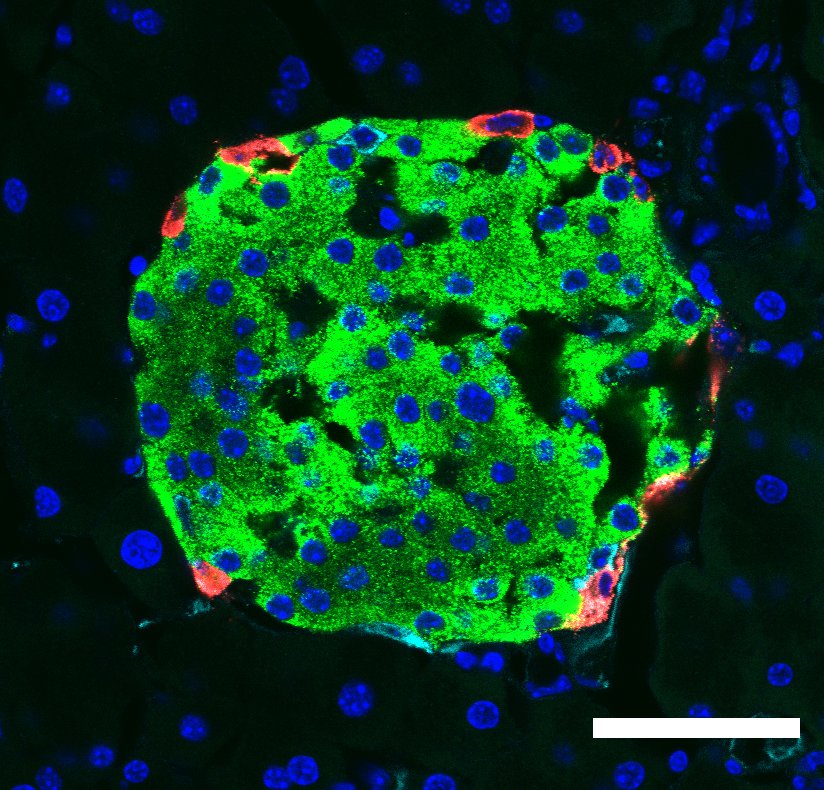

Supplement: Supplementary file 2 — Original as well as cropped pictures of islet histology shown in Extended Data Fig. 2i. [file 42255_2022_617_MOESM2_ESM.zip › Suppl_Fig_2i-islet_histology_pictures/All for Ilustrator/Composite dapi2 Vehicle.jpg]

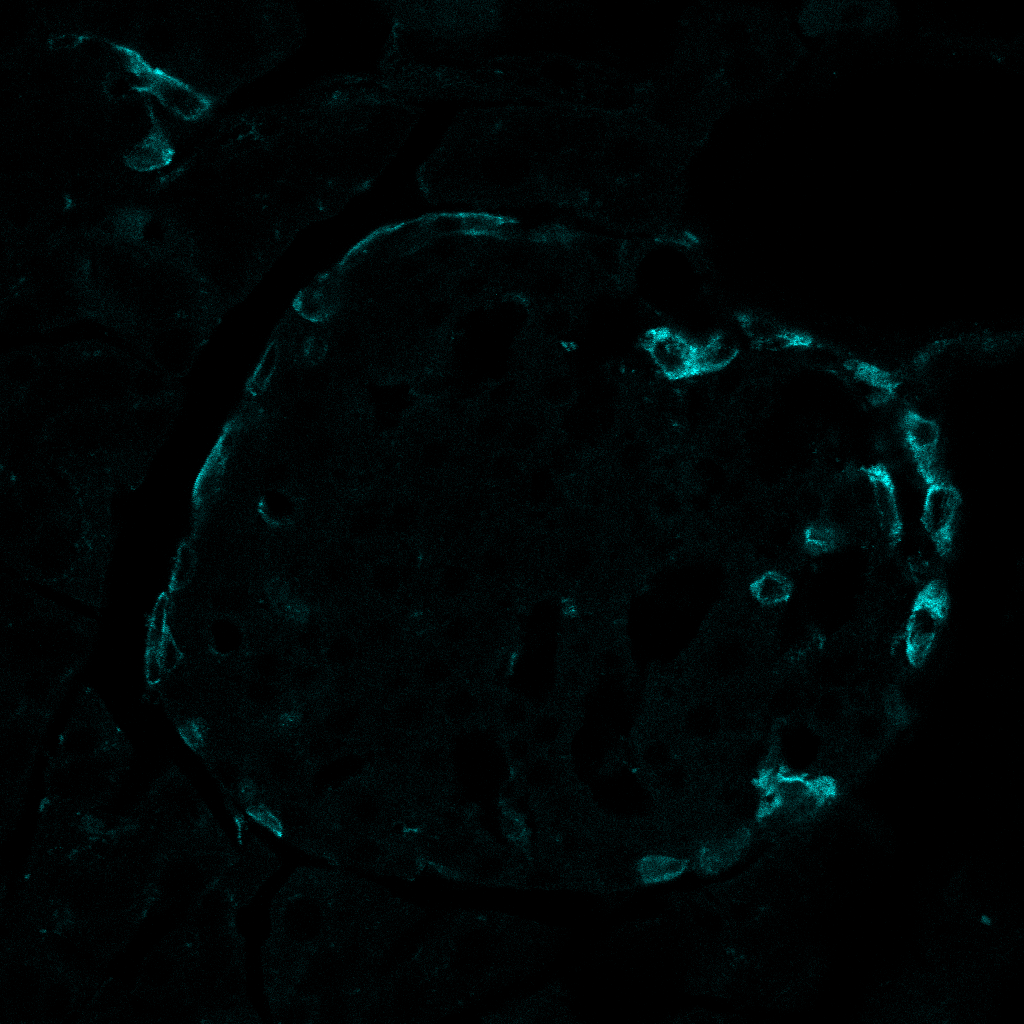

Supplement: Supplementary file 2 — Original as well as cropped pictures of islet histology shown in Extended Data Fig. 2i. [file 42255_2022_617_MOESM2_ESM.zip › Suppl_Fig_2i-islet_histology_pictures/GLP-1RA+Tesaglitazar/original/sst.tif]

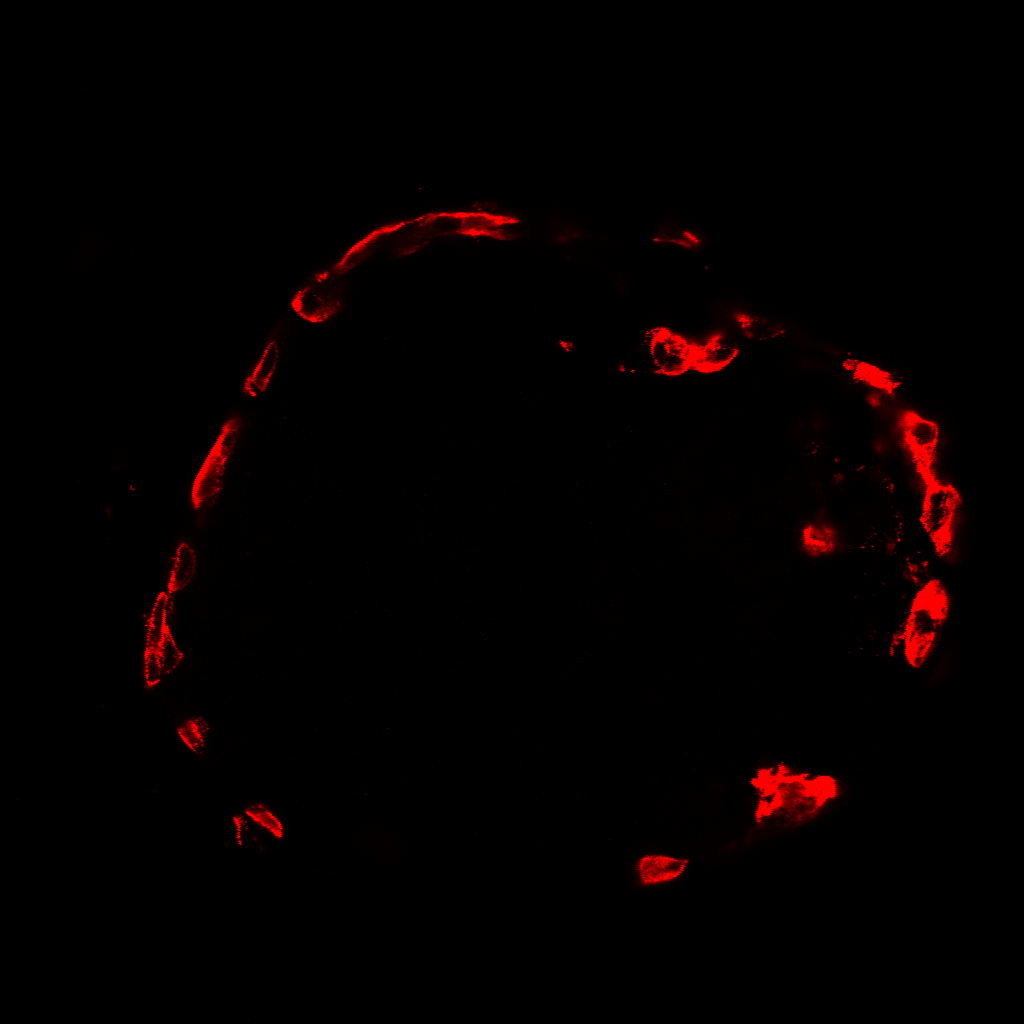

Supplement: Supplementary file 2 — Original as well as cropped pictures of islet histology shown in Extended Data Fig. 2i. [file 42255_2022_617_MOESM2_ESM.zip › Suppl_Fig_2i-islet_histology_pictures/GLP-1RA+Tesaglitazar/original/gcg.tif]

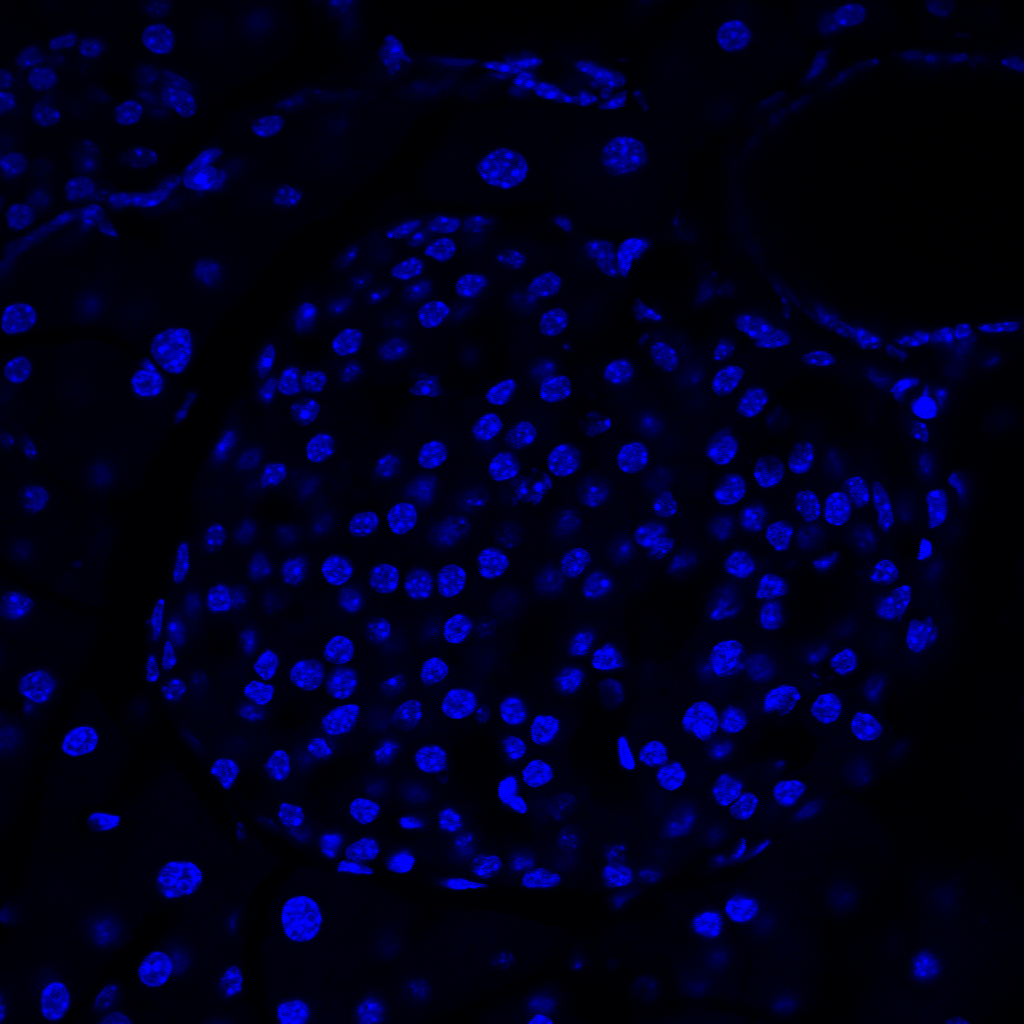

Supplement: Supplementary file 2 — Original as well as cropped pictures of islet histology shown in Extended Data Fig. 2i. [file 42255_2022_617_MOESM2_ESM.zip › Suppl_Fig_2i-islet_histology_pictures/GLP-1RA+Tesaglitazar/original/dapi.tif]

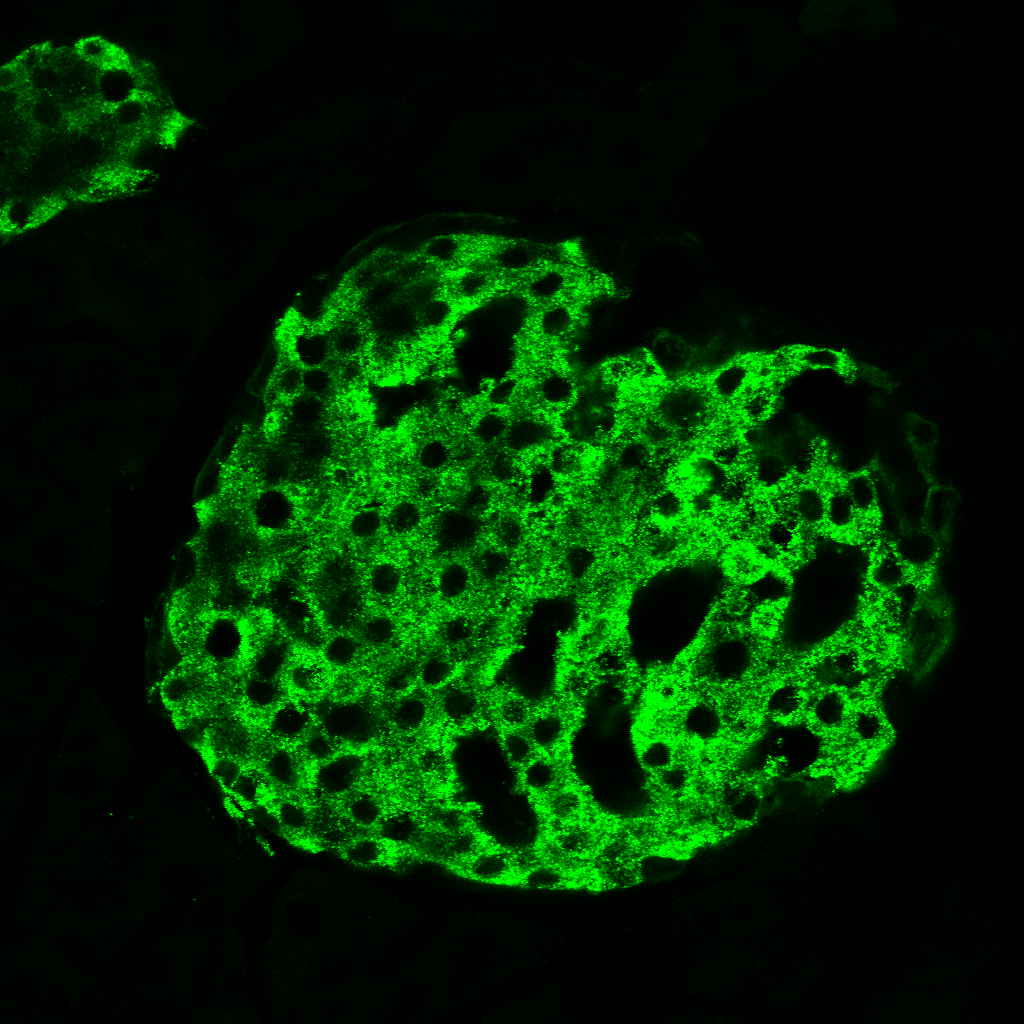

Supplement: Supplementary file 2 — Original as well as cropped pictures of islet histology shown in Extended Data Fig. 2i. [file 42255_2022_617_MOESM2_ESM.zip › Suppl_Fig_2i-islet_histology_pictures/GLP-1RA+Tesaglitazar/original/ins.tif]

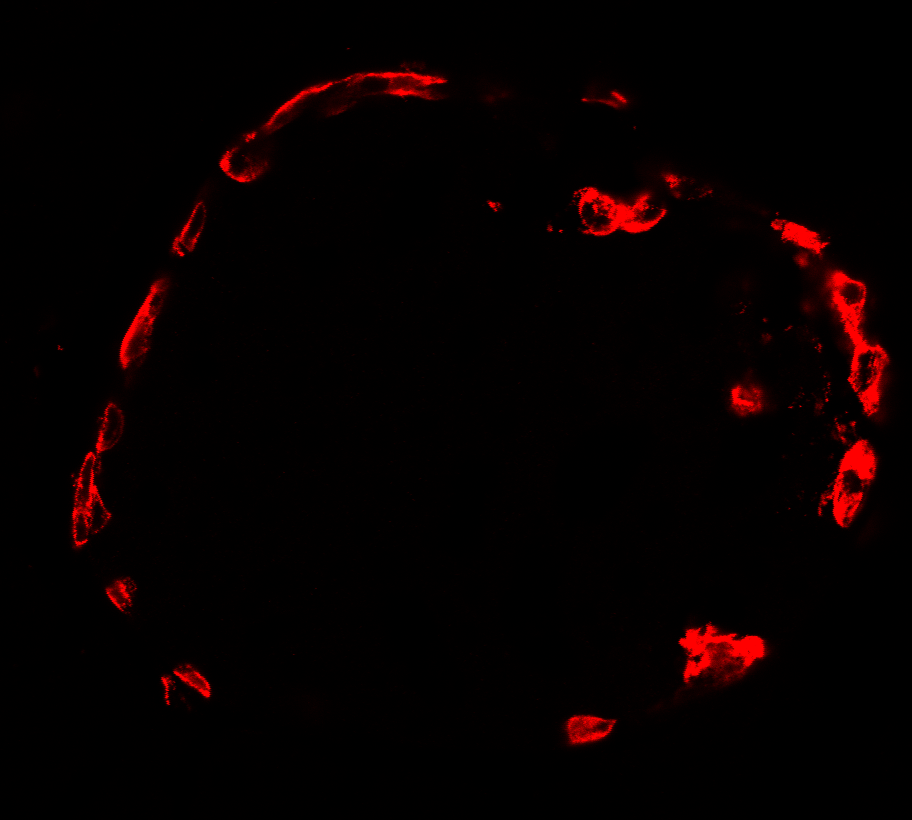

Supplement: Supplementary file 2 — Original as well as cropped pictures of islet histology shown in Extended Data Fig. 2i. [file 42255_2022_617_MOESM2_ESM.zip › Suppl_Fig_2i-islet_histology_pictures/GLP-1RA+Tesaglitazar/Cropped/gcg cropped.tif]

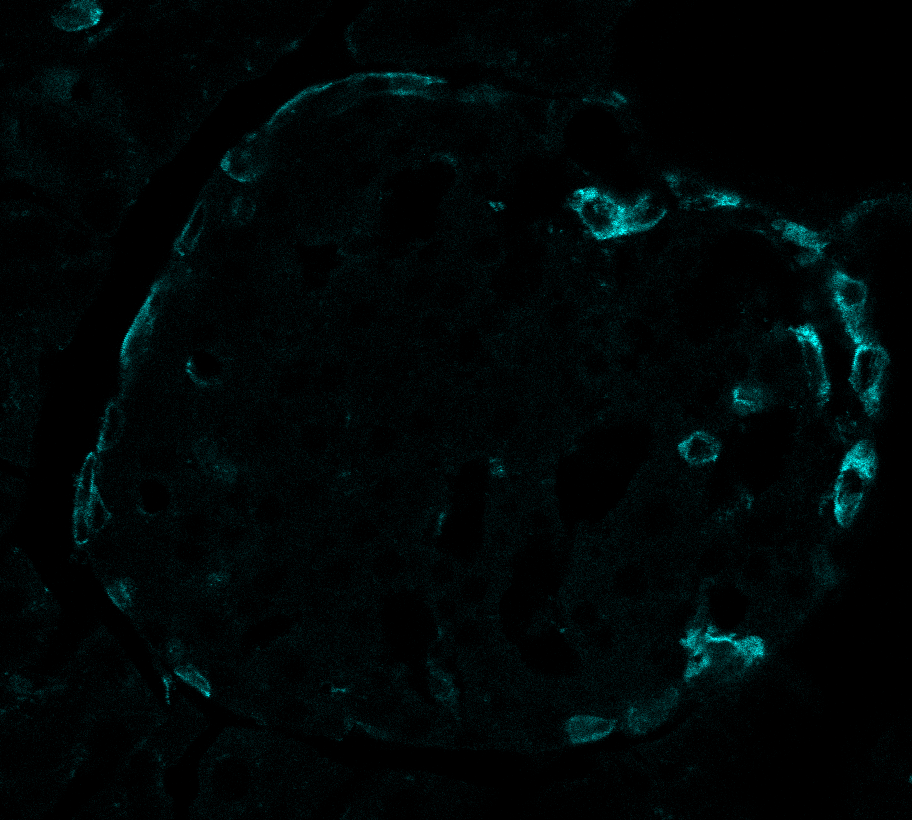

Supplement: Supplementary file 2 — Original as well as cropped pictures of islet histology shown in Extended Data Fig. 2i. [file 42255_2022_617_MOESM2_ESM.zip › Suppl_Fig_2i-islet_histology_pictures/GLP-1RA+Tesaglitazar/Cropped/sst cropped.tif]

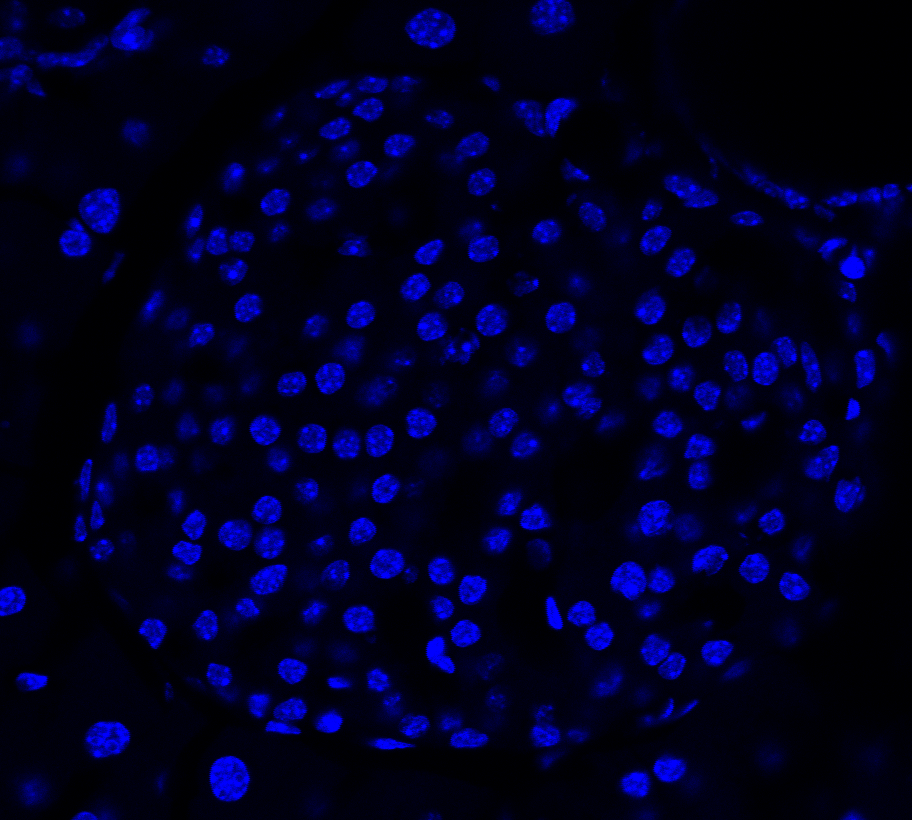

Supplement: Supplementary file 2 — Original as well as cropped pictures of islet histology shown in Extended Data Fig. 2i. [file 42255_2022_617_MOESM2_ESM.zip › Suppl_Fig_2i-islet_histology_pictures/GLP-1RA+Tesaglitazar/Cropped/dapi cropped.tif]

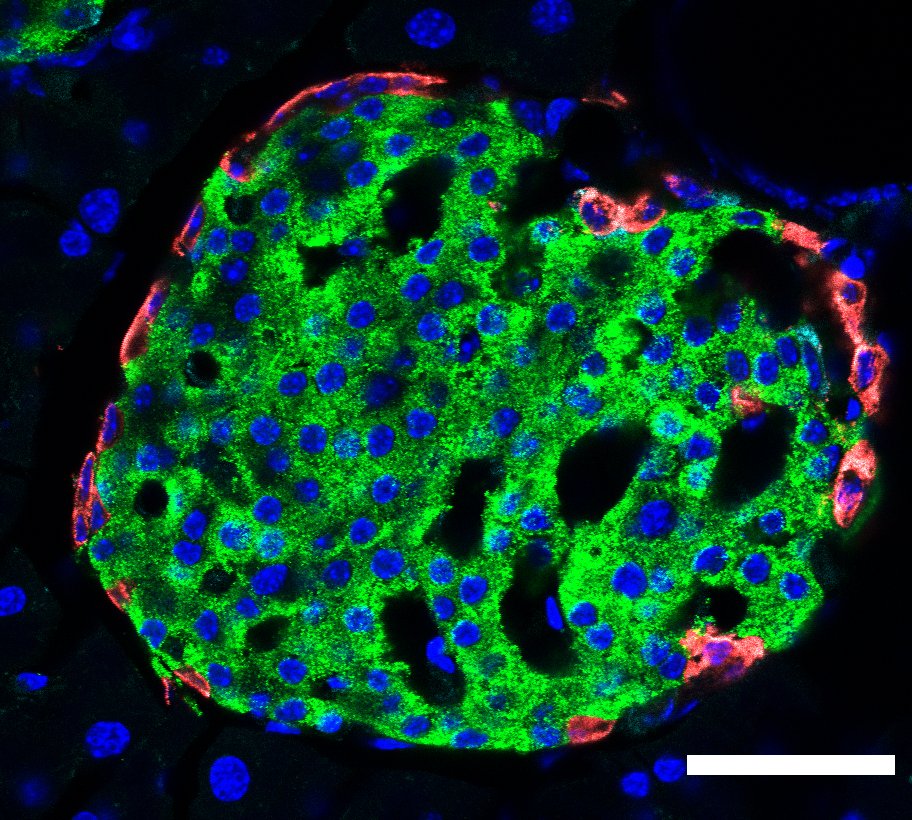

Supplement: Supplementary file 2 — Original as well as cropped pictures of islet histology shown in Extended Data Fig. 2i. [file 42255_2022_617_MOESM2_ESM.zip › Suppl_Fig_2i-islet_histology_pictures/GLP-1RA+Tesaglitazar/Cropped/Composite dapi3 cropped.jpg]

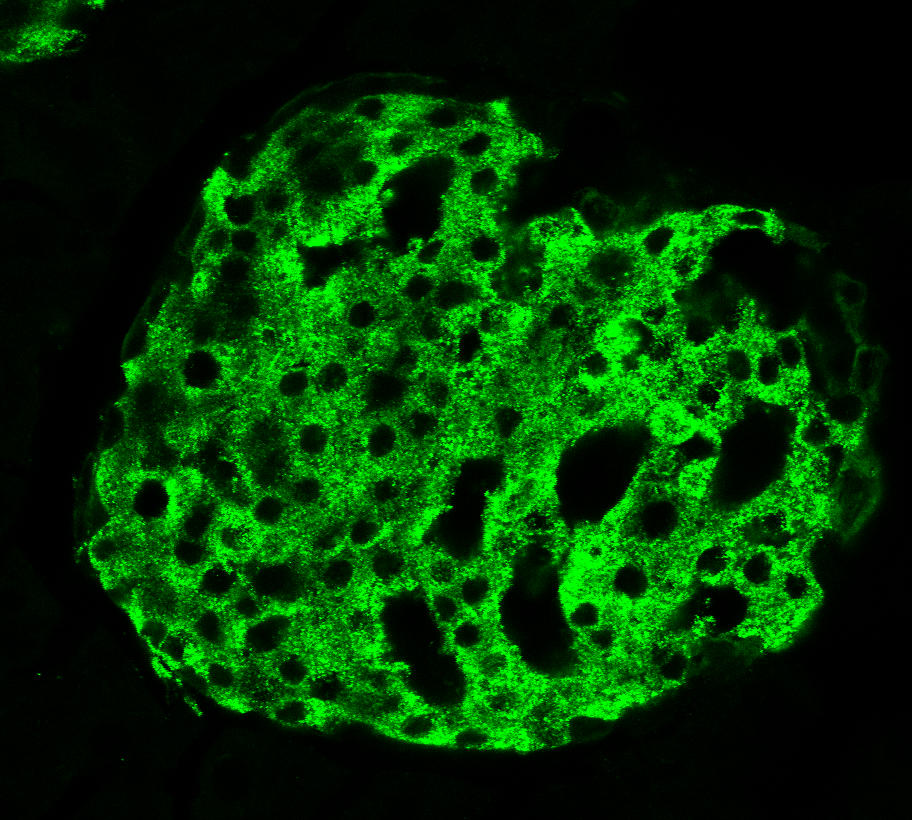

Supplement: Supplementary file 2 — Original as well as cropped pictures of islet histology shown in Extended Data Fig. 2i. [file 42255_2022_617_MOESM2_ESM.zip › Suppl_Fig_2i-islet_histology_pictures/GLP-1RA+Tesaglitazar/Cropped/ins cropped.tif]

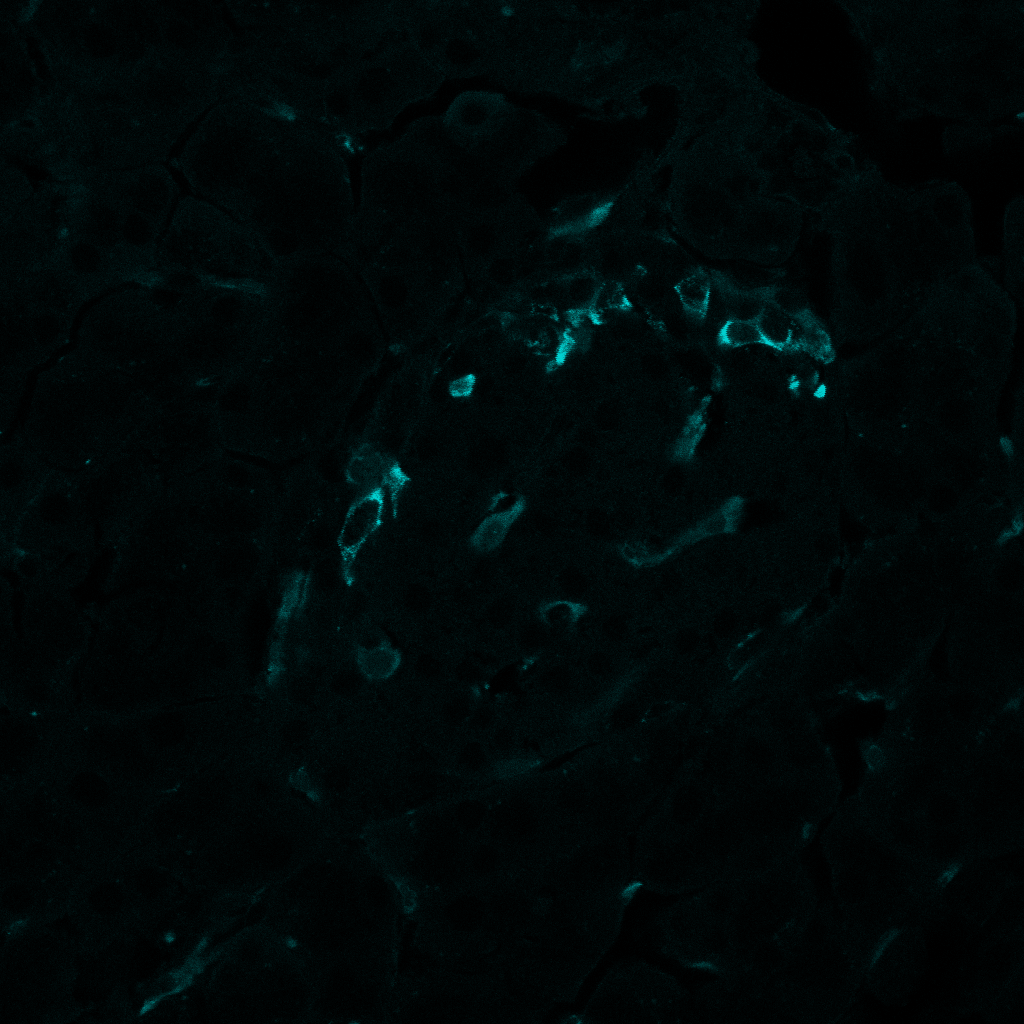

Supplement: Supplementary file 2 — Original as well as cropped pictures of islet histology shown in Extended Data Fig. 2i. [file 42255_2022_617_MOESM2_ESM.zip › Suppl_Fig_2i-islet_histology_pictures/GLP-1RA/original/sst.tif]

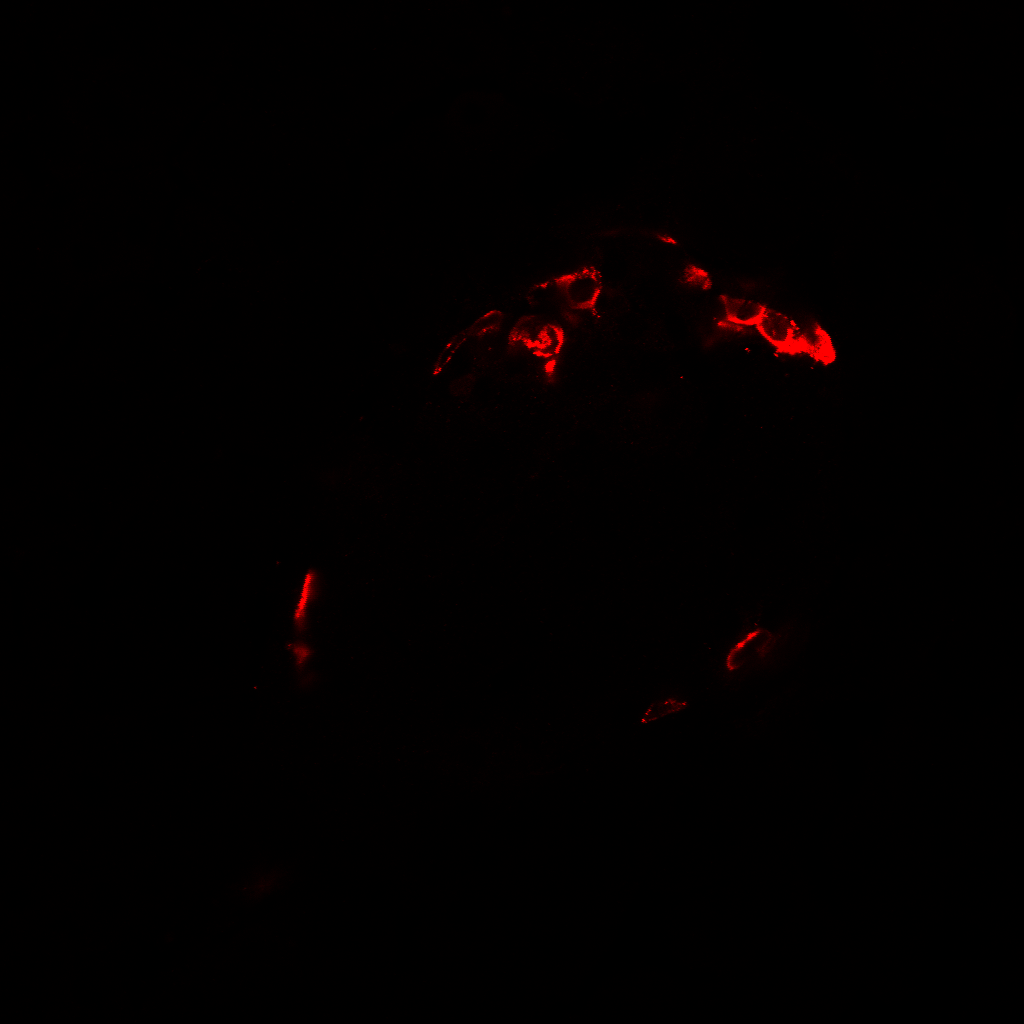

Supplement: Supplementary file 2 — Original as well as cropped pictures of islet histology shown in Extended Data Fig. 2i. [file 42255_2022_617_MOESM2_ESM.zip › Suppl_Fig_2i-islet_histology_pictures/GLP-1RA/original/gcg.tif]

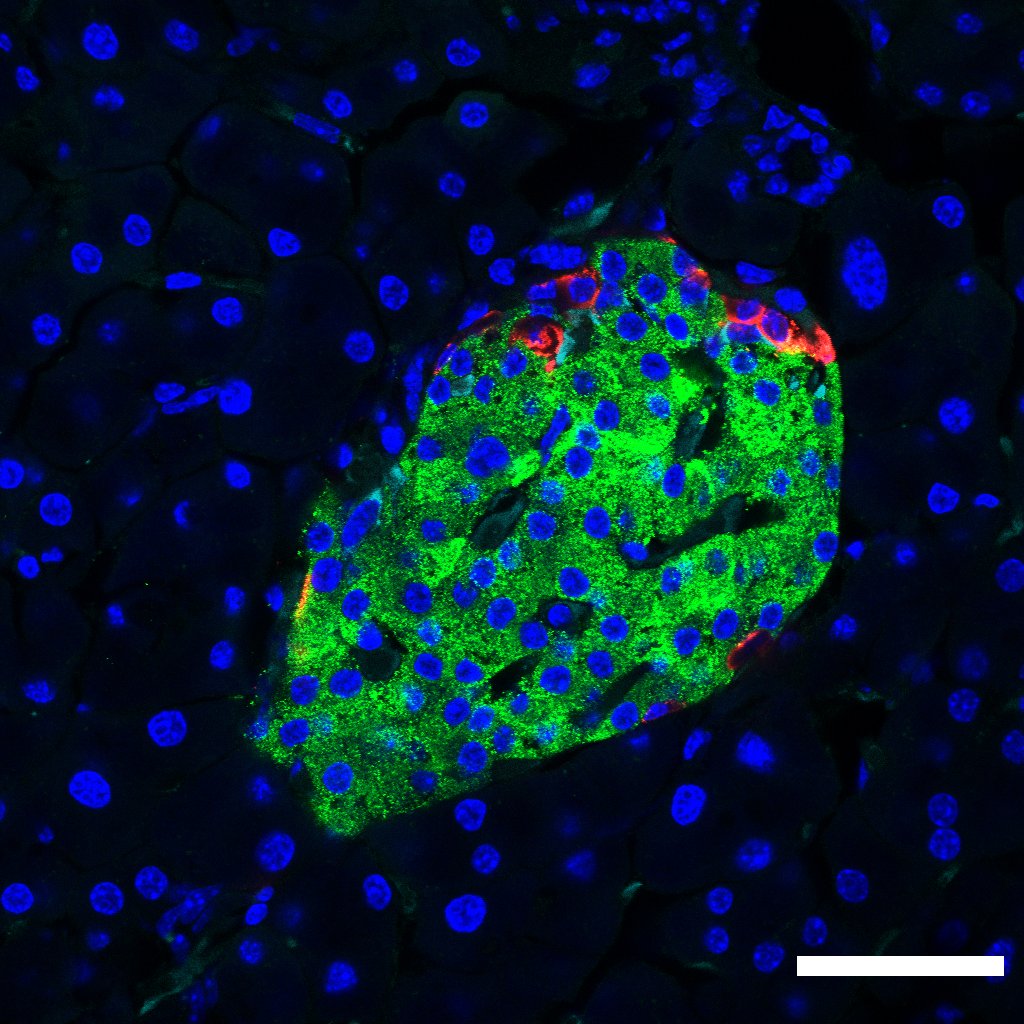

Supplement: Supplementary file 2 — Original as well as cropped pictures of islet histology shown in Extended Data Fig. 2i. [file 42255_2022_617_MOESM2_ESM.zip › Suppl_Fig_2i-islet_histology_pictures/GLP-1RA/original/Composite dapi normal.jpg]

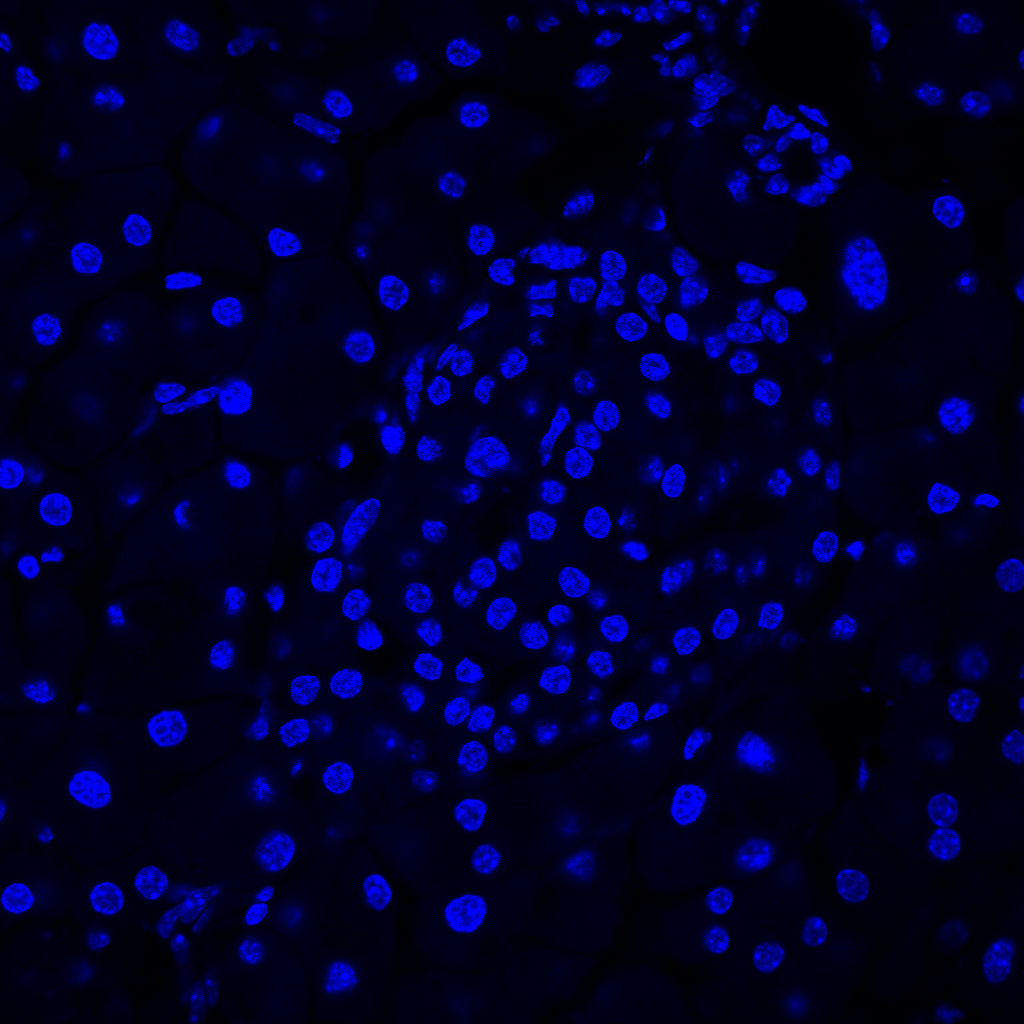

Supplement: Supplementary file 2 — Original as well as cropped pictures of islet histology shown in Extended Data Fig. 2i. [file 42255_2022_617_MOESM2_ESM.zip › Suppl_Fig_2i-islet_histology_pictures/GLP-1RA/original/dapi.tif]

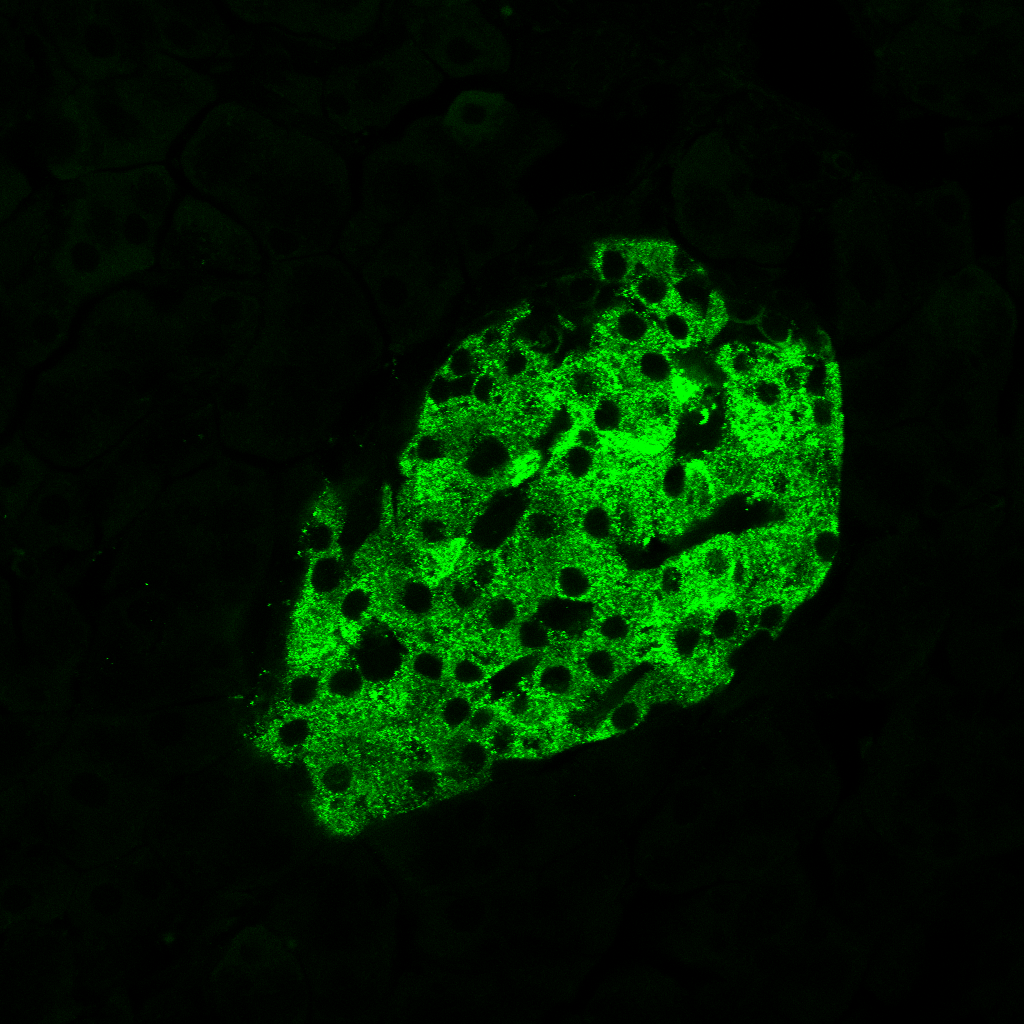

Supplement: Supplementary file 2 — Original as well as cropped pictures of islet histology shown in Extended Data Fig. 2i. [file 42255_2022_617_MOESM2_ESM.zip › Suppl_Fig_2i-islet_histology_pictures/GLP-1RA/original/ins.tif]

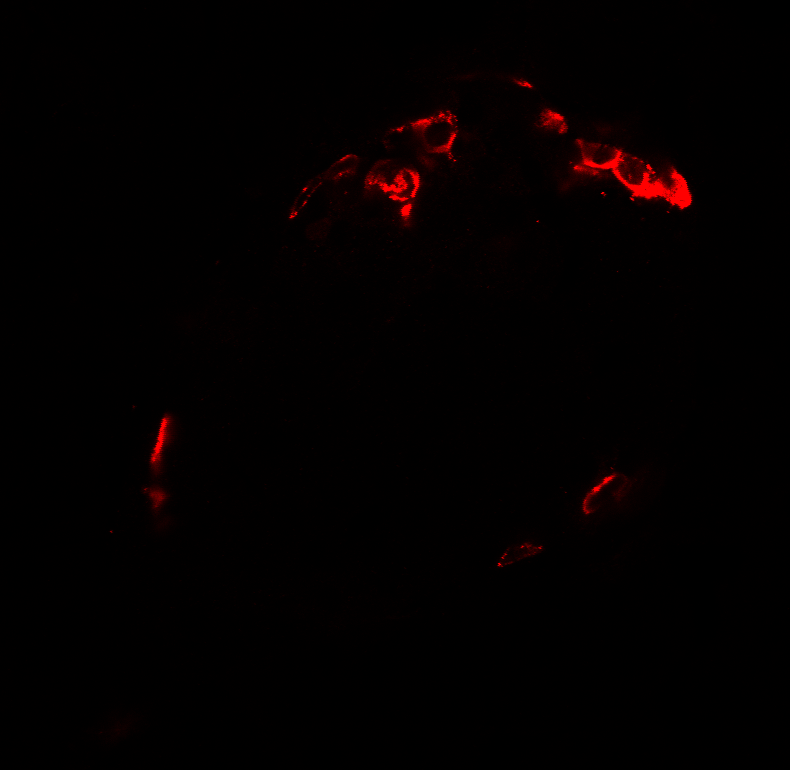

Supplement: Supplementary file 2 — Original as well as cropped pictures of islet histology shown in Extended Data Fig. 2i. [file 42255_2022_617_MOESM2_ESM.zip › Suppl_Fig_2i-islet_histology_pictures/GLP-1RA/cropped/gcg cropped.tif]

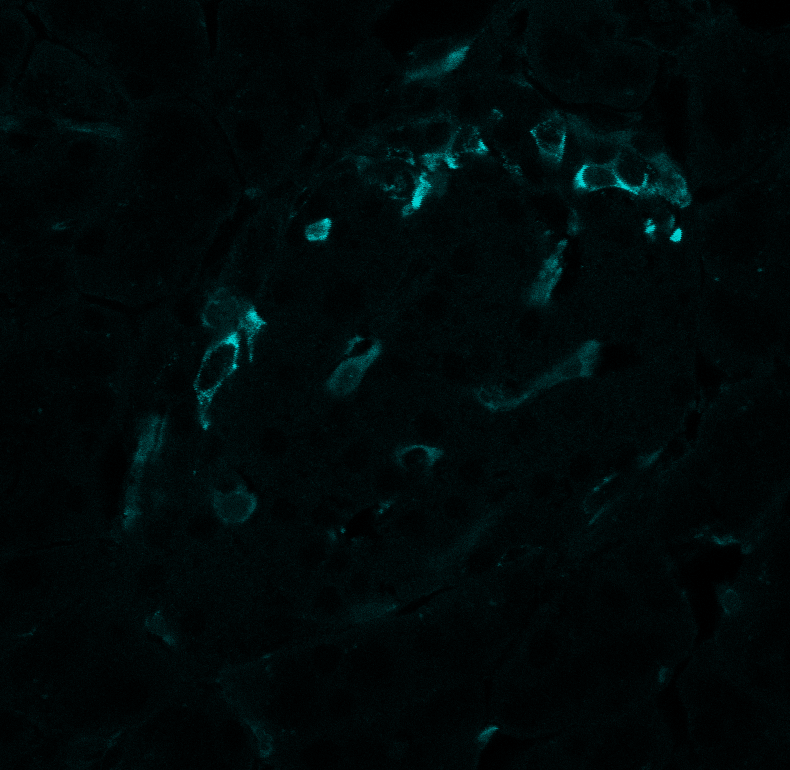

Supplement: Supplementary file 2 — Original as well as cropped pictures of islet histology shown in Extended Data Fig. 2i. [file 42255_2022_617_MOESM2_ESM.zip › Suppl_Fig_2i-islet_histology_pictures/GLP-1RA/cropped/sst cropped.tif]

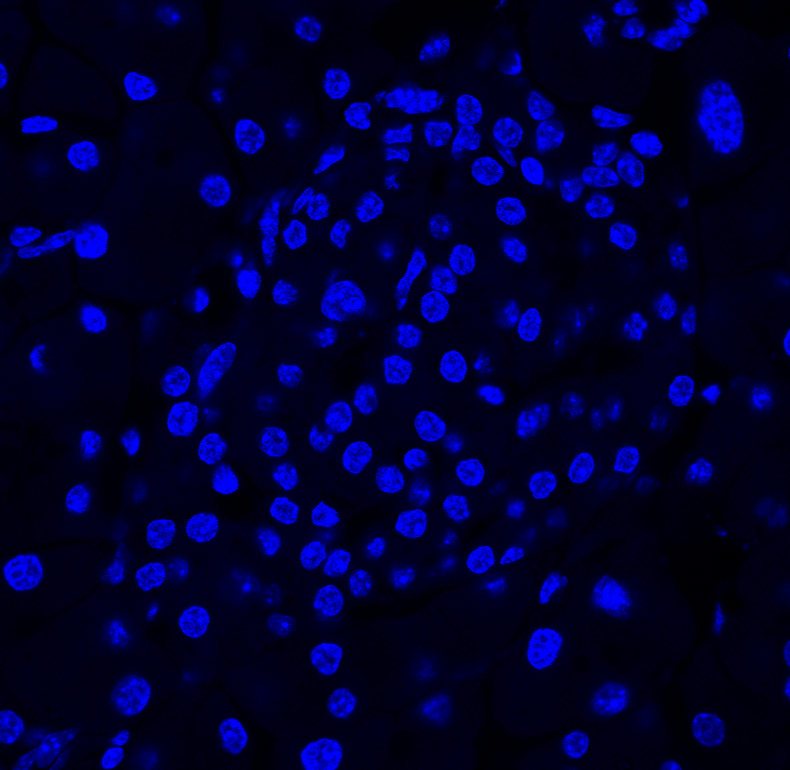

Supplement: Supplementary file 2 — Original as well as cropped pictures of islet histology shown in Extended Data Fig. 2i. [file 42255_2022_617_MOESM2_ESM.zip › Suppl_Fig_2i-islet_histology_pictures/GLP-1RA/cropped/dapi cropped.tif]

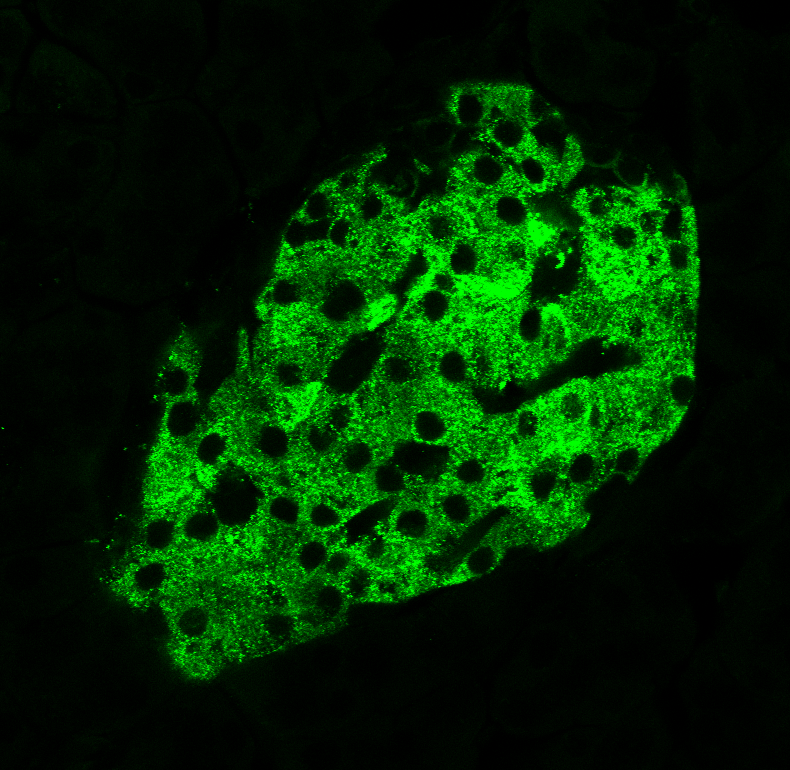

Supplement: Supplementary file 2 — Original as well as cropped pictures of islet histology shown in Extended Data Fig. 2i. [file 42255_2022_617_MOESM2_ESM.zip › Suppl_Fig_2i-islet_histology_pictures/GLP-1RA/cropped/ins cropped.tif]

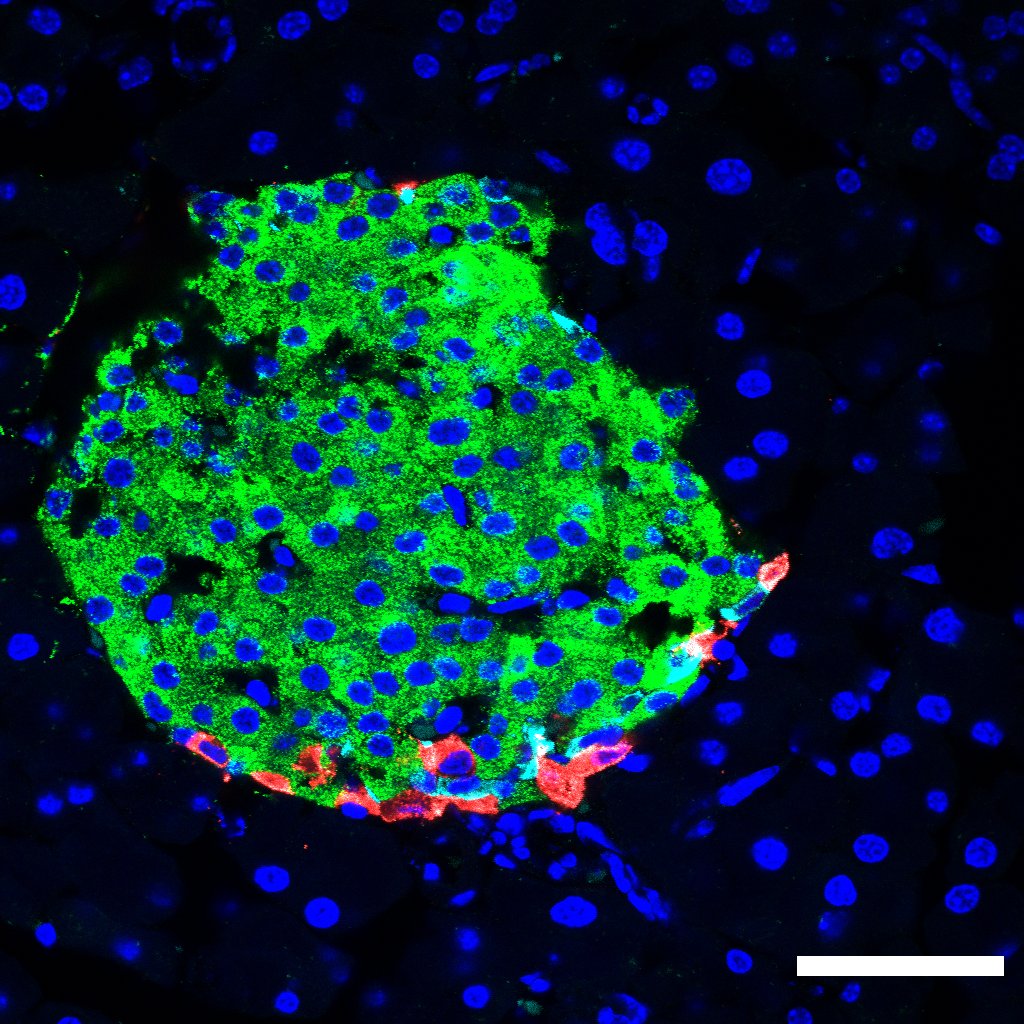

Supplement: Supplementary file 2 — Original as well as cropped pictures of islet histology shown in Extended Data Fig. 2i. [file 42255_2022_617_MOESM2_ESM.zip › Suppl_Fig_2i-islet_histology_pictures/Tesaglitazar/original/Composite dapi3.jpg]

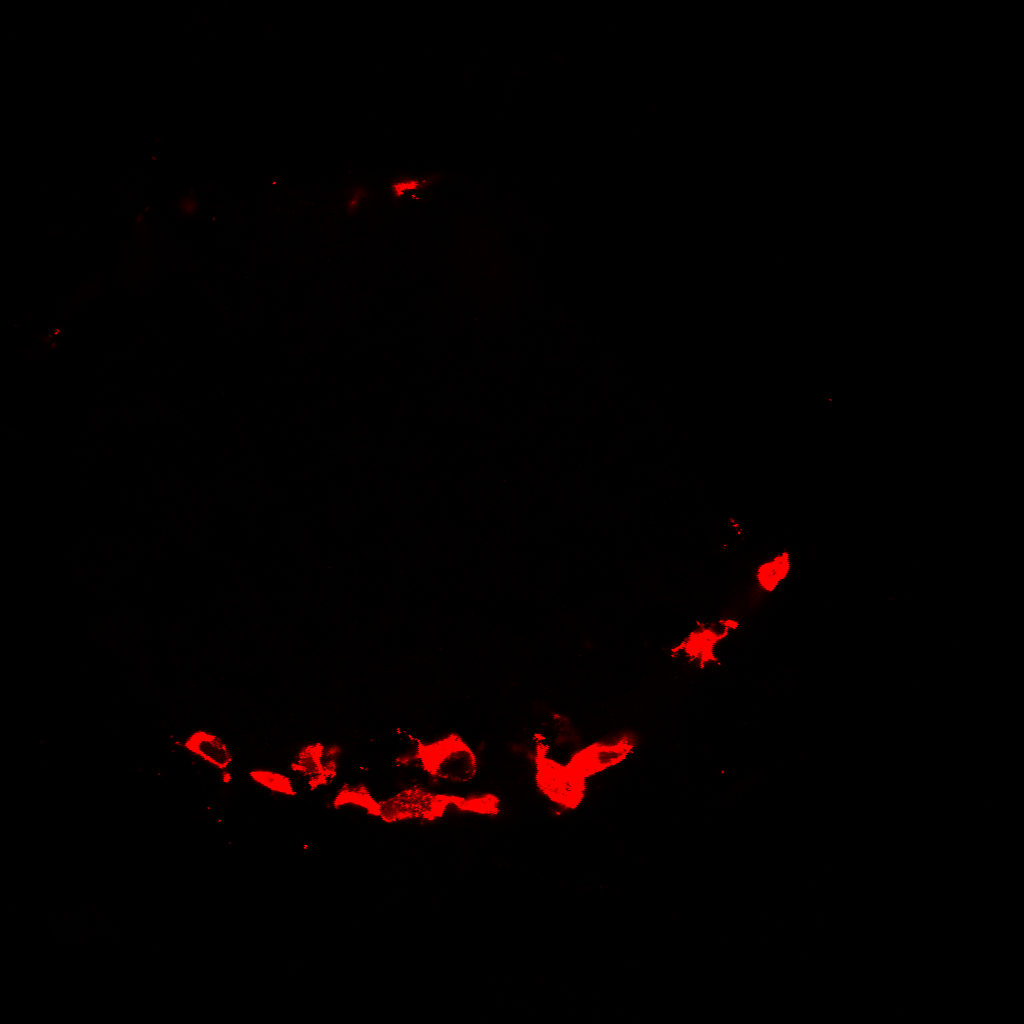

Supplement: Supplementary file 2 — Original as well as cropped pictures of islet histology shown in Extended Data Fig. 2i. [file 42255_2022_617_MOESM2_ESM.zip › Suppl_Fig_2i-islet_histology_pictures/Tesaglitazar/original/gcg.tif]

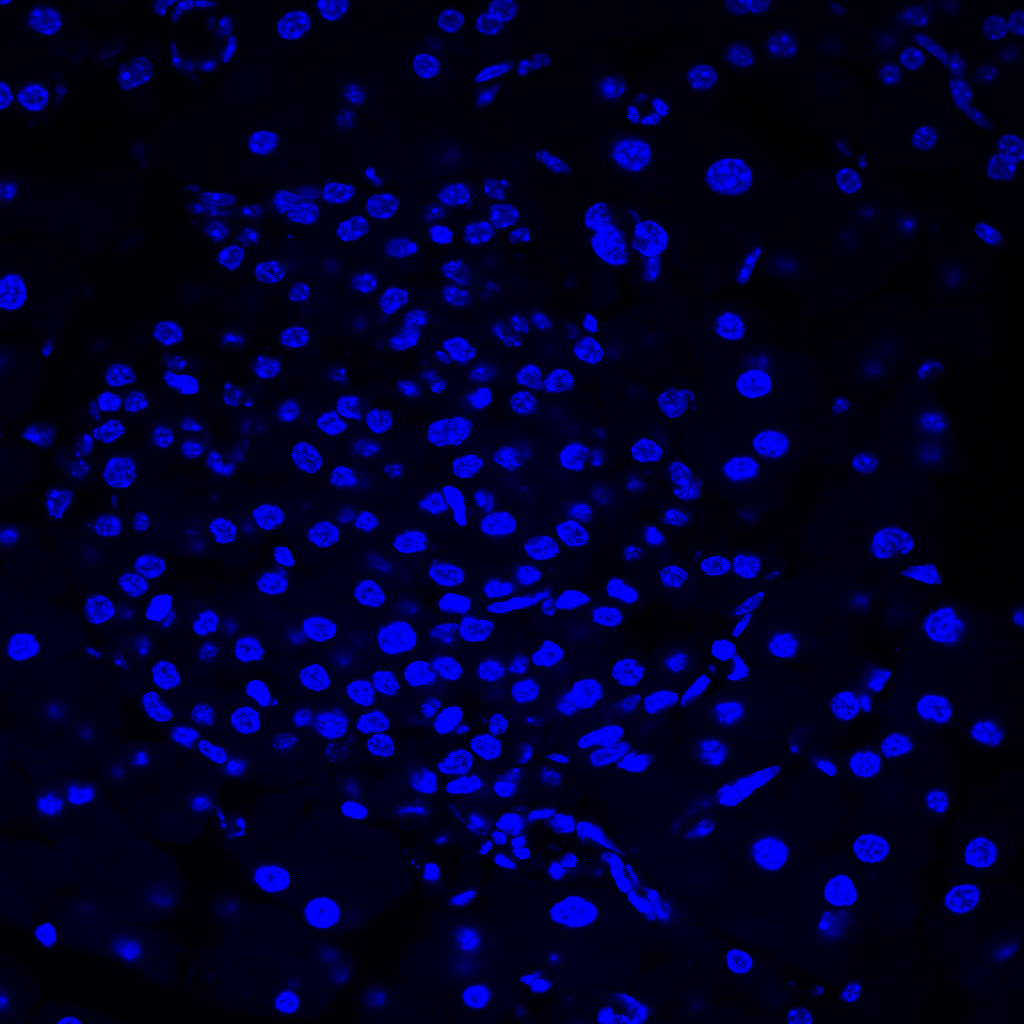

Supplement: Supplementary file 2 — Original as well as cropped pictures of islet histology shown in Extended Data Fig. 2i. [file 42255_2022_617_MOESM2_ESM.zip › Suppl_Fig_2i-islet_histology_pictures/Tesaglitazar/original/dapi.tif]

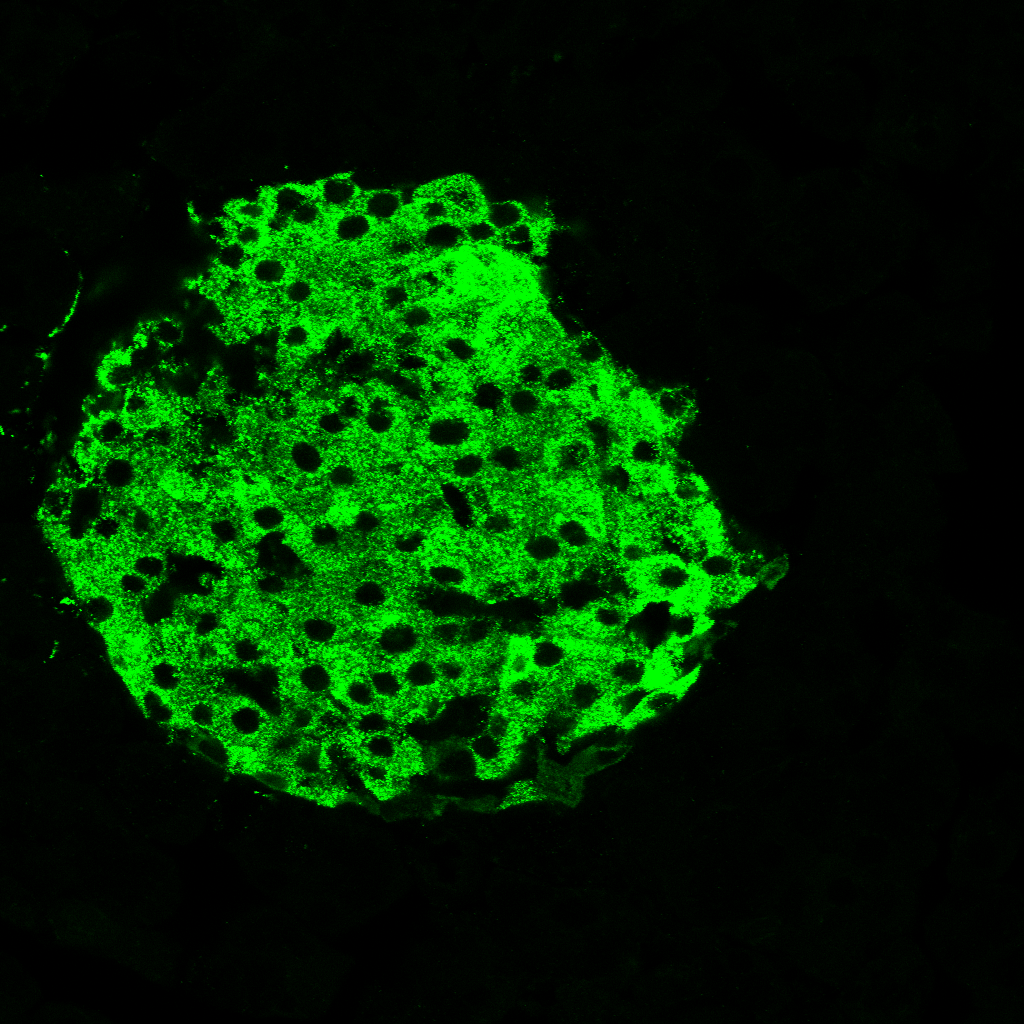

Supplement: Supplementary file 2 — Original as well as cropped pictures of islet histology shown in Extended Data Fig. 2i. [file 42255_2022_617_MOESM2_ESM.zip › Suppl_Fig_2i-islet_histology_pictures/Tesaglitazar/original/ins.tif]

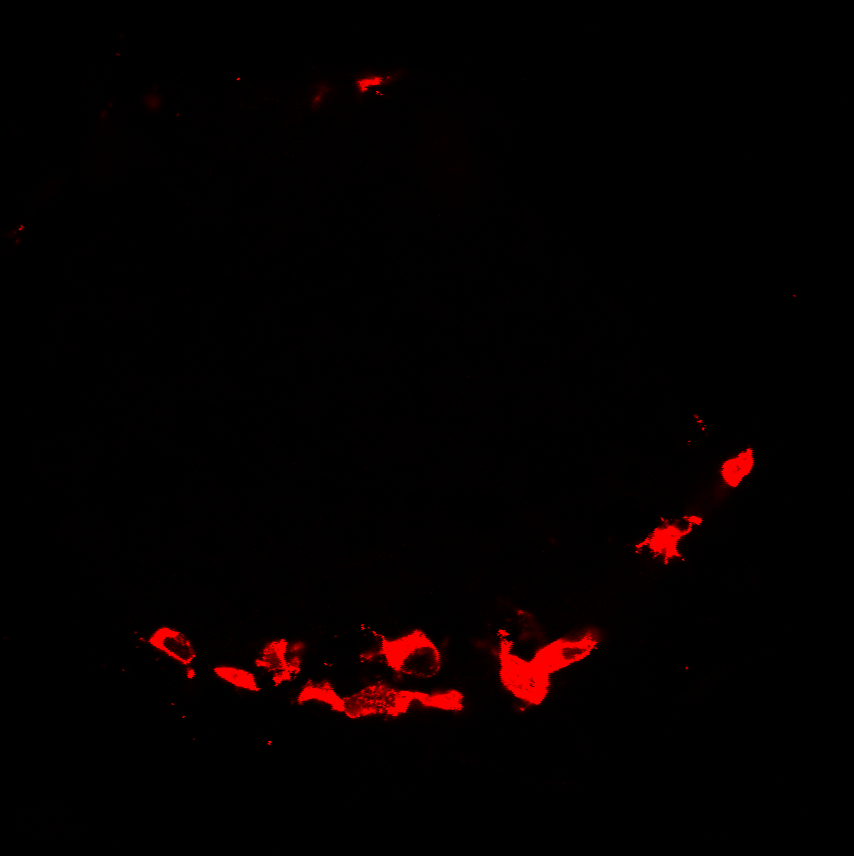

Supplement: Supplementary file 2 — Original as well as cropped pictures of islet histology shown in Extended Data Fig. 2i. [file 42255_2022_617_MOESM2_ESM.zip › Suppl_Fig_2i-islet_histology_pictures/Tesaglitazar/cropped/gcg cropped.tif]

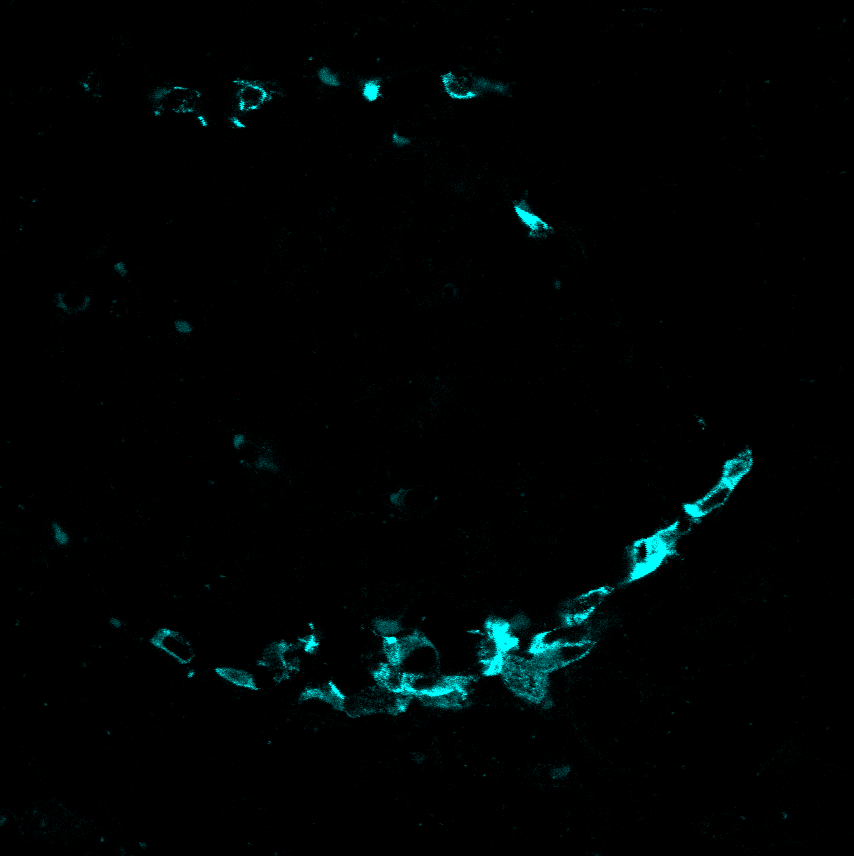

Supplement: Supplementary file 2 — Original as well as cropped pictures of islet histology shown in Extended Data Fig. 2i. [file 42255_2022_617_MOESM2_ESM.zip › Suppl_Fig_2i-islet_histology_pictures/Tesaglitazar/cropped/sst cropped.tif]

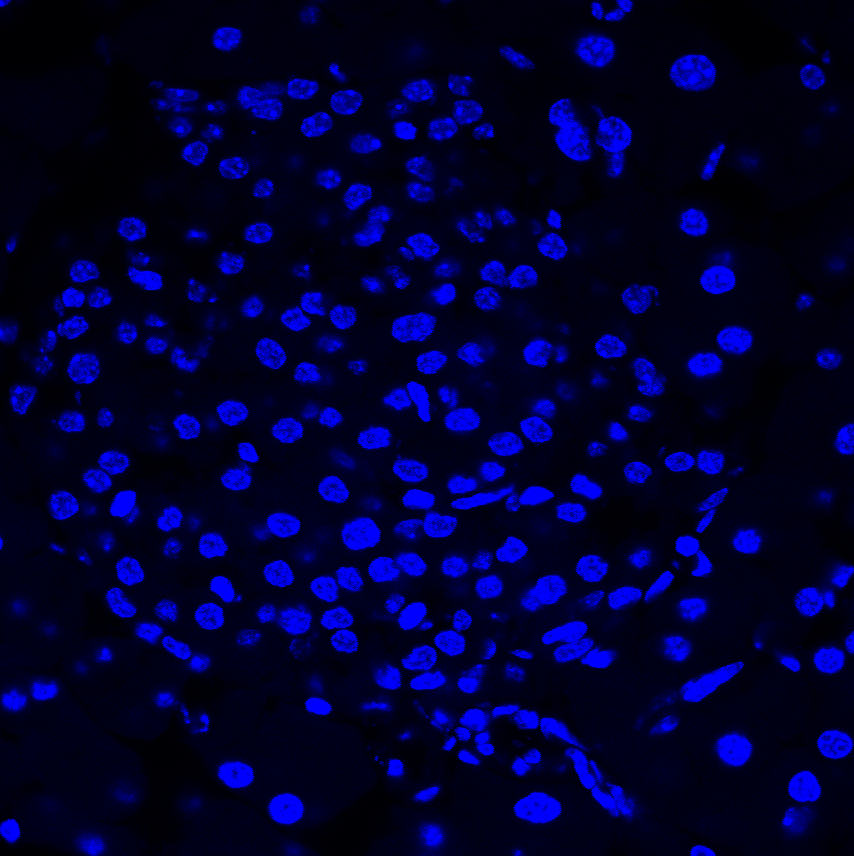

Supplement: Supplementary file 2 — Original as well as cropped pictures of islet histology shown in Extended Data Fig. 2i. [file 42255_2022_617_MOESM2_ESM.zip › Suppl_Fig_2i-islet_histology_pictures/Tesaglitazar/cropped/dapi cropped.tif]

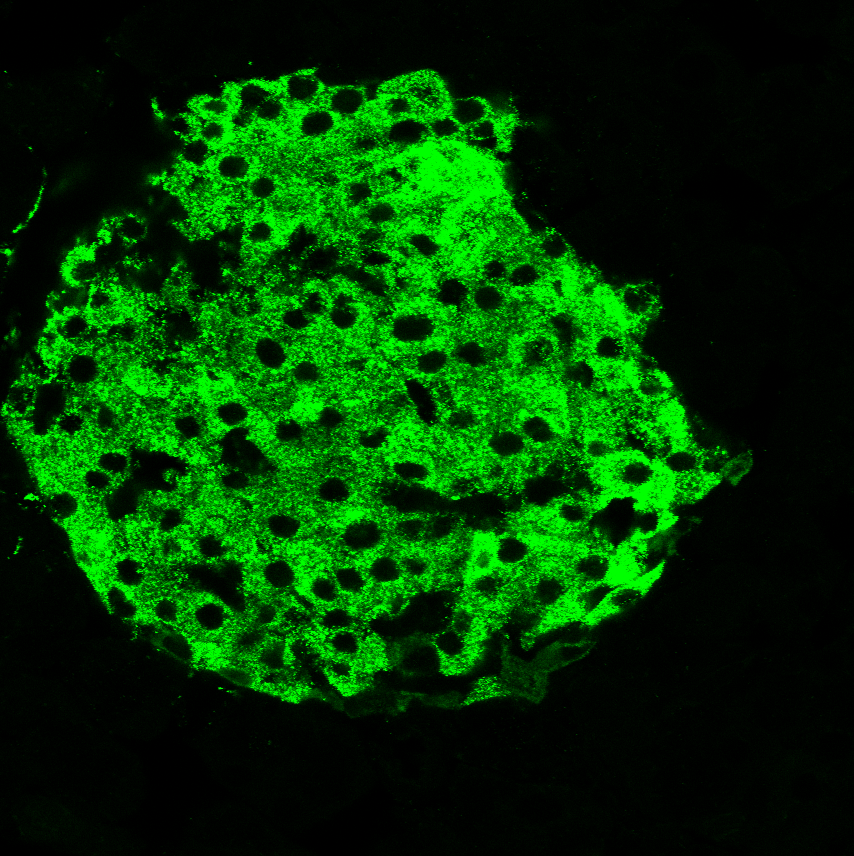

Supplement: Supplementary file 2 — Original as well as cropped pictures of islet histology shown in Extended Data Fig. 2i. [file 42255_2022_617_MOESM2_ESM.zip › Suppl_Fig_2i-islet_histology_pictures/Tesaglitazar/cropped/cropped ins.tif]

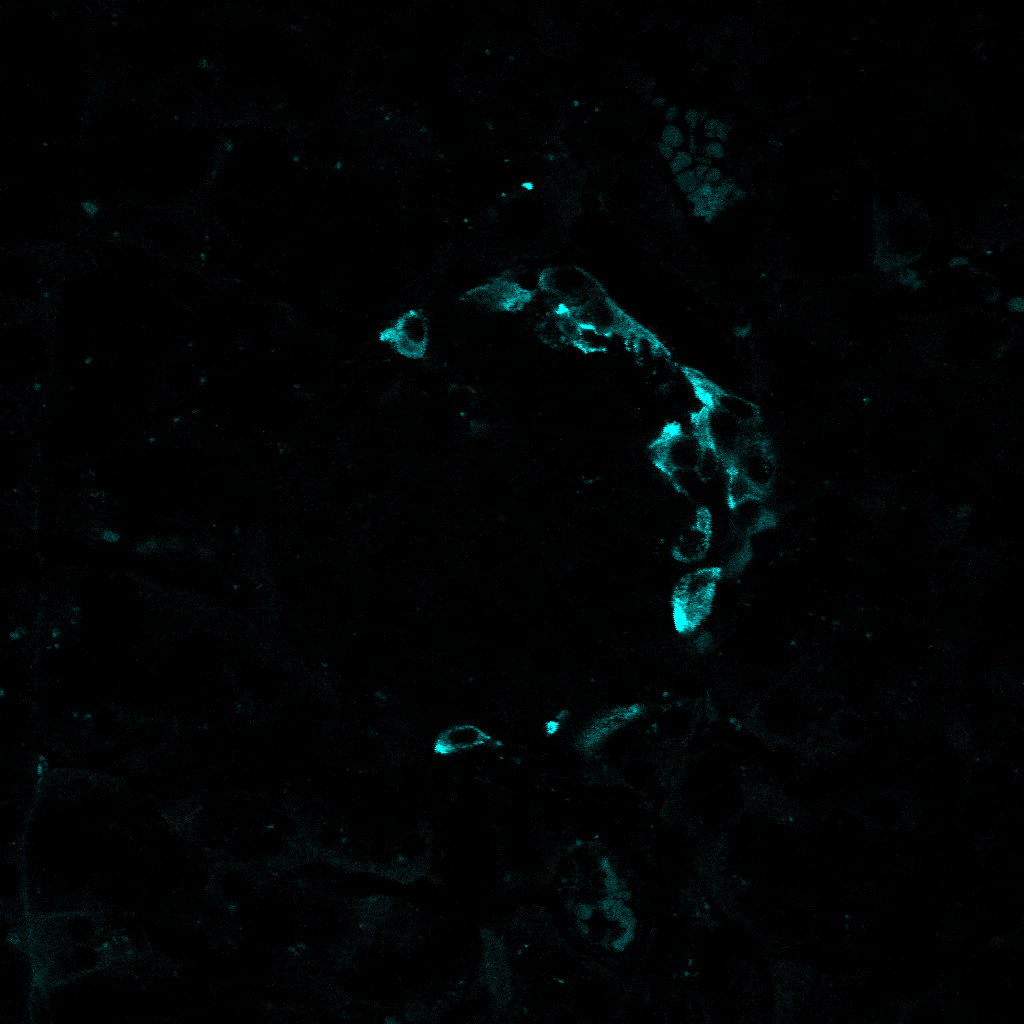

Supplement: Supplementary file 2 — Original as well as cropped pictures of islet histology shown in Extended Data Fig. 2i. [file 42255_2022_617_MOESM2_ESM.zip › Suppl_Fig_2i-islet_histology_pictures/GLP-1RA:Tesaglitazar/Original/sst.tif]

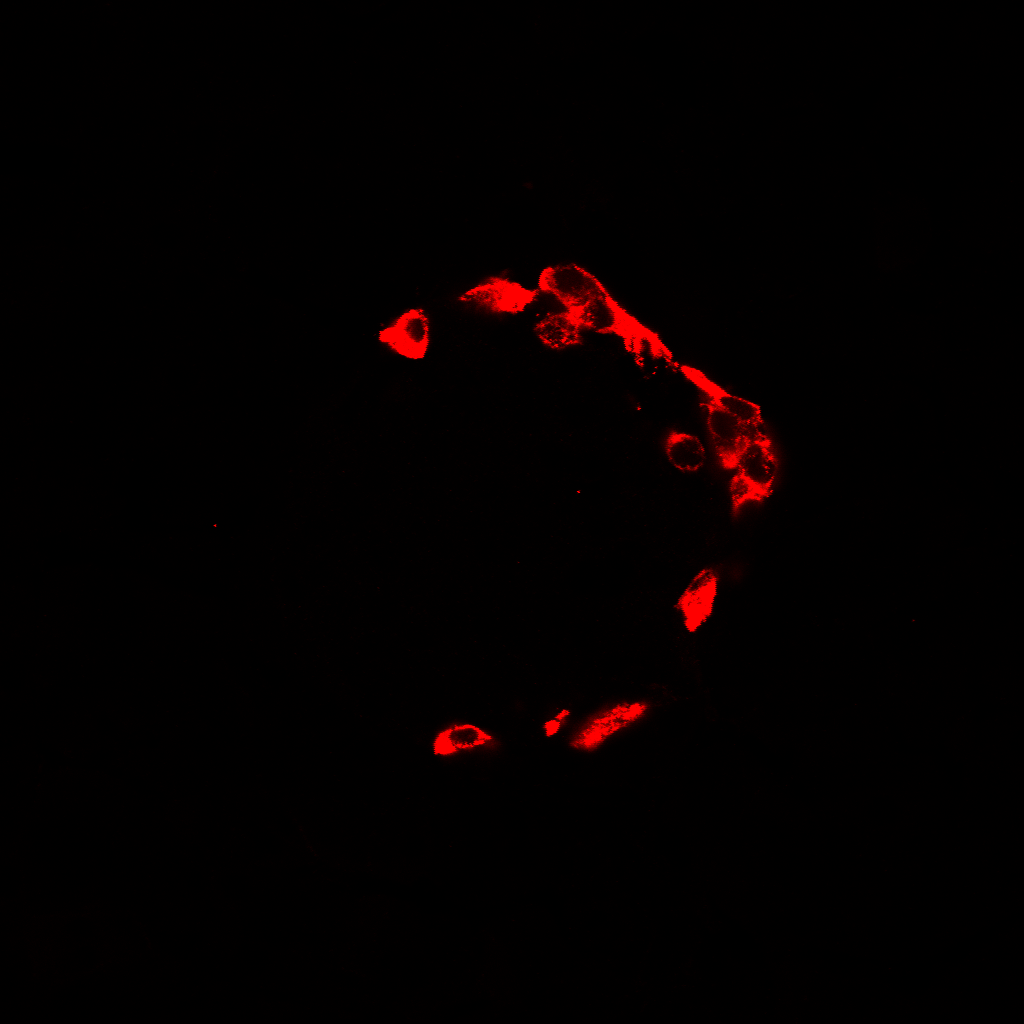

Supplement: Supplementary file 2 — Original as well as cropped pictures of islet histology shown in Extended Data Fig. 2i. [file 42255_2022_617_MOESM2_ESM.zip › Suppl_Fig_2i-islet_histology_pictures/GLP-1RA:Tesaglitazar/Original/gcg.tif]

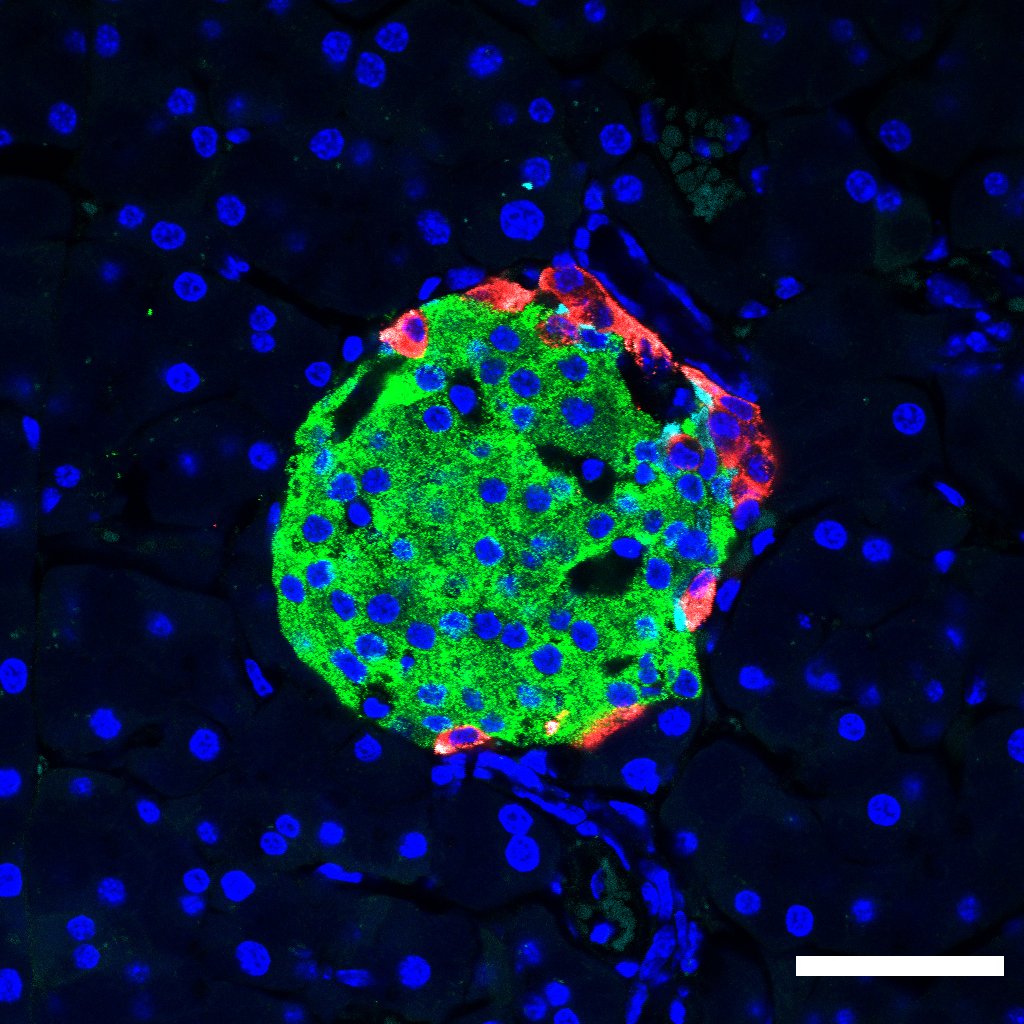

Supplement: Supplementary file 2 — Original as well as cropped pictures of islet histology shown in Extended Data Fig. 2i. [file 42255_2022_617_MOESM2_ESM.zip › Suppl_Fig_2i-islet_histology_pictures/GLP-1RA:Tesaglitazar/Original/Compositedapi3.jpg]

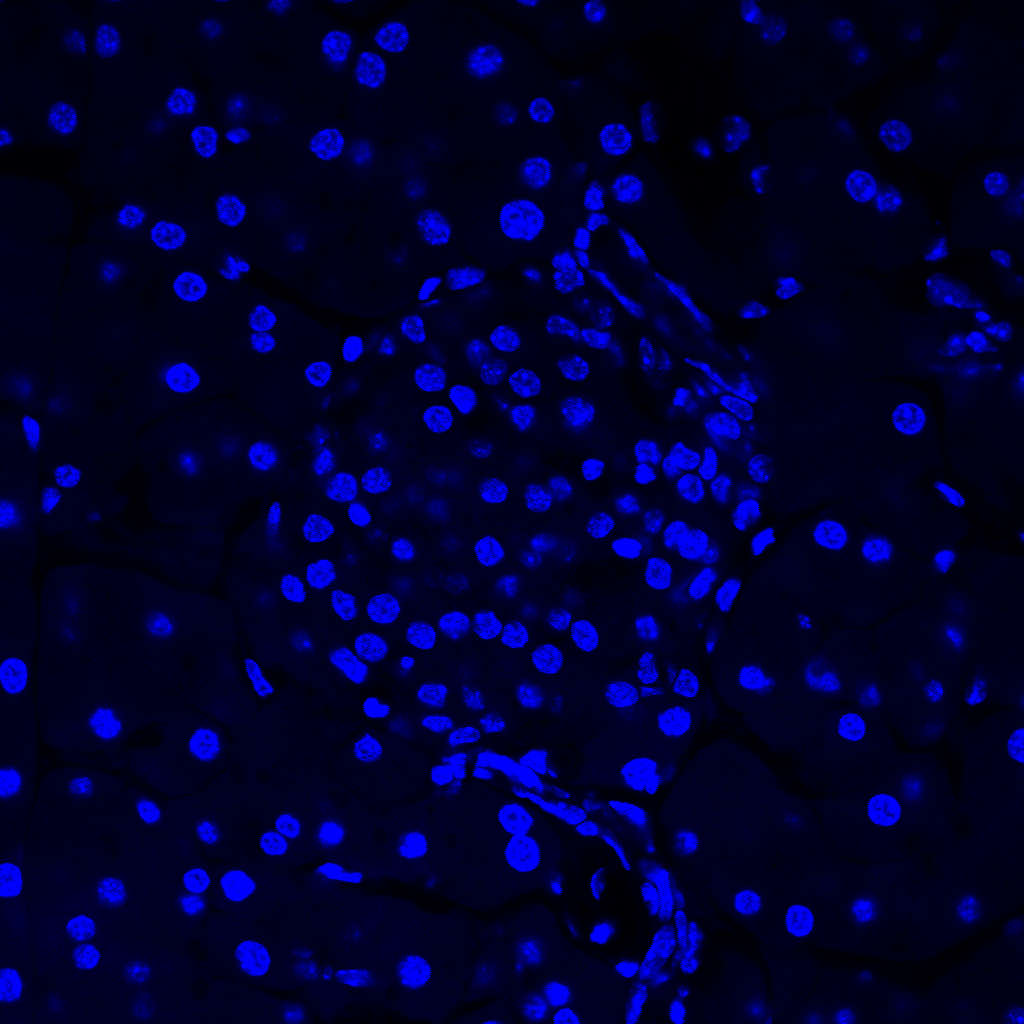

Supplement: Supplementary file 2 — Original as well as cropped pictures of islet histology shown in Extended Data Fig. 2i. [file 42255_2022_617_MOESM2_ESM.zip › Suppl_Fig_2i-islet_histology_pictures/GLP-1RA:Tesaglitazar/Original/dapi.tif]

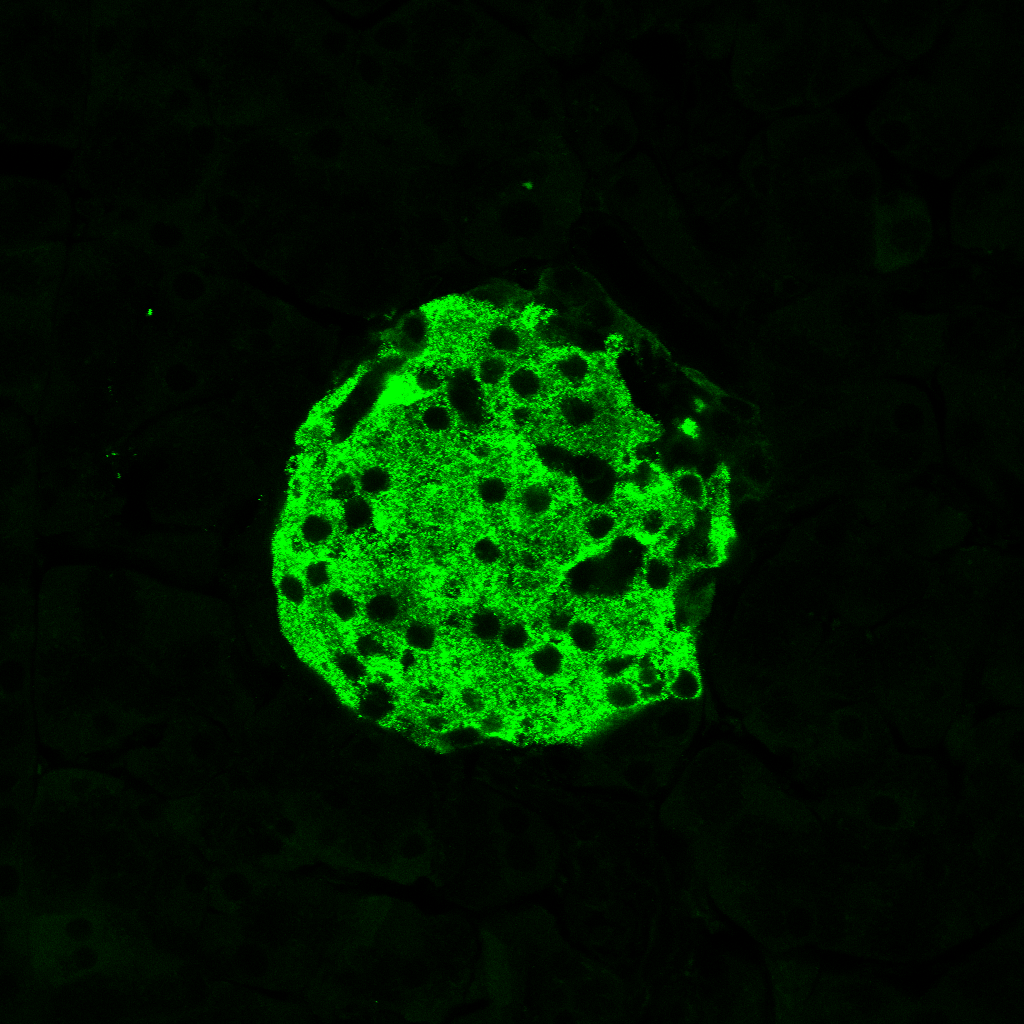

Supplement: Supplementary file 2 — Original as well as cropped pictures of islet histology shown in Extended Data Fig. 2i. [file 42255_2022_617_MOESM2_ESM.zip › Suppl_Fig_2i-islet_histology_pictures/GLP-1RA:Tesaglitazar/Original/ins.tif]

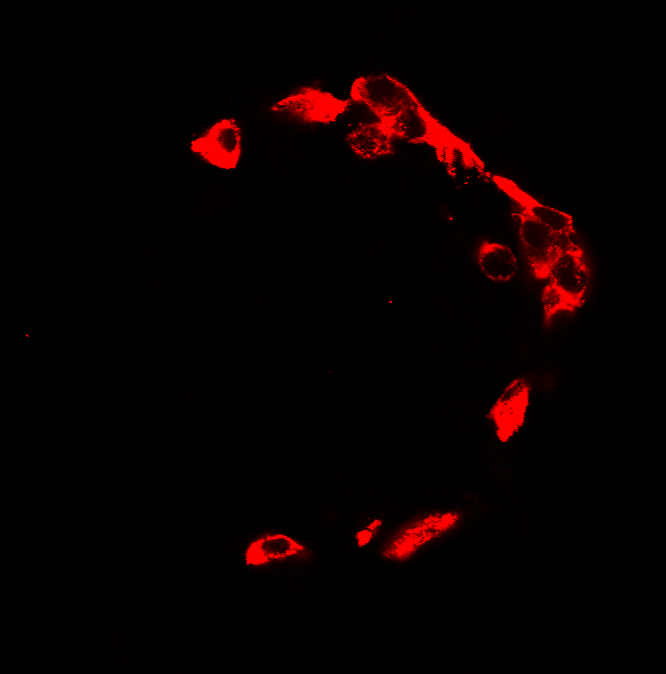

Supplement: Supplementary file 2 — Original as well as cropped pictures of islet histology shown in Extended Data Fig. 2i. [file 42255_2022_617_MOESM2_ESM.zip › Suppl_Fig_2i-islet_histology_pictures/GLP-1RA:Tesaglitazar/Cropped/gcg cropped.tif]

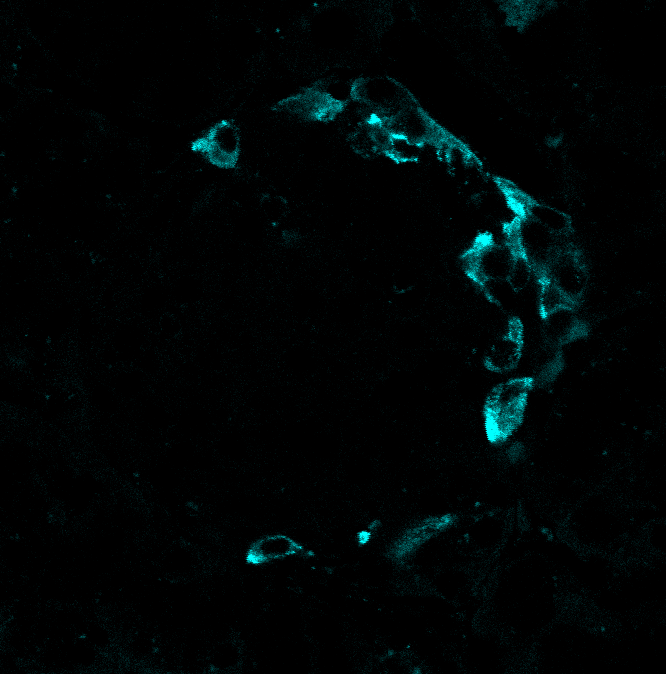

Supplement: Supplementary file 2 — Original as well as cropped pictures of islet histology shown in Extended Data Fig. 2i. [file 42255_2022_617_MOESM2_ESM.zip › Suppl_Fig_2i-islet_histology_pictures/GLP-1RA:Tesaglitazar/Cropped/sst cropped.tif]

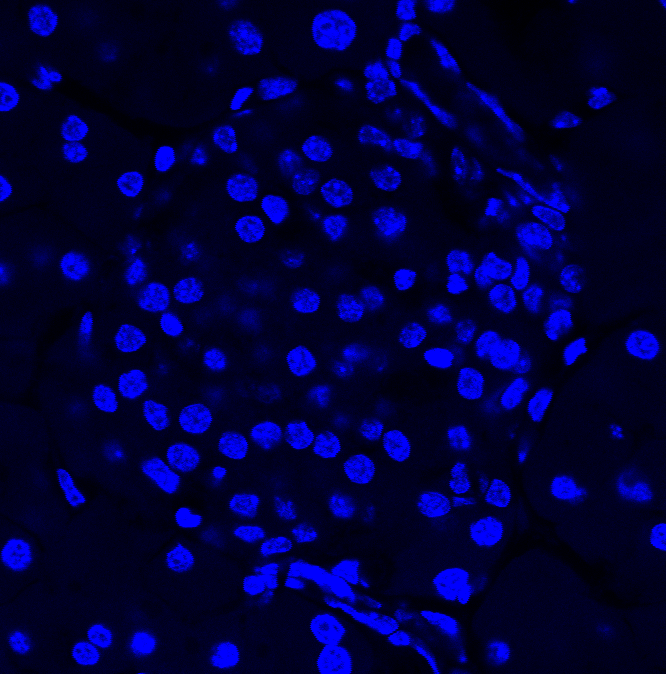

Supplement: Supplementary file 2 — Original as well as cropped pictures of islet histology shown in Extended Data Fig. 2i. [file 42255_2022_617_MOESM2_ESM.zip › Suppl_Fig_2i-islet_histology_pictures/GLP-1RA:Tesaglitazar/Cropped/dapi cropped.tif]

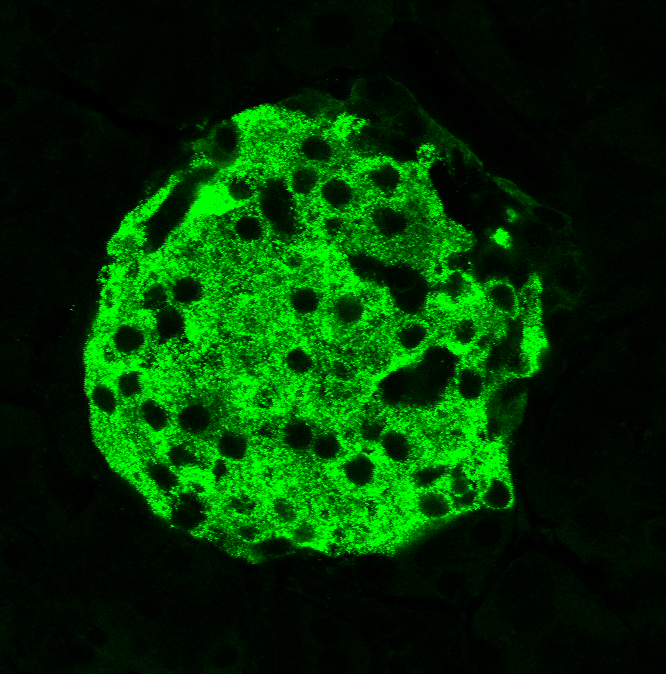

Supplement: Supplementary file 2 — Original as well as cropped pictures of islet histology shown in Extended Data Fig. 2i. [file 42255_2022_617_MOESM2_ESM.zip › Suppl_Fig_2i-islet_histology_pictures/GLP-1RA:Tesaglitazar/Cropped/ins cropped.tif]

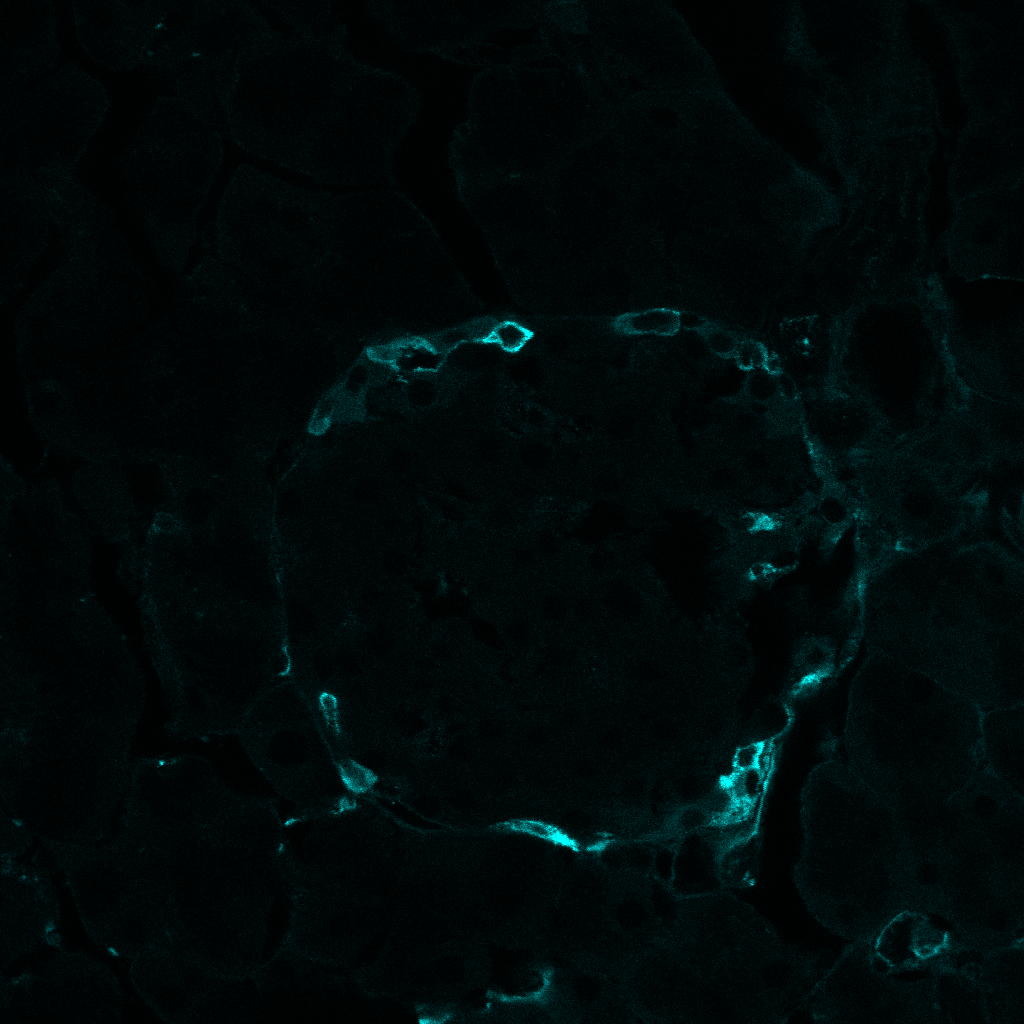

Supplement: Supplementary file 2 — Original as well as cropped pictures of islet histology shown in Extended Data Fig. 2i. [file 42255_2022_617_MOESM2_ESM.zip › Suppl_Fig_2i-islet_histology_pictures/Vhcl/Original/sst.tif]

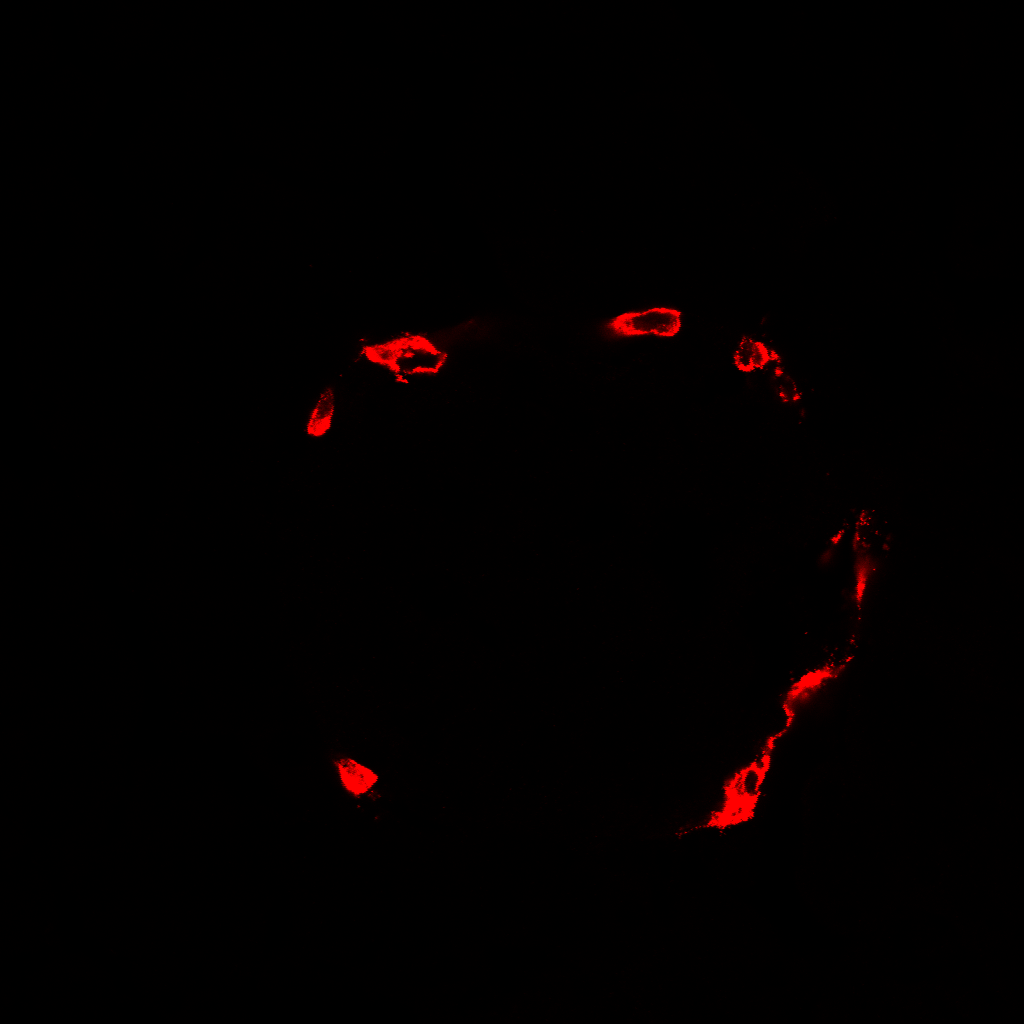

Supplement: Supplementary file 2 — Original as well as cropped pictures of islet histology shown in Extended Data Fig. 2i. [file 42255_2022_617_MOESM2_ESM.zip › Suppl_Fig_2i-islet_histology_pictures/Vhcl/Original/gcg.tif]

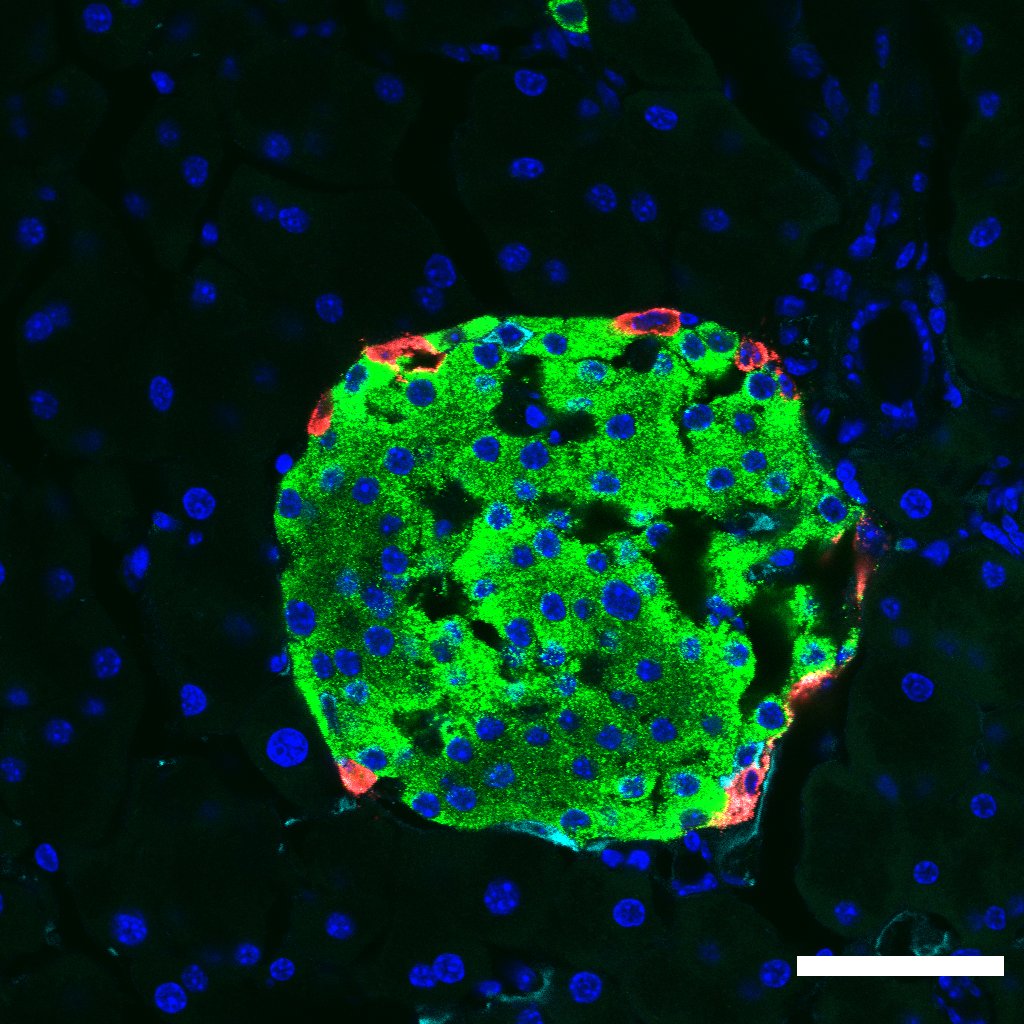

Supplement: Supplementary file 2 — Original as well as cropped pictures of islet histology shown in Extended Data Fig. 2i. [file 42255_2022_617_MOESM2_ESM.zip › Suppl_Fig_2i-islet_histology_pictures/Vhcl/Original/Compositedapi3.jpg]

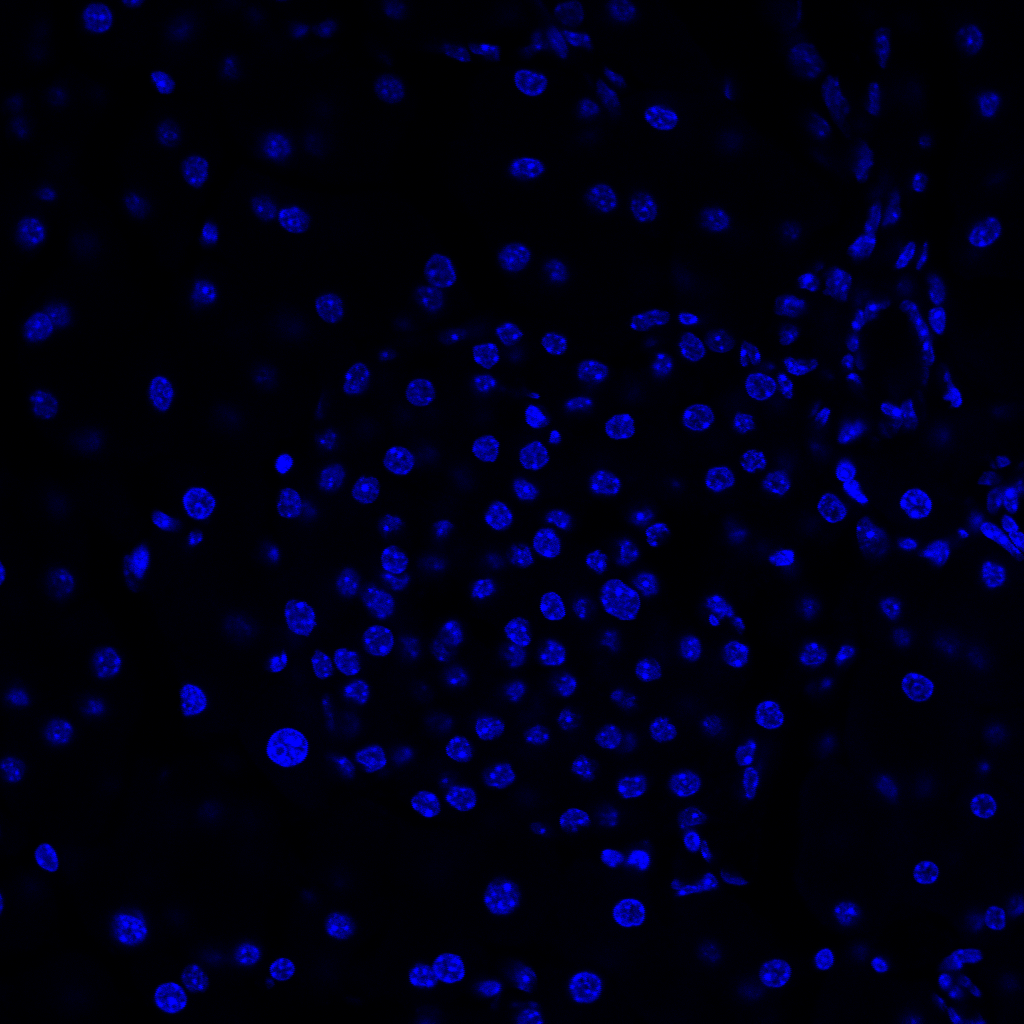

Supplement: Supplementary file 2 — Original as well as cropped pictures of islet histology shown in Extended Data Fig. 2i. [file 42255_2022_617_MOESM2_ESM.zip › Suppl_Fig_2i-islet_histology_pictures/Vhcl/Original/dapi.tif]

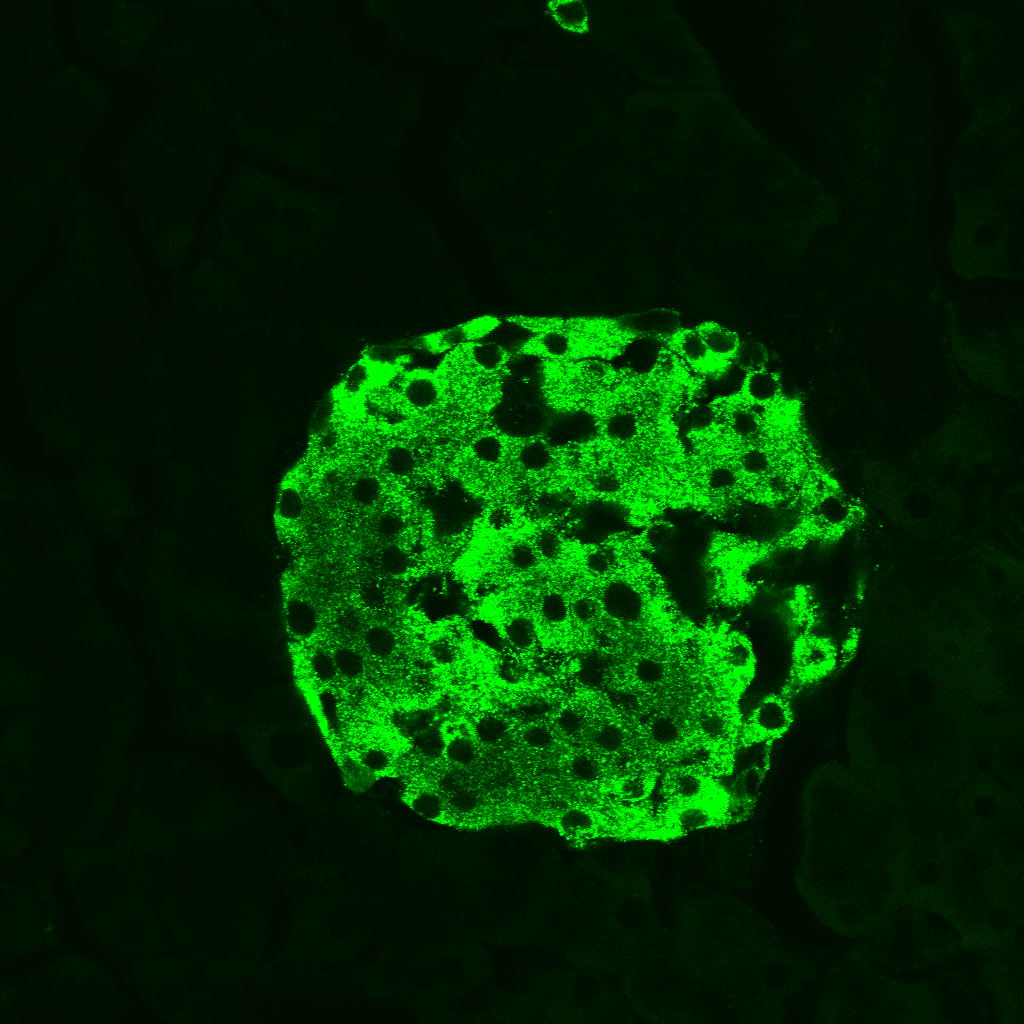

Supplement: Supplementary file 2 — Original as well as cropped pictures of islet histology shown in Extended Data Fig. 2i. [file 42255_2022_617_MOESM2_ESM.zip › Suppl_Fig_2i-islet_histology_pictures/Vhcl/Original/ins.tif]

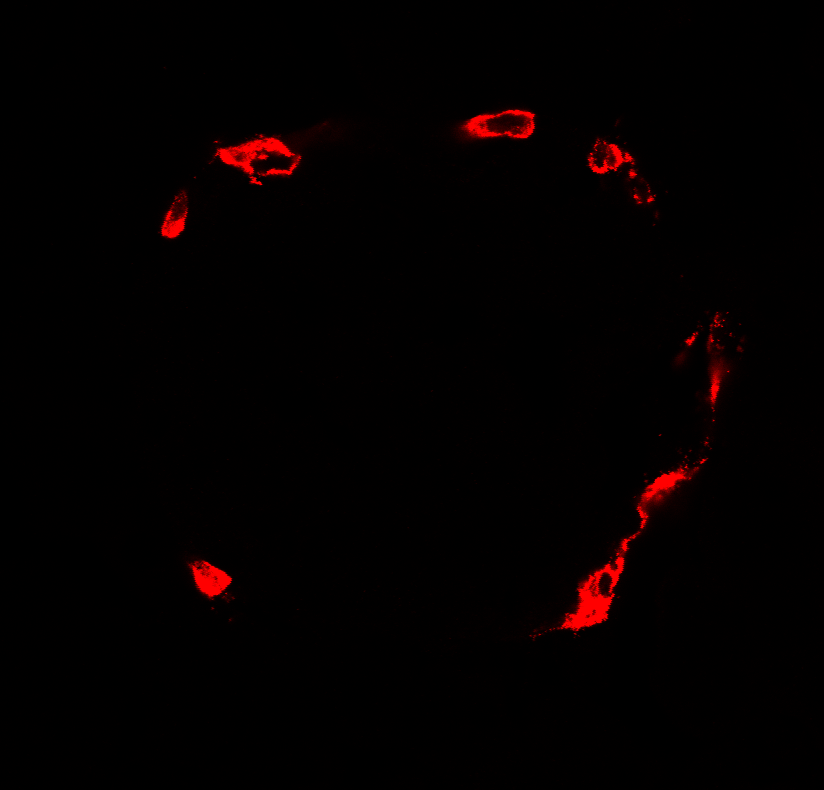

Supplement: Supplementary file 2 — Original as well as cropped pictures of islet histology shown in Extended Data Fig. 2i. [file 42255_2022_617_MOESM2_ESM.zip › Suppl_Fig_2i-islet_histology_pictures/Vhcl/Cropped/gcg cropped.tif]

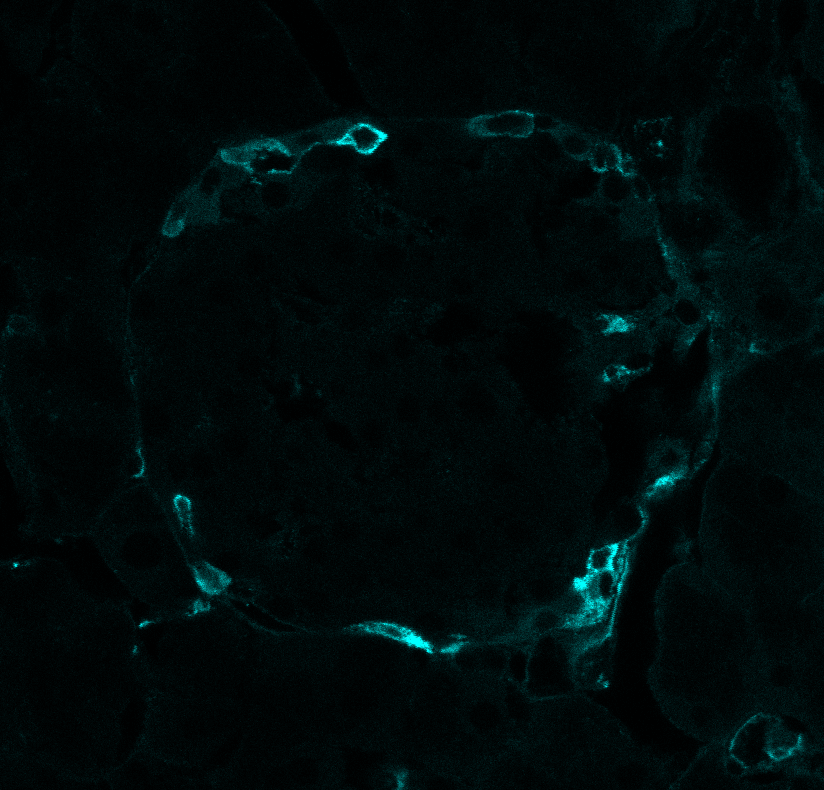

Supplement: Supplementary file 2 — Original as well as cropped pictures of islet histology shown in Extended Data Fig. 2i. [file 42255_2022_617_MOESM2_ESM.zip › Suppl_Fig_2i-islet_histology_pictures/Vhcl/Cropped/sst cropped.tif]

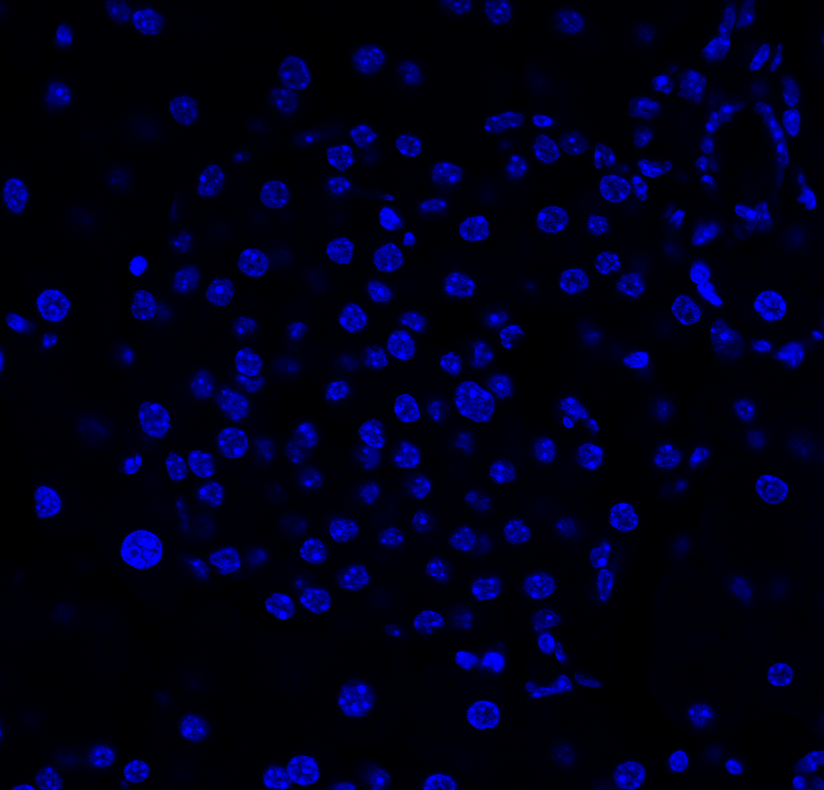

Supplement: Supplementary file 2 — Original as well as cropped pictures of islet histology shown in Extended Data Fig. 2i. [file 42255_2022_617_MOESM2_ESM.zip › Suppl_Fig_2i-islet_histology_pictures/Vhcl/Cropped/dapi cropped.tif]

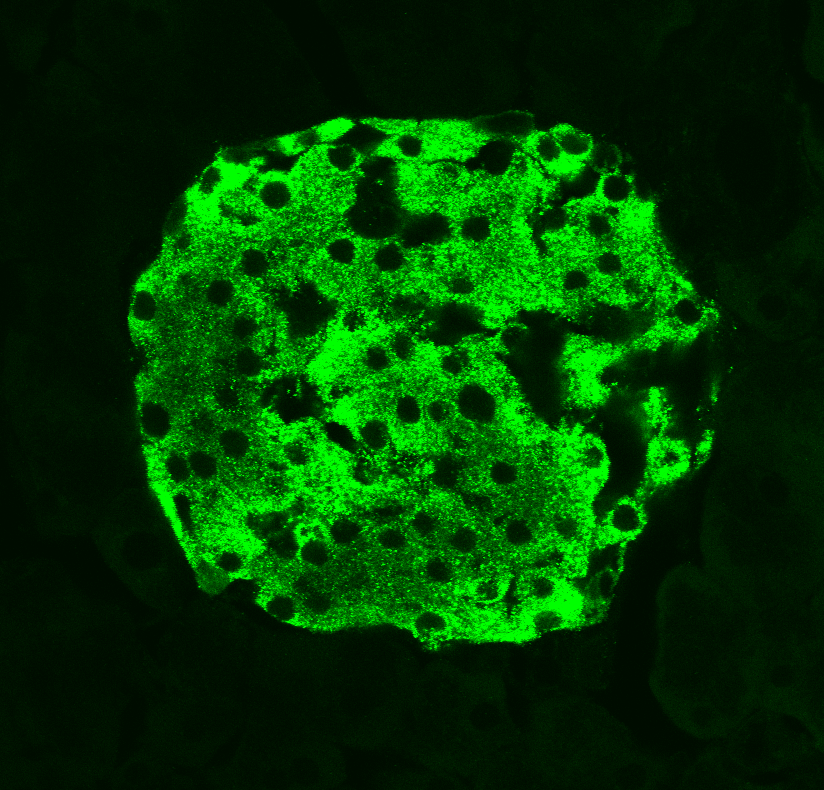

Supplement: Supplementary file 2 — Original as well as cropped pictures of islet histology shown in Extended Data Fig. 2i. [file 42255_2022_617_MOESM2_ESM.zip › Suppl_Fig_2i-islet_histology_pictures/Vhcl/Cropped/ins cropped.tif]

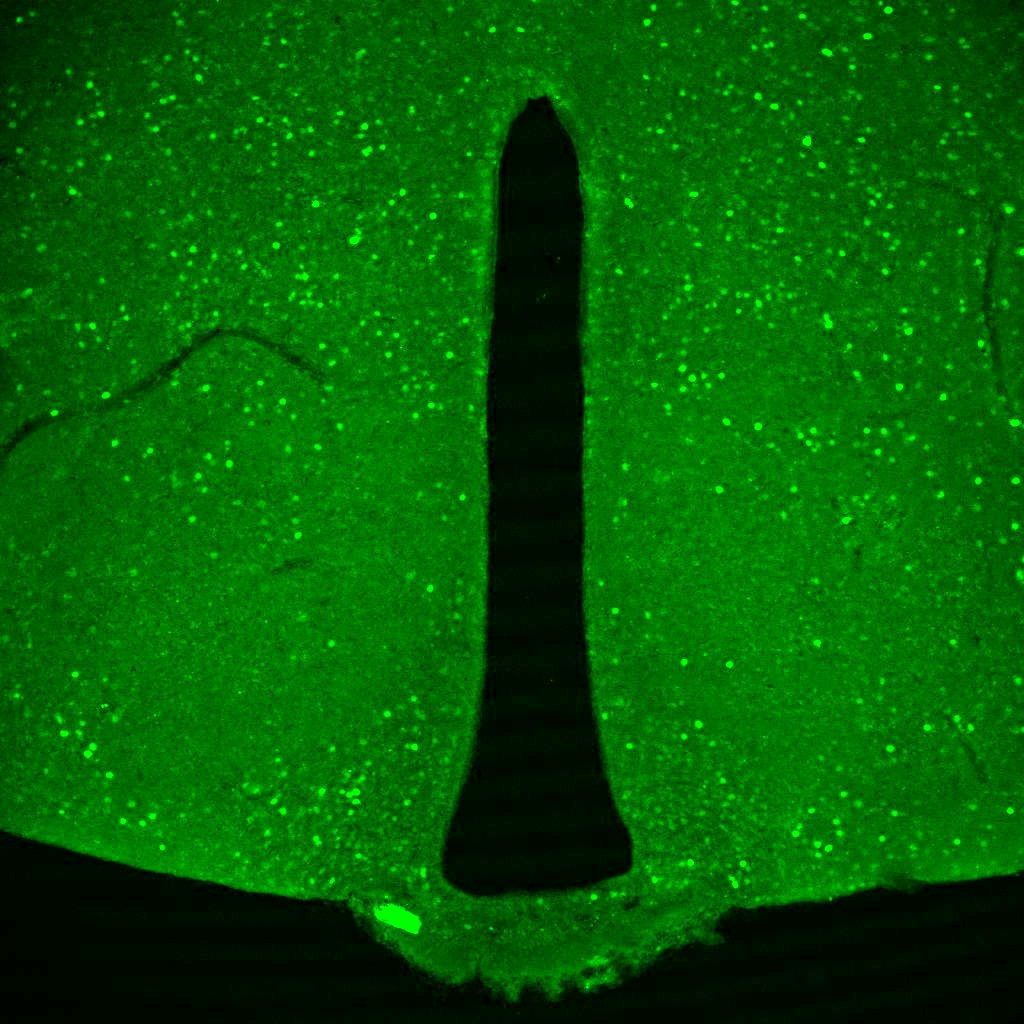

Supplement: Supplementary file 3 — Original pictures of cFOS and Cy5 drug appearance shown in Extended Data Fig. 4a–c, including replicates. [file 42255_2022_617_MOESM3_ESM.zip › Original cFOS/original_cFOS_GLP1RA_3.jpg]

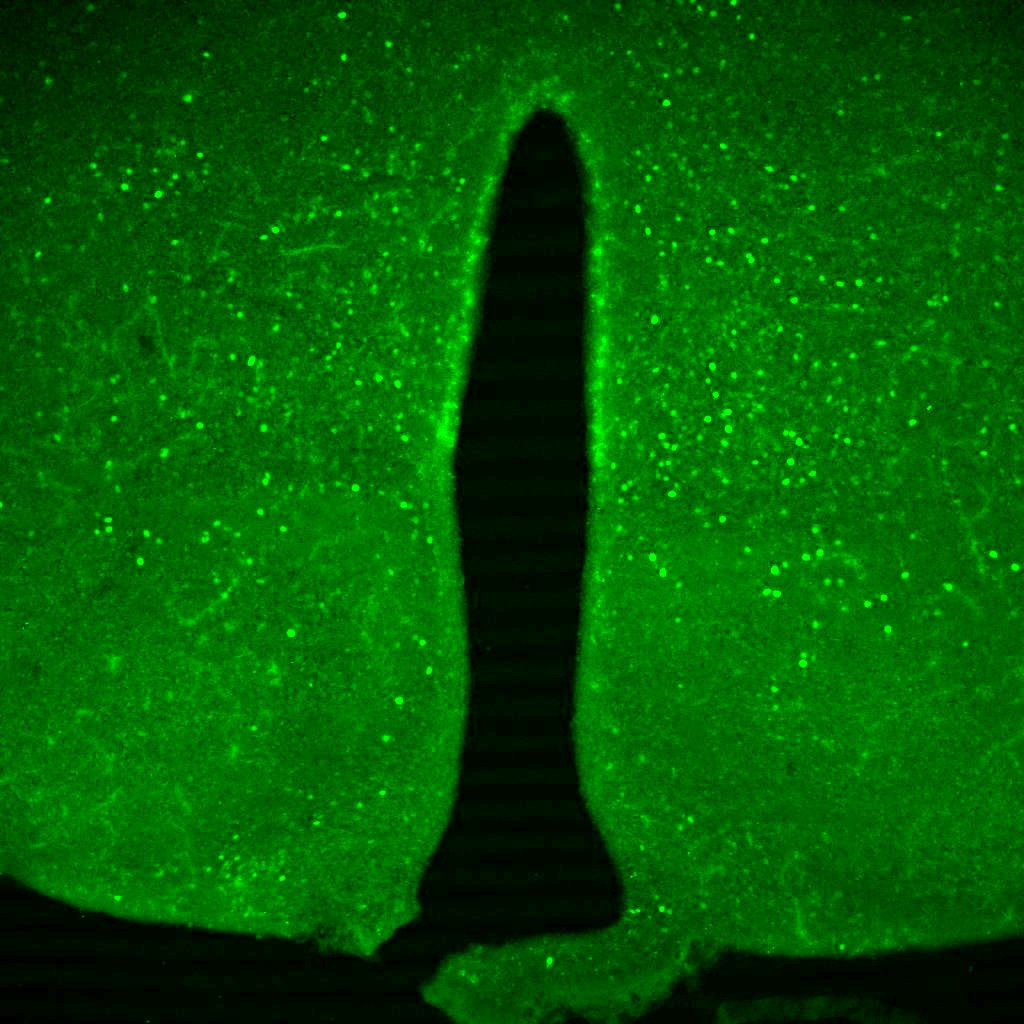

Supplement: Supplementary file 3 — Original pictures of cFOS and Cy5 drug appearance shown in Extended Data Fig. 4a–c, including replicates. [file 42255_2022_617_MOESM3_ESM.zip › Original cFOS/original_cFOS_GLP1RA_Tesa_3.jpg]

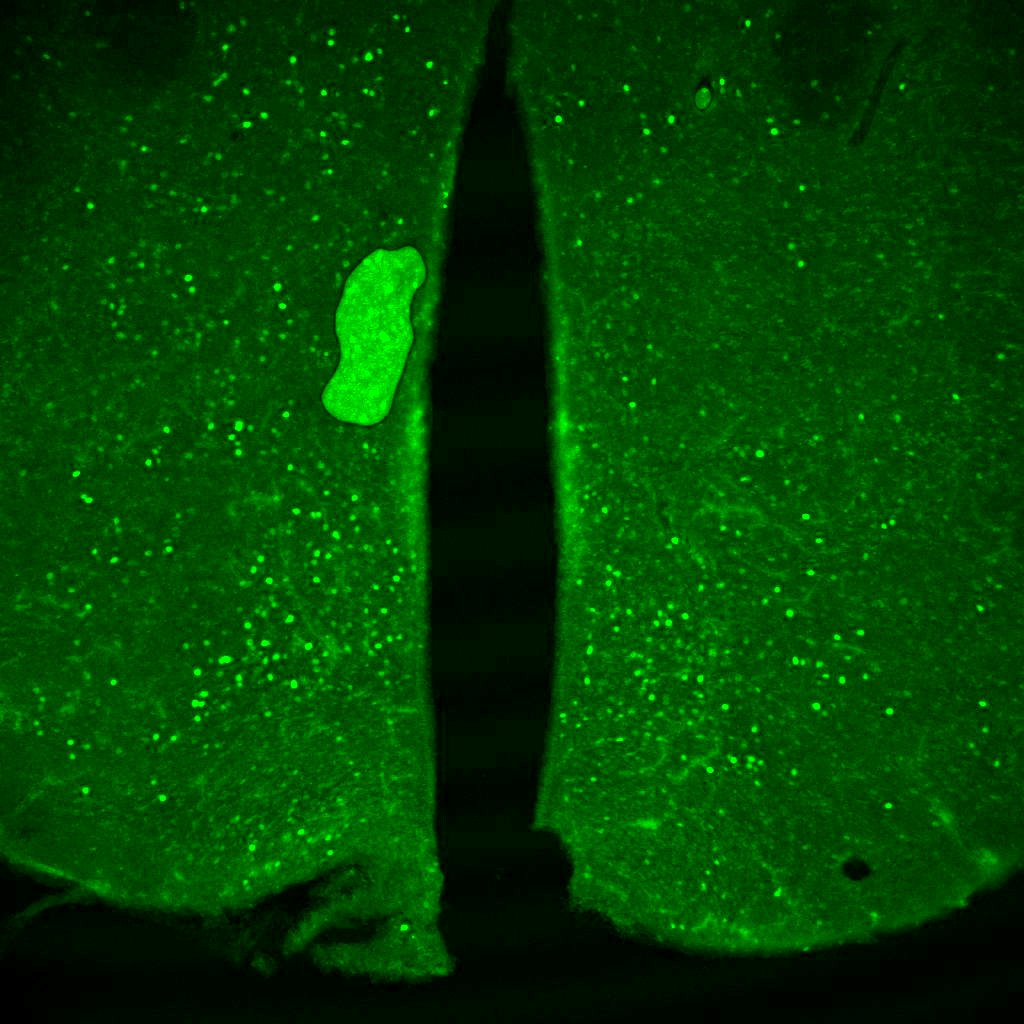

Supplement: Supplementary file 3 — Original pictures of cFOS and Cy5 drug appearance shown in Extended Data Fig. 4a–c, including replicates. [file 42255_2022_617_MOESM3_ESM.zip › Original cFOS/original_cFOS_GLP1RA_Tesa_2.jpg]

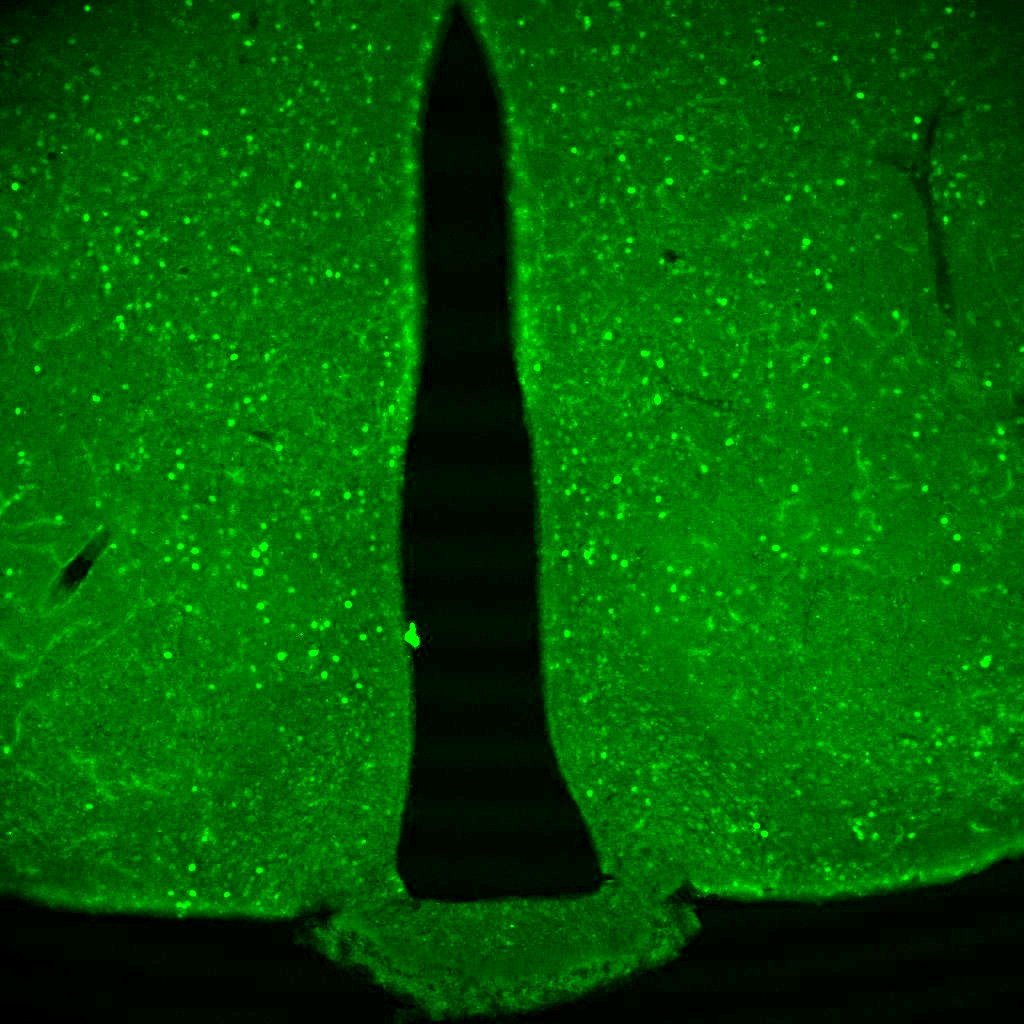

Supplement: Supplementary file 3 — Original pictures of cFOS and Cy5 drug appearance shown in Extended Data Fig. 4a–c, including replicates. [file 42255_2022_617_MOESM3_ESM.zip › Original cFOS/original_cFOS_GLP1RA_2.jpg]

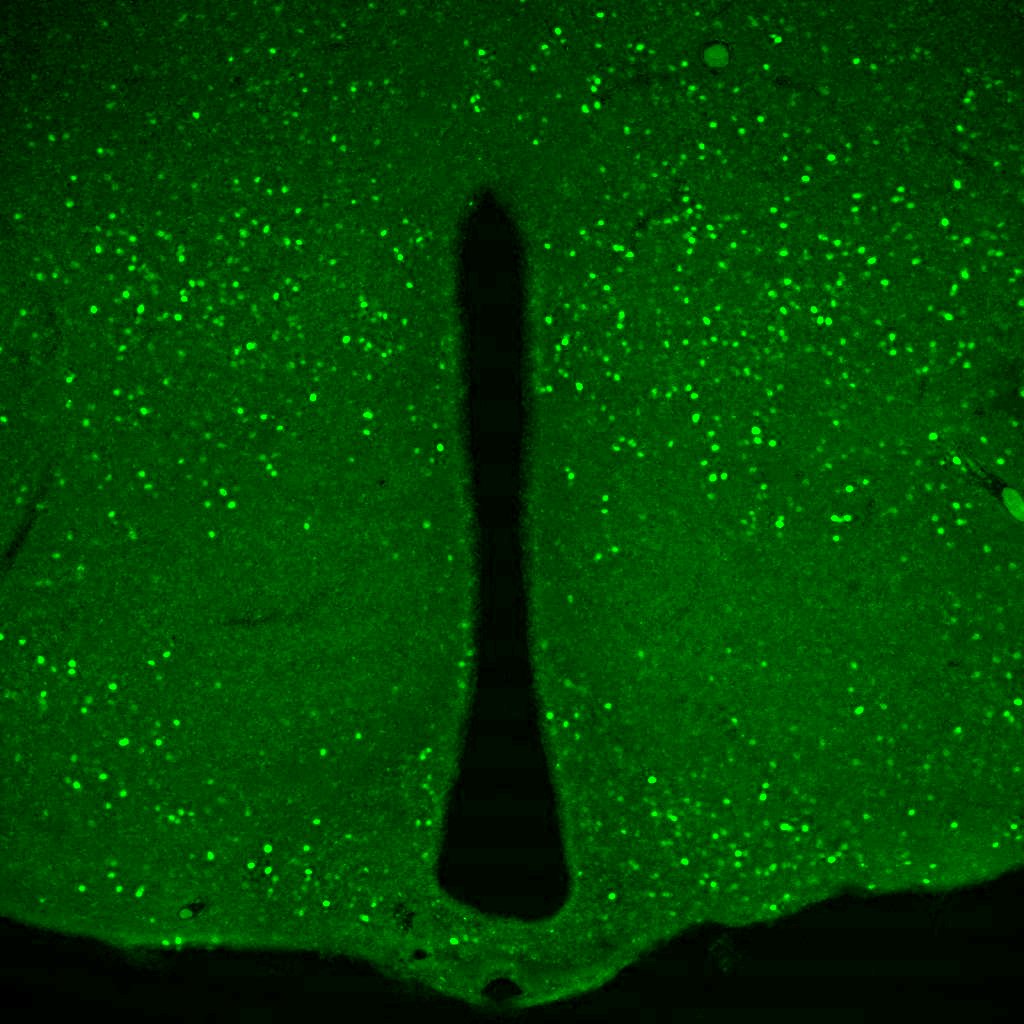

Supplement: Supplementary file 3 — Original pictures of cFOS and Cy5 drug appearance shown in Extended Data Fig. 4a–c, including replicates. [file 42255_2022_617_MOESM3_ESM.zip › Original cFOS/original_cFOS_GLP1RA_Tesa_1.jpg]

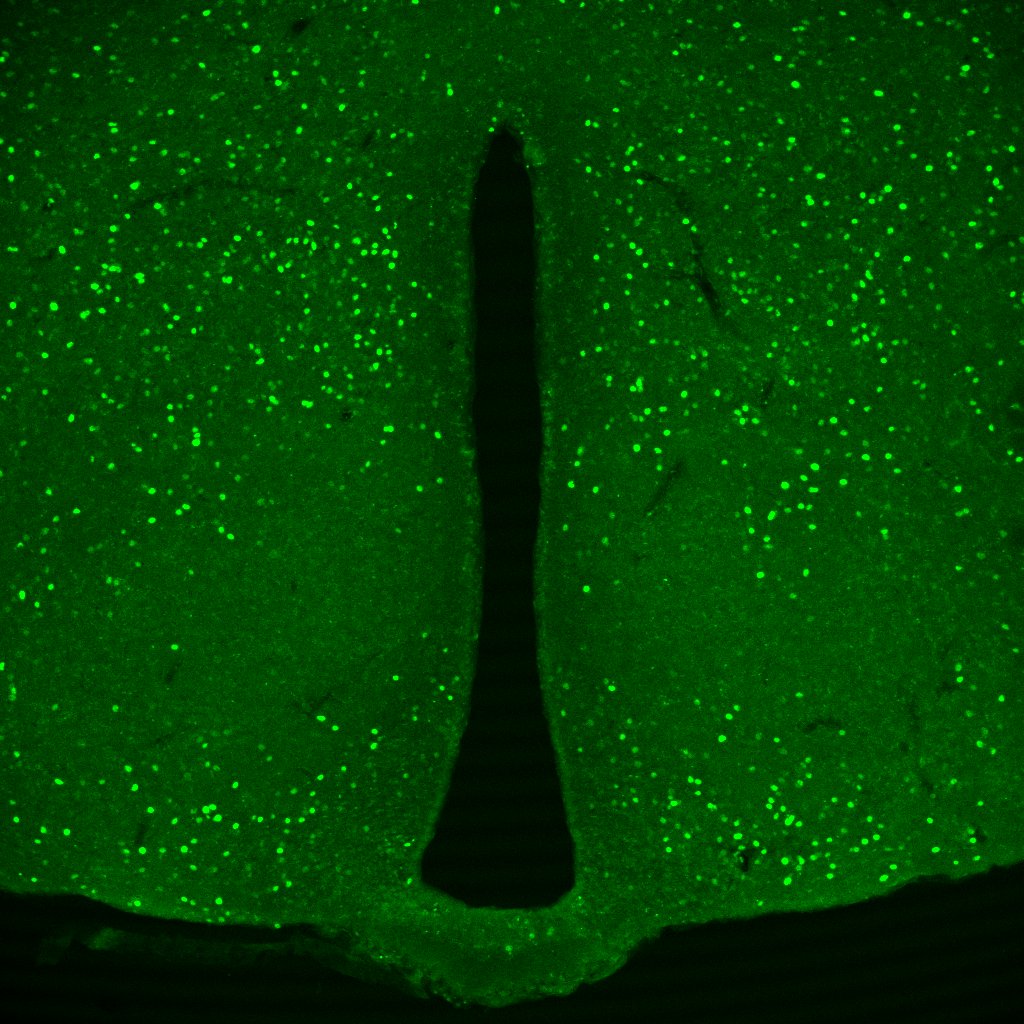

Supplement: Supplementary file 3 — Original pictures of cFOS and Cy5 drug appearance shown in Extended Data Fig. 4a–c, including replicates. [file 42255_2022_617_MOESM3_ESM.zip › Original cFOS/original_cFOS_GLP1RA_1.jpg]

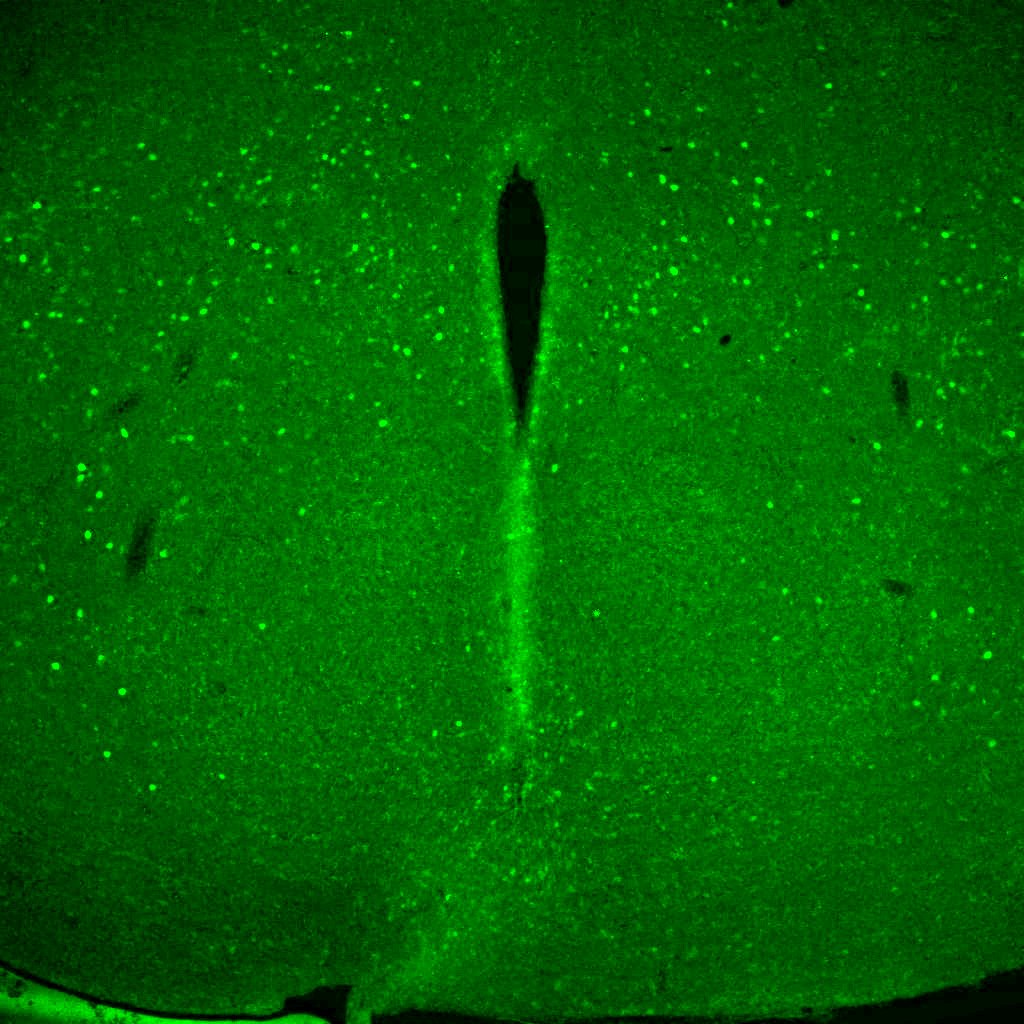

Supplement: Supplementary file 3 — Original pictures of cFOS and Cy5 drug appearance shown in Extended Data Fig. 4a–c, including replicates. [file 42255_2022_617_MOESM3_ESM.zip › Original cFOS/original_cFOS_GLP1RA_Tesa_4.jpg]

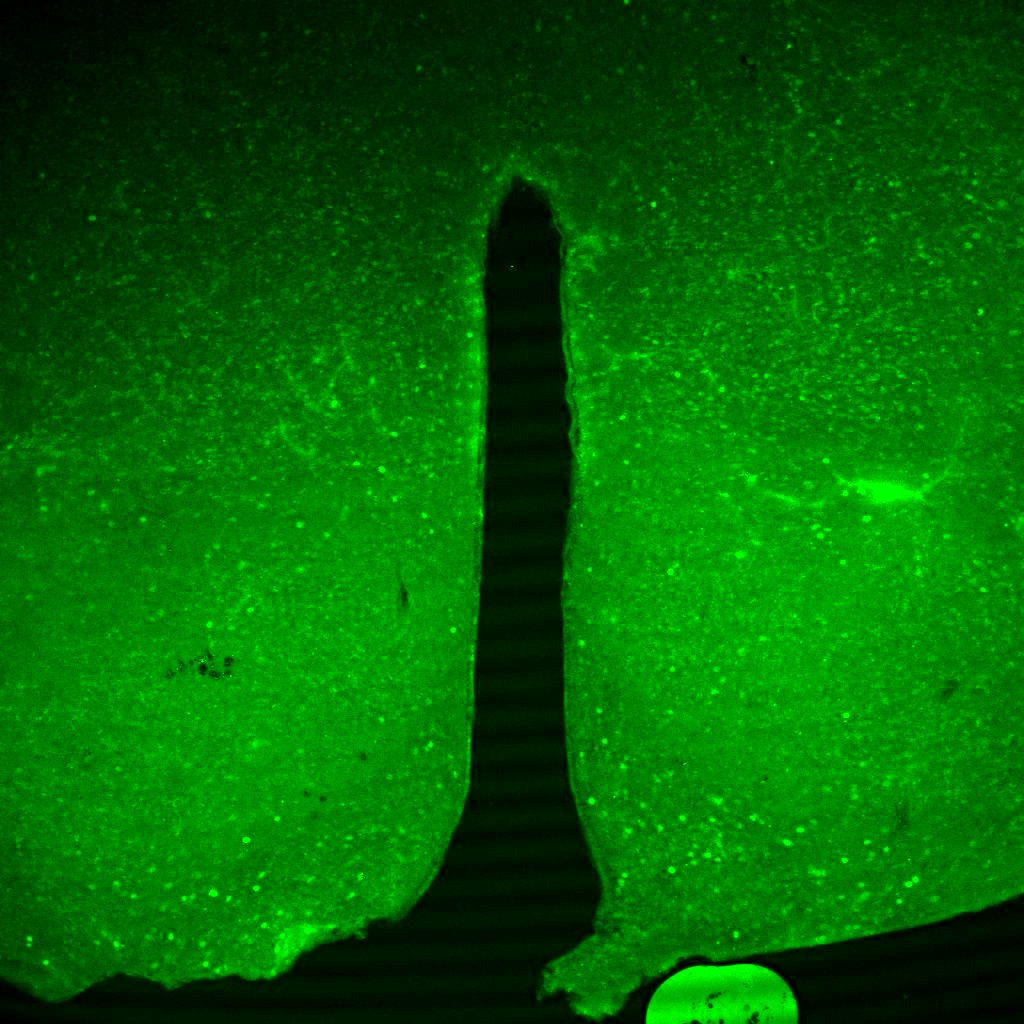

Supplement: Supplementary file 3 — Original pictures of cFOS and Cy5 drug appearance shown in Extended Data Fig. 4a–c, including replicates. [file 42255_2022_617_MOESM3_ESM.zip › Original cFOS/original_cFOS_GLP1RA_4.jpg]

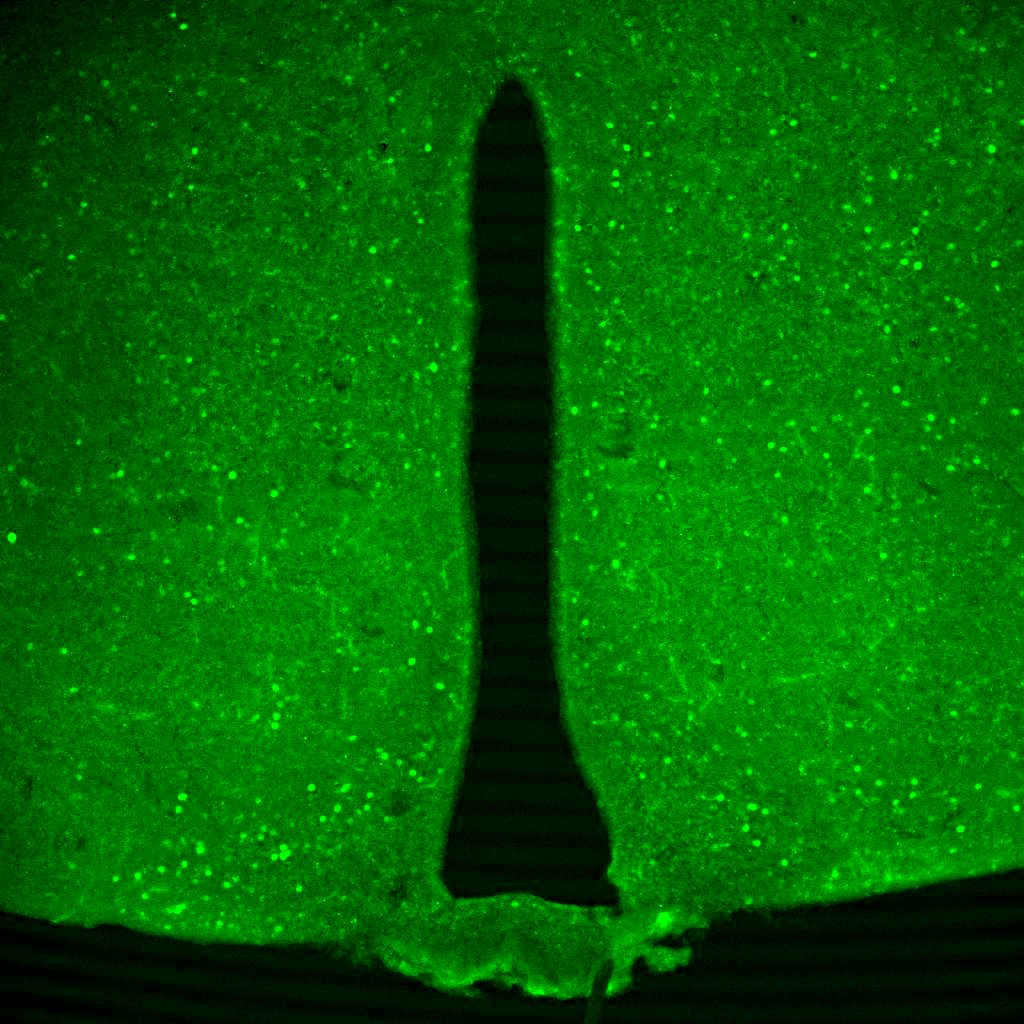

Supplement: Supplementary file 3 — Original pictures of cFOS and Cy5 drug appearance shown in Extended Data Fig. 4a–c, including replicates. [file 42255_2022_617_MOESM3_ESM.zip › Original cFOS/original_cFOS_Veh_1.jpg]

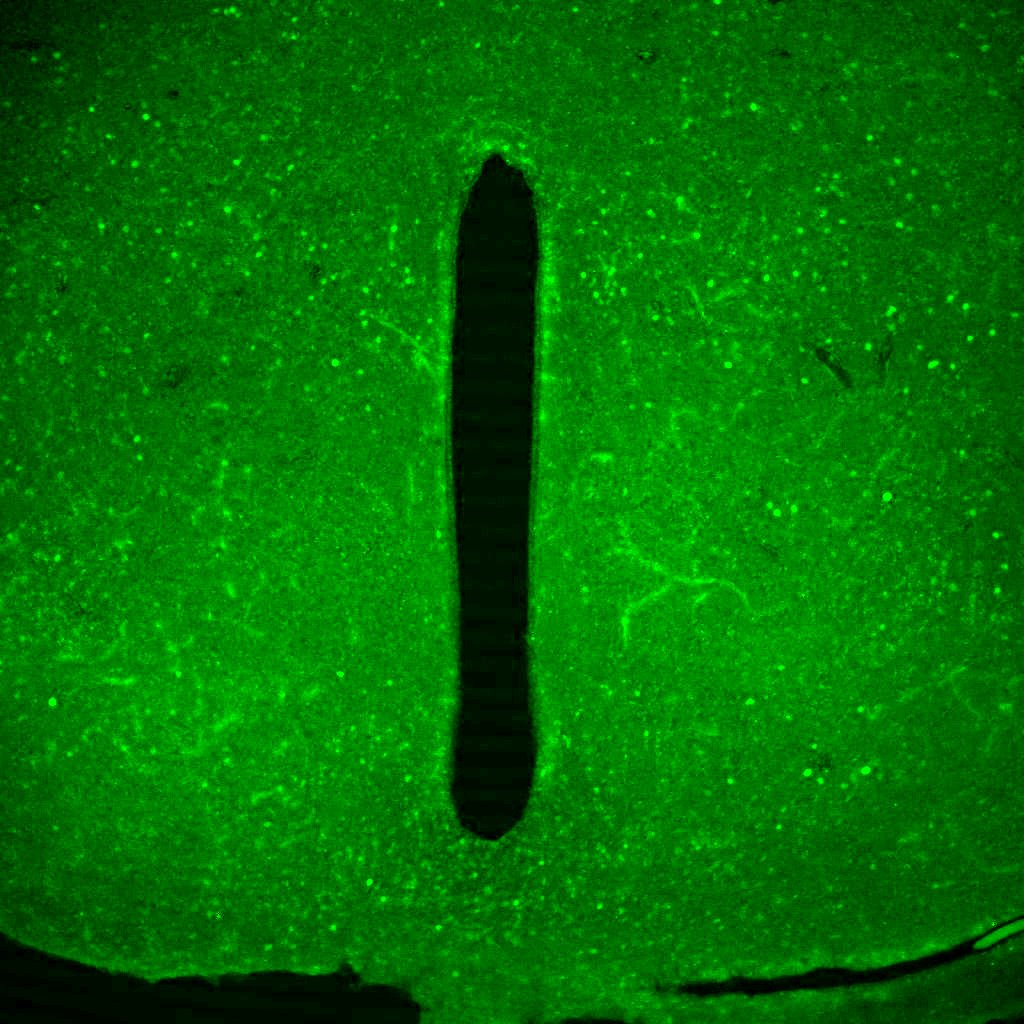

Supplement: Supplementary file 3 — Original pictures of cFOS and Cy5 drug appearance shown in Extended Data Fig. 4a–c, including replicates. [file 42255_2022_617_MOESM3_ESM.zip › Original cFOS/original_cFOS_Tesa_4.jpg]

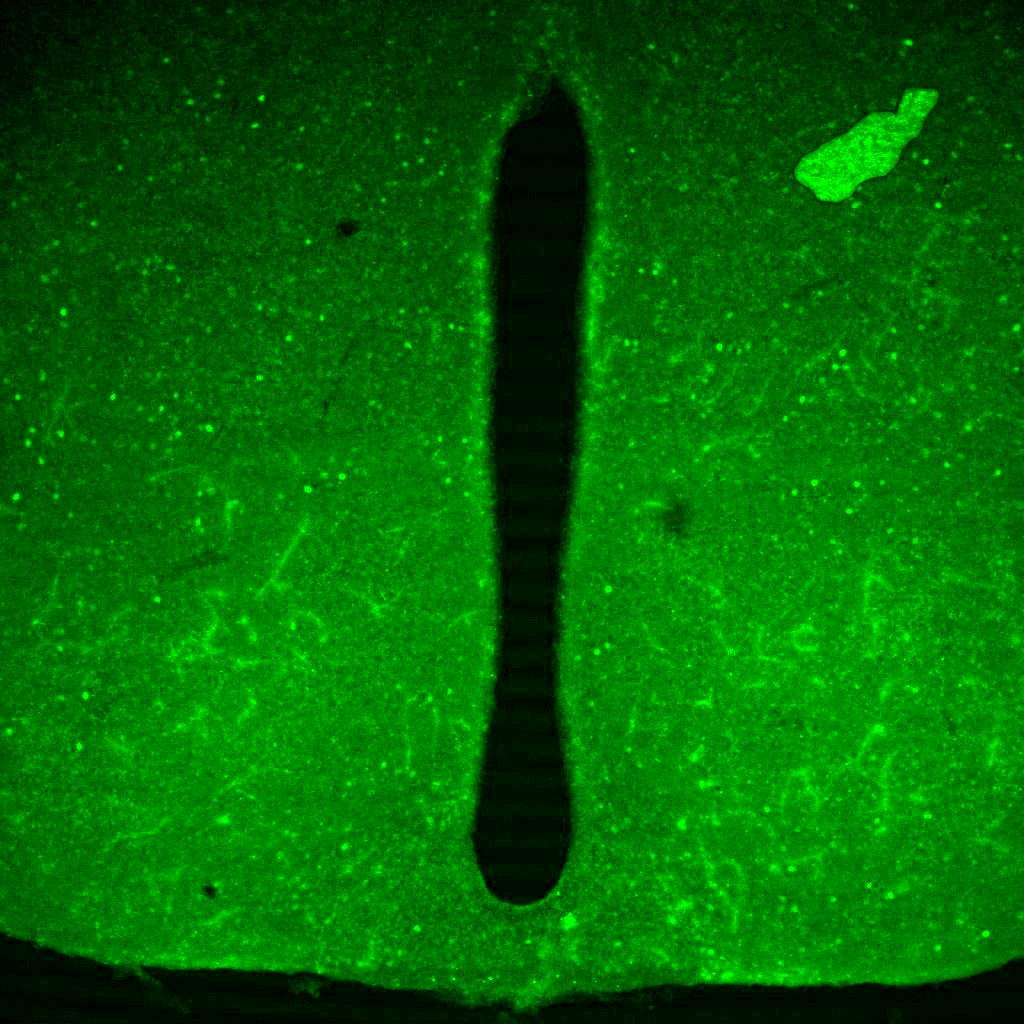

Supplement: Supplementary file 3 — Original pictures of cFOS and Cy5 drug appearance shown in Extended Data Fig. 4a–c, including replicates. [file 42255_2022_617_MOESM3_ESM.zip › Original cFOS/original_cFOS_Veh_2.jpg]

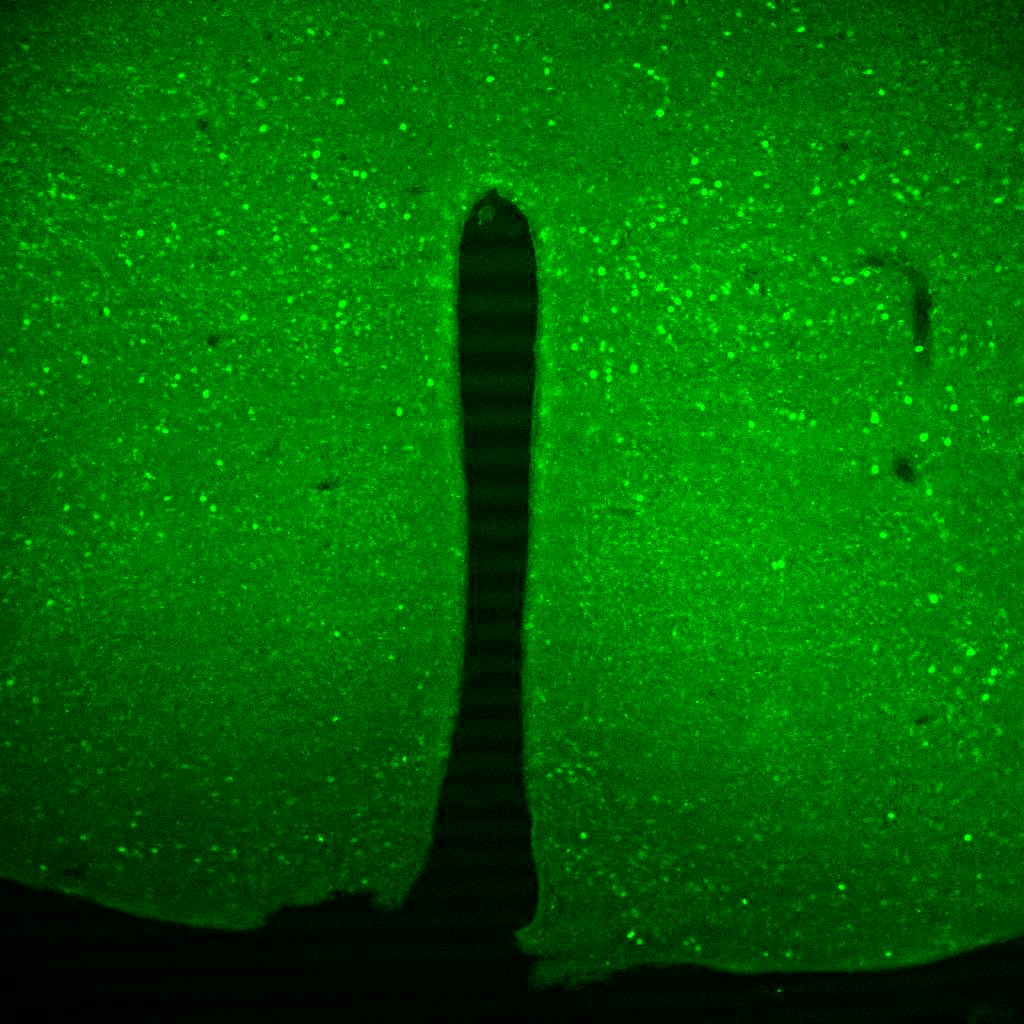

Supplement: Supplementary file 3 — Original pictures of cFOS and Cy5 drug appearance shown in Extended Data Fig. 4a–c, including replicates. [file 42255_2022_617_MOESM3_ESM.zip › Original cFOS/original_cFOS_Veh_3.jpg]

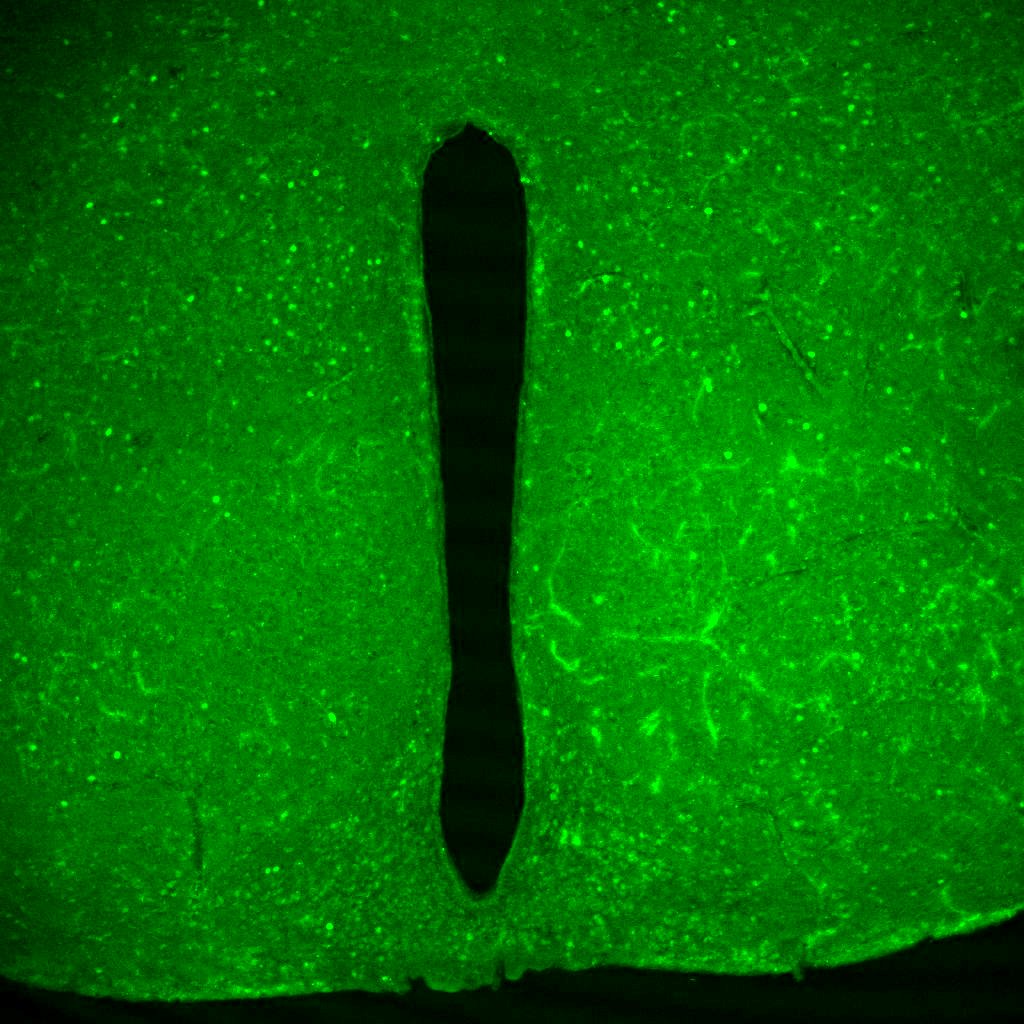

Supplement: Supplementary file 3 — Original pictures of cFOS and Cy5 drug appearance shown in Extended Data Fig. 4a–c, including replicates. [file 42255_2022_617_MOESM3_ESM.zip › Original cFOS/original_cFOS_Tesa_3.jpg]

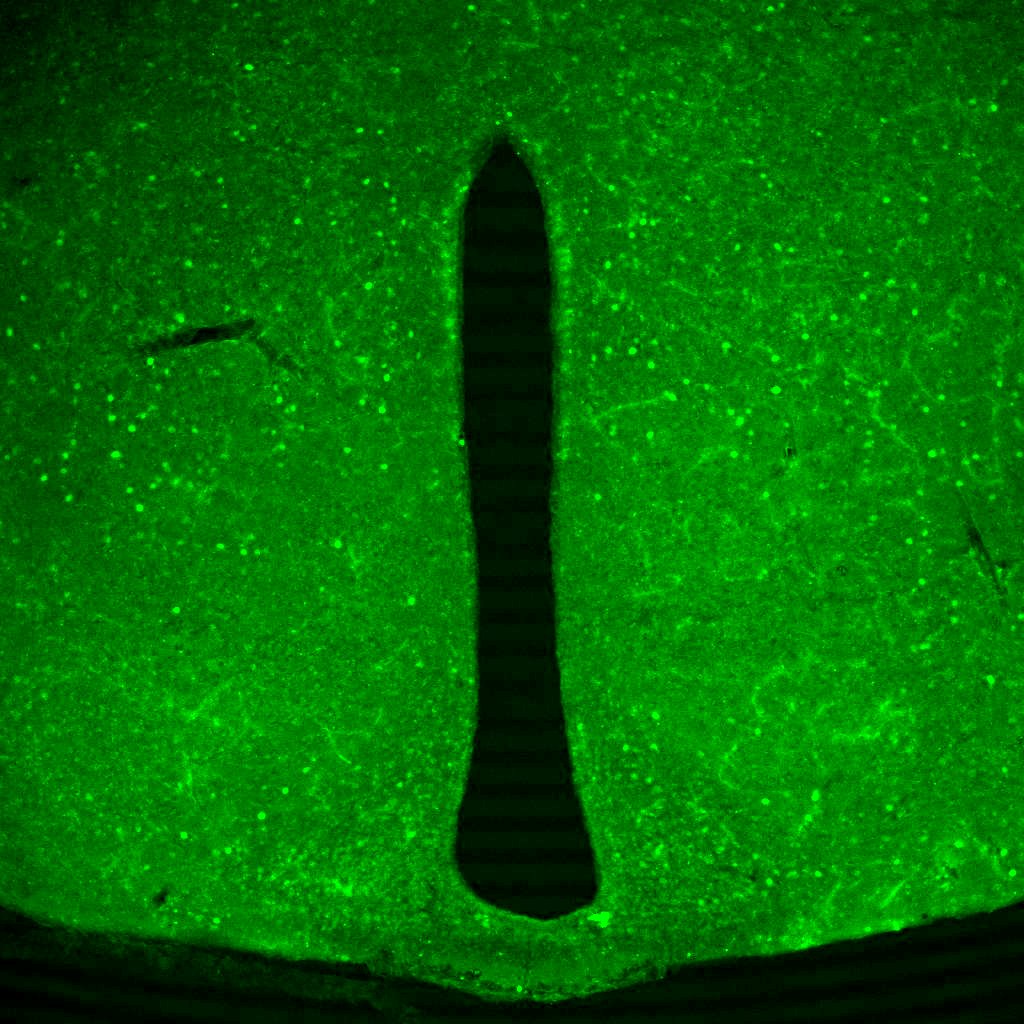

Supplement: Supplementary file 3 — Original pictures of cFOS and Cy5 drug appearance shown in Extended Data Fig. 4a–c, including replicates. [file 42255_2022_617_MOESM3_ESM.zip › Original cFOS/original_cFOS_Tesa_2.jpg]

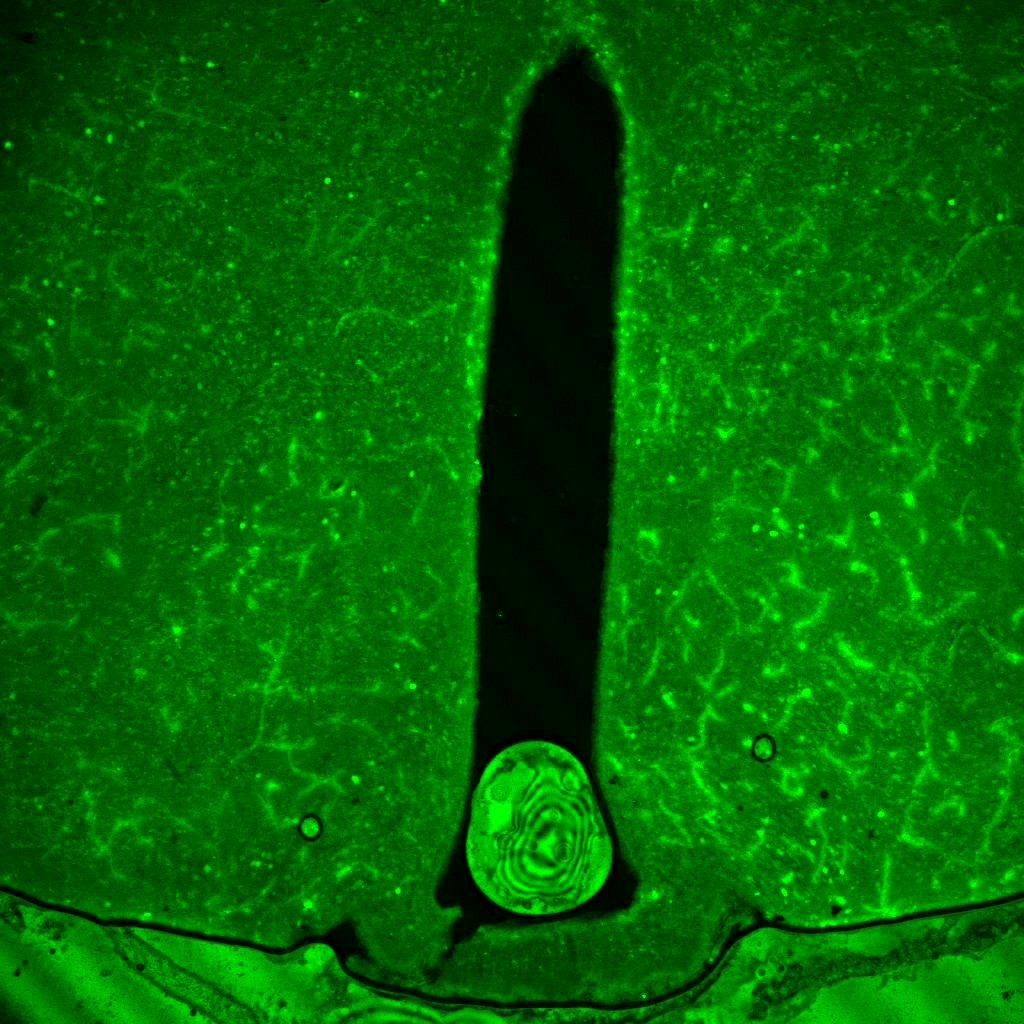

Supplement: Supplementary file 3 — Original pictures of cFOS and Cy5 drug appearance shown in Extended Data Fig. 4a–c, including replicates. [file 42255_2022_617_MOESM3_ESM.zip › Original cFOS/original_cFOS_Veh_4.jpg]

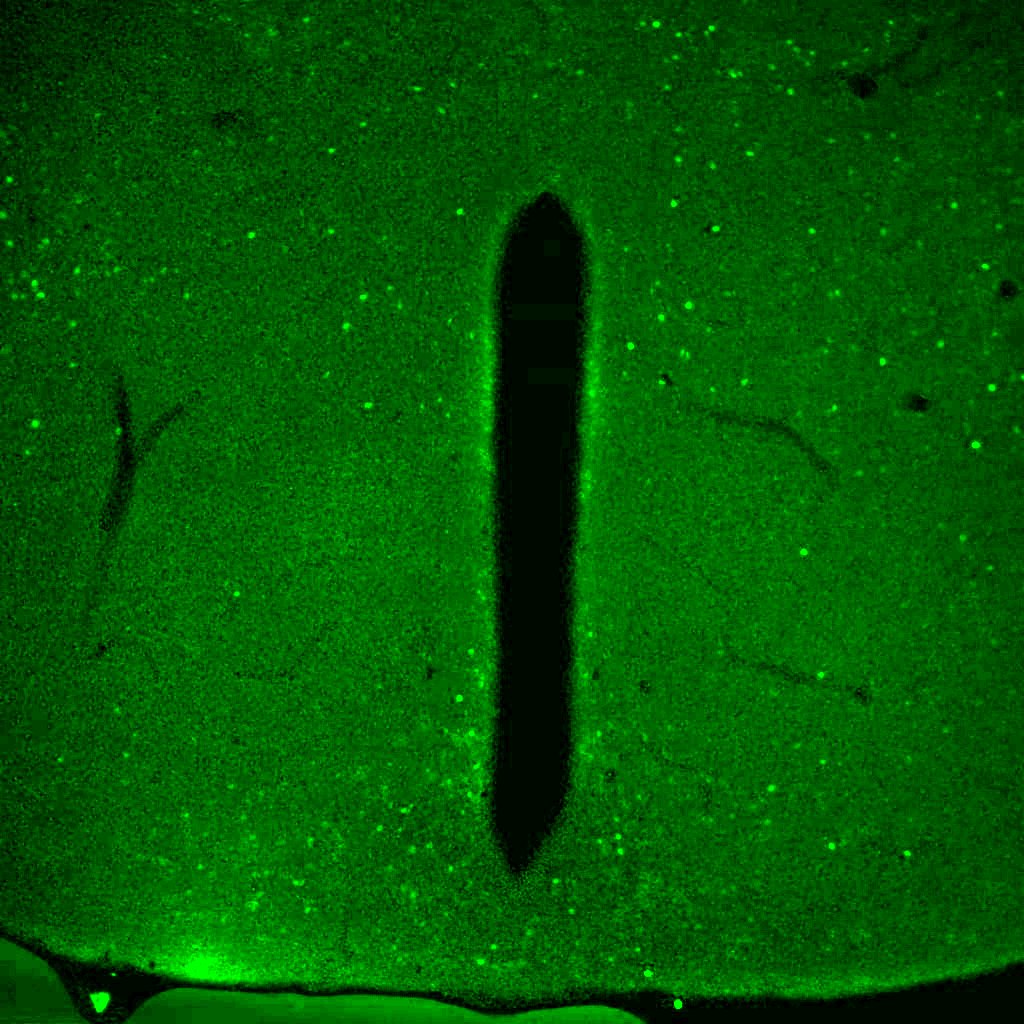

Supplement: Supplementary file 3 — Original pictures of cFOS and Cy5 drug appearance shown in Extended Data Fig. 4a–c, including replicates. [file 42255_2022_617_MOESM3_ESM.zip › Original cFOS/original_cFOS_Tesa_1.jpg]

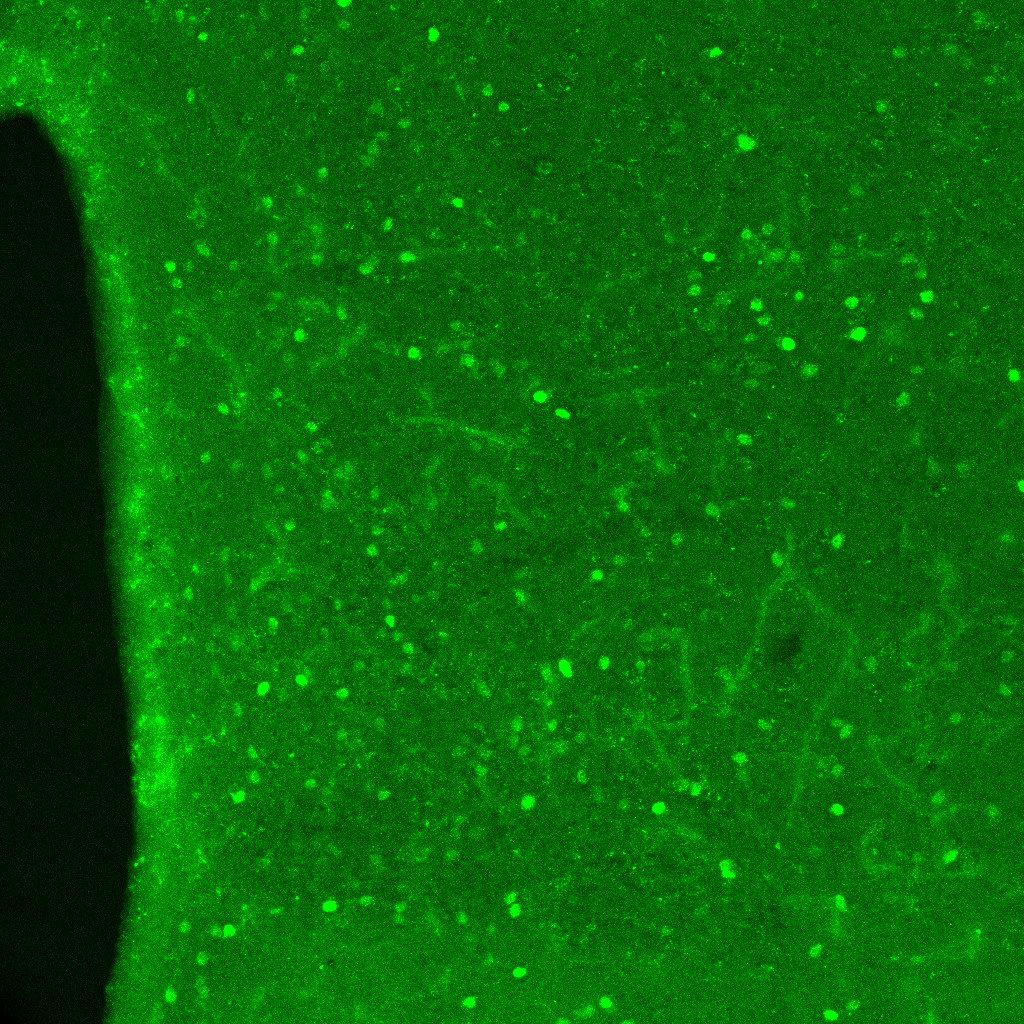

Supplement: Supplementary file 3 — Original pictures of cFOS and Cy5 drug appearance shown in Extended Data Fig. 4a–c, including replicates. [file 42255_2022_617_MOESM3_ESM.zip › Suppl_Fig_4a-GLP-1:Tesa_x20_DMH.jpg]

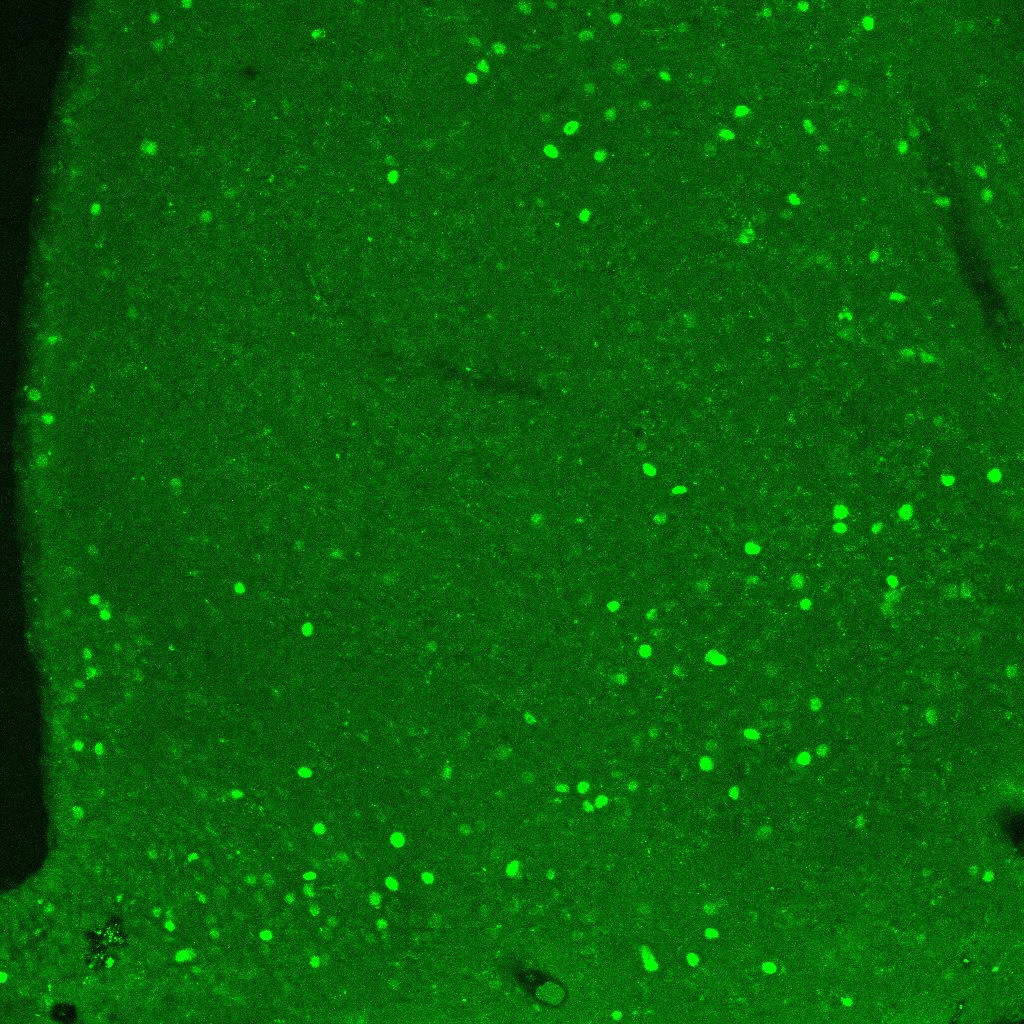

Supplement: Supplementary file 3 — Original pictures of cFOS and Cy5 drug appearance shown in Extended Data Fig. 4a–c, including replicates. [file 42255_2022_617_MOESM3_ESM.zip › Suppl_Fig_4a-GLP-1:Tesa_x20_VMH.jpg]

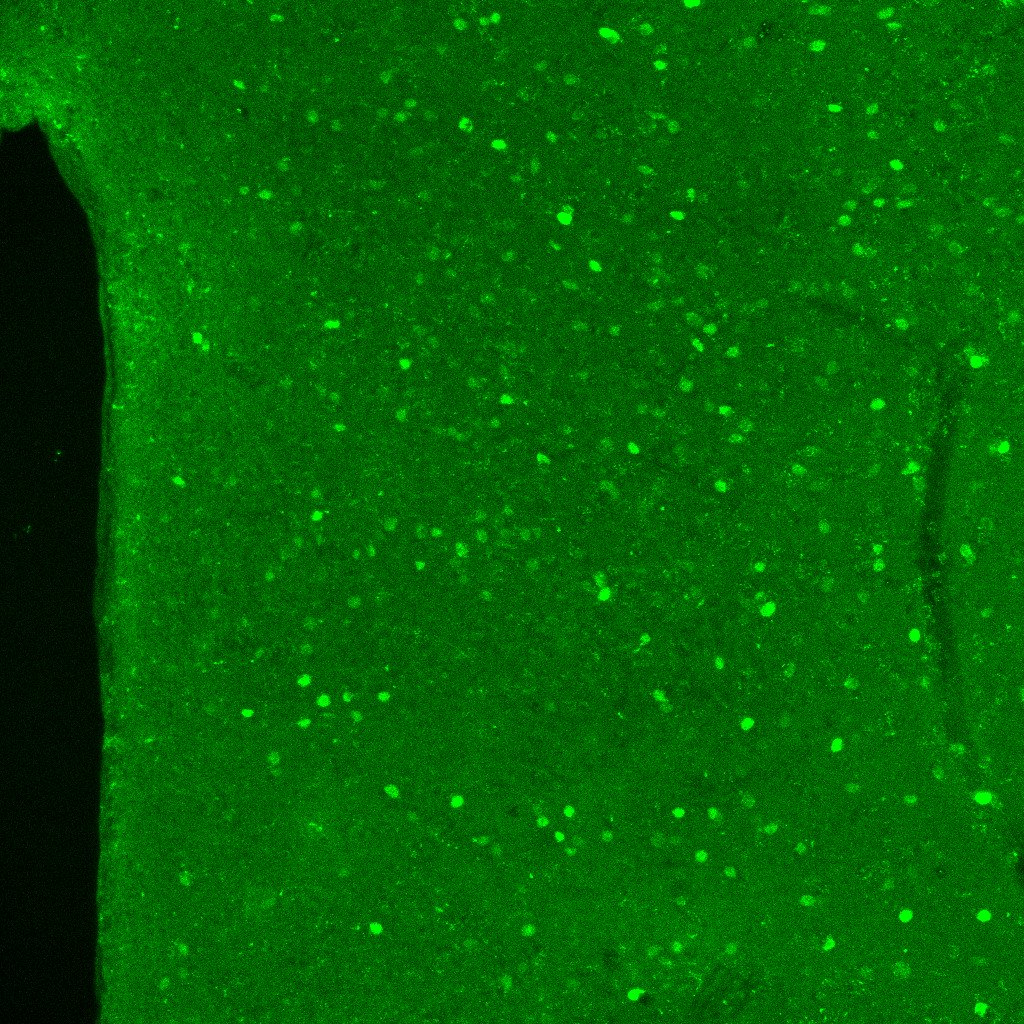

Supplement: Supplementary file 3 — Original pictures of cFOS and Cy5 drug appearance shown in Extended Data Fig. 4a–c, including replicates. [file 42255_2022_617_MOESM3_ESM.zip › Suppl_Fig_4a-GLP1RA_x20_DMH.jpg]

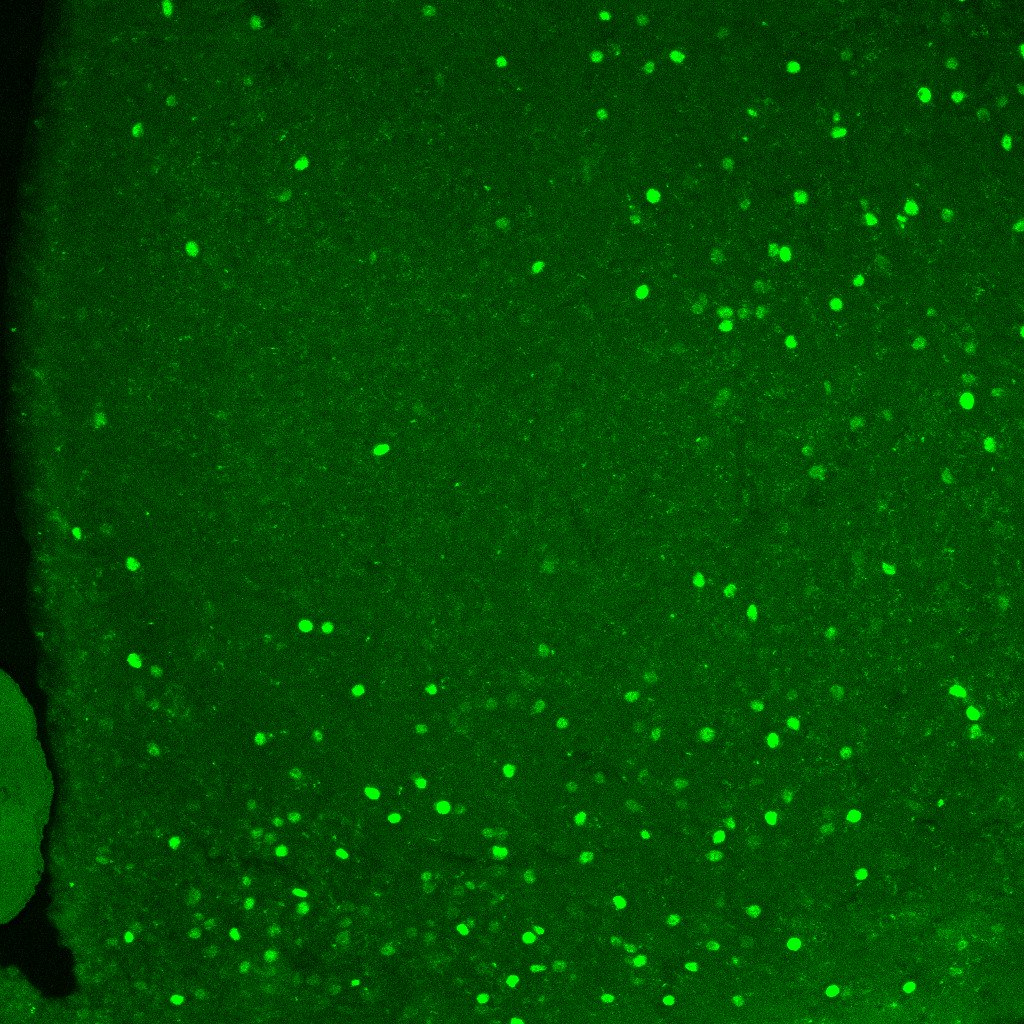

Supplement: Supplementary file 3 — Original pictures of cFOS and Cy5 drug appearance shown in Extended Data Fig. 4a–c, including replicates. [file 42255_2022_617_MOESM3_ESM.zip › Suppl_Fig_4a-GLP1RA_x20_VMH.jpg]

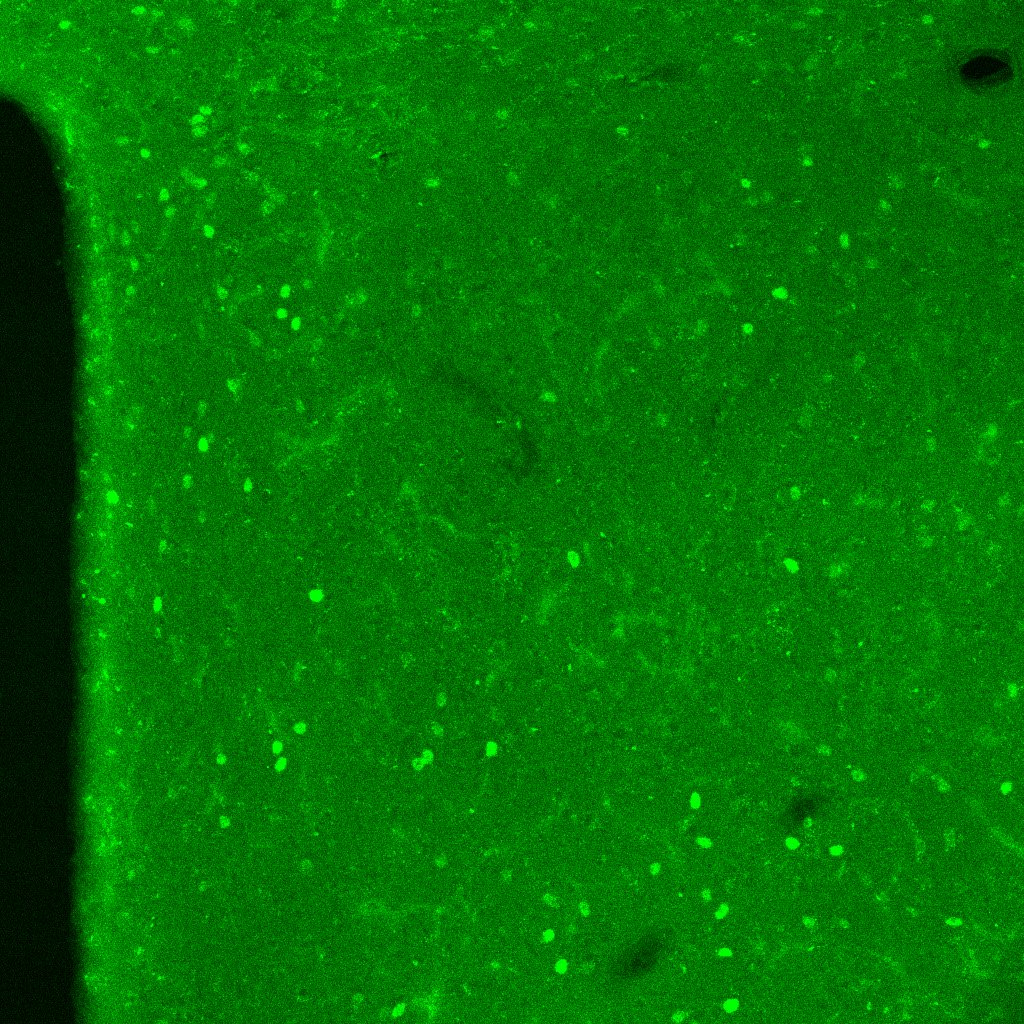

Supplement: Supplementary file 3 — Original pictures of cFOS and Cy5 drug appearance shown in Extended Data Fig. 4a–c, including replicates. [file 42255_2022_617_MOESM3_ESM.zip › Suppl_Fig_4a-Tesa_x20_DMH.jpg]

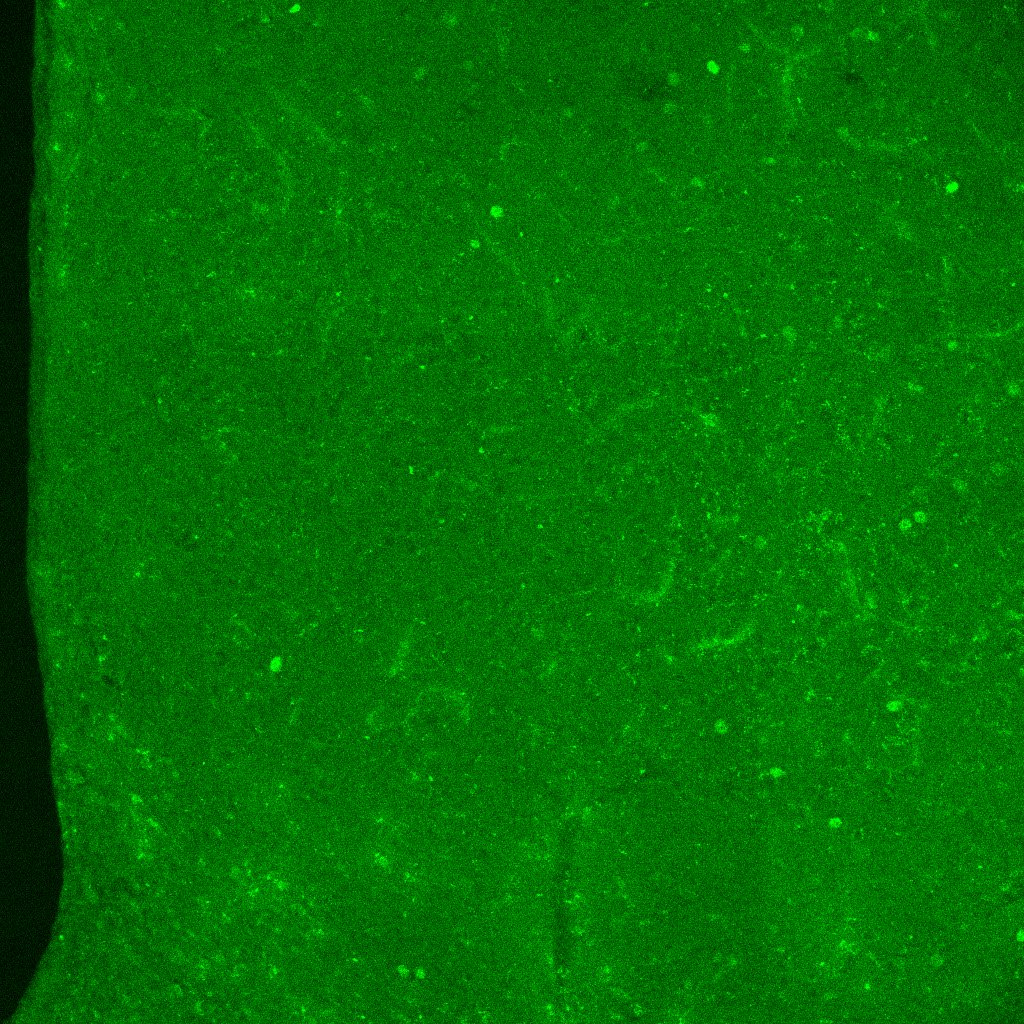

Supplement: Supplementary file 3 — Original pictures of cFOS and Cy5 drug appearance shown in Extended Data Fig. 4a–c, including replicates. [file 42255_2022_617_MOESM3_ESM.zip › Suppl_Fig_4a-Tesa_x20_VMH.jpg]

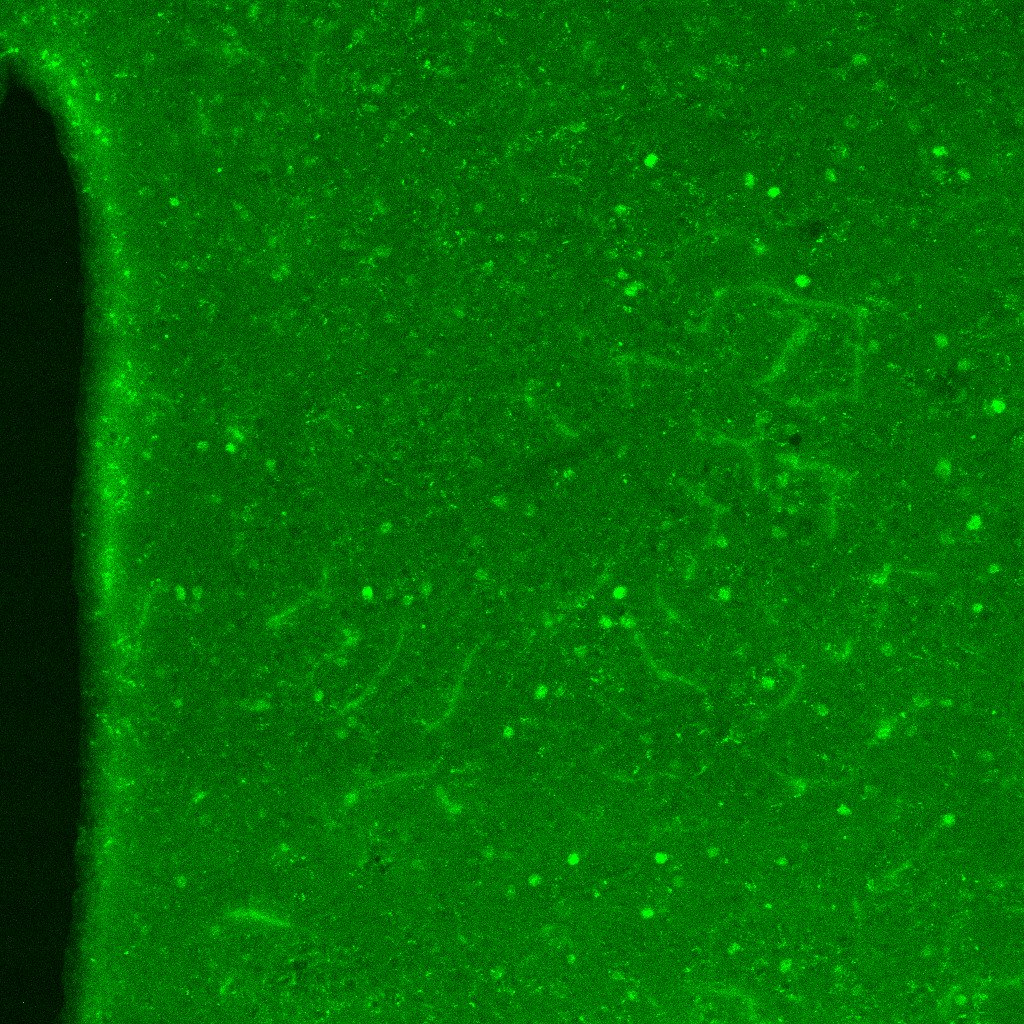

Supplement: Supplementary file 3 — Original pictures of cFOS and Cy5 drug appearance shown in Extended Data Fig. 4a–c, including replicates. [file 42255_2022_617_MOESM3_ESM.zip › Suppl_Fig_4a-Vhcl_x20_DMH.jpg]

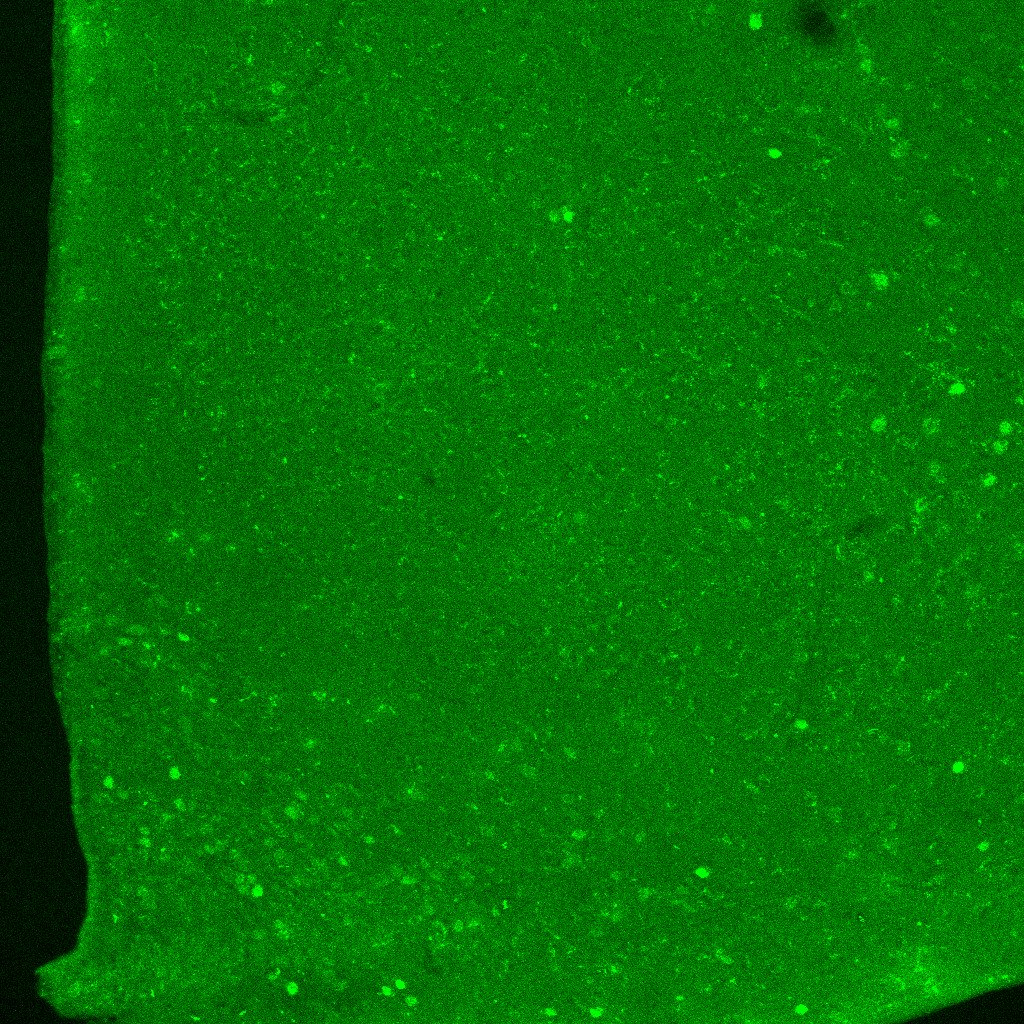

Supplement: Supplementary file 3 — Original pictures of cFOS and Cy5 drug appearance shown in Extended Data Fig. 4a–c, including replicates. [file 42255_2022_617_MOESM3_ESM.zip › Suppl_Fig_4a-Vhcl_x20_VMH.jpg]

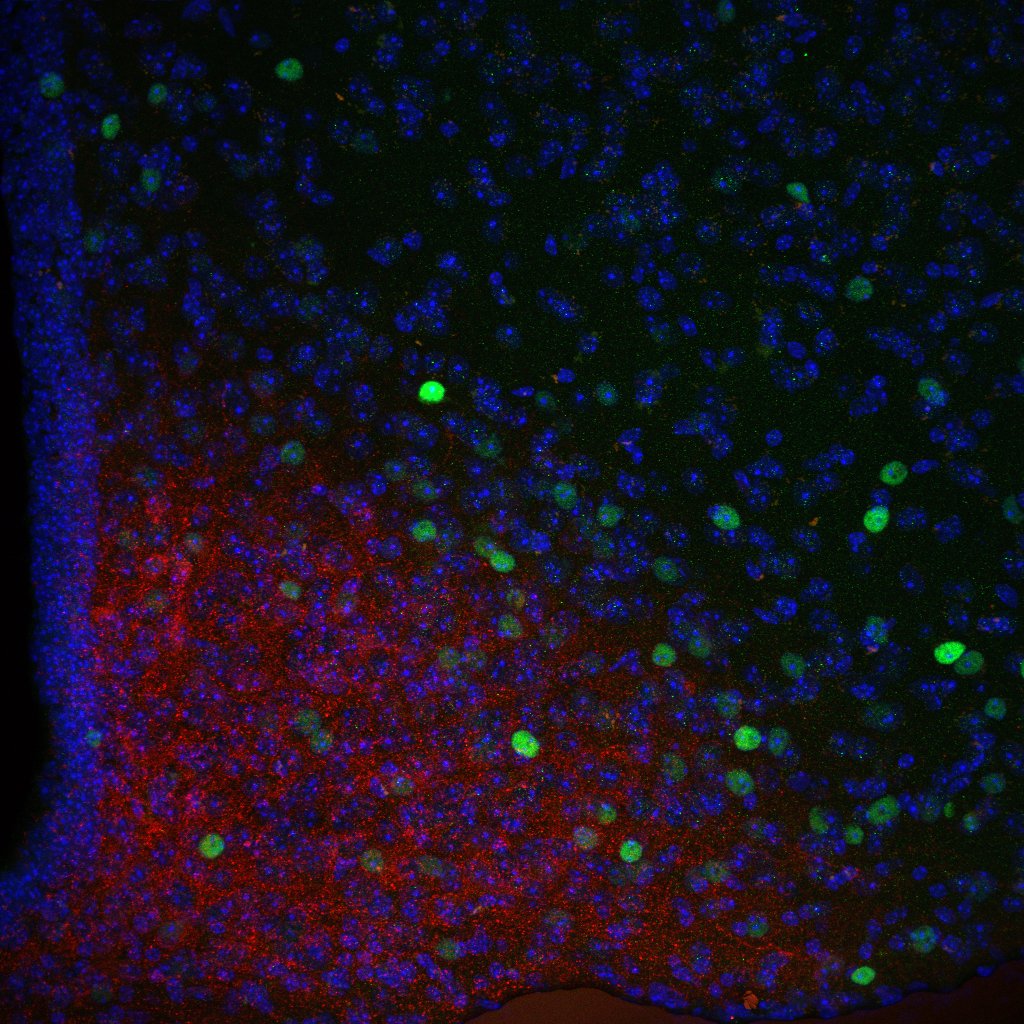

Supplement: Supplementary file 3 — Original pictures of cFOS and Cy5 drug appearance shown in Extended Data Fig. 4a–c, including replicates. [file 42255_2022_617_MOESM3_ESM.zip › Suppl_Fig_4d-GLP-1:Tesa-Cy5 ARC.jpg]

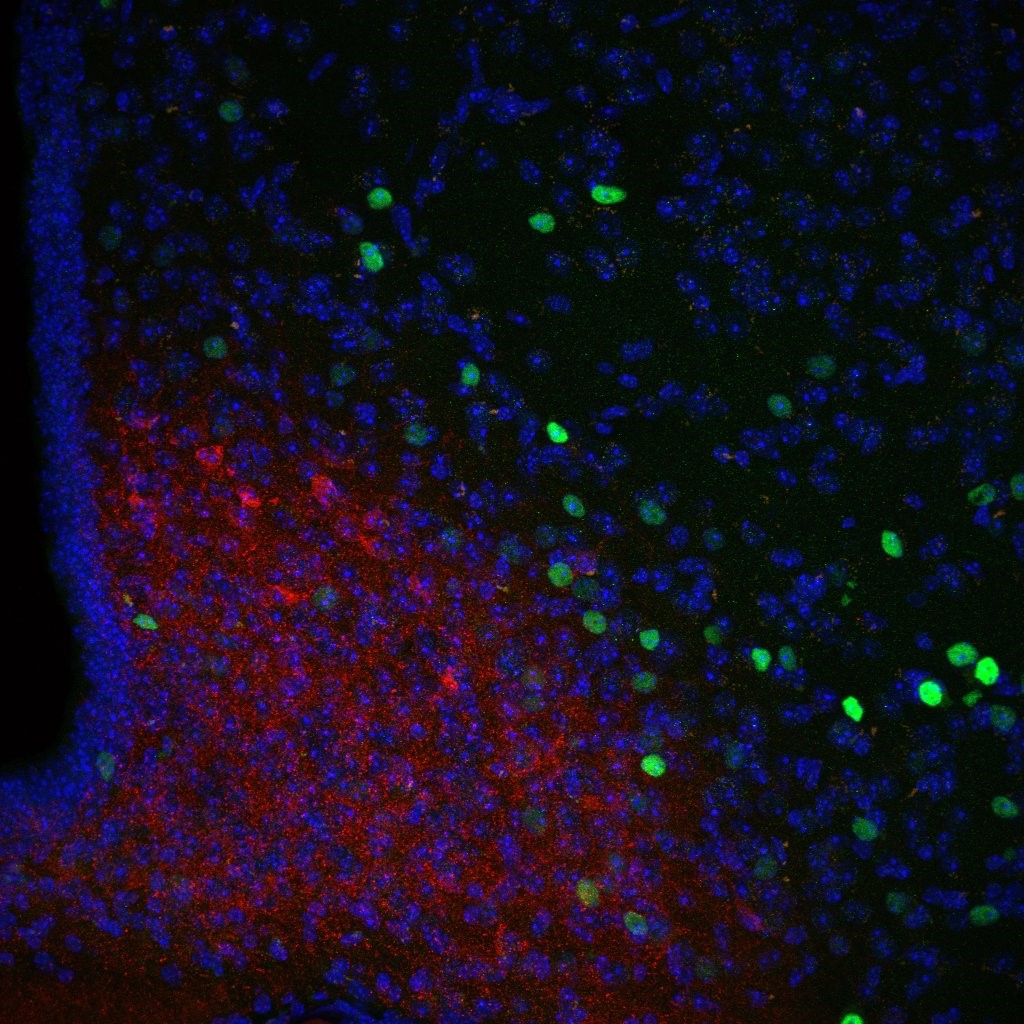

Supplement: Supplementary file 3 — Original pictures of cFOS and Cy5 drug appearance shown in Extended Data Fig. 4a–c, including replicates. [file 42255_2022_617_MOESM3_ESM.zip › Suppl_Fig_4d-GLP-1RA-Cy5 ARC.jpg]

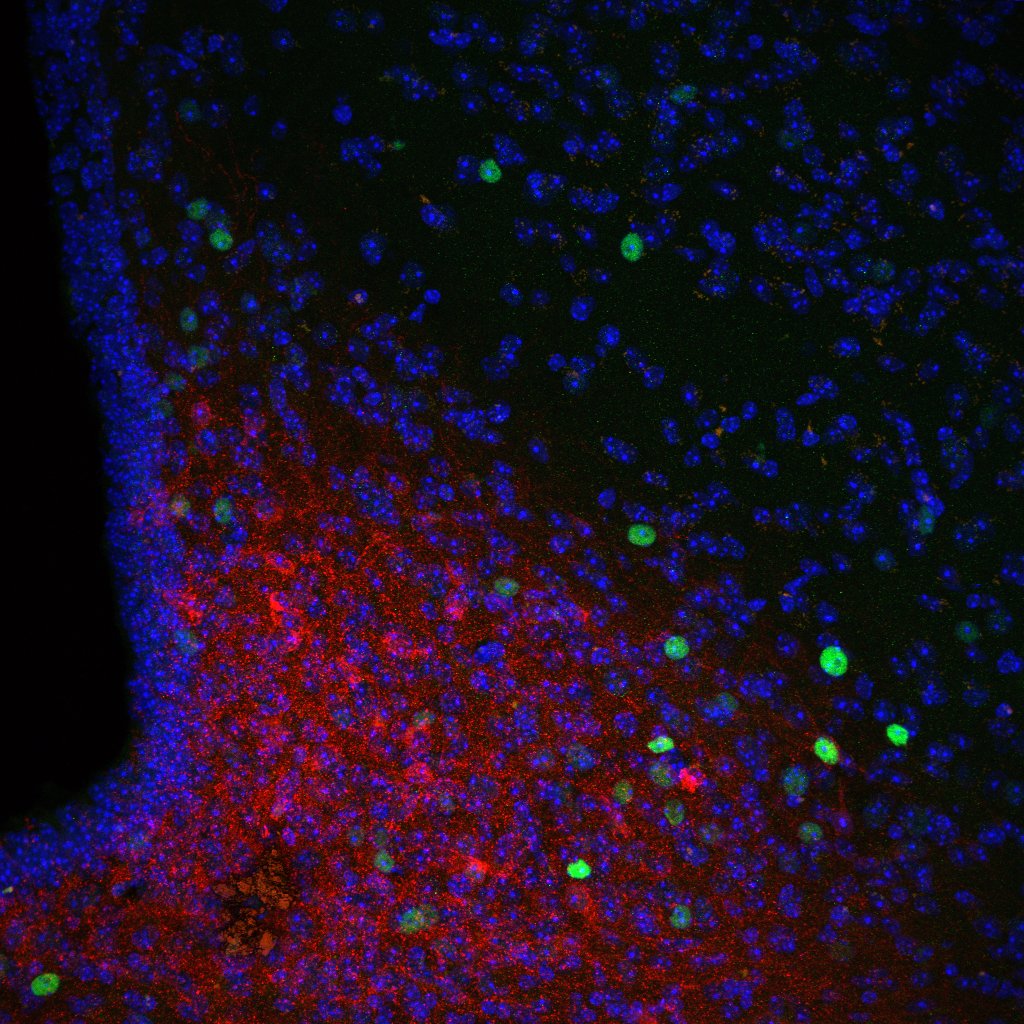

Supplement: Supplementary file 3 — Original pictures of cFOS and Cy5 drug appearance shown in Extended Data Fig. 4a–c, including replicates. [file 42255_2022_617_MOESM3_ESM.zip › Suppl_Fig_4d-replicate2-GLP-1:Tesa-Cy5 ARC.jpg]

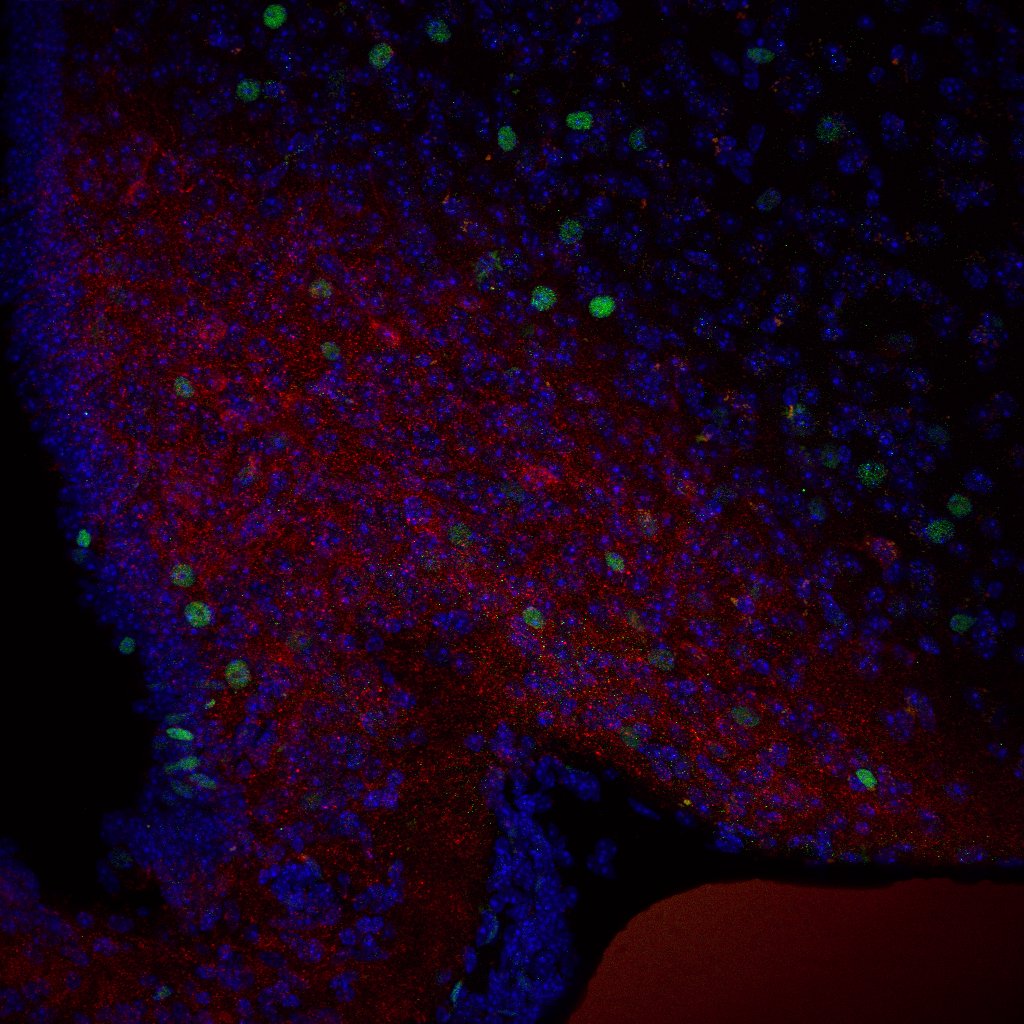

Supplement: Supplementary file 3 — Original pictures of cFOS and Cy5 drug appearance shown in Extended Data Fig. 4a–c, including replicates. [file 42255_2022_617_MOESM3_ESM.zip › Suppl_Fig_4d-replicate2-GLP-1RA-Cy5 ARC.jpg]

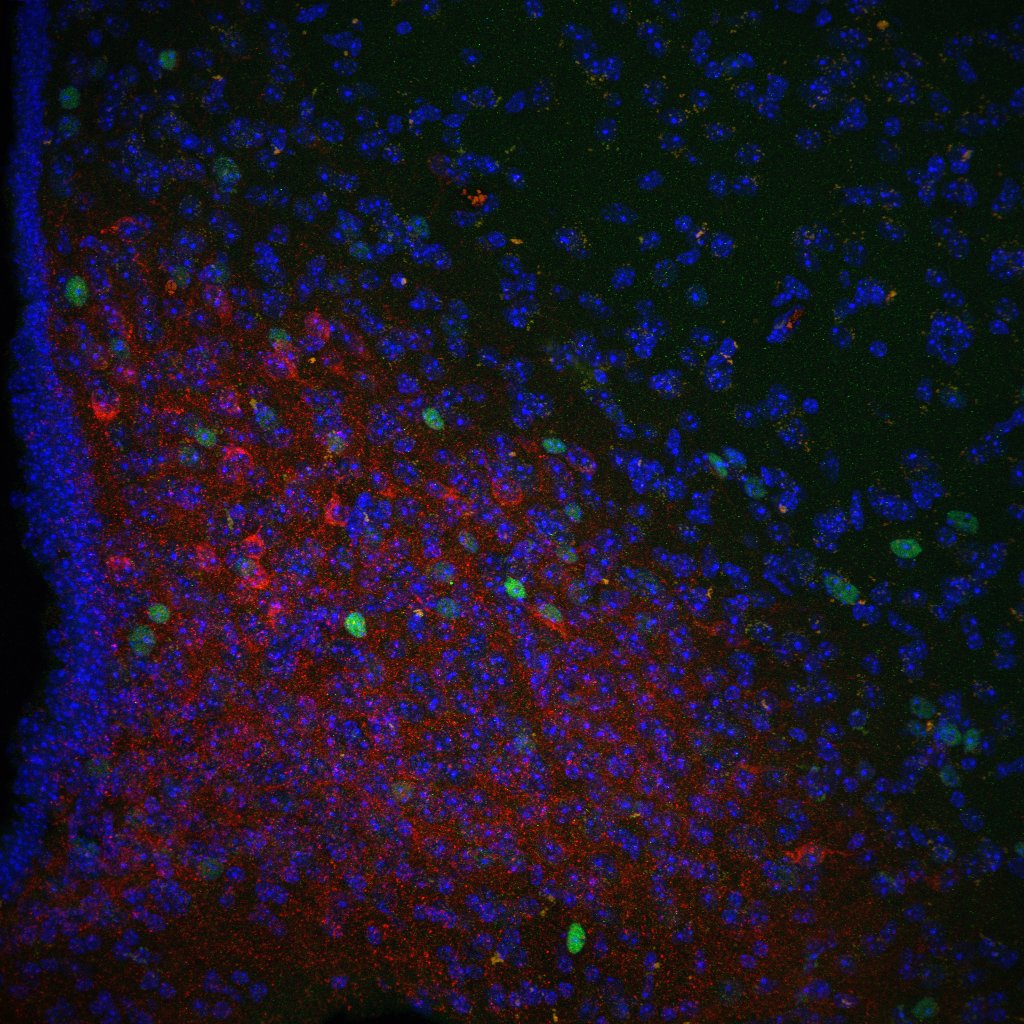

Supplement: Supplementary file 3 — Original pictures of cFOS and Cy5 drug appearance shown in Extended Data Fig. 4a–c, including replicates. [file 42255_2022_617_MOESM3_ESM.zip › Suppl_Fig_4d-replicate3-GLP-1:Tesa-Cy5 ARC.jpg]

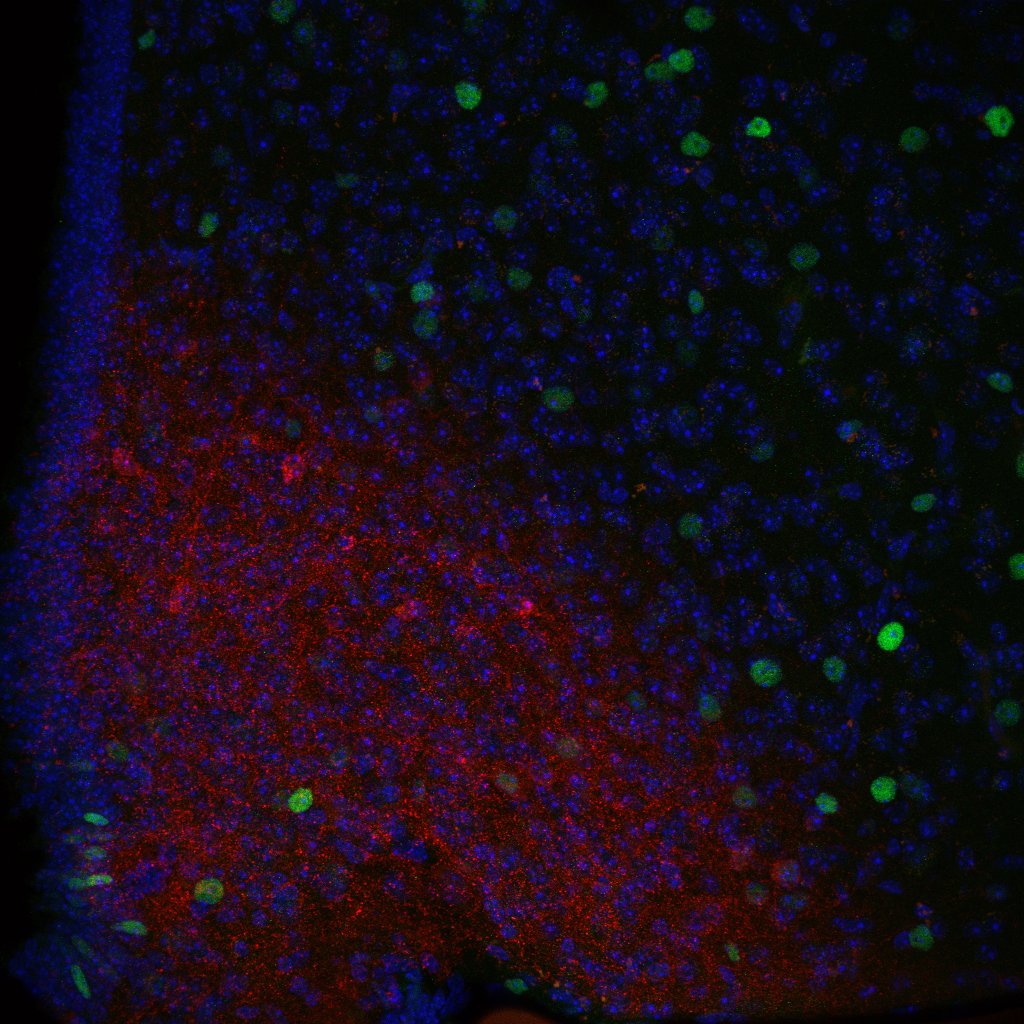

Supplement: Supplementary file 3 — Original pictures of cFOS and Cy5 drug appearance shown in Extended Data Fig. 4a–c, including replicates. [file 42255_2022_617_MOESM3_ESM.zip › Suppl_Fig_4d-replicate3-GLP-1RA-Cy5 ARC.jpg]

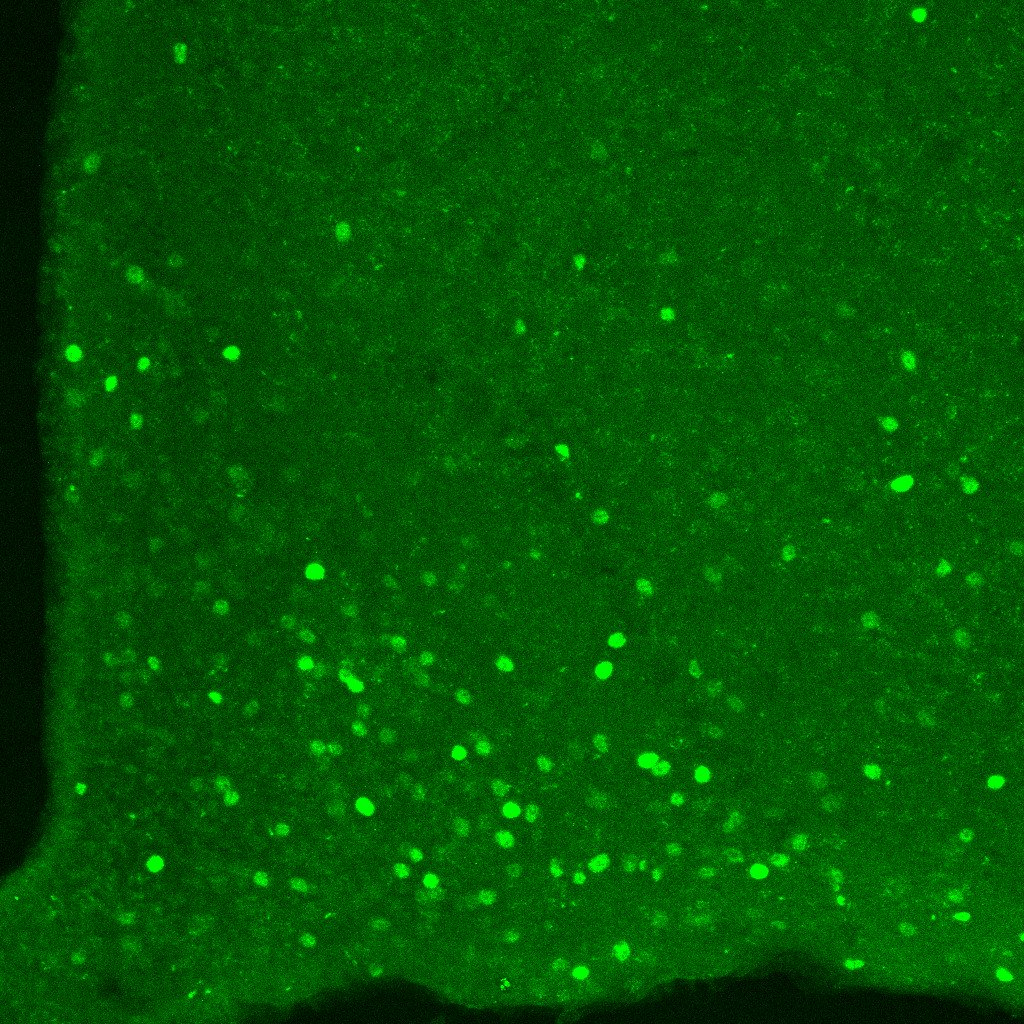

Supplement: Supplementary file 4 — Original pictures of cFOS shown in Fig. 7a,b, including replicates. [file 42255_2022_617_MOESM4_ESM.zip › #1-GLP-1:Tesa_x20_Arc.jpg]

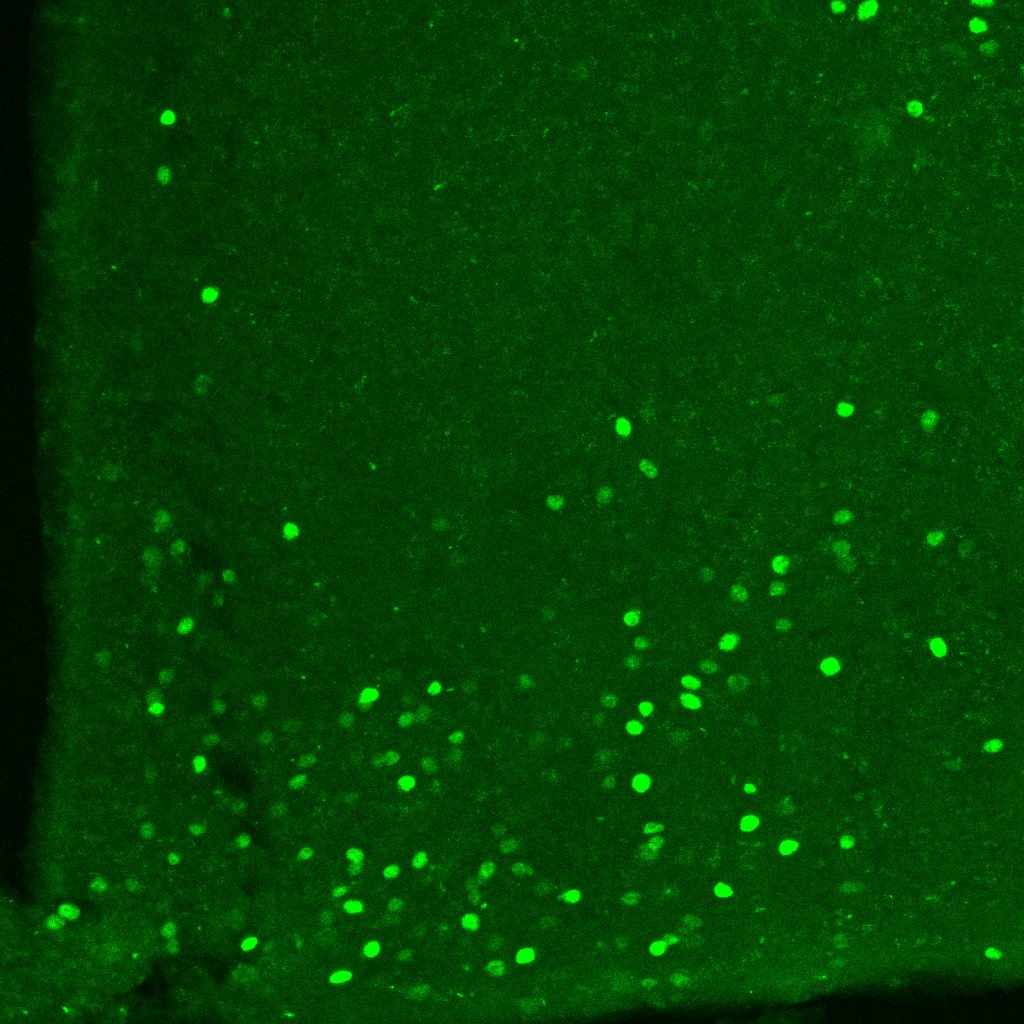

Supplement: Supplementary file 4 — Original pictures of cFOS shown in Fig. 7a,b, including replicates. [file 42255_2022_617_MOESM4_ESM.zip › #1-GLP1RA_x20_Arc.jpg]

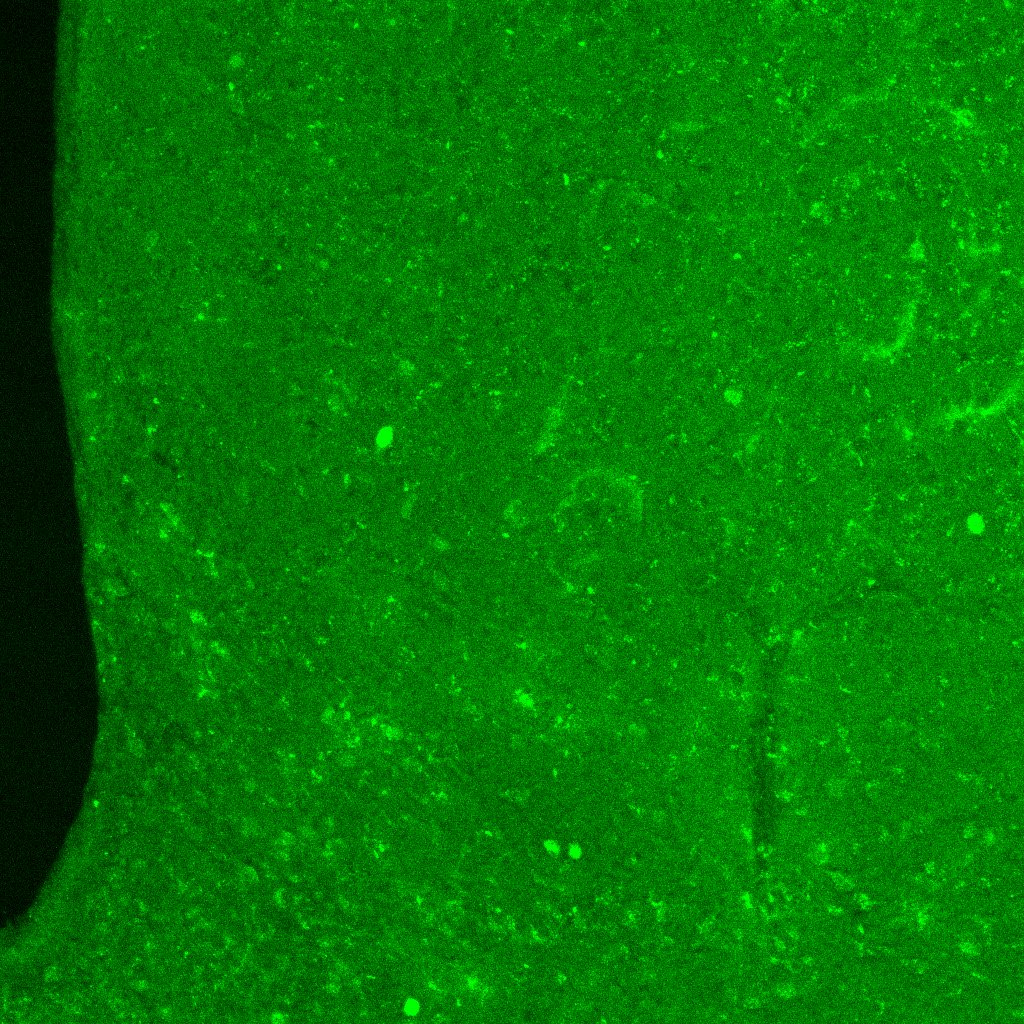

Supplement: Supplementary file 4 — Original pictures of cFOS shown in Fig. 7a,b, including replicates. [file 42255_2022_617_MOESM4_ESM.zip › #1-Tesa_x20_Arc.jpg]

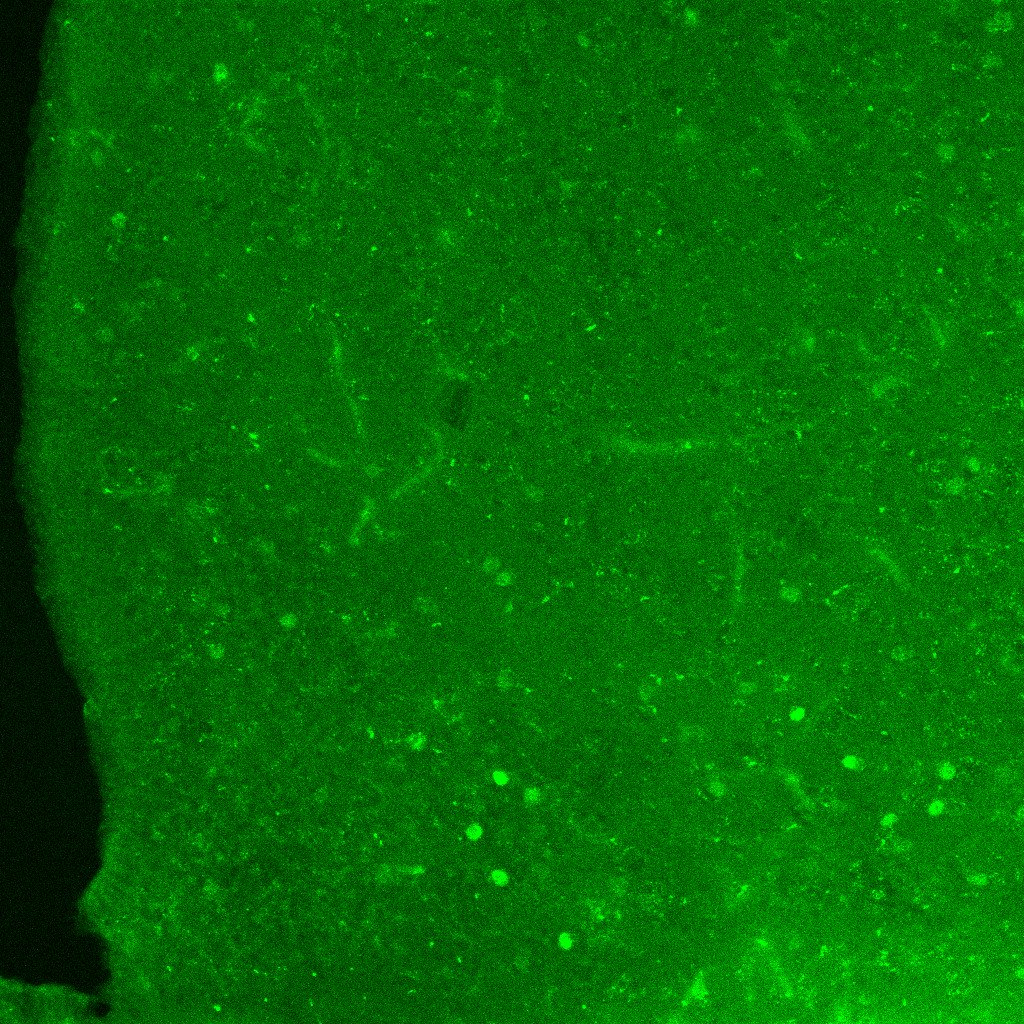

Supplement: Supplementary file 4 — Original pictures of cFOS shown in Fig. 7a,b, including replicates. [file 42255_2022_617_MOESM4_ESM.zip › #1-Vhcl_x20_Arc.jpg]

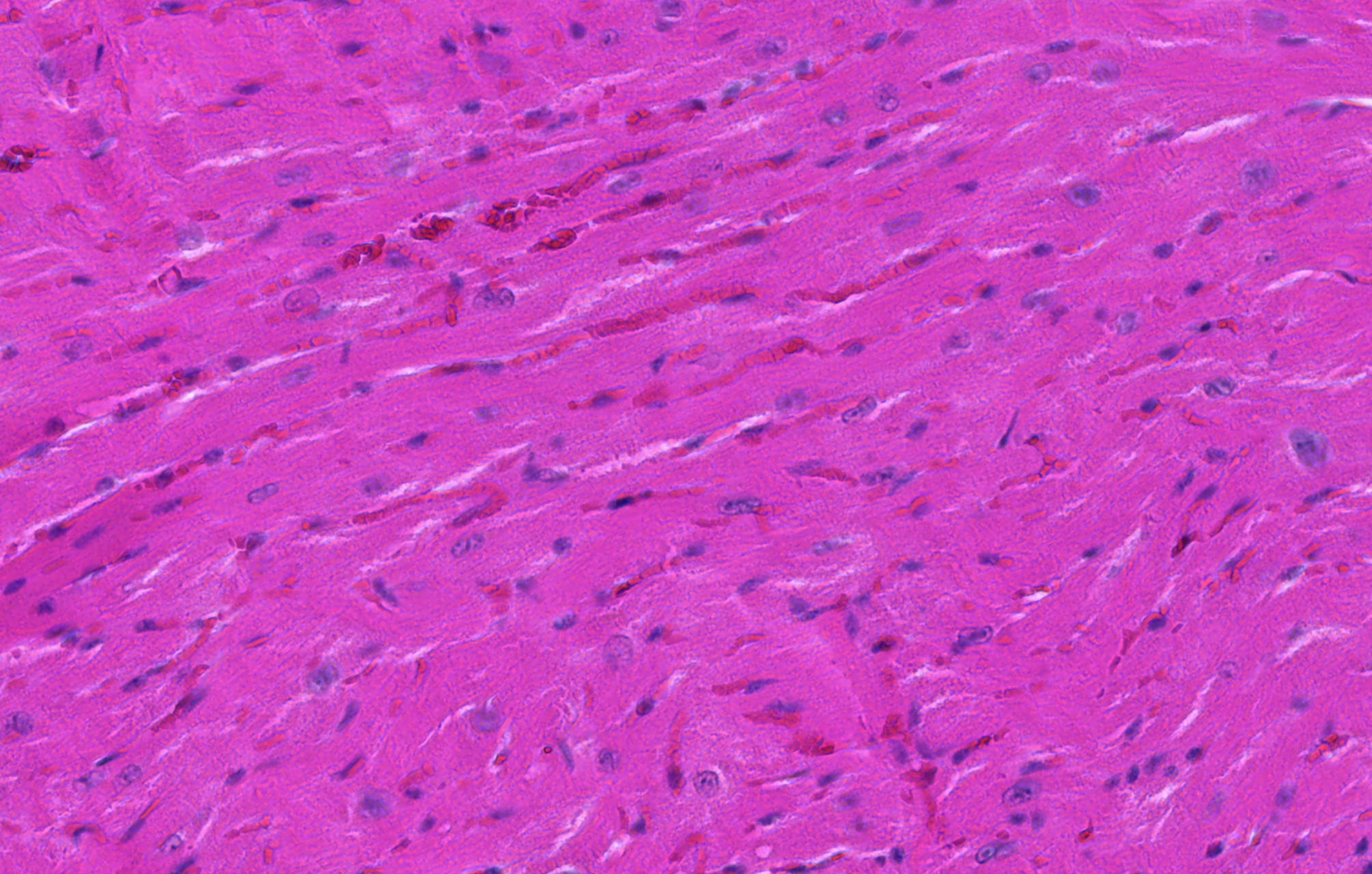

Supplement: Supplementary file 5 — Original histology pictures. [file 42255_2022_617_MOESM5_ESM.zip › Heart_GLP-1RA.TIF]

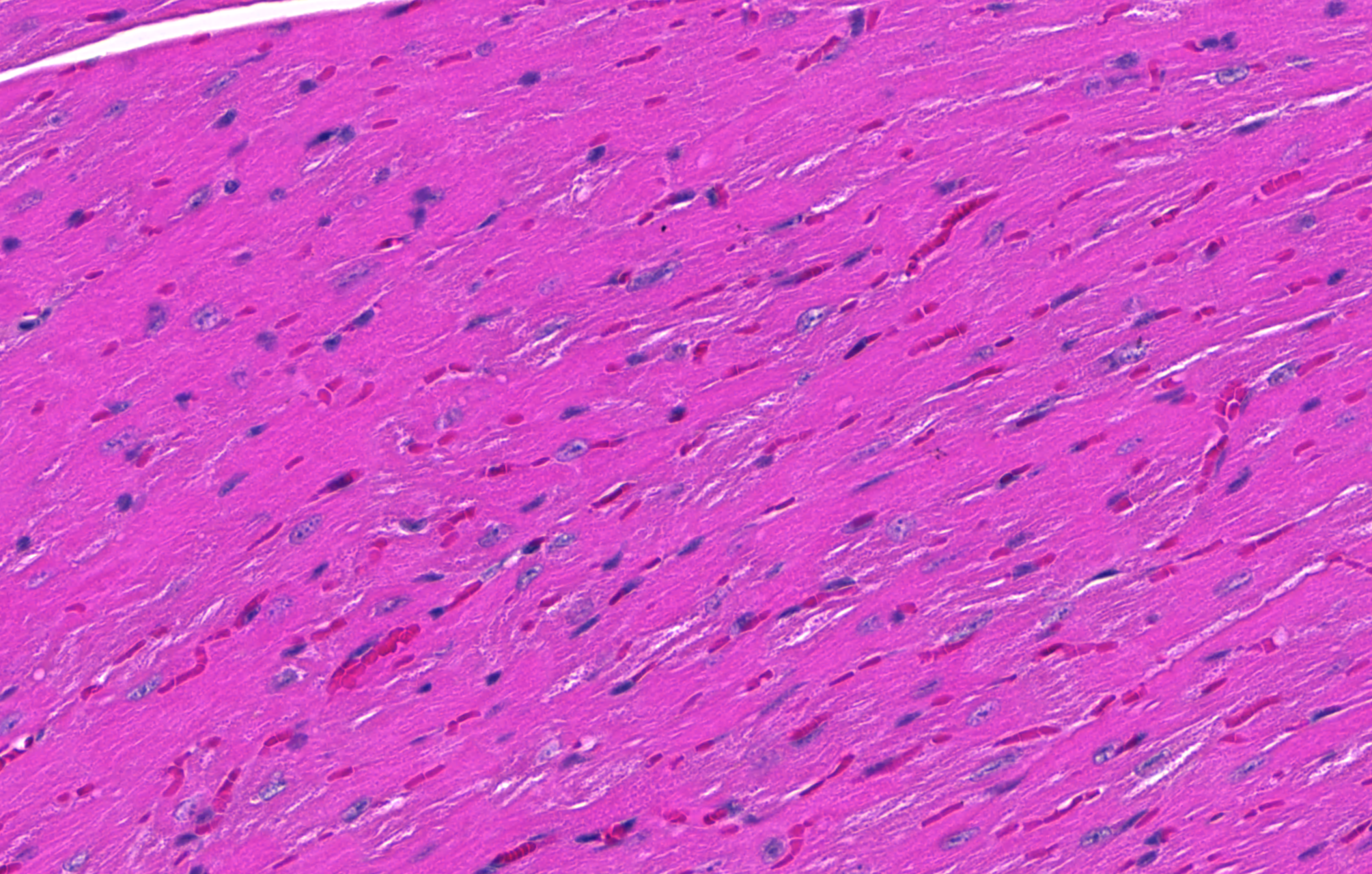

Supplement: Supplementary file 5 — Original histology pictures. [file 42255_2022_617_MOESM5_ESM.zip › Heart_GLP-1RA:Tesaglitazar.TIF]

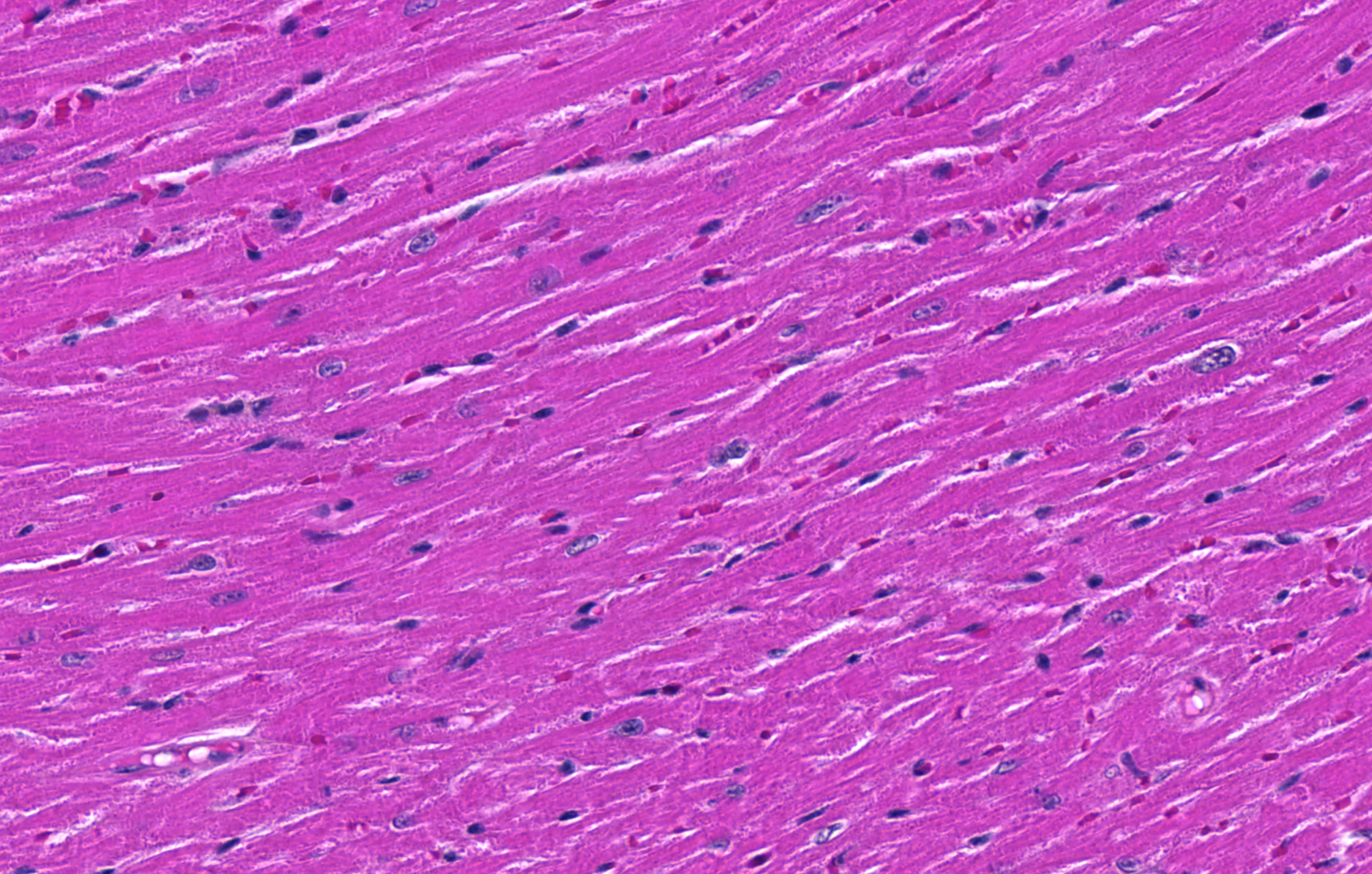

Supplement: Supplementary file 5 — Original histology pictures. [file 42255_2022_617_MOESM5_ESM.zip › Heart_GLP-1RA+Tesaglitazar.TIF]

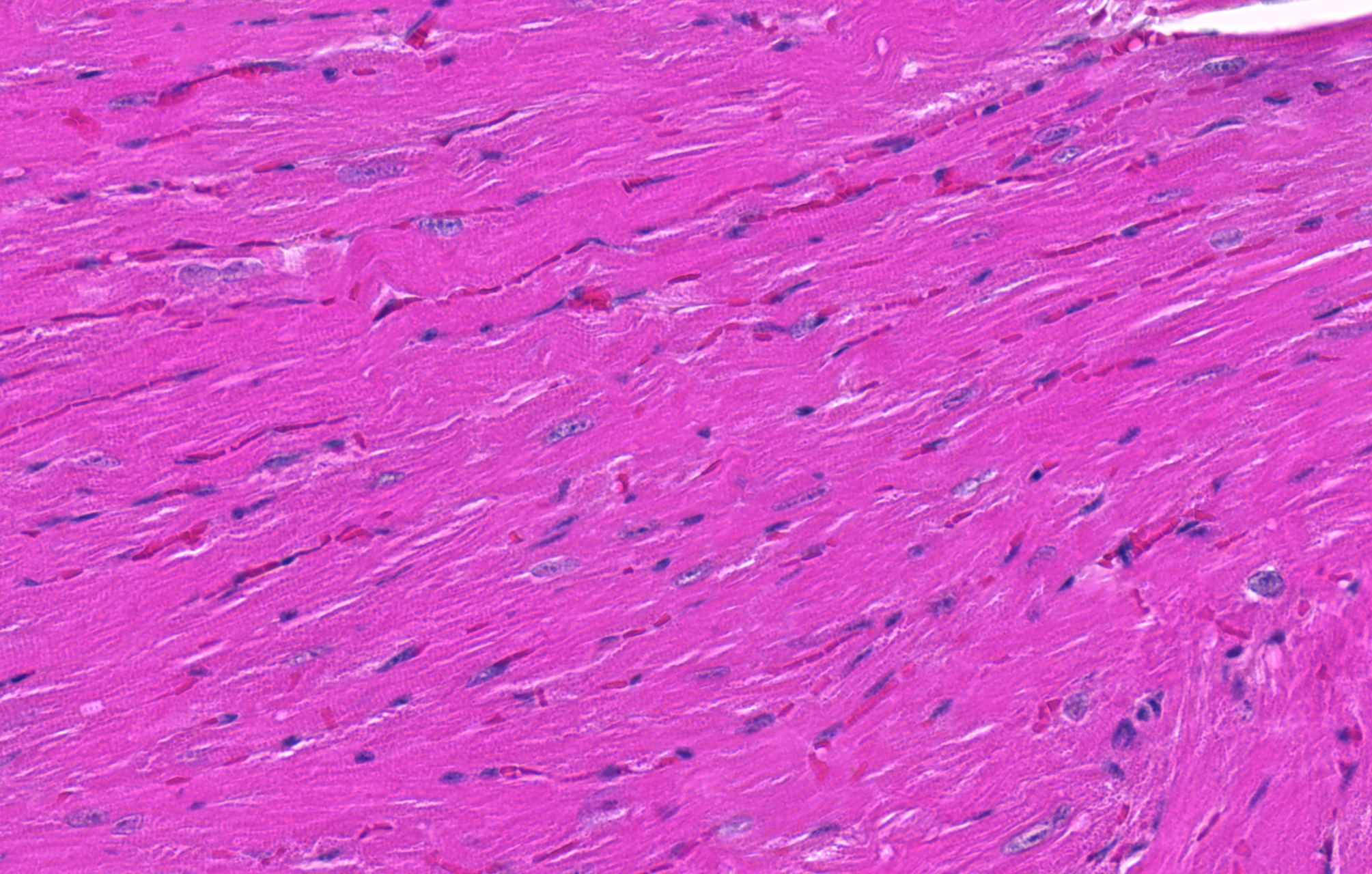

Supplement: Supplementary file 5 — Original histology pictures. [file 42255_2022_617_MOESM5_ESM.zip › Heart_tesaglitazar.TIF]

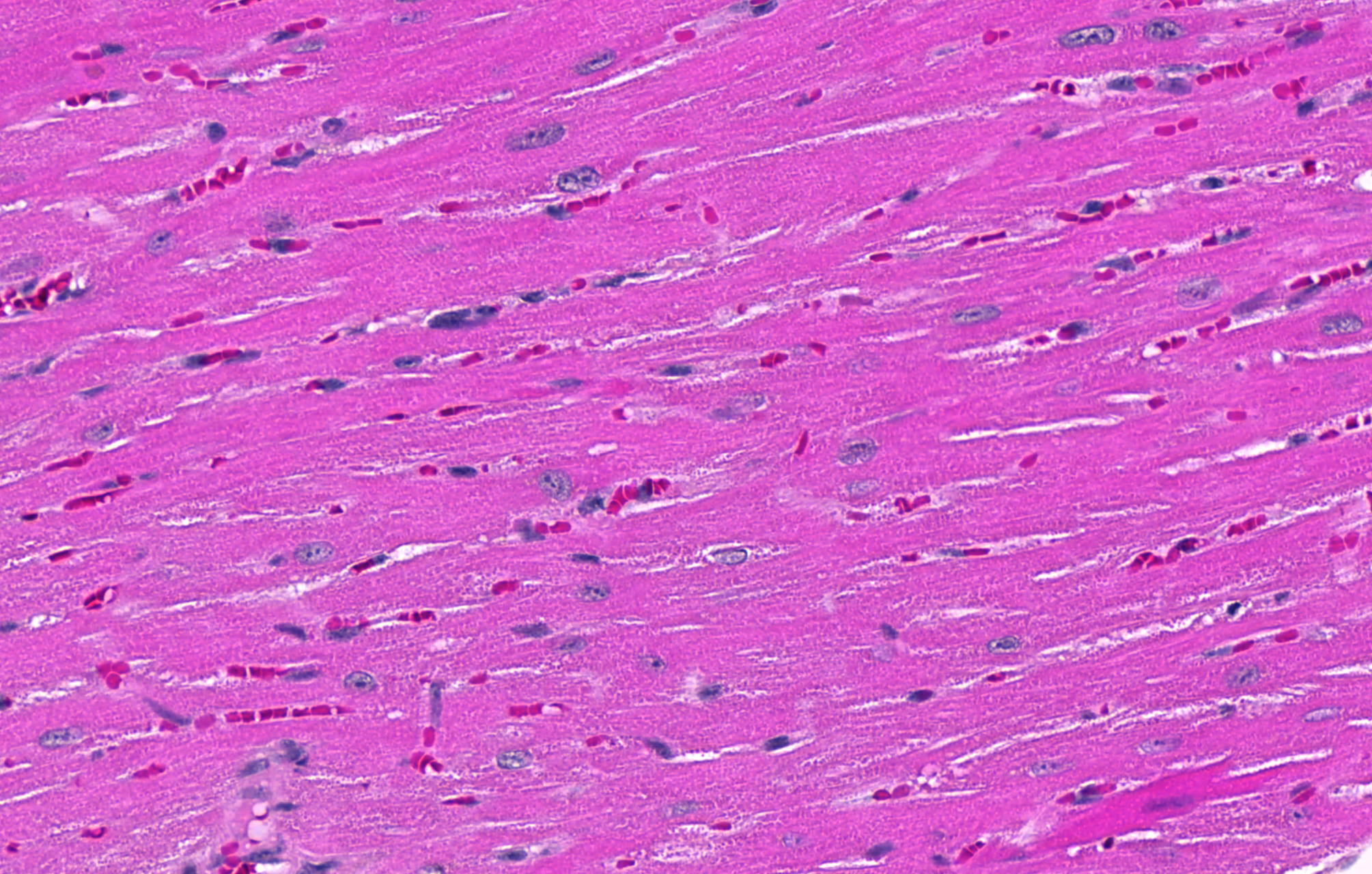

Supplement: Supplementary file 5 — Original histology pictures. [file 42255_2022_617_MOESM5_ESM.zip › Heart_Vhcl.TIF]

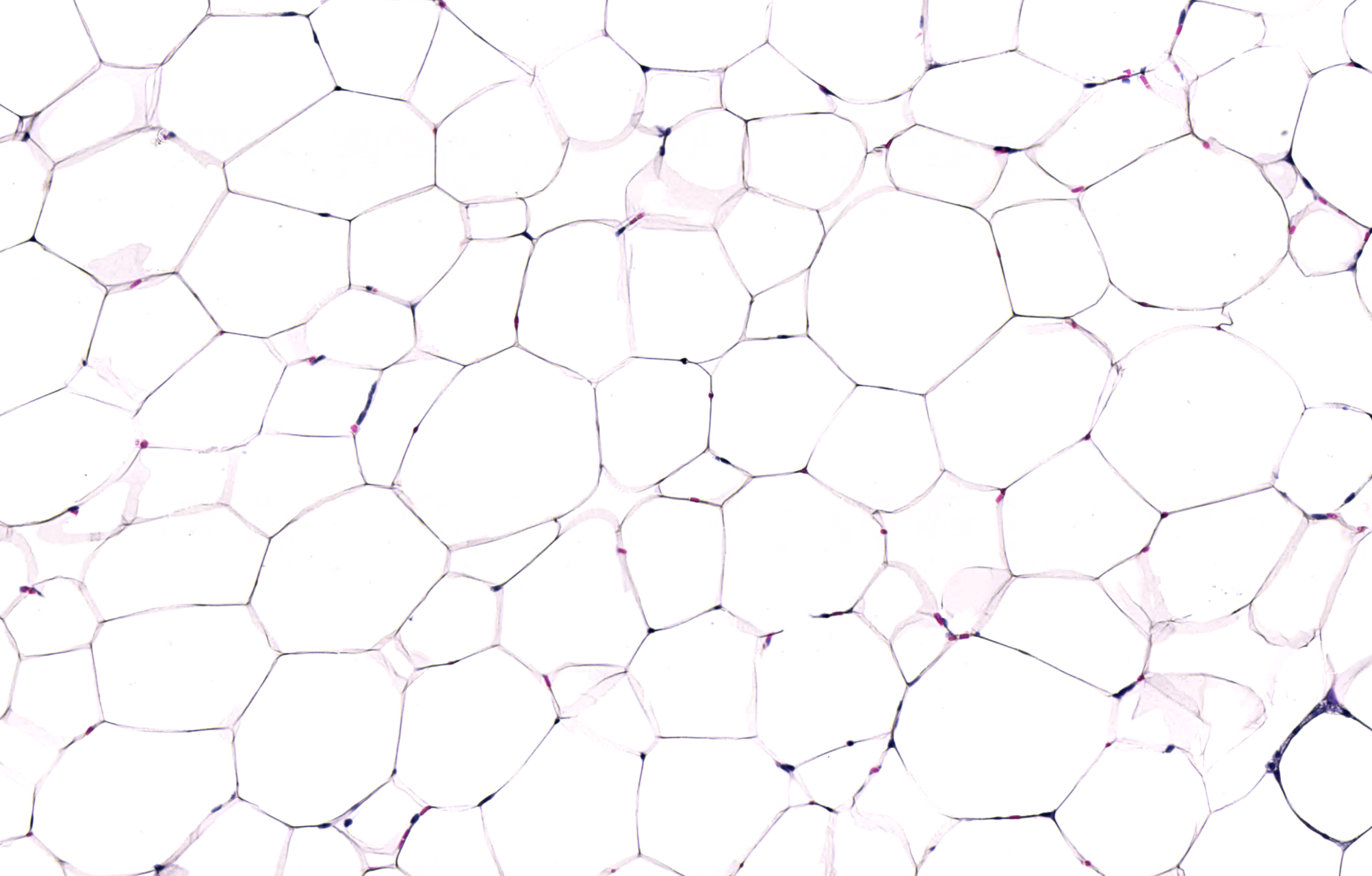

Supplement: Supplementary file 5 — Original histology pictures. [file 42255_2022_617_MOESM5_ESM.zip › iWAT_GLP-1RA.TIF]

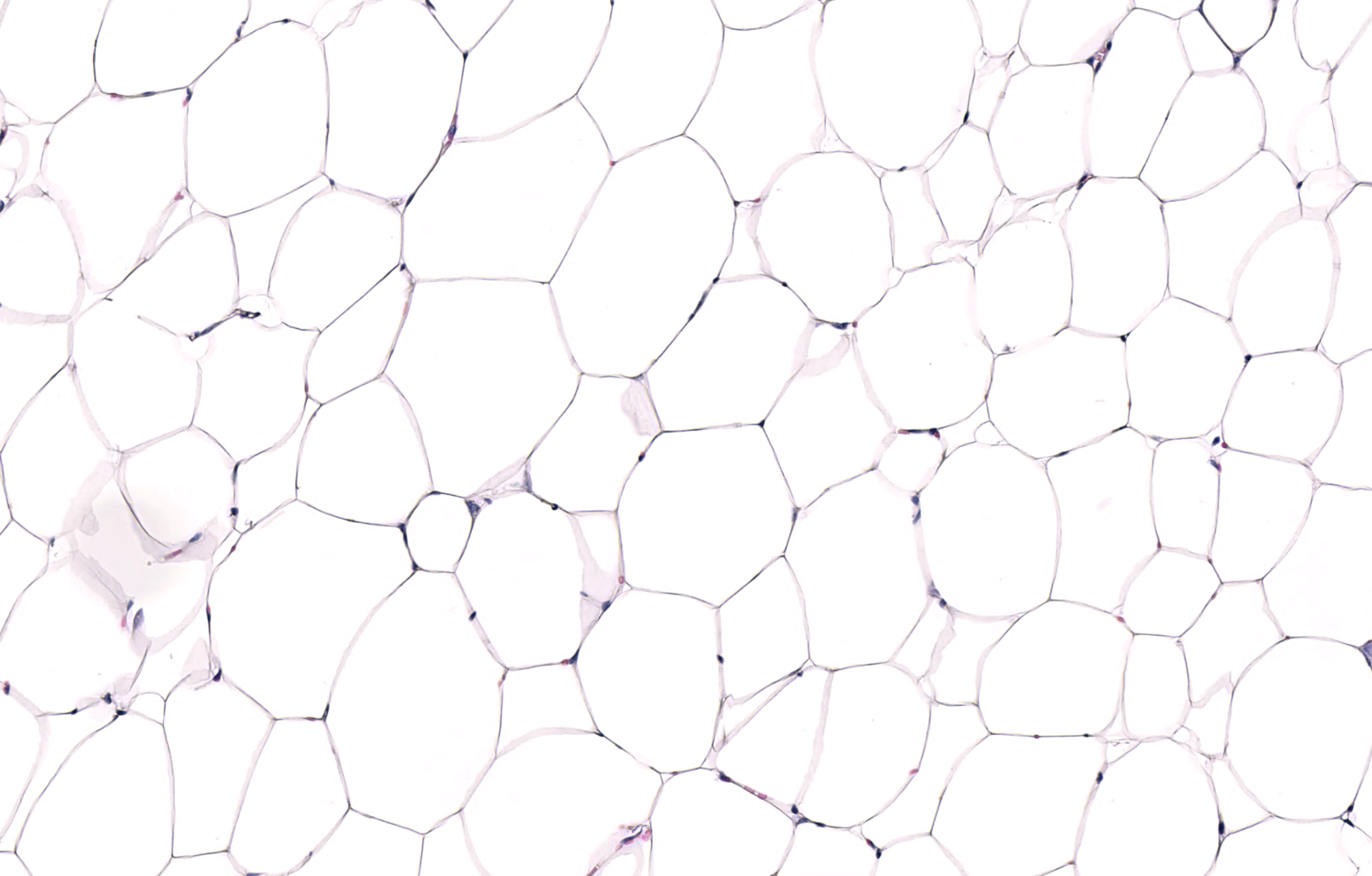

Supplement: Supplementary file 5 — Original histology pictures. [file 42255_2022_617_MOESM5_ESM.zip › iWAT_GLP-1RA:Tesaglitazar.TIF]

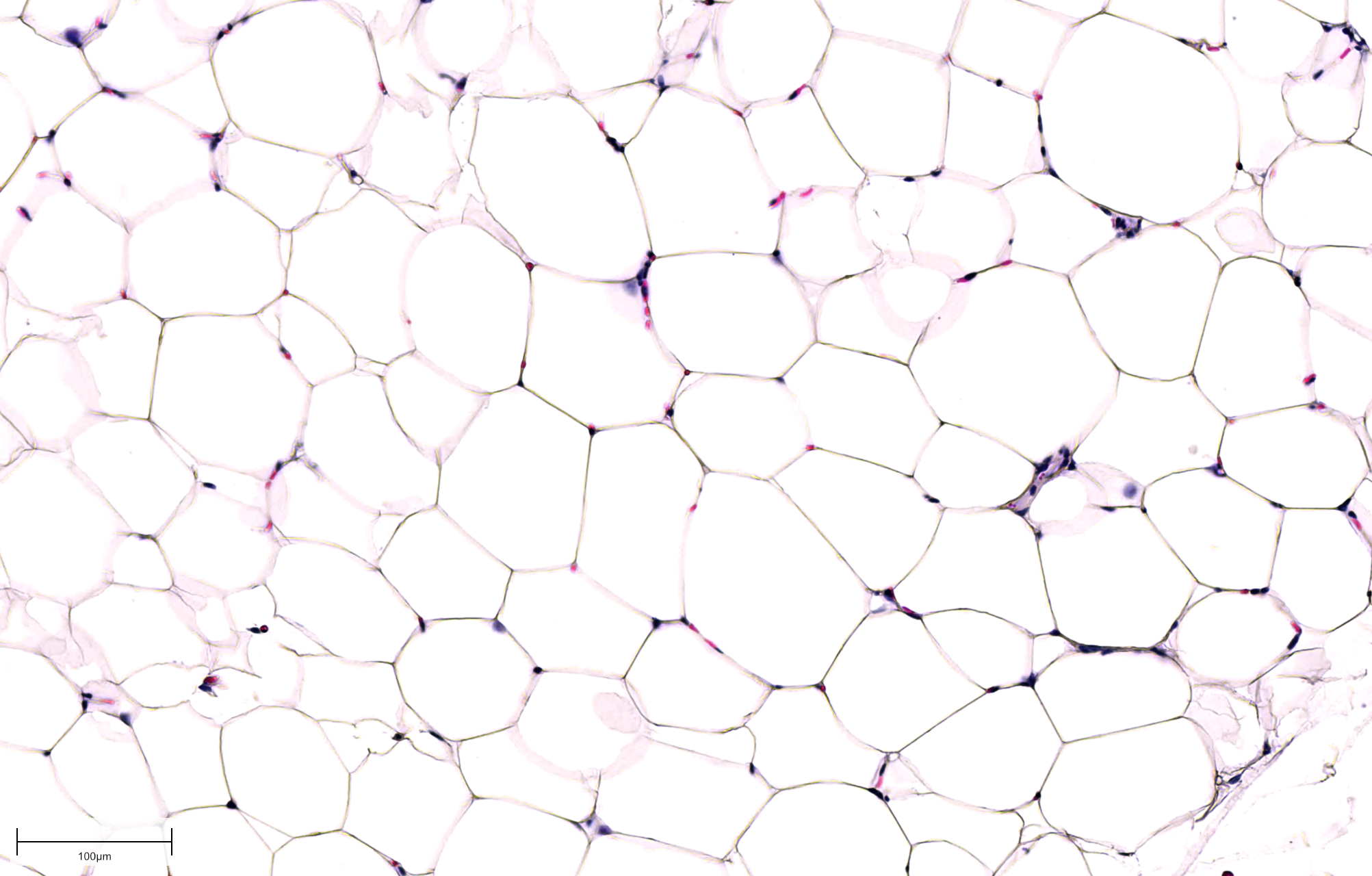

Supplement: Supplementary file 5 — Original histology pictures. [file 42255_2022_617_MOESM5_ESM.zip › iWAT_GLP-1RA+Tesaglitazar.TIF]

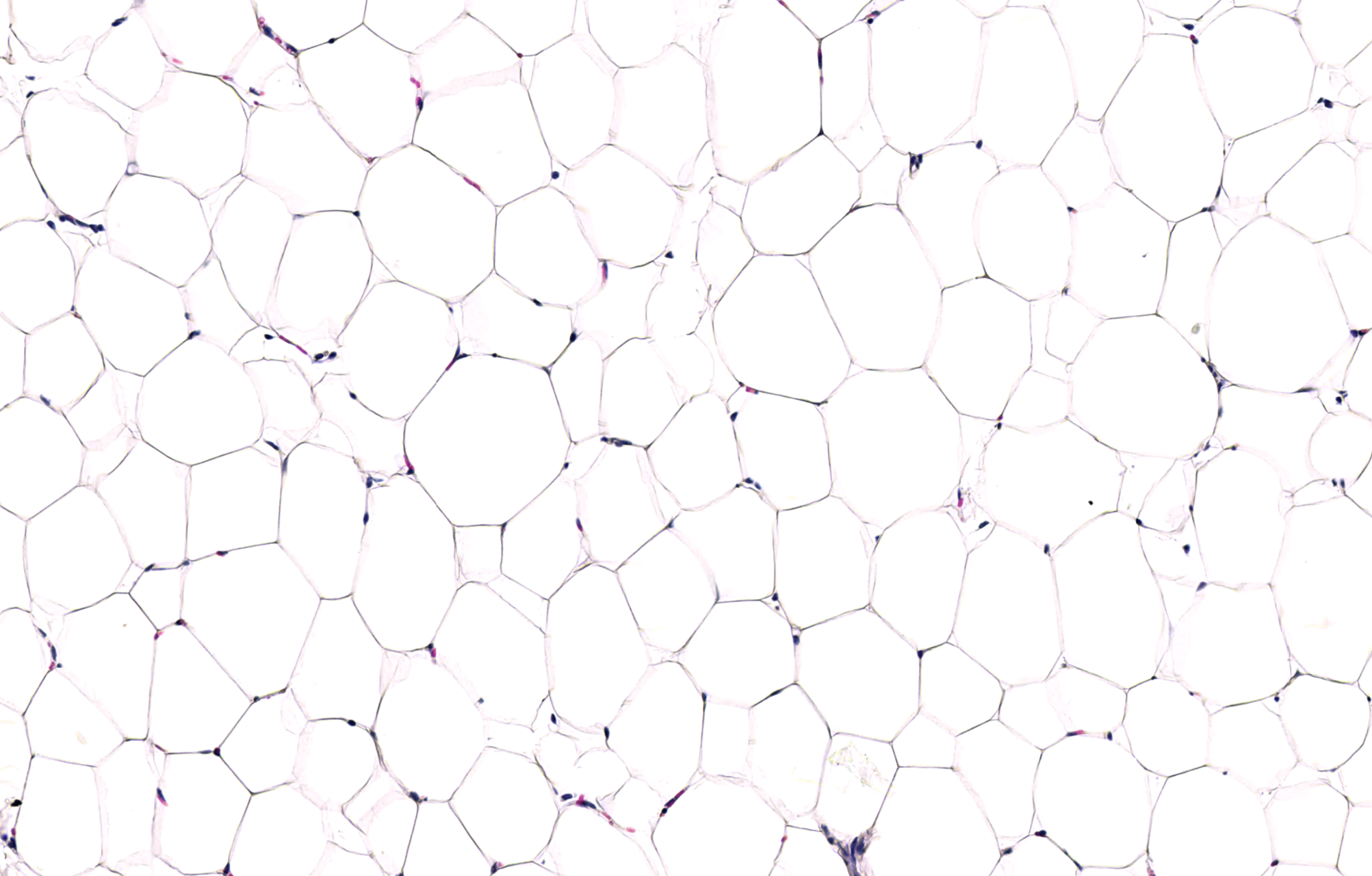

Supplement: Supplementary file 5 — Original histology pictures. [file 42255_2022_617_MOESM5_ESM.zip › iWAT_Tesaglitazar.TIF]

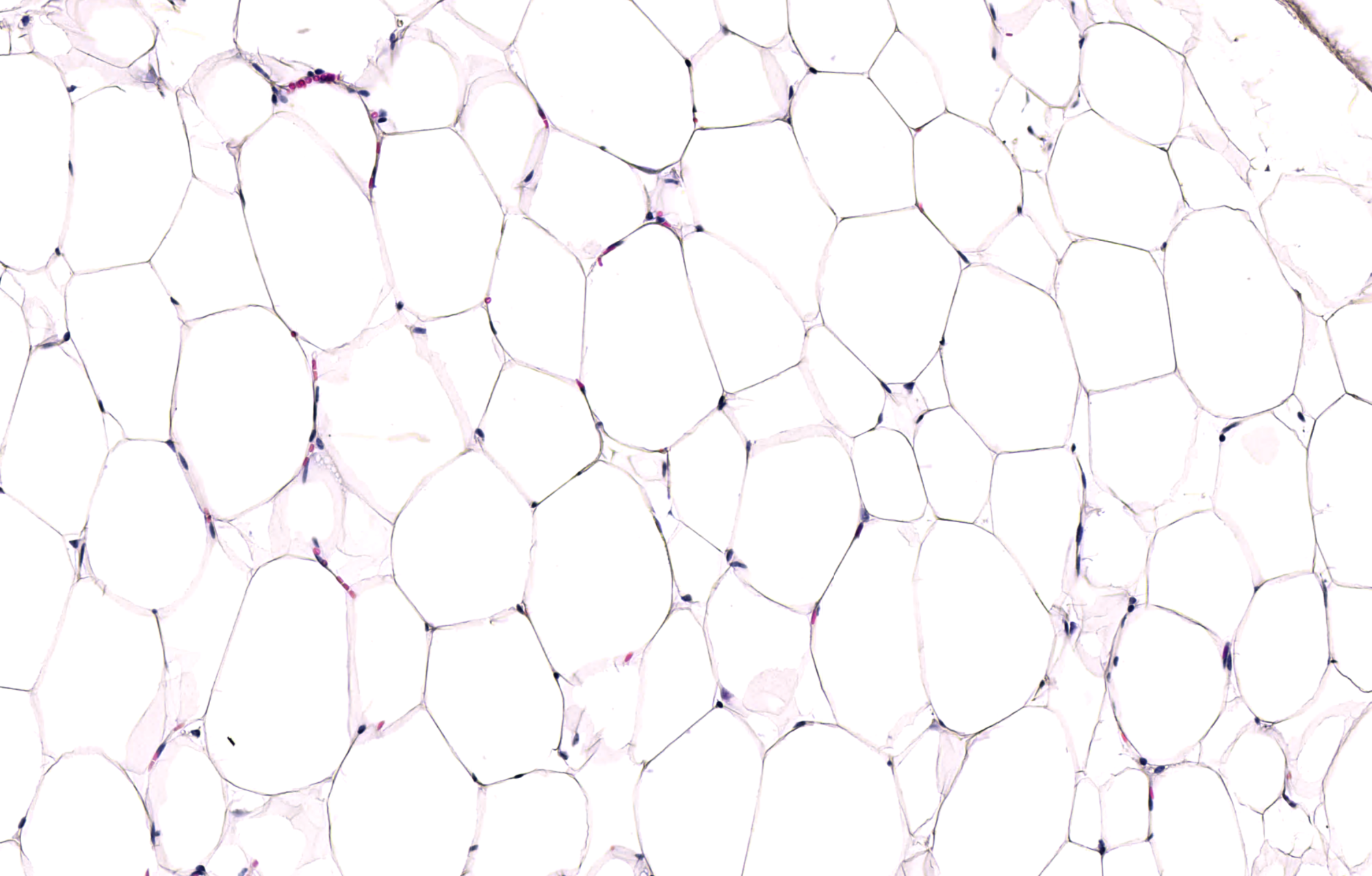

Supplement: Supplementary file 5 — Original histology pictures. [file 42255_2022_617_MOESM5_ESM.zip › iWAT_Vhcl.TIF]

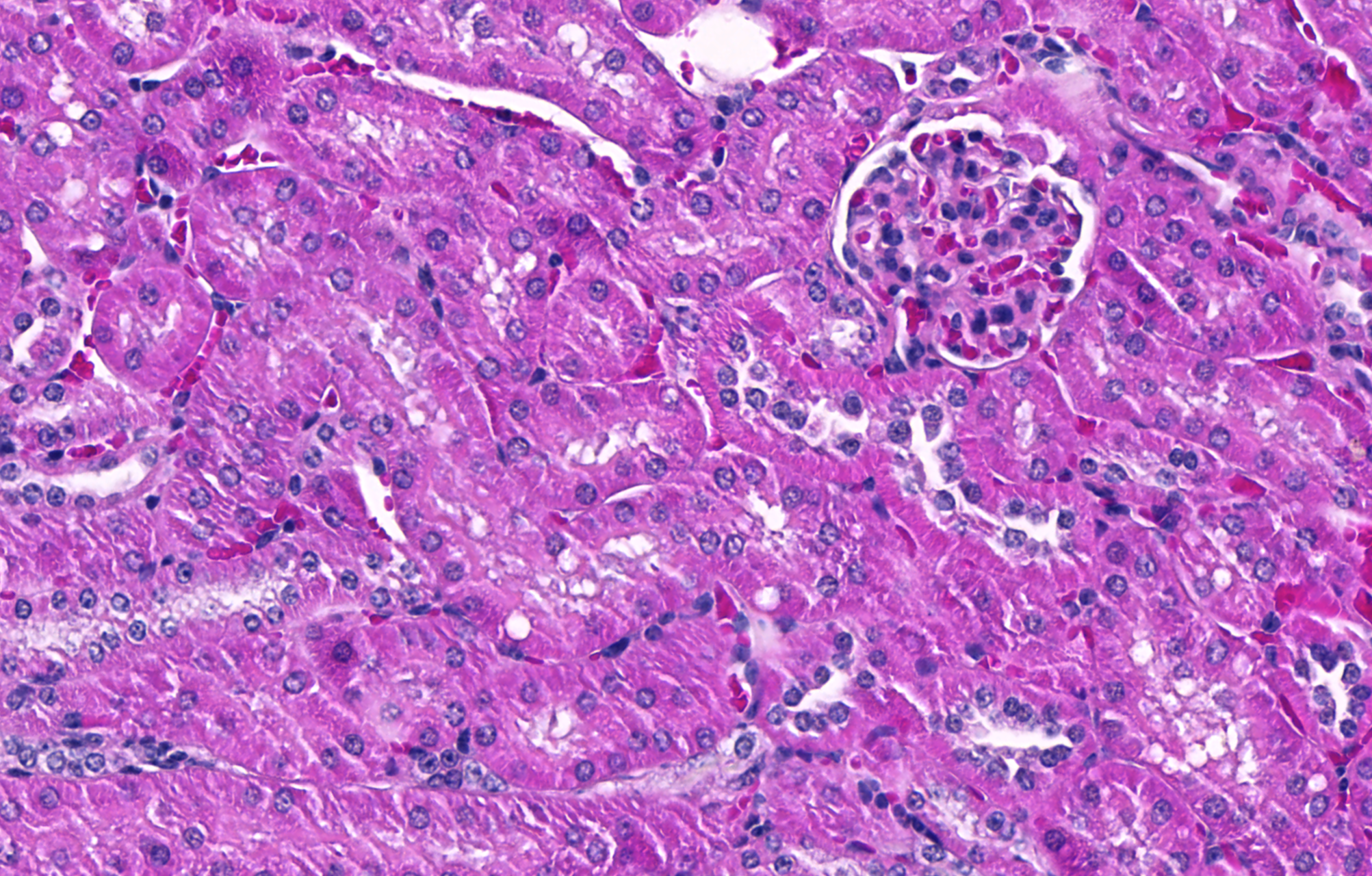

Supplement: Supplementary file 5 — Original histology pictures. [file 42255_2022_617_MOESM5_ESM.zip › Kidney_GLP-1RA_20x.TIF]

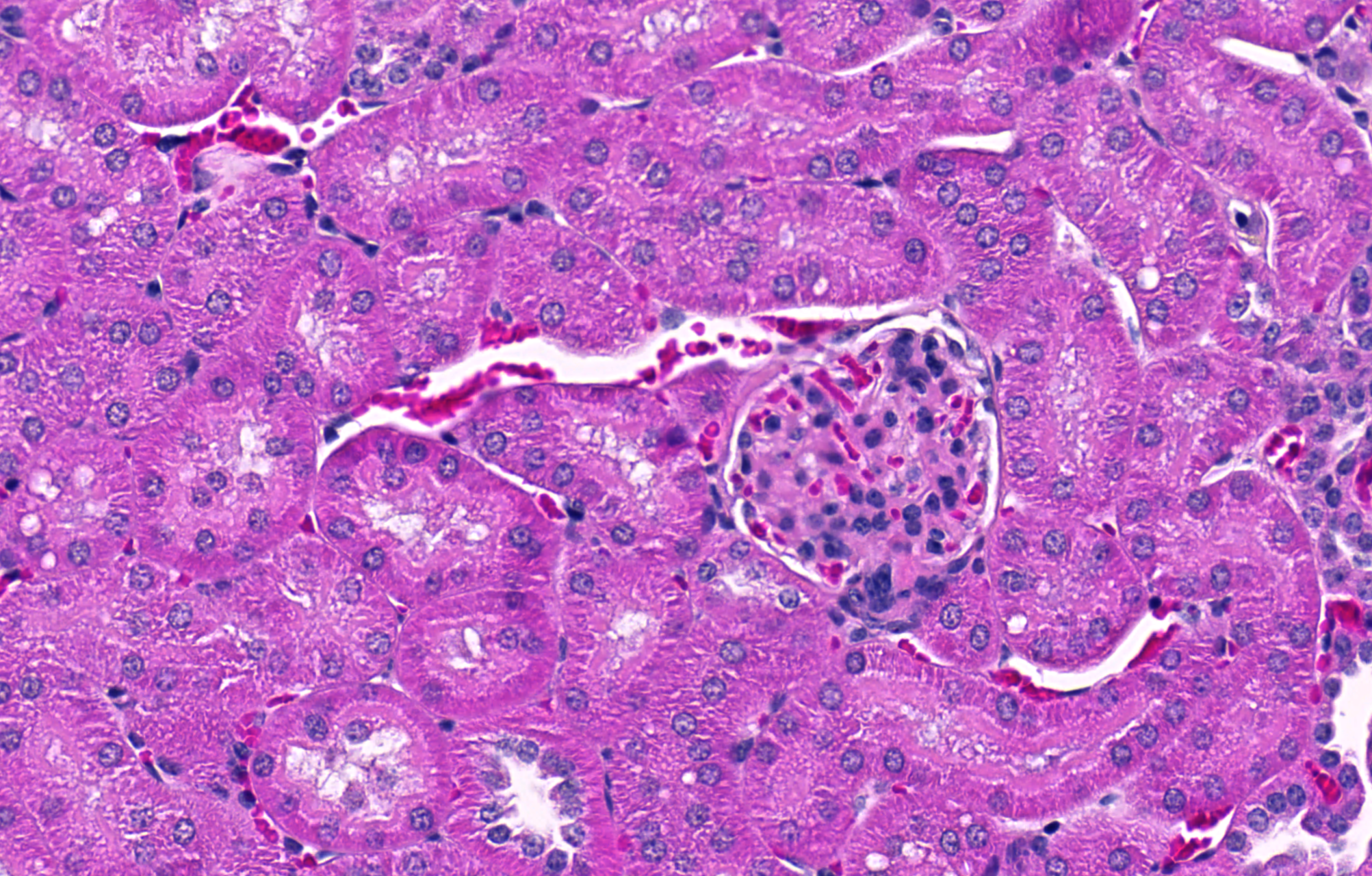

Supplement: Supplementary file 5 — Original histology pictures. [file 42255_2022_617_MOESM5_ESM.zip › Kidney_GLP-1RA:Tesaglitazar_20x.TIF]

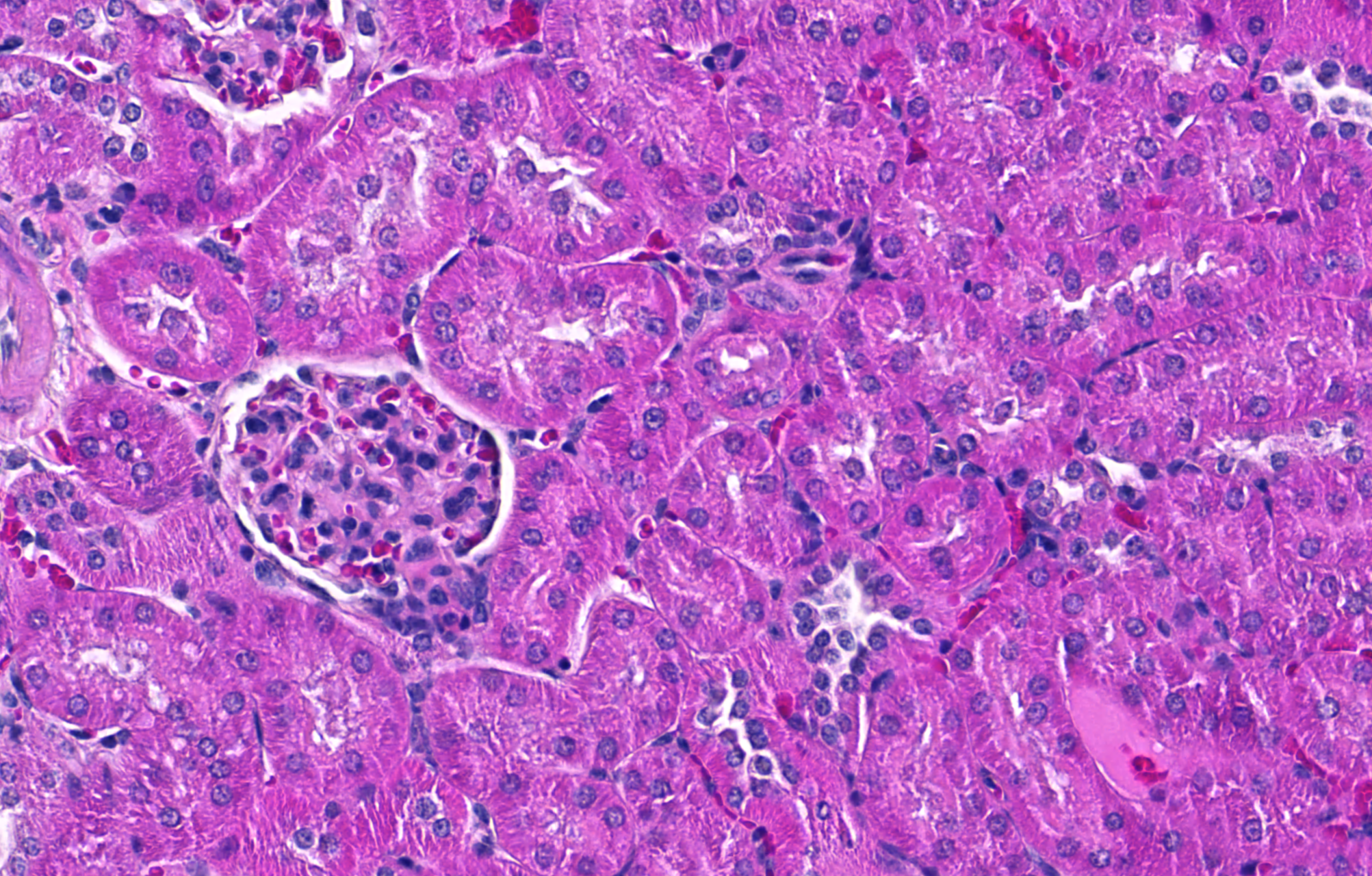

Supplement: Supplementary file 5 — Original histology pictures. [file 42255_2022_617_MOESM5_ESM.zip › Kidney_GLP-1RA+Tesaglitazar_20x.TIF]

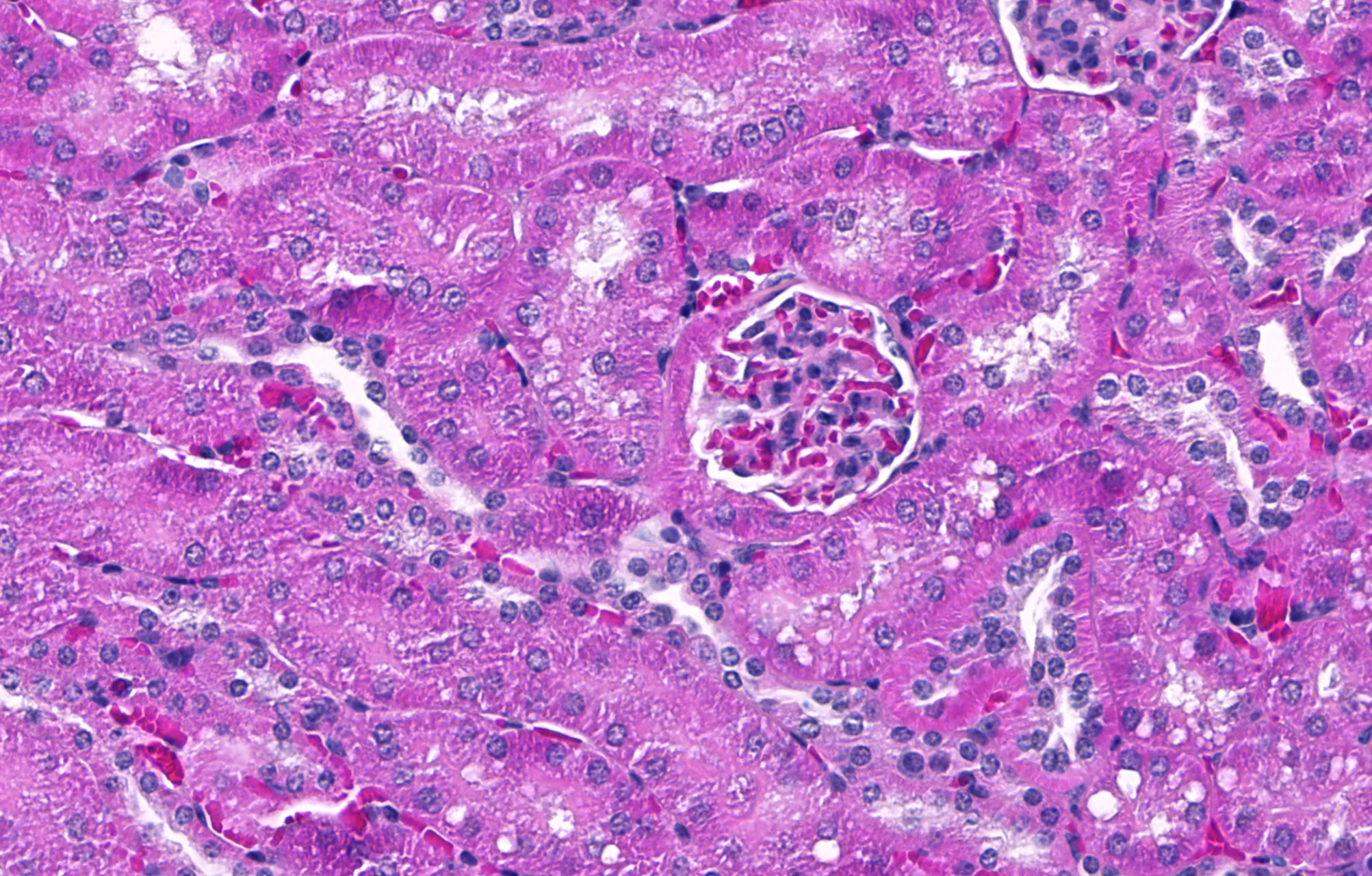

Supplement: Supplementary file 5 — Original histology pictures. [file 42255_2022_617_MOESM5_ESM.zip › Kidney_Tesaglitazar_20x.TIF]

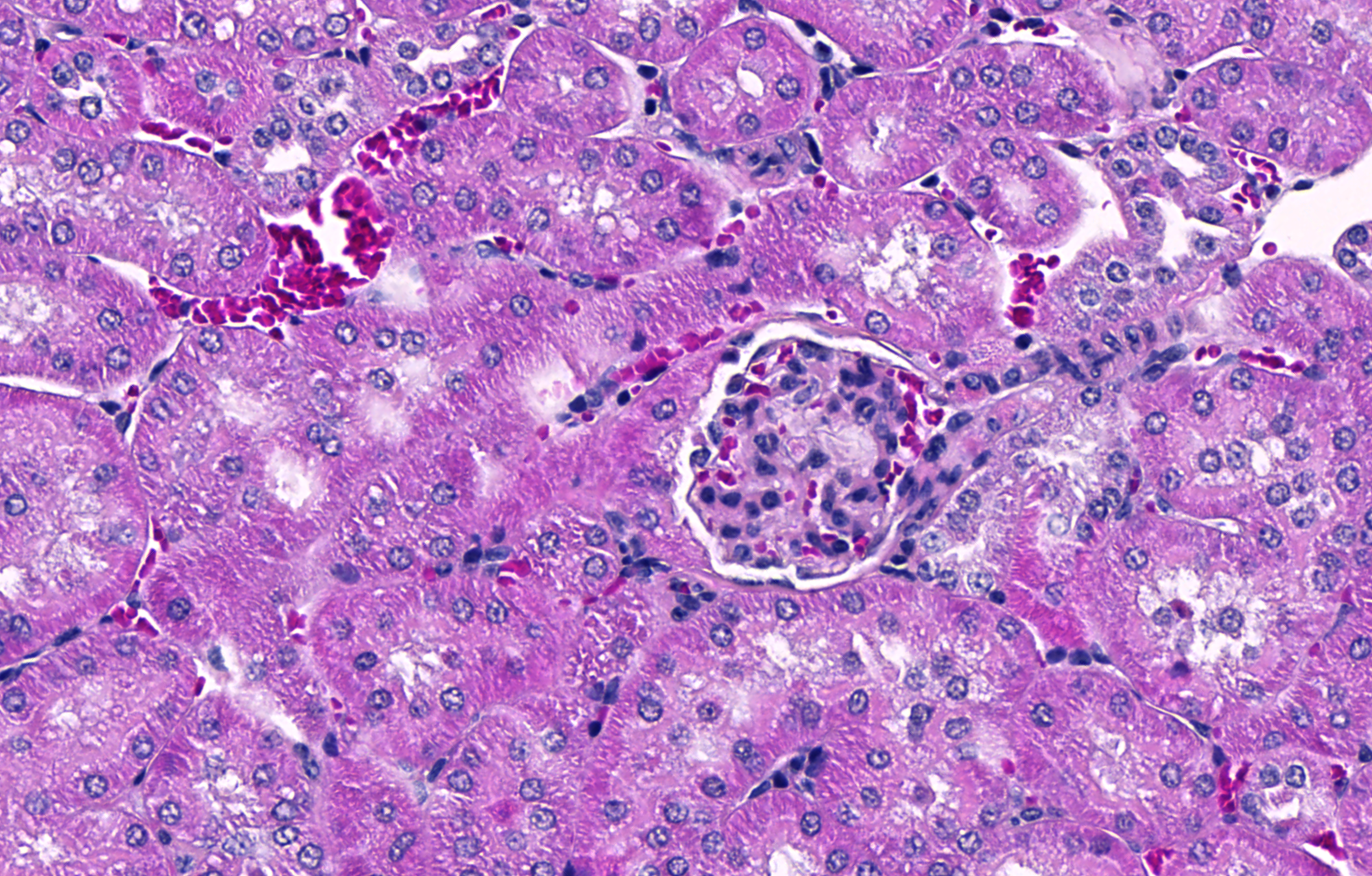

Supplement: Supplementary file 5 — Original histology pictures. [file 42255_2022_617_MOESM5_ESM.zip › Kidney_Vhcl_20x.TIF]

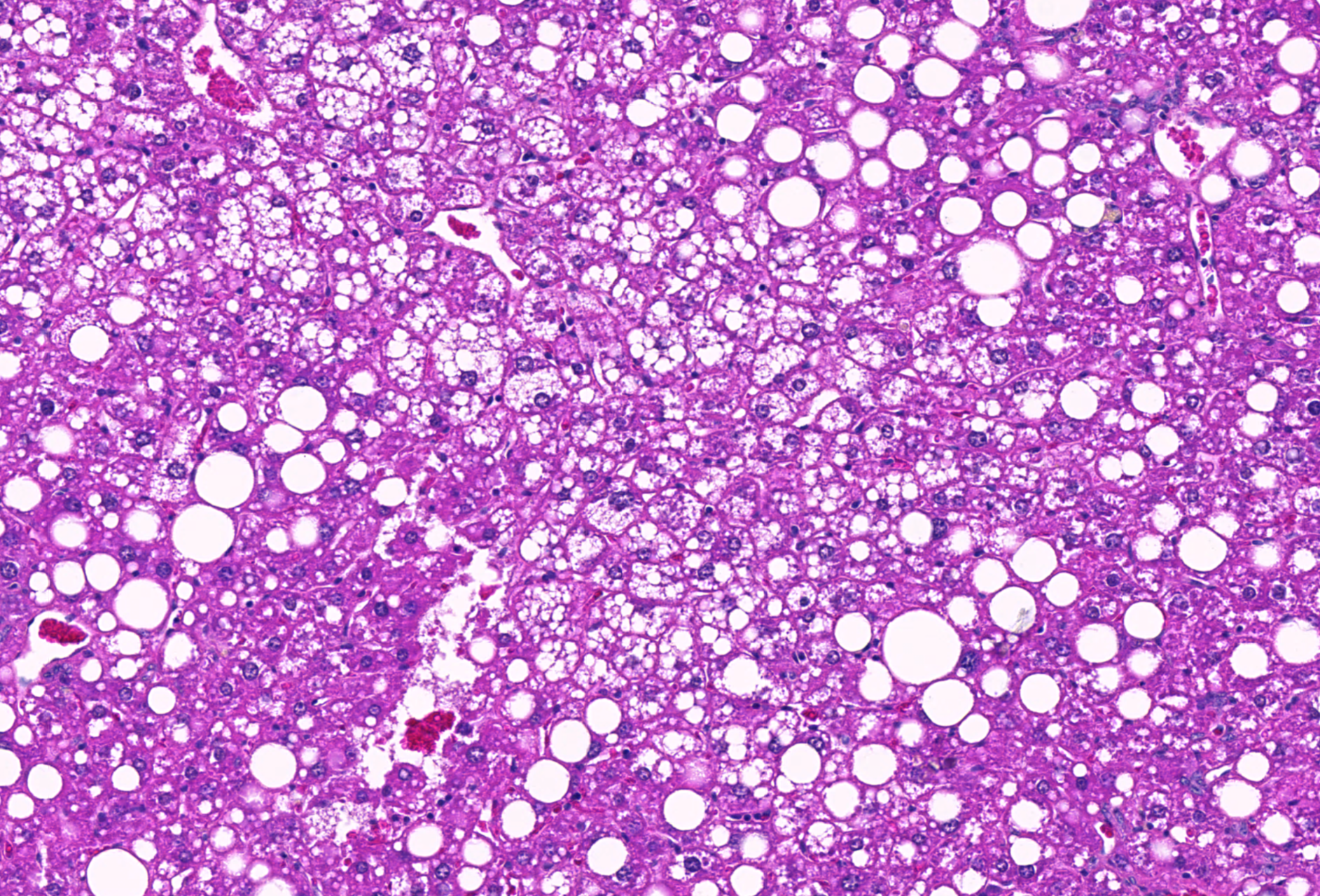

Supplement: Supplementary file 5 — Original histology pictures. [file 42255_2022_617_MOESM5_ESM.zip › Liver_GLP-1RA.TIF]

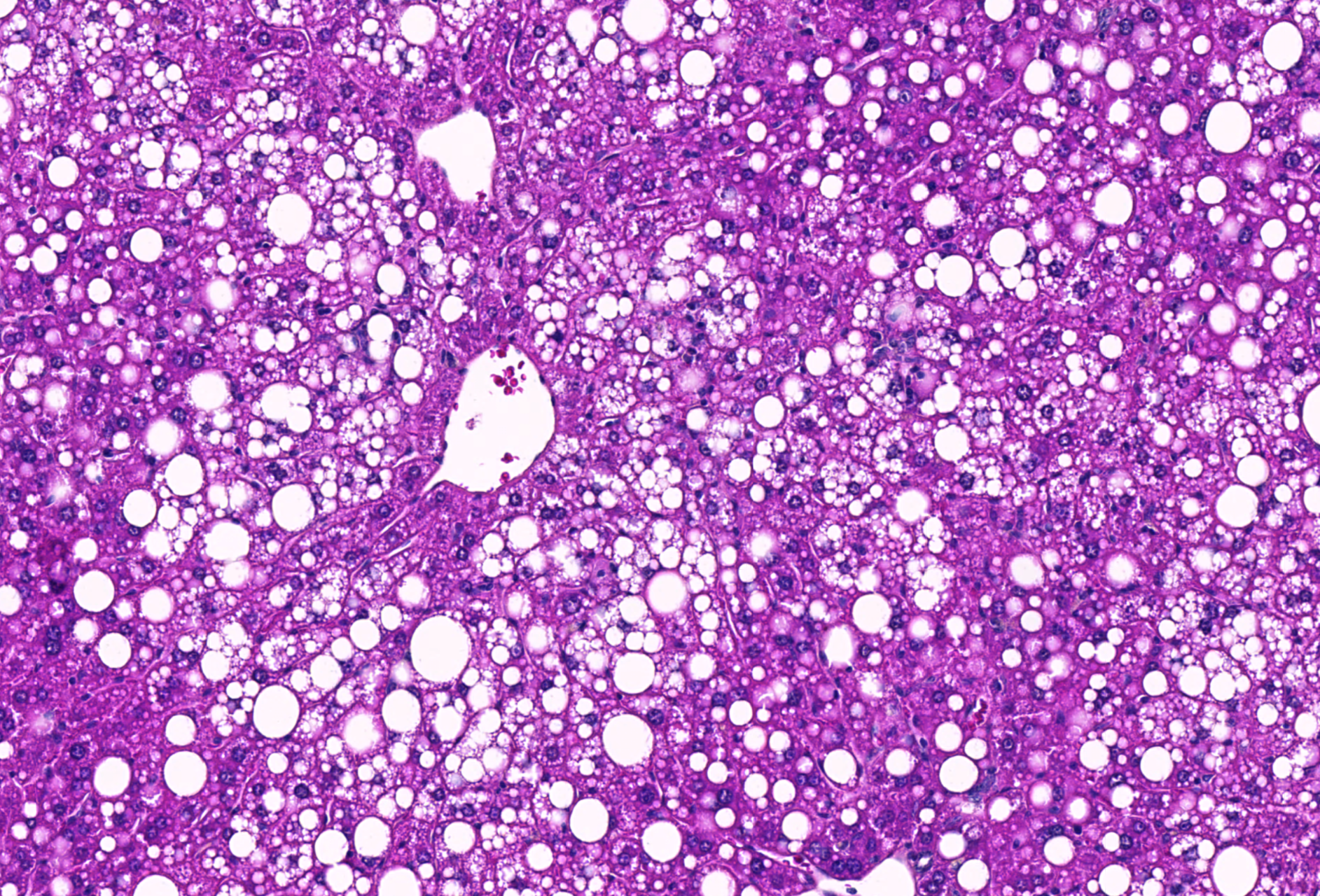

Supplement: Supplementary file 5 — Original histology pictures. [file 42255_2022_617_MOESM5_ESM.zip › Liver_GLP-1RA:Tesaglitazar.TIF]
