# Supplementary figures and images for: GLP-1-mediated delivery of tesaglitazar improves obesity and glucose metabolism in male mice (part 2 of 2)
Source: Nat Metab. 2022 Aug 22;4(8):1071–83. doi: 10.1038/s42255-022-00617-6 (PMC9398908; doi:10.1038/s42255-022-00617-6)

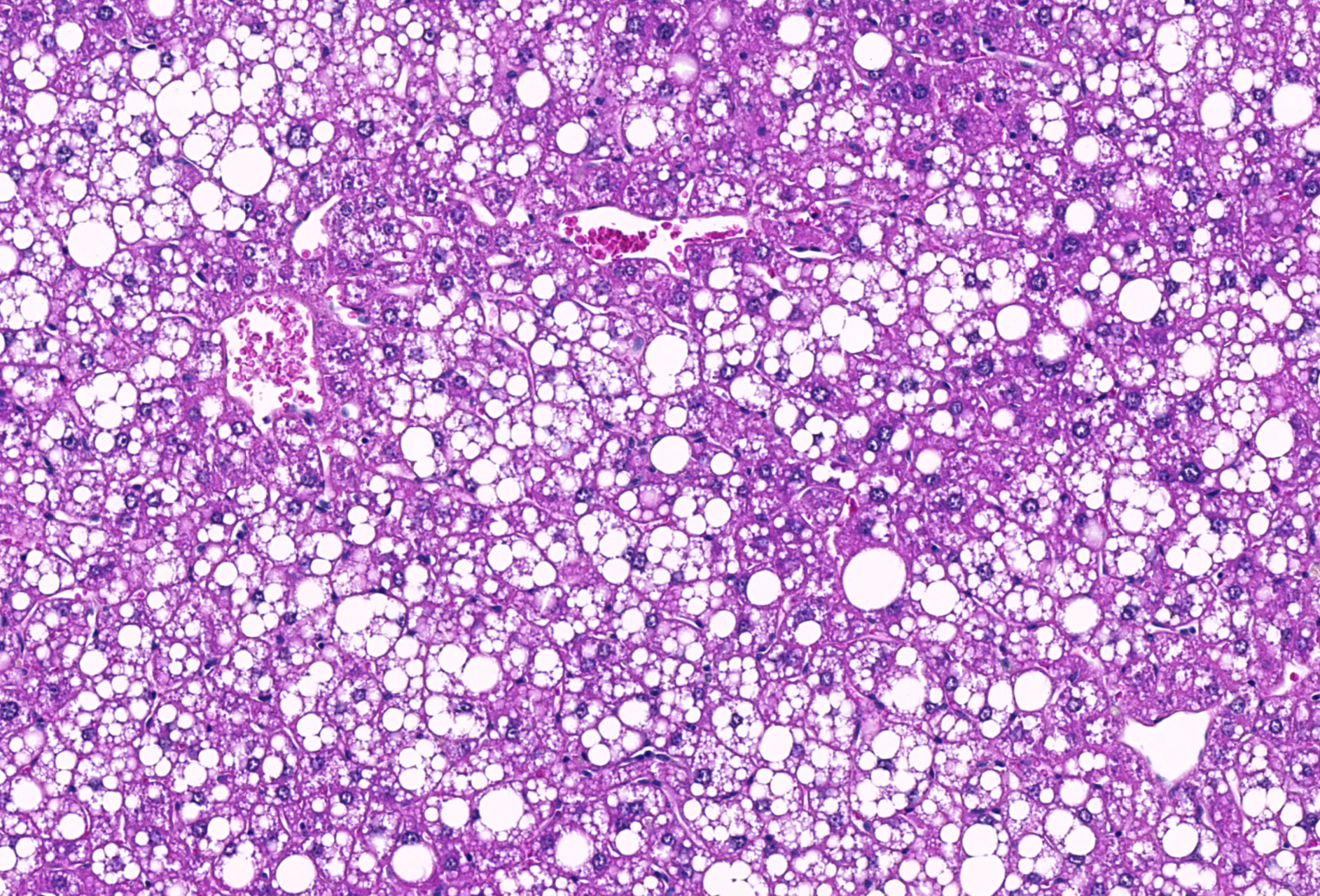

Supplement: Supplementary file 5 — Original histology pictures. [file 42255_2022_617_MOESM5_ESM.zip › Liver_GLP-1RA+TesaglitazarTIF.TIF]

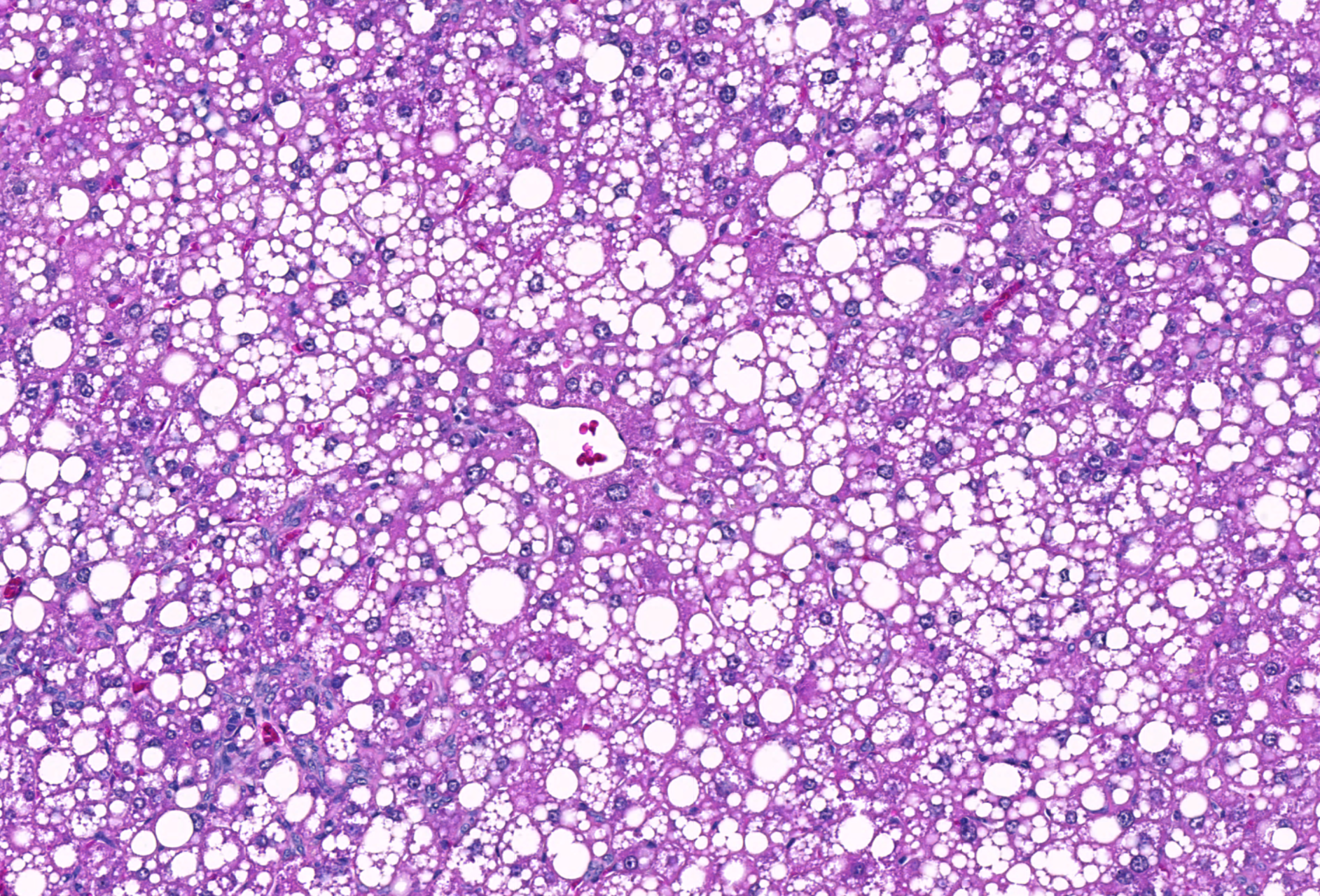

Supplement: Supplementary file 5 — Original histology pictures. [file 42255_2022_617_MOESM5_ESM.zip › Liver_Tesaglitazar.TIF]

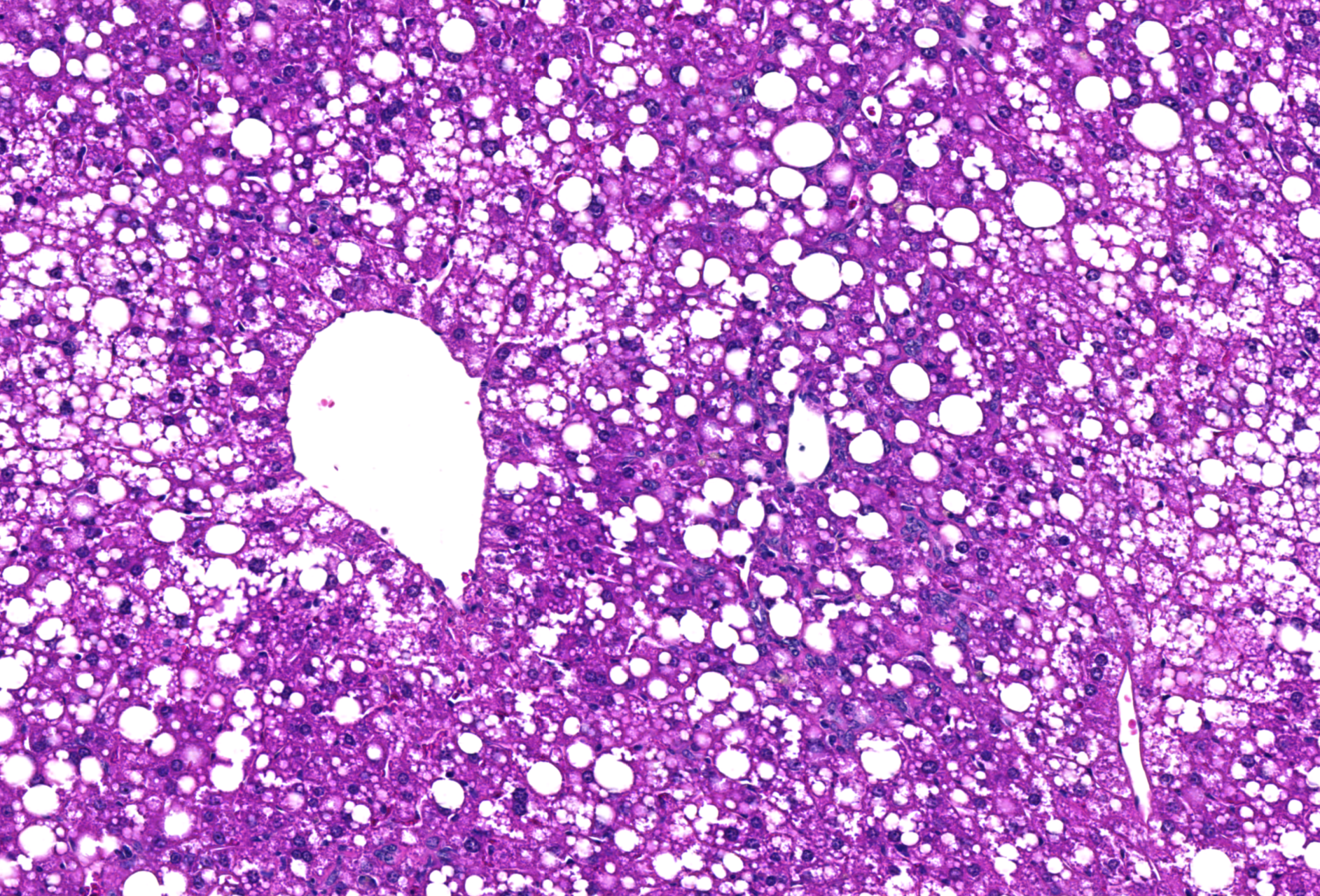

Supplement: Supplementary file 5 — Original histology pictures. [file 42255_2022_617_MOESM5_ESM.zip › Liver_Vhcl.TIF]
